# Transcriptomic profiling reveals p53 as a key regulator of doxorubicin-induced cardiotoxicity

**DOI:** 10.1038/s41420-019-0182-6

**Published:** 2019-06-12

**Authors:** K. Melodi McSweeney, William P. Bozza, Wei-Lun Alterovitz, Baolin Zhang

**Affiliations:** 1 Office of Biotechnology Products, Center for Drug Evaluation and Research, Food and Drug Administration, Silver Spring, MD 20993 USA; 2 High-performance Integrated Virtual Environment, Center for Biologics Evaluation and Research, Food and Drug Administration, Silver Spring, MD 20993 USA

**Keywords:** Predictive markers, Chemotherapy

## Abstract

Doxorubicin is an important anticancer drug in the clinic. Unfortunately, it causes cumulative and dose-dependent cardiotoxic side effects. As the population of cancer survivors who have been exposed to treatment continues to grow, there is increased interest in assessing the long-term cardiac effects of doxorubicin and understanding the underlying mechanisms at play. In this study, we investigated doxorubicin-induced transcriptomic changes using RNA-sequencing (RNAseq) and a cellular model comprised of human induced pluripotent stem cell-derived cardiomyocytes (hiPSC-CMs). Analyses of predicted upstream regulators identified the p53 protein as a key regulator of transcriptomic changes induced by doxorubicin. Clustering and pathway analyses showed that increased death receptor (DR) expression and enrichment of the extrinsic apoptotic pathway are significantly associated with doxorubicin-induced cardiotoxicity. Increased expression of p53 and DRs were confirmed via immunoblotting. Our data pinpoints increased DR expression as an early transcriptomic indicator of cardiotoxicity, suggesting that DR expression might function as a predictive biomarker for cardiac damage.

## Introduction

Doxorubicin is the most commonly used member of the anthracycline family, which is widely prescribed for the treatment of various cancer types. However, the clinical use of doxorubicin, and anthracyclines in general, is hindered by cumulative and dose-dependent cardiotoxic side effects, leading to myocardial dysfunction and even heart failure. Given the accumulating population of cancer survivors that have been exposed to treatment as children or adults, the potential long-term cardiac risk has become an interdisciplinary point of interest. While it remains unclear how exactly anthracyclines cause cardiotoxicity, several mechanisms have been shown to contribute to the development of cardiac damage, including the generation of reactive oxygen species (ROS)^1–4^, inhibition of topoisomerase II^5^, and disruption of calcium ion channel function^6–8^. Most of the reported mechanistic data were derived from rat or mouse models. These animal models often do not fully or accurately recapitulate doxorubicin-induced cardiotoxicity in humans due to inter-species differences in both drug metabolism and cardiac structure and function. In particular, significant differences between mouse and human cardiac electrophysiology and contractility limit the extrapolation of findings from studies in murine systems to humans^9–11^.

Human induced pluripotent stem cell-derived cardiomyocytes (hiPSC-CMs) were recently introduced for assessing drug-induced cardiac toxicity^12,13^. These hiPSC-CMs functionally express most of the ion channels and sarcomeric proteins found in adult human cardiomyocytes and can spontaneously contract, serving as a species-specific complimentary platform to existing animal models. Using this model, we recently identified the upregulation of death receptor (DR) expression and subsequent DR-mediated apoptosis as a potential mechanisms for doxorubicin-induced cardiotoxicity^13^. However, given the complexity of cardiotoxicity, it is most likely that several mechanisms synergize to result in cardiac damage^14^. Influenced by several lines of evidence that point to the role of altered gene and protein expression resulting in apoptosis as an important early mechanism^15–17^, we used a global transcriptomic approach, RNA-sequencing (RNAseq), to broadly assess the impact of doxorubicin on human cardiomyocytes. Such an approach may shed light on previously unrecognized aspects of cardiotoxicity and help to develop biomarkers to predict cardiac risks in individual patients and to develop clinical cardioprotective strategies.

The goals of this study were to use hiPSC-CMs to identify early transcriptomic signatures of doxorubicin-induced cardiotoxicity, to highlight novel pathways upstream of those previously proposed, and to further explore the DR-mediated apoptosis hypothesis. Our results confirm the role of DR-mediated apoptosis as an early response to doxorubicin and describe the role of p53 as a central mediator of DR upregulation and subsequent activation of apoptosis in hiPSC-CMs.

## Results

### Characterization of hiPSC-derived cardiomyocytes

**Fig. 1:**
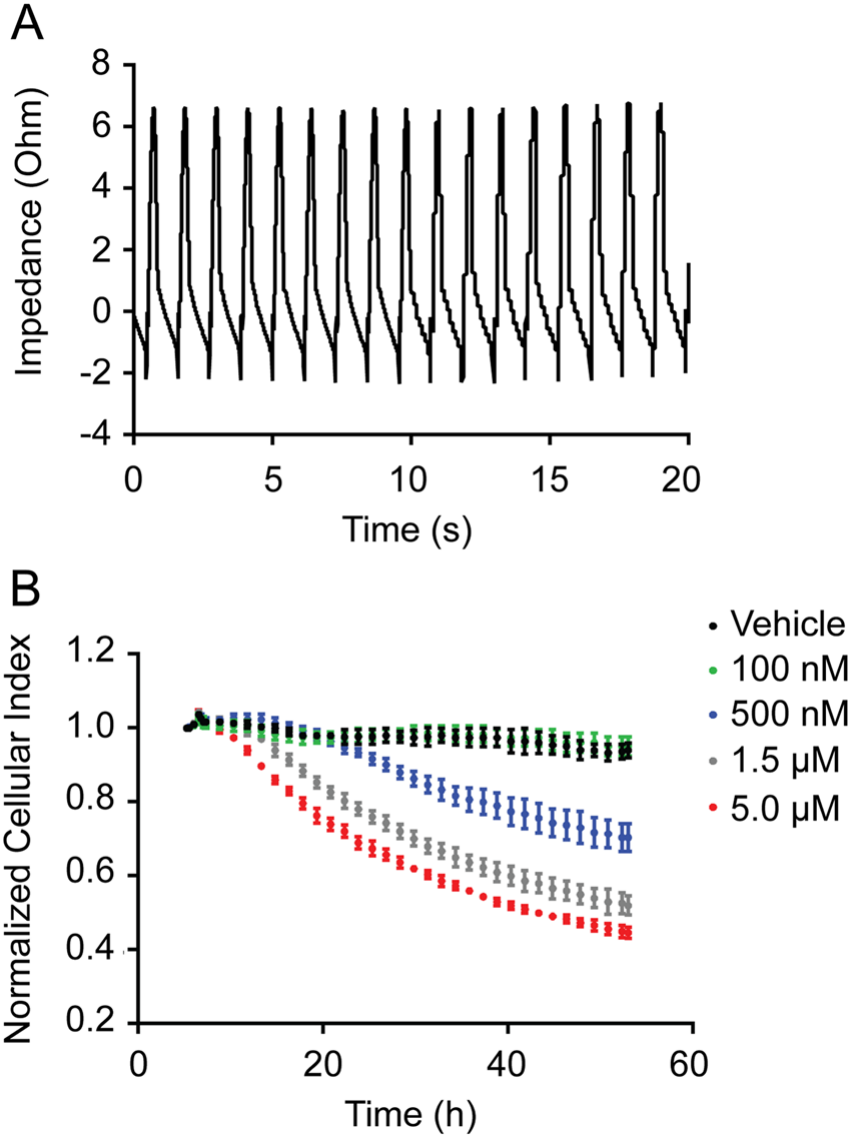
Cardiomyocyte beating profile and cellular index. Cardiomyocytes were cultured onto CardioExcyte 96 sensor plates for 5 days before impedance measurements were recorded. **a** 20 s beating profile of healthy untreated cardiomyocytes. **b** A dose-dependent decrease in cellular index was observed when cardiomyocytes were treated with doxorubicin at the indicated concentrations. Shown are representatives of triplicate measurements

hiPSC-derived cardiomyocytes (iCell Cardiomyocytes^2^) were purchased from Cellular Dynamics International (CDI). The cardiomyocyte differentiation protocol has been validated and extensively shown to produce cardiomyocytes of high quality and purity^18^. Flow cytometry analyses of cardiomyocytes stained for cardiac troponin-T (cTNT) demonstrated a >99% pure population^18^. The manufacturer (CDI) performs batch release analysis and provides Certificate of Testing results that demonstrate cardiomyocyte identity, purity, and functionality. Upon receiving iPSC-CMs, we first determined the cardiomyocyte specific beating profile using the CardioExcyte 96 impedance-based platform. The results revealed a consistent beating profile for all the batches used in this study (Fig. 1a). An impedance value measurement is generated for each well by instrument initiation of a small excitation voltage, resulting in a current between a sensing and reference electrode^19^. Impedance of this current is caused by the adherent cardiomyocyte monolayer. Cardiomyocyte contraction changes the impedance baseline which allows measurement of the beating profile. The impedance system was also used to evaluate doxorubicin-induced changes in cellular index, a measure of cardiomyocyte viability, attachment, and morphology (Fig. 1b). Dose-dependent changes in cardiomyocyte cellular index were observed in response to doxorubicin treatment for 48 h. This data was used to select appropriate doses for subsequent RNAseq and immunoblot analyses.

### RNAseq global transcriptome analysis

**Fig. 2:**
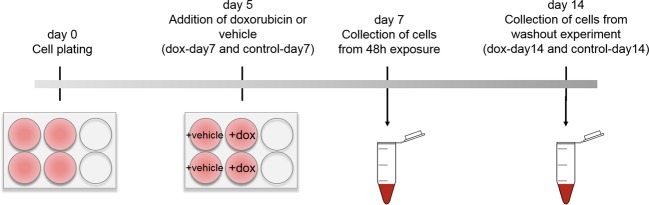
Schematic of experimental design. Cardiomyocytes were cultured onto 6-well cell culture plates for 5 days before the addition of 150 nM doxorubicin (dox-day7) or vehicle (control-day7) for 48 h. RNA was collected on day 7 for groups dox-day7 and control-day7. In a parallel experiment, doxorubicin or vehicle was removed and cells recovered for 7 days (dox-day14 and control-day14) before RNA extraction and RNAseq of all samples

**Fig. 3:**
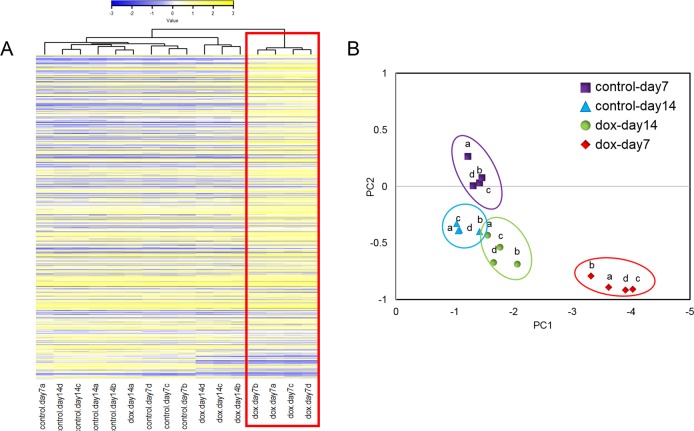
Hierarchical clustering and principal component analyses revealed different expression patterns between treatment groups. **a** Heat map of the sample-to-sample Euclidean distance using the *z*-score values. The red box highlights the clustering of the four replicates (a, b, c, d) of the dox-day7 treatment group separate from all other groups. **b** Principal component analysis of the *z*-scores. The first two principal components (PC1 and PC2) are plotted against each other for each sample. Replicate samples (a, b, c, d) clearly cluster together with dox-day-7 samples separating from the other three groups. Red = dox-day7, purple = control-day7; green = dox-day14, blue = control-day14

We determined transcriptomic alterations in hiPSC-derived cardiomyocytes upon doxorubicin treatment. In brief, cells were plated at high density, according to the manufacturer’s protocol, and exposed to 150 nM of doxorubicin for 48 h on day 5 after plating. The hiPSC-CMs exhibit spontaneous and synchronous beating patterns by day 5, indicating the formation of a functional cardiac network. Previous studies have shown that 48 h exposure to 150 nM doxorubicin is sufficient to induce marked changes in cardiomyocyte gene expression while inducing minimal cellular damage and loss of viability^16,17,20^. Total RNA was extracted from cardiomyocytes on day 7, after 48 h exposure, and on day 14, after a 7-day washout period, and subjected to RNA sequencing (Fig. 2). To determine data reproducibility across replicate samples we performed principal component analysis (PCA) and hierarchal clustering on the top 600 transcripts with greatest variance and with an RPKM > 0 per sample. PCA and hierarchal clustering clearly show that samples exposed to doxorubicin for 48 h cluster separately from the control samples and samples after a 7-day washout of the drug (Fig. 3). Sample clustering show that data is reproducible across biological and technical replicates and were therefore analyzed together.

### Differential expression analysis after 48 h exposure (day 7)

**Fig. 4:**
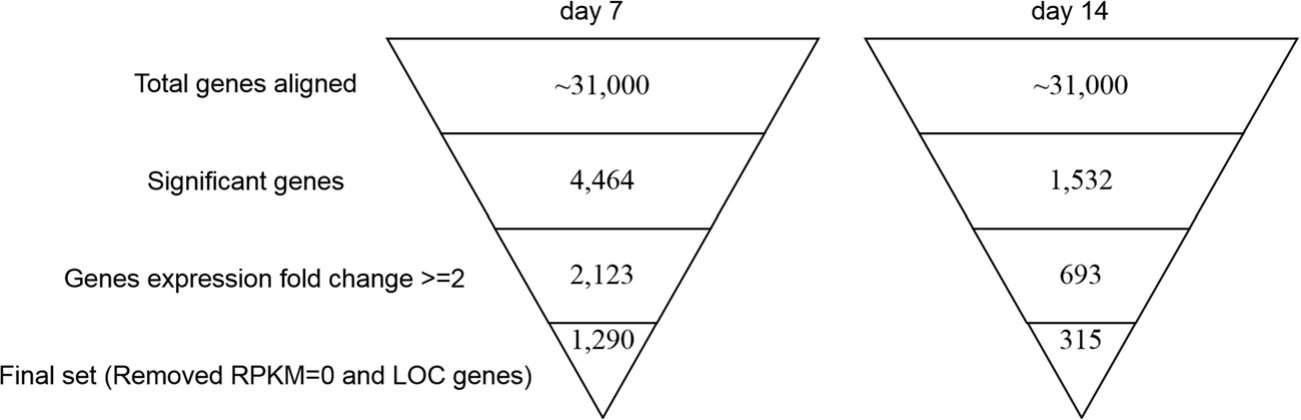
Schematic of analysis and data filtration. The Regularized Linear Discriminant Analysis (RLDA) algorithm was used to determine significant differential expression. Well-established gene transcripts (no predicted gene transcripts, gene names starting with “LOC”) with greater than 0 RPKMs per sample and with greater or equal to 2-fold change in expression between doxorubicin-treated and control groups were used in pathway analyses. On day 7: 1290 genes were dysregulated when compared to control samples. On day 14: 315 genes were dysregulated

**Fig. 5:**
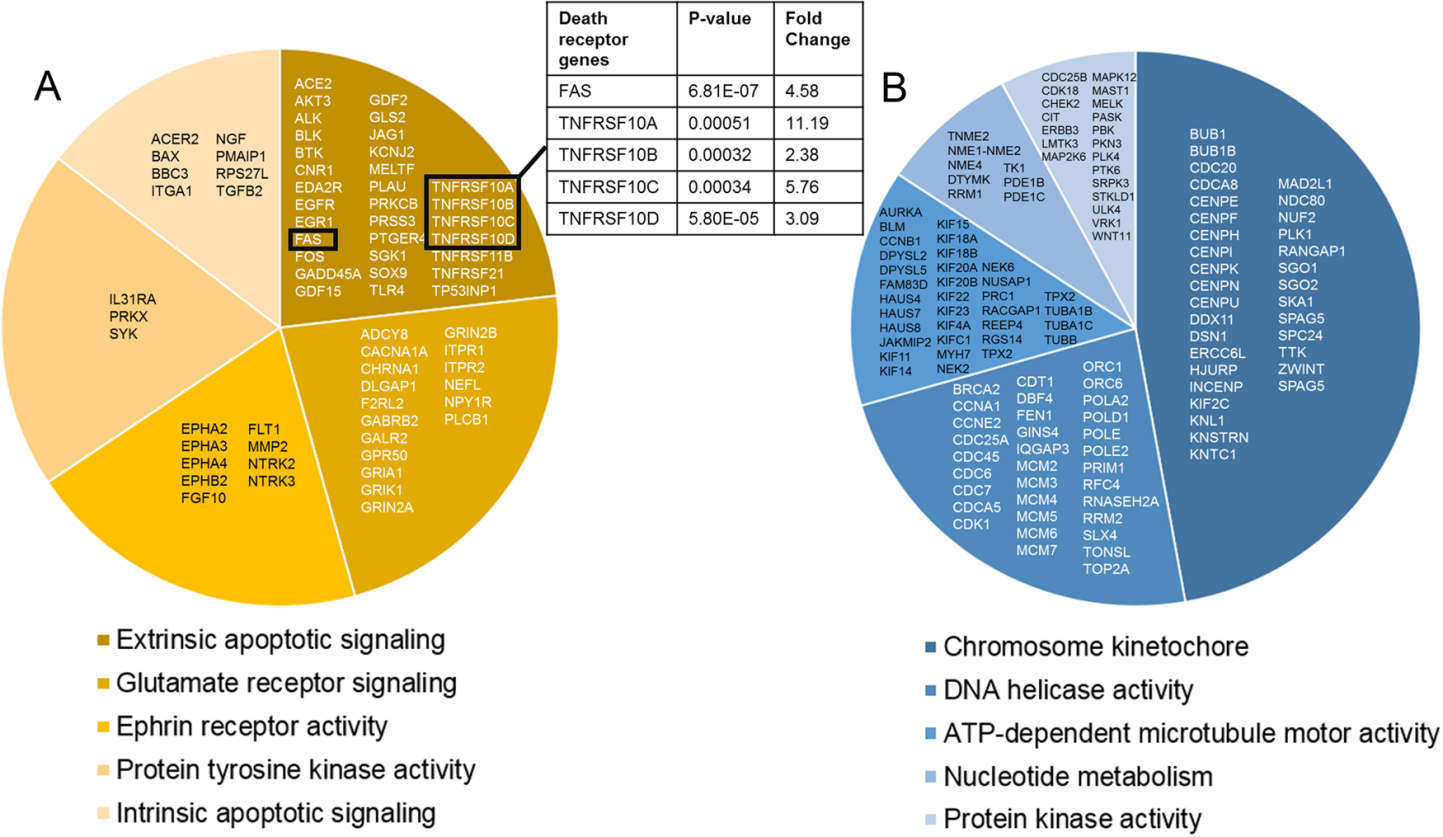
Clustering analysis was performed to identify gene ontology (GO) terms of significantly dysregulated genes. The top five GO clusters are depicted for the **a** upregulated, in yellow, and **b** downregulated genes, in blue. Clusters are listed followed by the enrichment score calculated by DAVID, which is used to determine the percentage of pie chart. The darker the color, the more enriched the cluster. The genes per cluster are listed within the given pie slice. Inset: The death receptors (*FAS, TNFRSF10A, TNFRSF10B, TNFRSF10C*, and *TNFRSF10D*) are among the most significantly upregulated genes in the most enriched cluster

After 48 h exposure to doxorubicin, 1290 genes were significantly dysregulated (Fig. 4), 694 of which increased and 596 of which decreased in expression in comparison to the control group (Supplemental Table 1). To highlight gene clusters, the upregulated and downregulated gene sets were analyzed separately using DAVID (Supplemental Table 1). The extrinsic apoptotic pathway was the most significant gene ontology (GO) cluster among upregulated genes, highlighting an important role for the genes involved in apoptotic signaling at the plasma membrane (Fig. 5a). Within this cluster, *FAS, TNFRSF10A* (DR4), *TNFRSF10B* (DR5)*, TNFRSF10C* (decoy receptor 1), and *TNFRSF10D* (decoy receptor 2) are all upregulated. We’ve previously shown that increased expression of these proteins was associated with cardiotoxicity in hiPSC-CMs^13^. This data provides additional evidence to support the DR-mediated apoptosis hypothesis.

**Fig. 6:**
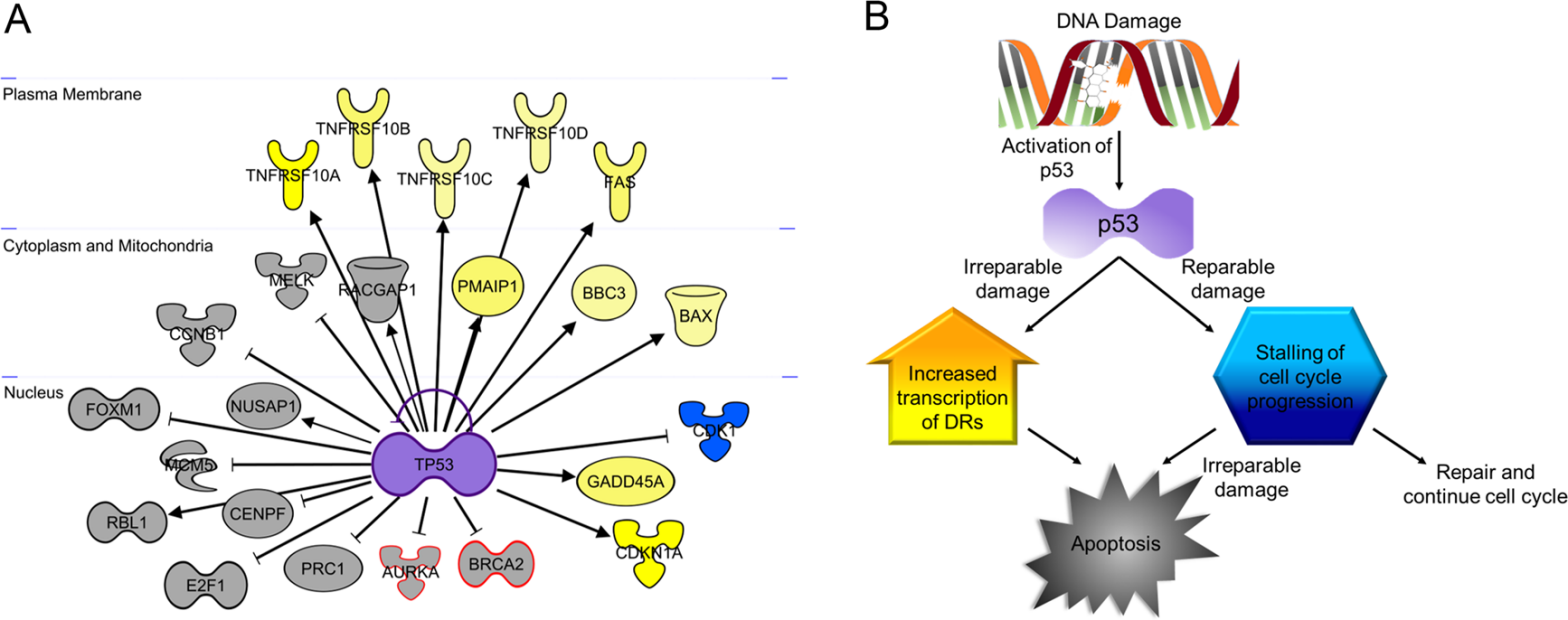
*TP53* is the central mediator of transcriptomic changes associated with acute doxorubicin exposure. **a** The transcription factor encoded by *TP53* increases or decreases the transcription of genes involved in apoptotic (*TNFRSF10A, TNFRSF10B, TNFRSF10C, TNFRSF10D, FAS, PMAIP1, BBC3, BAX*) and cell cycle progression pathways (*CDK1, GADD45A, CDKN1A*), respectively. Genes in gray depict overlap with Reyes et al., genes depicted in gray with red outline indicate diffirent isoforms of genes identified in Reyes et al.; Yellow = increased expression, Blue = decreased expression. **b** Damage caused by interaction of doxorubicin into the DNA activated p53, which then increases transcription of DRs and stalls cell cycle progression. If the damage is deemed irreparable by DNA repair mechanisms, apoptosis is triggered

Pathways involved in cell cycle progression and cell division are among the most enriched pathways in the downregulated gene set (Fig. 5b), and includes the *CDK1*, *CDKN1A*, and *GADD45A* genes (Fig. 6a). Mature cardiomyocytes have limited ability to divide. Given that hiPSC-CMs are functionally more similar to immature cardiomyocytes^21^, hiPSC-CMs may retain some capacity to divide^22,23^. This data indicates that mechanisms involved in cell division are halted by reductions in expression of cell cycle progression genes.

The entire gene set, including expression data, was then uploaded to Ingenuity Pathway Analysis (IPA) for core analysis. The top upstream regulator was predicted to be *TP53*, the gene encoding p53, with a *p*-value of 1.28 × 10^−38^. The second likely upstream regulator was predicted to be *CDKN1A*, the gene encoding cyclin dependent kinase inhibitor 1A, also called p21 (*p*-value of 4.43 × 10^−37^). p21 is a target of p53 and is upregulated in this data set (Fig. 6a), indicating that genes regulated by p21 are downstream of p53. It is of note that the *TP53* gene itself is not upregulated in this dataset, as might be expected due to the fact that DNA damage can induce p53 protein stabilization without affecting *TP53* transcription^24,25^. The top three toxicological lists were (1) cell cycle: G2/M DNA damage checkpoint regulation, (2) p53 signaling, and (3) cell death. In the gene set, 217 genes are directly regulated by *TP53*, among which are the death receptors, *TNFRSF10A*, *TNFRSF10B*, *TNFRSF10C*, *TNFRSF10D*, and *FAS* (Supplemental Table 2 and Fig. 6a).

Taken together, this data highlights p53-regulated inhibition of cell cycle progression and intrinsic and extrinsic apoptotic pathways as key mechanisms involved in early cardiotoxicity. p53 is a central mediator of the dysregulated genes (Fig. 6b).

### Differential expression analysis after 7-day washout period (day 14)

To investigate whether the acute responses to doxorubicin were reversible, doxorubicin was removed from cells and cells were allowed to recover for 7 days with regular media replenishment. In comparison to the day 7 data set, only 315 genes were dysregulated (Supplemental Table 3), 84 of which increased and 231 of which decreased in expression in the doxorubicin-treated group compared to the control group.

**Fig. 7:**
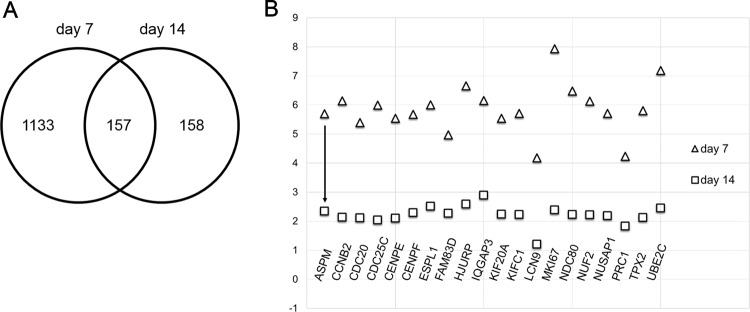
Transcriptomic changes largely do not persist after a period of washout and recovery. **a** Venn diagram showing the overlap between genes dysregulated on day 7 and day 14. **b** Of the 142/157 genes that trend toward baselined, the 20 genes with the greatest absolute fold change in Log2 expression are shown. The arrow indicated the direction of expression fold change returning toward baseline (zero)

The list of genes that were commonly dysregulated on day 7 and on day 14, comprised of 157 genes (Fig. 7a), was subjected to cluster analysis to determine key GO terms. All of the gene clusters identified among the overlapping genes pertained to cell cycle regulation. Furthermore, we found that the absolute value of fold change of 90% (142 out of 157) of the overlapping genes decreased after 7 days of washout, indicating that expression was returning to baseline for most genes (Fig. 7b).

Genes that were not common between days 7 and 14, a total of 158 with 44 increased and 114 decreased, were further analyzed. Most of the genes within both the upregulated and downregulated gene sets did not cluster together, indicating that there was little relationship between the genes and likely no significant role of any pathway in recovery from doxorubicin exposure. Regulation of transcription was the only common cluster among genes, indicating the activation of compensatory mechanisms to restore gene expression patterns to baseline.

### Verification data: western blot confirmation of RNAseq data

**Fig. 8:**
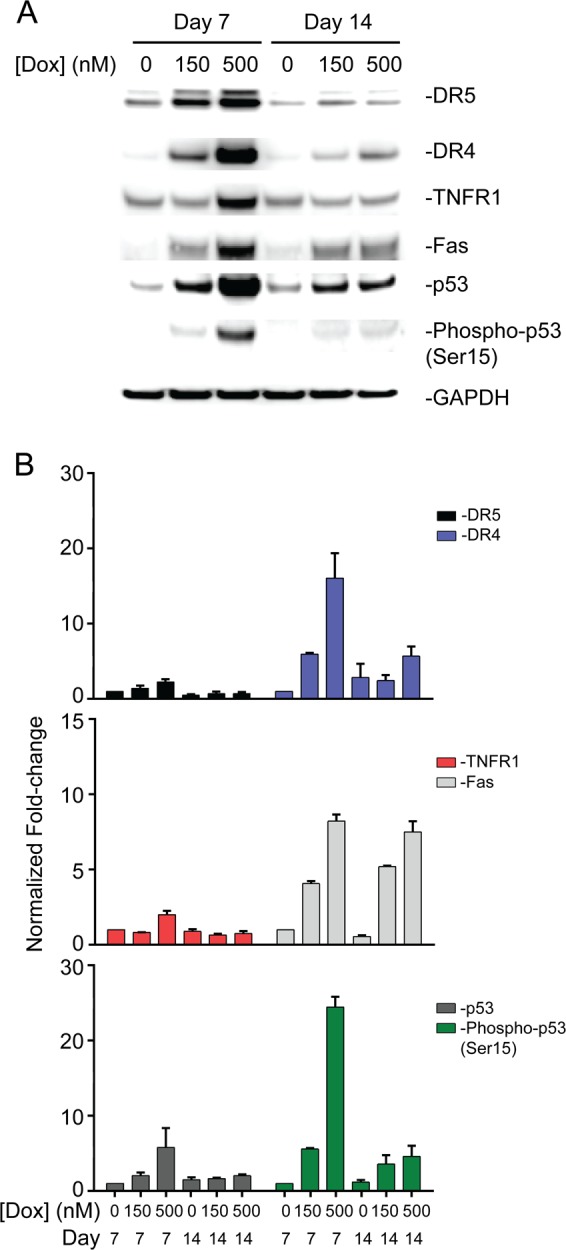
Western blotting confirmed doxorubicin-induced increase in expression of p53 and DRs. **a** Immunoblot and **b** densitometric quantification demonstrate after exposing cardiomyocytes to 150 nM or 500 nM of doxorubicin for 48 h the phosphorylation of p53 and total expression of p53, TNFR1, DR4, DR5, and Fas increase. TNFR1 protein expression increased only after exposure to 500 nM of doxorubicin. The protein expression of p53 targets, DR4 and DR5, decrease after a 7-day washout period. Fas remains slightly upregulated after 7-day washout

We performed immunoblotting to confirm changes observed in RNAseq data analysis. We show that total p53 protein expression as well as p53 phosphorylation are increased in response to 48 h exposure to doxorubicin (Fig. 8), supporting the RNAseq data analysis and identification of p53 as a key upstream activator of gene expression changes. The expression levels of DR4, DR5, and Fas were also probed, showing increased expression of these protein products after doxorubicin treatment. Furthermore, the expression of these proteins decreased after a 7-day washout period – with the exception of Fas, corroborating RNAseq data. Fas mRNA expression decreased (Supplemental Table 1 and 3) but did not return to baseline on day 14 whereas the Fas protein remained slightly upregulated after the 7-day washout (Fig. 8). The expression of TNFR1, previously shown to be increased in response to high doses of doxorubicin^13^, did not increase in response to the low dose of 150 nM of doxorubicin but did increase at the higher dose of 500 nM. This data is consistent with the RNAseq data showing no increase in mRNA expression of *TNFR1* and supports our previously published data^13^.

## Discussion

Doxorubicin is an effective chemotherapeutic drug that is prescribed for the treatment of numerous cancer types. Due to the known cumulative and dose-dependent cardiotoxic side effects of doxorubicin, there is a growing interest in assessing the long-term cardiac effects of doxorubicin on patients previously exposed to treatment. Current measures of cardiac damage include evaluation of left ventricular ejection fraction (LVEF) and circulating troponin concentration. Cardiac damage can cause a decline in the LVEF measurements, indicating a decrease in the heart’s ability to pump blood, and an increase in troponin, a cardiac protein that is released into circulation upon heart tissue damage. Both measurements identify patients who have already begun to experience cardiotoxic side effects and are often observed after when the damage has progressed to be irreversible. To date, there are no predictive biomarkers that can determine a patient’s risk before the onset of heart damage. Consequently, understanding the molecular mechanisms that result in cardiotoxicity is important to pinpoint pathways that may be predictive of heart damage.

Mechanistic evaluation of how cardiotoxicity occurs is now more accessible due to increasing availability of human induced pluripotent stem cell-derived cardiomyocytes (hiPSC-CMs). hiPSC-CMs provide a cellular model that more closely recapitulates features of the human heart than previously used animal models and can also be used to screen for drug-induced alterations in cardiac cellular contractility, electrophysiology, and viability, making them a particularly appealing model to investigate doxorubicin-induced cardiotoxicity. In this study, we used a transcriptomic approach to evaluate the early gene expression signatures of cardiomyocyte exposure to doxorubicin and discover potential biomarkers of cardiotoxicity.

We exposed hiPSC-CMs to a low dose of doxorubicin for 48 h. Previous studies have indicated that this dose and exposure time are sufficient to induce transcriptomic changes while sparing cells from widespread toxicity^13,16,17,26^. We found that over a thousand genes were significantly dysregulated after 48 h. Gene ontology (GO) analysis revealed that the most significantly enriched GO terms among the downregulated genes pertained to cell cycle progression and cell division (Fig. 5b). When cells are exposed to DNA damaging agents, two pathways can be initiated. The first is stalling of the cell cycle, which causes DNA repair processes to correct minor DNA damage and restore genomic stability^27^. If the damage cannot be repaired, then apoptosis is initiated (Fig. 6b). It follows that genes involved in cell cycle progression are downregulated in the presence of the potent DNA damaging agent doxorubicin. However, this observation is somewhat surprising as mature cardiomyocytes are terminally differentiated and do not divide. It is important to note here that stem-cell-derived cardiomyocytes are more characteristically similar to immature cardiomyocytes^21^, which maintain the capacity to divide. Meaning that, in our cellular system, cells that are capable of dividing might be halted in the presence of doxorubicin. Others have suggested that cell cycle genes can be downregulated in cells that have initiated apoptosis even if those cells were not undergoing active cellular replication^28,29^. Although additional research is warranted to confirm the role of cell cycle stalling on the development of cardiotoxicity, it is plausible that this finding might have broad implications for the impact of doxorubicin on the developing heart, such as when administered to juvenile patients^30,31^.

The most significantly enriched term among the upregulated genes was the extrinsic apoptotic pathway (Fig. 5a). We have previously shown that acute exposure to doxorubicin induces the expression of death receptors (DRs) and proposed that this novel mechanism results in spontaneous or ligand-dependent apoptosis^13^. In this study, we confirmed that *FAS*, *TNFRSF10A* (DR4), and *TNFRSF10B* (DR5) are among the most significantly upregulated genes and are therefore major signatures of early cardiac damage induced by doxorubicin (Fig. 8). DRs are cell surface receptors of the TNF-receptor superfamily that transmit apoptosis or necroptosis signals in target cells upon ligation with their cognate death ligands, including TNFα, Fas ligand or TNF-related apoptosis-inducing ligand (TRAIL). These TNF-related cytokines circulate in the blood stream and are secreted by most normal tissue cells as an innate immune response. Numerous studies have reported that the concentration of circulating TNF cytokines is increased in some cancer patients and has been linked to progression of malignancy^32–35^. Taking into consideration our observation of increased DR expression in response to acute exposure to doxorubicin, it is likely that patients with increased cytokine levels at the time of treatment may be at a higher risk of developing cardiotoxicity.

**Fig. 9:**
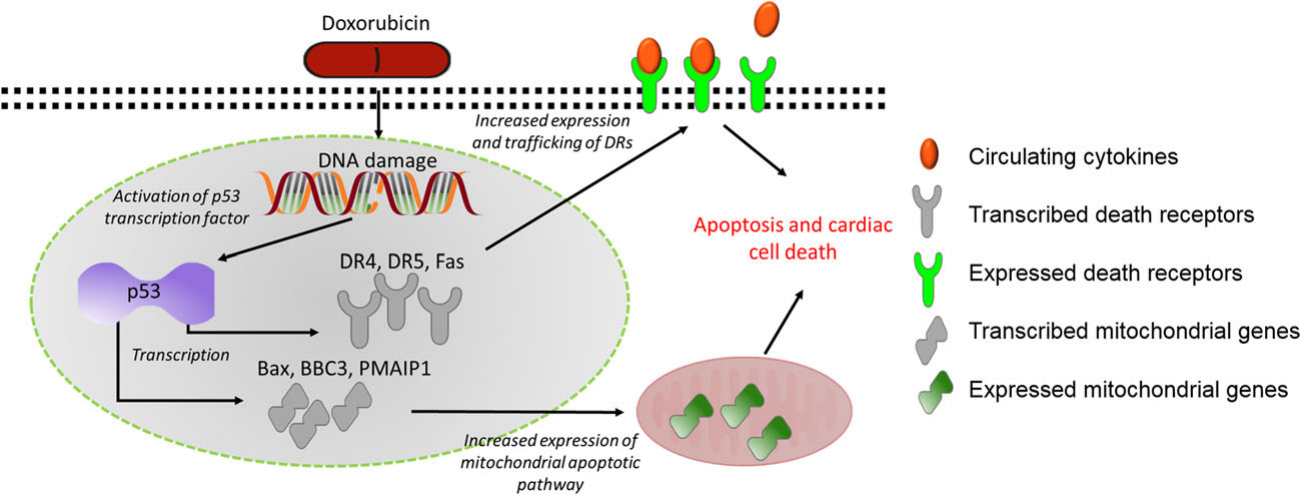
Proposed mechanism for doxorubicin-induced cardiotoxicity. Doxorubicin, a DNA intercalating agent, induces DNA strand breaks and triggers the activation of p53 to initiate DNA damage responses. Activation of p53 induces increased transcription of genes involved in the extrinsic and intrinsic apoptotic pathway. In the presence of circulating ligands, the extrinsic apoptotic pathway is activated

We next identified an upstream regulator that controls both the induction of DR expression and apoptotic pathways and the repression of cell cycle progression. Pathway analysis of the 1290 genes identified the *TP53* gene as the most likely upstream regulator (Fig. 6a)^28,29,36,37^. The *TP53* gene encodes the p53 protein. p53 has long been recognized as a cell cycle and apoptosis regulator. In fact, *TP53* is the most frequently mutated gene in human cancers^38,39^, rendering the gene inactive and resulting in chemo-resistance to chemotherapies that both halt cell cycle progression and trigger apoptosis via the p53 pathway. Cardiac cells are often spared from these mutations. Expression of wild-type-p53 leaves cardiac cells susceptible to p53 activation by DNA damage inducing agents, such as doxorubicin. Upon activation of p53, genes involved in cell cycle regulation and apoptotic pathways are dysregulated. Notably, the second most likely upstream regulator of the dysregulated genes was predicted to be the *CDKN1A* gene, which is downstream of p53 and controls cell cycle progression. It has been proposed that downregulation of genes essential to cell cycle progression are an initiation step toward apoptosis^27^, thereby making p53 the central mediator of molecular events that lead to the development of cardiotoxicity (Fig. 9). Although the expression of *TP53* was not upregulated in our data set, protein expression increased after 48 h (Fig. 8). This is not surprising, as DNA damage can induce the stabilization of the p53 protein via the phosphorylation of serine 15 by the *ATM* gene without affecting transcription of the gene^40,41^. However, our data does not imply that p53-dependent mechanisms are the only pathways important in the onset of cardiotoxicity. On the contrary, it is likely that numerous pathways, including previously proposed ROS, calcium channel interference, and topoisomerase inhibition, all work together to damage the heart. Nevertheless, our data not only closely replicate data published by several groups^16,17,20,26^, but also highlight potentially new pathways to explore. For example, the second most highly enriched GO cluster is glutamate receptor signaling (Fig. 5a). Glutamate signaling is an excitatory pathway that elicits cardiac beating and is closely related to processes that depend on calcium. Increased expression of genes within this pathway might indicate overexertion of cardiomyocytes and may lead to cardiac fatigue.

It is important to note that we observed significant overlap between our findings and that of published and independent research^26^. Reyes et al., using different data analysis tools and experimental design, reported p53-dependent activation of apoptotic pathways and inhibition of cell cycle genes^26^. Furthermore, Reyes et al. highlighted the role of twelve p53-dependent genes, ten of which overlap exactly with our data and two of which overlap with different isoforms of the gene, *BRCA1* vs *BRCA2* and *AURKB* vs *AURKA*, in Reyes et al. versus this report respectively (Fig. 6a, gray with red outline and gray, respectively). We also observed similar genes sets as described in other published datasets ^16,17^. The significant overlap between different datasets not only exemplifies the importance of using human cardiomyocytes to identify precise mechanisms, it also fortifies the role of the genes discussed herein as mediators and predictors of doxorubicin-induced cardiotoxicity.

Interestingly, more than 75% of the genes dysregulated on day 7 returned to normal expression levels after a period of recovery involving the washout of doxorubicin for seven additional days. These data support previously published observations that the damage caused by acute exposure to low doses of doxorubicin is mostly reversible in vitro^16,17,26^. Among the genes that were consistently dysregulated on day 14, 90% had decreased in the absolute fold change after the 7-day recovery, indicating a return to baseline expression levels (Fig. 7b). The data implies that, given a longer recovery period, more genes would return to baseline expression levels. However, according to the manufacturer, it is not recommended to culture the hiPSC-CMs beyond fourteen days. Genes that remained dysregulated on day 14 did not cluster or have a common predicted regulator. Lack of clustering illustrates the return to baseline expression levels genome wide.

Importantly, this work identifies, for the first time, a set of genes which may function as predictive biomarkers of cardiotoxicity. The upregulation of DRs and subsequent increased protein expression that occurs after 48 h exposure to doxorubicin (Fig. 8) can cause receptor clustering and trigger the activation of apoptosis^42^. Conversely, we have shown that doxorubicin-induced apoptosis is enhanced in the presence of death ligands such as TNF-related apoptosis-inducing ligand (TRAIL)^13,43^. This may be especially important in a clinical setting where patients with high levels of circulating cytokines during treatment may be at an increased risk of developing doxorubicin-induced cardiotoxicity. It follows that assessment of circulating cytokines can help determine at-risk patients and dictate the course of treatment in a clinical setting.

In conclusion, using hiPSC-CMs to model cardiotoxicity in vitro, we have shown that doxorubicin induces the expression of DRs through stabilization of the p53 protein. The early increase in DR mRNA and protein expression highlight a novel implication for prediction of cardiotoxic risk in patients undergoing treatment. Furthermore, the development of therapeutics such as monoclonal antibodies against death ligands may mitigate the cumulative cardiotoxic side effects of anthracyclines, such as doxorubicin, when used as combination therapies.

## Materials and methods

### Cell culture

Human induced pluripotent stem cell-derived cardiomyocytes (hiPSC-CMs) were purchased from Cellular Dynamics International (CDI Madison, WI). Cells were cultured according to the protocol provided for iCell Cardiomyocytes^2^. In brief, 6-well cell culture plates were coated with 50 µg/ml fibronectin solution (Sigma Aldrich, St. Louis, MO, cat no: F1141) overnight at 37 ^°^C. The following day, cells were gently thawed, resuspended in Plating Media, counted to determine cell number, and plated onto fibronectin coated plates at a concentration of 1.56 × 10^5^ cells/m^2^ immediately after aspiration of the fibronectin solution (*n* = 4 per treatment group). Cells were incubated at 37 °C for 4 h before the Plating Media was replaced with Maintenance Media (both provided by CDI). A 100% media change was performed every other day (days 2, 5, 7, 9, 11, and 13). On day 5 after cell plating, cells were treated with doxorubicin or vehicle for 48 h.

### Cardiomyocyte beating data recording and analysis

50,000 cells were seeded per well of a CardioExcyte 96 sensor plate (Nanion, Livingston, NJ, cat no: 20-1001) and were cultured as described above. 20 s cardiomyocyte beating profiles and cellular index measurements (Fig. 1) were recorded using the impedance mode of the Cardio-Excyte 96 system (Nanion, Livingston, NJ). The cellular response to doxorubicin treatment was recorded every 90 min.

### Total RNA isolation and quantification

Cells were treated with 150 nM doxorubicin (Sigma Aldric, St. Louis, MO, cat no: D1515) on day 5 for 48 h (*n* = 8) or treated with the vehicle (control), which is comprised of 0.0015% DMSO (Sigma Aldric, St. Louis, MO, cat no: 276855) in culture media in the control group (*n* = 8). On day 7, cells were collected from the doxorubicin-treated (dox-day7, *n* = 4) and control group (control-day7, *n* = 4) using a cell-scraper. Cells were centrifuged, and flash frozen before storage at −80 °C. Cells from the parallel experiment received a 100% media change on day 7 and were maintained for 7 additional days. On day 14, the remaining doxorubicin-treated (dox-day 14, *n* = 4) and control (control-day 14, *n* = 4) cells were collected (Fig. 2).

Cell pellets from doxorubicin-treated cells and control cells were thawed on ice before total RNA isolation. The miRNeasy Micro Kit (Qiagen, Hilden, Germany, cat no: 217084) was used to isolate total RNA as directed by the protocol. RNA was first quantified by UV spectrophotometry (ThermoFisher, Waltham, MA, NanoDrop 2000C) to determine concentration. The RNA was then tested for quality using the Agilent BioAnalyzer RNA Nano Kit. Samples with a RIN of greater than or equal to 8 were used for RNA sequencing (*n* = 16, 8 per group). A total amount of 200 ng of RNA was subjected to paired-end transcriptome wide sequencing at the Center for Biologics Evaluation and Research Next Generation Sequencing Core.

### RNAseq data generation, processing, and analysis

The Illumina TruSeq Stranded Messenger RNA Library Prep Kit (San Diego, CA, cat no: 20020594) was used to construct strand-specific libraries for sequencing. Samples were pooled together for sequencing on the Illumina HiSeq2500 as previously described^44^. Greater than 20 million reads of ~100 bp were generated per sample. Raw reads, fastq files, were aligned to the reference genome GRCh38 using the High-performance Integrated Virtual Environment (HIVE) moderated by the FDA^45^. The Reads Per Kilobase Million (RPKM) matrix files were generated and genes were annotated using the HIVE. Roughly 31,000 gene transcripts were mapped and annotated across all 16 samples. To visualize differences between the doxorubicin-treated and control groups, principal component analysis and hierarchal clustering was performed using the HIVE and the R Heatmap function, for the top 600 transcripts with the greatest variance across entire data set with RPKM values >0 (Fig. 3).

The Regularized Linear Discriminant Analysis (RLDA) algorithm was used to normalize the data and determine differential expression among genes in the doxorubicin-treated versus control groups on days 7 and 14. Gene transcripts with greater than 0 RPKMs per sample, with greater or equal to 2-fold change in expression between doxorubicin-treated and control groups, and with a significance of ≤0.05, as determined by a two-tailed paired *t*-test (Fig. 4), were further analyzed using gene ontology (GO) software.

The final list of genes was analyzed using The Database for Annotation, Visualization and Integrated Discovery (DAVID) version 6.8 (david.ncifcrf.gov) and Ingenuity Pathway Analysis software. For DAVID, GO enrichment analyses for upregulated and downregulated genes were performed separately against the *H. sapiens* proteome. For IPA analyses, the Log2 fold change was used to interpret gene expression of the entire gene list using default settings.

### Immunoblotting

To validate RNAseq findings, select proteins were analyzed by immunoblot (Fig. 8). Cardiomyocytes were cultured on 6-well plates at the recommended density, as previously described. On day 5, cardiomyocytes were exposed to 150 nM doxorubicin, 500 nM doxorubicin or vehicle control. On day 7, after 48 h of exposure to doxorubicin, cells were washed and lysed in RIPA buffer and spun down to pellet cellular debris. Cell free lysate was quantified using the Pierce^TM^ BCA Protein Assay Kit (ThermoFisher, Waltham, MA, cat no: 23225) and 30 ug of protein was used for immunoblotting using NuPAGE^TM^4-12% Bis-Tris Protein cells (ThermoFisher, Waltham, MA, cat no: NPO322PK2). Dilutions of each antibody were made in 5% Bovine Serum Albumin (Sigma Aldric, St. Louis, MO, cat no: A7906). A 1:2000 dilution of anti-p53 antibody (R&D Systems, Minneapolis, MN, cat. no: HAF1355) was used and a 1:1000 of each of the following antibodies was used: anti-phosphorylated-S15-p53 (Cell Signaling Technology, Danvers, MA, cat. no: 9286); anti-DR4 (Cell Signaling Technology, Danvers, MA, cat. no: 42533); anti-DR5 (Cell Signaling Technology, Danvers, MA, cat. no: 8074); anti-Fas (Santa Cruz Biotechnology, Dallas, TX, cat. no: sc-715); anti-TNFR1 (Cell Signaling Technology, Danvers, MA, cat. no: 3736). Immunoblots were imaged and quantified using Immobilon Western Chemiluminescent HRP Substrate (Millipore) and an ImageQuant LAS 4000 imager (GE). Protein expression levels were quantified by densitometry analysis of immunoblots using the imager built-in software and were normalized to the corresponding GAPDH (Novus Biologicals, Centennial, CO, cat. no: 2D4A7) loading controls.

## Supplementary information

Supplemental Table 1

Supplemental Table 2

Supplemental Table 3

Supplemental Table 3-part 2

Supplemental Material File #1
