# Supplementary material for: Transcriptomic profiling reveals p53 as a key regulator of doxorubicin-induced cardiotoxicity
Source: Cell Death Discov. 2019 Jun 12;5:102. doi: 10.1038/s41420-019-0182-6 (PMC6561911; doi:10.1038/s41420-019-0182-6)
Supplement: Supplementary file 1 — Supplemental Table 1 [file 41420_2019_182_MOESM1_ESM.pdf]

All 1290 dysreg. genes on day 7										
			Log 2 values per sample: red = upregulated; green = downregulated							
Gene Name	P-Value	Fold Change	dox-day7-a	dox-day7-b	dox-day7-c	dox-day7-d	control-day7-a	control-day7-b	control-day7-c	control-day7-d
A2M	0.00141964	3.054487179	6.617651119	6.977279923	6.350497247	6.238404739	5.129283017	4.87774425	4.672425342	5.125155131
ABALON	0.0153293	2.058394161	1.851998837	1.570462931	1.189033824	1.263034406	0.948600847	0.757023247	-0.913216234	0.40053793
ABCB1	0.00192435	5.127701375	-1.77102743	-1.671163536	-1.77102743	-2.795859283	-5.184424571	-4.205563338	-4.075824085	-4.002310161
ABCC11	0.00321327	2.495543672	-3.365871442	-2.531156057	-2.708396442	-2.836501268	-4.506352666	-3.662003536	-4.221623189	-4.392894616
ABCC6	0.00316169	0.482631579	2.879705766	3.142413438	3.215678597	3.485426827	4.392317423	4.510961919	3.807354922	4.193771743
ABCC6P1	0.00151835	0.377256318	-2.54793177	-2.60823228	-2.190997225	-1.826232932	-0.606034724	-0.75389599	-1.347398782	-0.805912948
ABCC6P2	0.00309154	0.231612903	-1.971430848	-1.98279071	-1.164884385	-1.049904906	0.150559677	0.941106311	1.021479727	0.214124805
ABRA	1.08E-05	4.582441113	1.056583528	1.117695043	1.263034406	0.925999419	-0.997117491	-1.883635243	-0.985644707	-0.76121314
ACAT2	0.0374416	0.442537313	3.283921772	2.769771739	1.722466024	1.952333566	4.292781749	3.510961919	3.827819025	3.075532631
ACBD7	0.0117199	0.392631579	-3.74081792	-3.801883071	-3.494922085	-3.98165069	-2.506352666	-3.224317298	-2	-2.13289427
ACE2	0.000672688	3.622047244	0.565597176	0.799087306	0.263034406	0.137503524	-1.514573173	-1.055891201	-2.035046947	-1.158429363
ACER2	0.000127512	8.181818182	2.66448284	2.09085343	2.121015401	2.280956314	-0.428565884	-0.295128036	-2.40354186	-0.57132159
ACHE	0.0330062	3.195652174	3.089159132	3.498250868	2.480265122	1.992768431	0.956056652	2.23878686	0.86393845	-0.45600928
ACMSD	0.00379616	4.345132743	-0.788364747	-1.224317298	-0.652901329	-1.648371671	-3.79385693	-2.988504361	-4.943416472	-2.184424571
ACOT7	0.0108743	0.26637931	1.769771739	2.185866545	1.070389328	1.195347598	3.288358562	3.523561956	4.161887682	2.857980995
ACSS1	0.0117676	0.48973607	-2.727379545	-2.795859283	-2.089267338	-2.867752202	-1.415037499	-1.826232932	-1.943416472	-1.164884385
ACTA2	0.00601991	2.005571031	5.970393538	6.475733431	6.261154673	5.899659026	4.765534746	4.882643049	5.689299161	5.141596278
ACTN3	0.000499259	0.075411765	-4.947862377	-5.088040035	-3.426625474	-3.293358943	-0.340075442	-0.516635639	0.250961574	-0.467932448
ADAM12	0.00967968	2.963576159	-2.365871442	-1.971430848	-2.746615764	-3.070966521	-3.537842952	-3.789860543	-4.895394957	-4.333516069
ADAM20	0.00807963	2.405128205	-4.658355759	-4.232429944	-4.74081792	-4.127841043	-6.200249538	-5.327710447	-6.805509453	-5.020925839
ADAMTS1	0.000138323	3.308988764	2.613531653	2.625270489	2.500802053	2.482848283	1.269033146	-0.659722595	0.555816155	1.378511623
ADAMTS10	0.000333462	0.250704225	-1.092340172	-1.067938829	-0.875671865	-0.63039393	0.695993813	1.411426246	1.10433666	1.056583528
ADAMTS12	0.000107726	0.460992908	0.970853654	1.056583528	0.956056652	0.871843649	2.263034406	2.028569152	1.859969548	2.140778656
ADAMTS17	0.00757797	6.25698324	1.384049807	1.761285273	0.782408565	0.310340121	-1.418889825	-1.104697379	-1.841662973	-1.671163536
ADAMTS8	0.0207197	2.190082645	0.250961574	0.516015147	-0.148800661	-0.477944251	-1.430508908	-0.401634795	-1.634867407	-1.046921047
ADAMTSL1	0.00333831	2.283783784	1.778208576	2.13422094	1.521050737	1.500802053	0.650764559	0.632268215	-0.013043037	0.86393845
ADAMTSL5	0.0284639	0.468361582	2.853995647	2.965322548	3.241840184	3.117695043	3.837943242	4.716990894	4.240314329	3.510961919
ADCY8	0.013691	7.590206186	-0.212567535	-0.289827252	-1.418889825	-1.732164608	-3	-3.120294234	-5.232429944	-4.526161147
ADD2	0.0247161	0.359570662	0.839959587	0.584962501	1.014355293	1.443606651	2.9202933	2.241840184	1.40599236	2.887525271
ADGRG6	0.0186997	2.101876676	3.203201156	3.272023189	2.807354922	2.454175893	1.726831217	2.531069493	0.815575429	2.017921908
ADGRV1	0.00147661	4.647435897	-0.76121314	-1.254977851	-1.407363571	-1.49410907	-3.861447625	-3.23786383	-5.259806383	-2.522840789
ADH6	0.00320575	2.492753623	-2.671163536	-2.64385619	-2.114035243	-2.836501268	-3.874084451	-3.649276466	-3.624154275	-4.40506933
ADRA2B	0.0215594	0.431304348	-5.687799537	-5.380821784	-3.965784285	-3.456405136	-3.070966521	-3.477140745	-3.279283757	-2.756330919
AEN	7.12E-06	3.317919075	5.951867504	5.703211467	5.8899602	5.815063017	4.459431619	4.087462841	3.711494907	4.09592442
AFF1-AS1	0.00904591	0.476021314	-2.307572802	-2.126580497	-2.114035243	-1.282789701	-0.655171503	-1.304006187	-0.910501849	-0.55851652
AFF2	0.00025476	4.595744681	-0.428565884	-0.40354186	-0.736965594	-1.023269779	-2.490050854	-3.429731384	-3.023269779	-2.53951953
AGAP2	0.0345174	2.476363636	-3.977099598	-3.456405136	-3.954557029	-4.227016448	-6.77675046	-5.803896602	-3.952322025	-5.764150423
AHNAK2	0.00965389	3.23782235	0.604071324	0.475084883	-0.351074441	-0.251538767	-1.058893689	-0.897006007	-2.634867407	-2.184424571
AKAP11	0.0190588	2.027027027	4.03562391	3.364572432	3.689299161	3.847996907	3.111031312	2.253989266	1.521050737	3.378511623
AKAP5	0.00152014	2.041036717	-2.272297327	-2.481968507	-2.23786383	-2.652901329	-3.66383089	-3.541198058	-3.717856771	-2.943416472

AKR1B1	0.0093365	2	9.162391329	9.321928095	8.714245518	8.54303182	7.813781191	8.108524457	8.214319121	7.672425342
AKR1B10	0.00126567	4.642201835	-4.840629153	-3.993091631	-4.068543859	-4.470728756	-5.993091631	-6.392894616	-8.04739805	-6.345197874
AKT3	0.00401076	2.463601533	2.687060688	2.375734539	2.849999259	2.780310099	1.40599236	0.90303827	0.378511623	2.204766751
ALDH3B1	0.000966809	0.342975207	-1.418889825	-1.948975997	-0.675765438	-1.318325858	0.575312331	0.310340121	-0.002888279	0.137503524
ALDOC	0.0122154	0.386809269	4.277984747	4.217230716	4.472487771	4.738767837	5.719731057	5.66106548	6.375039431	5.251719093
ALK	0.026619	3.394077449	-2.418889825	-2.120294234	-3.279283757	-3.685941591	-4.480357457	-4.563198526	-5.112786697	-4.063710705
AMHR2	0.0293001	0.290540541	2	1.891419187	1.887525271	2.5360529	3.807354922	3.111031312	4.66106548	3.498250868
AMMECR1	0.000404842	2.473498233	0.516015147	0.678071905	0.40053793	0.310340121	-0.720231578	-1.358453971	-1.077041036	-0.336427665
AMPD3	0.000618159	2.157088123	-0.662003536	-0.77349147	-1.074000581	-0.836501268	-2.351074441	-1.514573173	-2.077041036	-1.948975997
ANGPTL2	0.0429085	0.470634921	-0.641603738	-0.910501849	-0.935117148	-0.567040593	0.275007047	0.98550043	0.111031312	-0.378944497
ANK1	0.000323264	3.849693252	4.981852653	4.638073837	4.419538892	4.498250868	3.217230716	2.722466024	2.007195501	2.62058641
ANKRD18A	0.00051233	3.024316109	4.03562391	4.432959407	4.371558863	4.392317423	2.15704371	3.459431619	2.060047384	2.752748591
ANKRD18B	3.07E-05	3.262295082	-0.543719518	-0.394031641	-0.152003093	-0.253257284	-2.224317298	-1.836501268	-1.915935735	-2.204233052
ANKRD20A12P	0.00566829	3.212389381	-5.276485124	-4.506352666	-5.216250017	-4.359934417	-6.687799537	-6.506352666	-6.573466862	-6.13796526
ANKRD20A9P	0.0329567	0.308839779	-8.082163468	-6.680382066	-8.464982232	-7.359342057	-5.448508591	-5.622376462	-7.506352666	-5.392894616
ANKRD34C	0.0111931	2.482269504	-2.888968688	-2.450084446	-3.095419565	-3.023269779	-5.083141235	-3.224317298	-4.803896602	-4.221623189
ANKRD45	1.74E-06	3.661327231	3.99095486	4.070389328	4.044394119	3.87774425	2.153805336	2.313245852	1.655351829	2.289834465
ANLN	0.00714308	0.097460317	1.748461233	1.344828497	1.695993813	1.650764559	4.822730148	3.963474124	4.995484519	5.64385619
ANXA4	0.000220265	2.004008016	4.193771743	4.459431619	4.412781525	4.193771743	2.941106311	3.336283388	3.459431619	3.485426827
AP1S1	0.000718417	2.256410256	1.827819025	1.815575429	1.827819025	1.799087306	0.275007047	0.367371066	1.316145742	0.333423734
APBA1	0.000797291	2.801843318	-0.564904848	-0.522840789	-0.793356776	-1.049904906	-2.064917477	-1.666576266	-2.465938398	-2.910501849
APLP1	0.00549265	5.317829457	6.209453366	6.686500527	5.678071905	5.526694846	3.498250868	4.137503524	3.867896464	3.047887329
APOBEC3A	0.0462901	0.402054795	-4.629500897	-3.279283757	-5.23786383	-3.932361283	-3.687799537	-2.988504361	-2.164884385	-2.671163536
APOL2	0.000979346	0.283589744	2.150559677	1.992768431	2.64385619	2.895302621	4.371558863	3.817623258	4.62058641	4.209453366
APOLD1	0.0213655	0.260377358	-3.095419565	-2.816037165	-2.582079992	-3	-1.043943348	-2.388355457	-0.502259911	-0.434402824
AQP1	0.00152834	0.44566474	2.531069493	2.211012193	3.137503524	3.5360529	3.935459748	4.078951341	4.209453366	4.209453366
AQP10	0.00157125	2.779411765	3.058316496	3.267535798	2.625270489	2.608809243	1.298658316	1.659924558	1.560714954	1.214124805
AQP3	0.0267104	2.417142857	-0.910501849	-1.713118852	-1.126580497	-1.329159664	-3.625934282	-1.388355457	-2.816037165	-3.368849142
ARHGAP11A	0.0111875	0.070776256	-2.816037165	-2.680382066	-2.680382066	-2.573466862	0.970853654	-0.015957574	1.084064265	1.891419187
ARHGAP22	0.0181626	0.409703504	0.201633861	0.933572638	0.641546029	0.526068812	1.298658316	2.301587647	2.260025656	1.416839742
ARHGAP33	0.00432289	0.419354839	0.941106311	1.464668267	1.056583528	1.372952098	2.070389328	2.682573297	2.827819025	2.201633861
ARHGAP8	0.00901817	0.321088435	-4.061300187	-2.846843212	-3.853084152	-3.19759996	-2.158429363	-1.395928676	-1.407363571	-2.358453971
ARHGEF39	0.0386687	0.408839779	1.641546029	1.891419187	2.042644337	1.948600847	2.682573297	2.769771739	3.906890596	2.99095486
ARL4D	0.0359487	0.422330097	-1.407363571	-1.214240226	-1.251538767	-0.962969269	-0.727379545	0.678071905	0.137503524	-0.329159664
ARRB1	0.000315709	0.430842607	1.263034406	1.50589093	1.257010618	1.687060688	2.891419187	2.711494907	2.459431619	2.508428653
ARRDC4	0.0138973	2.320754717	-0.40354186	-0.336427665	-1.13289427	-1.12973393	-1.756330919	-1.634867407	-2.910501849	-1.698997744
ARSE	0.00501154	2.341666667	-1.63039393	-1.883635243	-2.395928676	-1.556393349	-2.756330919	-3.787866492	-3.023269779	-2.899695094
ASB10	0.0242234	0.183962264	-4.895394957	-5.117787378	-2.556393349	-2.662003536	-1.53951953	-1.648371671	-0.117161344	-0.902389203
ASB18	0.00931146	7	-3.095419565	-2.514573173	-3.279283757	-4.276485124	-5.921390165	-5.615287038	-6.276485124	-6.158429363
ASCC3	0.000304876	2.766631468	4.995484519	4.566815154	4.572889668	4.666756592	3.548436625	2.931683057	2.87774425	3.485426827
ASF1B	0.00370147	0.033140878	-4.216250017	-5.429731384	-5.53951953	-5.947862377	-0.459972731	-1.265344567	0.23878686	0.189033824
ASGR1	0.0200438	0.171323529	-2.795859283	-1.560642822	-1.873027144	-2.498178735	-0.380821784	0.286881148	1.263034406	0.097610797
ASIC1	0.000322821	0.398989899	1.23878686	1.35614381	1.084064265	1.292781749	2.639232163	2.718087584	2.684818738	2.17951105

ASIC4	0.0160616	0.340776699	-4.816037165	-4.345197874	-6.454822365	-4.49980982	-3.968040259	-2.977099598	-2.816037165	-3.662003536
ASL	0.0372255	0.485671192	1.700439718	1.82374936	1.59454855	1.618238656	2.17951105	2.933572638	3.286881148	2.195347598
ASNS	0.00548714	0.495049505	4.03562391	4.177917792	3.797012978	3.5360529	4.944858446	5.133399125	5.112700133	4.378511623
ASPG	0.00926639	0.341428571	-1.756330919	-0.682695932	0.298658316	-0.483984853	0.475084883	1.150559677	1.526068812	0.941106311
ASPM	0.0120816	0.019270833	-5.702749879	-5.304718805	-6.127841043	-6.030324537	-0.516635639	-1.422752464	0.250961574	0.650764559
ASTN1	0.0157137	3.037249284	-3.968040259	-3.083141235	-3.383830534	-2.785875195	-6.232429944	-6.179187923	-4.24331826	-4.061300187
ASTN2	7.54E-06	2.390946502	2.627606838	2.472487771	2.469885976	2.577730931	1.137503524	1.042644337	1.485426827	1.40599236
ATOH8	0.00234762	0.387878788	-1.502259911	-1.194294815	-0.800877358	-0.54793177	-0.083141235	0.344828497	0.584962501	0.659924558
ATP13A3	0.00508047	2.066502463	6.431288654	6.024585638	6.45779126	6.586464526	5.395748328	5.209453366	4.548436625	5.906890596
ATP13A4	0.000552335	4.852941176	-1.486004021	-1.63039393	-1.282789701	-2.139235797	-3.56149422	-4.058893689	-3.795859283	-4.173970214
ATP1A3	0.00168202	0.147606383	2.454175893	2.528571319	1.974529312	2.809414444	5.475733431	5.551516018	5.251719093	4.371558863
ATP6V1E2	0.005175	2.005277045	0.713695815	0.650764559	0.887525271	0.070389328	-0.187707155	-0.637109357	-0.545824107	-0.274040765
ATP8A1	0.00239334	2.457142857	3.906890596	3.807354922	3.62058641	3.378511623	2.64385619	2.759155834	0.895302621	2.61117238
ATP8A2	0.00047123	3.281690141	-0.420819852	-0.293358943	-0.77349147	-0.621488377	-2.634867407	-1.926865295	-3.265344567	-1.634867407
ATP8B3	0.0170818	0.159082569	-3.968040259	-5.853084152	-2.336427665	-3.884700356	-1.12973393	-1.351074441	-1.373327247	-0.078563669
AURKA	0.0105547	0.285185185	3.364572432	3.240314329	3.100977648	3.109360559	5.165911939	3.797012978	5.395748328	5.240314329
B3GNT7	0.00617149	2.205128205	-0.486004021	-0.588573754	-0.304006187	-0.828793173	-1.857259828	-0.918660373	-2.457989644	-1.932361283
BACH2	0.00453005	2.855172414	2.464668267	2.010779839	1.90303827	1.709290636	1.10433666	0.790772038	-1.286304185	0.575312331
BASP1-AS1	0.00400803	2.125	-5.66566056	-5.594225422	-5.658355759	-5.158429363	-6.615287038	-7.109794587	-6.767290131	-6.068543859
BAX	0.00107206	2.076109937	6.596935142	6.608809243	6.781359714	6.470862199	5.459431619	5.209453366	6.114783447	5.277984747
BBC3	8.10E-05	3.547120419	4.837943242	4.776103988	4.817623258	4.590961241	3.240314329	3.548436625	2.053111336	2.381283373
BEGAIN	0.0286229	2.651757188	-1.873027144	-1.694321257	-1.703689439	-3.224317298	-3.959037452	-2.573466862	-4.046921047	-3.585539694
BEST3	0.00725865	2.42962963	-0.554273297	-0.314732593	-0.682695932	-0.962969269	-2.040971781	-1.586405918	-3.76219157	-1.220950447
BEST4	0.0172838	2.280839895	-4.429731384	-3.343732465	-3.440655033	-3.184424571	-5.362899876	-4.345197874	-4.768076127	-4.570035956
BLK	0.0117822	2.96875	-2.64385619	-2.418889825	-3.23786383	-2.10780329	-3.891107598	-3.279283757	-4.729291666	-5.454822365
BLM	0.0124159	0.075564516	-5.461163892	-2.932361283	-2.258425153	-6.310432456	-0.710755715	-0.349235441	0.956056652	0.722466024
BMP2	0.000268733	2.231578947	-1.080087911	-1.177881725	-1.251538767	-1.469929258	-2.380821784	-2.899695094	-2.224317298	-2.184424571
BMPER	0.00346676	2.744505495	-3.74081792	-3.035046947	-3.426625474	-3.184424571	-6.221623189	-4.176576709	-4.938984225	-4.493296513
BORA	0.0112328	0.250413223	-1.810966176	-1.518701058	-1.805912948	-1.775959726	-0.087733372	-0.582079992	0.526068812	0.839959587
BRCA2	0.0314167	0.08577633	-7.63809692	-6.210896782	-7.661273243	-6.937215132	-4.037413962	-4.377819296	-3.660178495	-2.473931188
BRIP1	0.0176661	0.069337017	-2.481968507	-1.717856771	-1.64385619	-2.307572802	0.90303827	1.021479727	2.124328135	2.639232163
BTBD19	0.000637082	35.4368932	0.565597176	1.014355293	-0.061902439	0.475084883	-7.091971086	-3.952322025	-6.117787378	-3.673002535
BTK	0.0130276	4.009009009	-2.514573173	-1.921390165	-2.434402824	-3.481968507	-4.31329779	-6.058893689	-3.618827395	-5.016249352
BUB1	0.00588033	0.162849873	0.070389328	0.516015147	0.475084883	0.310340121	2.831877241	2.014355293	3.114367025	3.5360529
BUB1B	0.00523514	0.122627737	-2.805912948	-2.708396442	-2.251538767	-2.59946207	0.367371066	-0.450084446	0.422233001	1.070389328
C10orf55	0.00282856	3.27254306	-5.179187923	-4.636660688	-4.744680559	-5.374823043	-7.217323052	-5.861447625	-6.690777237	-7.40506933
C10orf95	0.040348	0.448979592	-5.904008087	-5.695255342	-4.122805453	-3.59946207	-3.685941591	-3.733123528	-3.210896782	-2.932361283
C11orf21	0.00720796	0.33937397	0.97819563	0.176322773	1.339137385	1.378511623	3.102658131	2.121015401	2.298658316	2.680324357
C11orf87	0.0385817	2.262135922	-5.673002535	-5.168771307	-6.158429363	-4.970299766	-7.043586436	-5.803896602	-7.786273234	-6.506352666
C16orf74	0.00720815	0.393194707	-2.358453971	-2.336427665	-2	-2.40354186	-1.10159814	-1.15521265	-0.386468347	-1.200912694
C16orf96	0.0345671	0.405405405	-3.023269779	-2.442222329	-2.411195433	-2.60823228	-1.388355457	-0.61705613	-2.070966521	-1.498178735
C17orf53	0.0109173	0.327142857	1.03562391	1.15704371	1.361768359	1.201633861	2.601696516	2.301587647	3.432959407	2.632268215
C17orf78	0.0460052	2.697674419	-2.418889825	-4.268121651	-3.035046947	-3.279283757	-5.270904092	-4.590744853	-4.625934282	-3.956795501

C18orf54	0.0231569	0.235294118	-2.652901329	-2.826232932	-3.346664773	-2.244685096	-1.415037499	-1.380821784	-0.329159664	0.042644337
C19orf57	0.00346635	0.148672566	-2.717856771	-1.790858602	-2.450084446	-4.354021725	0.163498732	-0.77349147	0.35614381	0.613531653
C1QL1	0.0136666	0.195538058	-3.40812913	-3.851000837	-3.531156057	-4.383830534	-1.411195433	-2.13289427	-0.666576266	-1.785875195
C1QTNF1	0.0114108	0.307471264	0.124328135	0.028569152	-0.19759996	0.389566812	1.678071905	1.427606173	2.440952198	1.384049807
C1R	0.0115709	0.264795918	-4.168771307	-3.546245393	-5.13796526	-4.710283552	-2.531156057	-2.434402824	-1.717856771	-3.023269779
C1S	0.00718572	0.404958678	0.575312331	1.5360529	0.526068812	1.014355293	1.992768431	2.748461233	2.150559677	2.070389328
C20orf144	0.00554362	2.08040201	-3.842697534	-3.594225422	-3.380821784	-3.594225422	-5.112786697	-3.930160375	-5.002310161	-4.886832943
C21orf58	0.00406516	0.182142857	0.150559677	0.150559677	0.056583528	-0.260151897	2.053111336	1.875780063	2.987320866	2.735522177
C21orf91	5.38E-05	3.626168224	1.981852653	1.967168608	2.084064265	1.778208576	-0.098505545	-0.298672743	-0.295128036	0.790772038
C2CD4C	0.0127892	0.275167785	-4.526161147	-4.439089439	-4.691522623	-4.811978949	-3.927962819	-2.224317298	-2.671163536	-2.652901329
C2orf91	0.016819	2.516746411	-4.787866492	-3.801883071	-4.386845572	-4.189680297	-6.693761096	-5.895394957	-5.733123528	-4.698997744
C3orf52	0.00401268	2.119205298	0.5360529	0.298658316	0.992768431	0.807354922	-0.698997744	-0.01887801	-0.680382066	-0.332789088
C5orf34	0.00306428	0.13960396	-2.61705613	-3.070966521	-3.210896782	-2.522840789	-0.883635243	-0.098505545	0.250961574	0.464668267
C8orf48	0.0251612	0.406666667	-4.676687582	-4.330610338	-4.284897364	-4.179187923	-3.527824196	-3.54962012	-2.418889825	-3.035046947
C9orf116	0.0484475	0.279896907	-1.046921047	-0.682695932	-0.625934282	-1.258425153	0.275007047	0.298658316	1.839959587	0.790772038
C9orf66	0.00375319	2.420408163	-4.556393349	-3.700872592	-4.216250017	-3.970299766	-5.362899876	-5.092955525	-5.66566056	-5.327710447
CABYR	0.0103405	2.421487603	1.500802053	1.98550043	1.500802053	1.077242999	-0.610433188	0.871843649	0.575312331	-0.226003675
CACNA1A	0.0219544	4.248120301	-4.779917739	-3.409661467	-4.848920527	-4.039784866	-6.189680297	-6.356975042	-7.011587974	-5.680382066
CACNA1S	0.0275212	0.269503546	-6.965784285	-5.756330919	-7.032211646	-6.467533417	-4.473931188	-3.886832943	-5.878321443	-4.654717182
CACNA2D2	0.0470058	0.427480916	2.641546029	2.3305584	2.195347598	2.709290636	4.137503524	4.217230716	2.61117238	3.364572432
CACNG6	7.55E-05	0.240628779	-2.395928676	-2.365871442	-2.083141235	-2.498178735	-0.612637459	-0.057391664	-0.200912694	-0.282789701
CAMK1D	0.00126943	2.989761092	3.485426827	3.084064265	3.107687869	2.746312766	1.970853654	1.682573297	0.918386234	1.438292852
CAMK1G	0.00142775	4.268585132	-2.61705613	-2.321928095	-2.164884385	-2.988504361	-4.636660688	-6.454822365	-3.789860543	-4.53616832
CARHSP1	0.0167394	0.370748299	3.27351589	3.350497247	3.560714954	3.596935142	4.602884409	4.350497247	5.51412226	4.776103988
CATIP	0.00325437	3.127272727	-2.314732593	-2.910501849	-2.244685096	-2.826232932	-4.680382066	-4.844768884	-3.362899876	-4.351074441
CATSPERG	0.00137761	3.117932149	1.021479727	1.269033146	0.799087306	0.632268215	-1.325539348	0.042644337	-1.142417045	-0.74178261
CAV3	0.0208169	0.132905983	-4.820106829	-6.41734766	-4.208227596	-5.423526235	-2.314732593	-3.13289427	-1.261880711	-2.300448367
CAVIN2	0.00384796	2.098265896	-1.450084446	-1.231074664	-1.648371671	-1.54793177	-2.40354186	-3.526161147	-2.625934282	-1.977099598
CBLN1	0.0189385	2.524084778	-2.564904848	-2.680382066	-3.158429363	-3.53449563	-4.470728756	-3.3703403	-5.321928095	-4.622376462
CBLN3	0.0446316	0.48655914	-3.748553568	-3.083141235	-3.332062472	-3.832385159	-3.224317298	-2.582079992	-1.831357964	-2.40354186
CCBE1	0.000836021	2.941605839	0.014355293	-0.579921884	-0.541617996	-0.217591435	-1.577766999	-1.857259828	-2.442222329	-1.722610301
CCDC141	0.0138316	2.259541985	5.177917792	4.689299161	4.727920455	4.906890596	4.263034406	3.765534746	0.214124805	4.209453366
CCDC148	0.0148712	6.256684492	0.35614381	0.941106311	-0.078563669	-0.867752202	-2.708396442	-2.358453971	-2.017417053	-2.708396442
CCDC150	0.0109607	0.376884422	-1.10159814	0.176322773	-0.095419565	-1.114035243	0.485426827	0.678071905	1.195347598	1.416839742
CCDC3	0.000308017	6.79245283	1.40599236	1.565597176	1.469885976	0.773996325	-1.74178261	-2.336427665	-0.857259828	-1.204233052
CCDC69	0.0163357	0.417105263	2.601696516	1.981852653	2.833902077	3.03562391	4.378511623	3.10433666	3.906890596	4.026800059
CCDC74A	0.00886493	0.323529412	0.731183242	1.257010618	0.98550043	0.545968369	1.500802053	2.715893371	2.87774425	2.669026766
CCDC80	0.000882343	2.838345865	0.422233001	0.97819563	0.516015147	0.389566812	-1.020340448	-1.011587974	-1.046921047	-0.60823228
CCDC88B	0.0162364	0.498194946	-1.477944251	-1.177881725	-1.120294234	-1.343732465	-0.40927823	0.250961574	-0.390245038	-0.694321257
CCDC96	0.00165793	2.18699187	-1.785875195	-1.708396442	-1.852042119	-2.286304185	-3.120294234	-2.573466862	-3.224317298	-3.321928095
CCL21	0.00938776	2.461538462	-1.265344567	-1	-1.145605322	-0.854648614	-3.947862377	-3.400491764	-1.473931188	-1.943416472
CCL3L3	0.00242218	8.19112628	-1.905088353	-1.639354798	-2.058893689	-2.943416472	-6.532824877	-5.756330919	-4.002310161	-5.299027693
CCNA1	0.048599	0.340378198	-1.347398782	-0.886299501	-2.251538767	-3.66566056	-1.586405918	-0.061902439	0.475084883	-0.139235797

CCNA2	0.0130085	0.06	-3.346664773	-2.785875195	-3.13289427	-3.51622291	0.555816155	-0.691988685	1.344828497	1.469885976
CCNB1	0.00754784	0.053703704	-0.905088353	-0.758769964	-0.708396442	-0.783389931	3.198494154	2.22650853	4.078951341	3.62058641
CCNB2	0.00840825	0.014166667	-5.122805453	-4.691522623	-4.799872346	-5.276485124	0.871843649	-0.144010303	1.843983844	1.480265122
CCNE2	0.0335467	0.225233645	-2.095419565	-1.994240731	-1.77102743	-2.426625474	-0.430508908	-0.991369695	0.214124805	0.910732662
CCNF	0.000284985	0.232954545	-1.994240731	-2.152003093	-2.573466862	-2.498178735	0.042644337	-0.671163536	-0.112474729	-0.09696173
CCNT2-AS1	8.31E-05	2.716723549	-0.253257284	-0.360304767	-0.36215794	-0.340075442	-1.708396442	-1.377069649	-2.775959726	-1.582079992
CD109	0.00441942	2.492682927	-1.008682243	-1.321928095	-0.744197163	-0.865121946	-2.152003093	-2.795859283	-3.307572802	-1.518701058
CD1D	0.0384653	0.292372881	-2.046921047	-2.388355457	-1.477944251	-1.685013515	-0.826232932	-0.147202107	0.731183242	-0.632628934
CD248	0.0257355	0.326415094	-4.717856771	-5.148161027	-5.519528055	-4.316168826	-4.080698059	-2.708396442	-2.846843212	-3.775959726
CD274	0.0311977	2.039215686	-1.790858602	-2.204233052	-1.190997225	-1.727379545	-2.307572802	-3.171368418	-3.615287038	-2.210896782
CDC20	0.00290144	0.023851852	-3.997693533	-4.127841043	-3.914847319	-3.807932116	1.40053793	0.704871964	2.073820233	1.214124805
CDC25A	0.0010372	0.229155313	-0.426625474	-0.498178735	-0.219269964	0.070389328	1.646162657	1.560714954	1.831877241	2.3305584
CDC25B	0.00653231	0.309248555	2.313245852	2.550900665	2.498250868	2.298658316	3.906890596	3.666756592	4.689299161	3.972692654
CDC25C	0.0151394	0.015721649	-2.442222329	-3.120294234	-5.24331826	-4.636660688	2.017921908	1.339137385	3.364572432	2.669026766
CDC42BPG	0.00229529	2.3125	1.545968369	1.526068812	1.589763487	1.599317794	0.565597176	0.963474124	-1.399730246	0.378511623
CDC45	0.0162536	0.0235	-4.161007907	-2.826232932	-1.510457064	-5.947862377	1.659924558	1.443606651	3.142413438	3.212569339
CDC6	0.0131685	0.032012987	-3.385337265	-2.846843212	-4.145605322	-3.279283757	1.056583528	0.378511623	1.944858446	2.350497247
CDC7	0.0275165	0.256302521	0.22650853	0.464668267	0.454175893	-0.045431429	1.827819025	0.963474124	2.655351829	2.851998837
CDCA2	0.0128119	0.048220065	-2.899695094	-2.481968507	-2.231074664	-3.867752202	1.097610797	0.286881148	2.229587923	2.111031312
CDCA3	0.00346152	0.148110317	0.286881148	1.049630768	0.411426246	0.22650853	3.03562391	2.523561956	3.797012978	3.498250868
CDCA5	0.00795117	0.060119048	-2.531156057	-2.662003536	-1.954557029	-2.190997225	2.049630768	1.007195501	1.014355293	2.3950628
CDCA7	0.0432885	0.302439024	-1.53951953	-1.120294234	-1.415037499	-1.698997744	-0.224317298	-0.650634722	0.35614381	1.111031312
CDCA8	0.0142994	0.017210884	-5.232429944	-4.921390165	-6.030324537	-5.24331826	0.90303827	-0.537424112	-0.244685096	1.298658316
CDH10	0.0151303	2.080536913	1.09085343	1.263034406	0.659924558	0.422233001	0.275007047	-0.780908942	-0.54793177	0.137503524
CDH11	0.000696374	2.016949153	3.62058641	3.765534746	3.378511623	3.485426827	2.799087306	2.778208576	2.114367025	2.443606651
CDH18	0.0358493	17.63942931	-2.857259828	-2.336427665	-2.286304185	-9.449769138	-9.380821784	-8.196012545	-8.854752972	-5.392894616
CDH6	0.013796	2.837837838	-2.058893689	-2.61705613	-2.095419565	-3.046921047	-3.72356289	-5.194955239	-4.594225422	-3.035046947
CDH8	7.94E-06	5.912921348	2.269033146	1.952333566	1.959770155	2.09085343	-0.277533976	-0.929610672	-0.932361283	-0.040971781
CDK1	0.0252482	0.018030303	-3.506352666	-3.582079992	-0.66428809	-2.746615764	3.008988783	1.843983844	4.432959407	4.285402219
CDK18	0.00144999	0.330232558	1.782408565	1.974529312	2.316145742	2.22650853	3.722466024	3.87774425	3.906890596	3.084064265
CDK5R2	0.00771837	3.31002331	-5.184424571	-4.587272661	-5.493296513	-5.480357457	-6.902281342	-7.039784866	-6.117787378	-7.998615677
CDKN1A	0.000288738	4.981273408	10.65284497	10.57931594	10.11634396	10.0768156	8.28077077	8.21916852	8.022367813	7.62935662
CDON	0.017294	0.439882698	0.432959407	0.35614381	0.704871964	0.815575429	2.070389328	1.070389328	1.49057013	2.182692298
CDT1	0.0251219	0.051462995	-4.691522623	-5.158429363	-5.020925839	-5.49980982	-1.586405918	-1.61705613	0.163498732	-0.867752202
CELF6	0.0449704	2.189333333	-1.01449957	0.23878686	-0.030619235	-0.659722595	-2.652901329	-1.194294815	-0.865121946	-1.486004021
CEMIP	0.0156874	2.173745174	-0.575615328	-0.465938398	-1.01449957	-1.461958547	-1.486004021	-2.457989644	-2.224317298	-1.821126042
CENPE	0.0170434	0.021511628	-4.383830534	-3.884700356	-4.848920527	-3.803896602	0.526068812	0.739848103	1.263034406	2.269033146
CENPF	0.00655505	0.019605911	-3.760235373	-3.704629612	-3.727379545	-3.437525542	1.803227036	1.207892852	1.835924074	2.778208576
CENPH	0.0279149	0.09005848	-2.836501268	-3.120294234	-2.910501849	-2.139235797	0.150559677	-0.816037165	1.124328135	1.570462931
CENPI	0.000929487	0.135119048	-2.046921047	-2.017417053	-2.265344567	-2.231074664	0.765534746	0.176322773	0.659924558	1.201633861
CENPK	0.0381633	0.063580247	-1.234465254	0.464668267	0.189033824	0.22650853	2.843983844	2.448900951	4.765534746	4.632268215
CENPL	0.0487357	0.469105691	-0.388355457	-1.086201035	-0.768567592	-1.043943348	0.516015147	-1.043943348	0.432959407	0.713695815
CENPN	0.00937322	0.401041667	2.12763328	1.827819025	1.937344392	1.867896464	3.100977648	2.622930351	3.744161096	3.364572432

CENPU	0.0447439	0.142388759	-0.954557029	-0.639354798	-0.477944251	-0.846843212	1.15704371	0.739848103	2.981852653	2.397802962
CENPVL1	0.0248362	2.004008016	-2.932361283	-3.437525542	-3.345197874	-3.647467443	-4.333516069	-3.69712533	-6.058893689	-4.10780329
CENPVL3	0.0034665	2.251131222	-2.307572802	-2.395928676	-2.23786383	-2.380821784	-4.28771238	-2.76611194	-4.284897364	-3.265344567
CEP128	0.00736035	0.171775701	-3.330610338	-3.590744853	-4.070966521	-2.988504361	-0.554273297	-1.304006187	-1.76611194	-0.394031641
CEP72	0.00301296	0.439568345	-1.02620507	-0.597277823	-0.3382504	-0.994240731	0.613531653	0.748461233	0.014355293	0.411426246
CERS1	0.000707283	0.417757009	-0.236163541	0.111031312	-0.106249498	-0.488026018	1.117695043	1.03562391	1.372952098	0.790772038
CFC1	0.00601618	0.335454545	-5.993091631	-4.651087759	-3.712173133	-6.002310161	-3.058893689	-3.364384894	-2.888968688	-3.480357457
CFD	0.0332109	0.327862595	-4.31329779	-3.674843882	-3.120294234	-3.326262685	-2.457989644	-2.888968688	-1.612637459	-1.304006187
CFL1P1	0.000447206	3.233404711	-2.943416472	-2.785875195	-2.411195433	-2.836501268	-4.339345148	-5.339345148	-4.066125258	-4.235144329
CHAC1	0.0382237	0.418181818	0.070389328	0.214124805	-0.330973234	-0.214240226	1.773996325	1.077242999	1.422233001	0.056583528
CHAF1A	0.00153658	0.149633252	-0.545824107	-0.637109357	-0.886299501	-0.783389931	1.580145484	1.584962501	2.397802962	2.347665656
CHAF1B	0.00150933	0.10625	-2.717856771	-3	-3.293358943	-3.361416385	-0.209227962	-0.195946441	0.124328135	0.704871964
CHEK2	0.0138613	0.322123894	1.389566812	2.253989266	1.887525271	1.794935663	3.485426827	2.989139007	3.102658131	4.129283017
CHI3L1	0.0361719	2.152073733	-4.748553568	-4.764150423	-3.936773198	-4.392894616	-7.786273234	-5.143054137	-5.673002535	-4.861447625
CHODL	0.0242198	4.933333333	0.014355293	0.389566812	-0.77349147	-1.506352666	-3.251538767	-2.921390165	-1.98279071	-2.54793177
CHRNA1	0.0147597	2.614980289	-2.40354186	-1.988504361	-2.089267338	-3.083141235	-3.475535074	-3.493296513	-3.19759996	-5.930160375
CHST13	0.00684819	0.22244898	-5.454822365	-4.398969131	-4.993091631	-5.092955525	-3.023269779	-2.336427665	-2.418889825	-3.64385619
CHTF18	0.0315065	0.321131448	0.070389328	1.50589093	1.063502942	0.807354922	1.570462931	2.427606173	3.289834465	2.560714954
CIP2A	0.030312	0.366257669	-0.883635243	-0.634867407	-0.637109357	-0.844250767	0.422233001	0.659924558	-0.080087911	1.40599236
CIT	0.00183955	0.017191977	-5.448508591	-3.425075022	-3.307572802	-5.454822365	1.996388746	0.948600847	1.627606838	2.313245852
CKAP2L	0.0151519	0.017857143	-5.194955239	-4.454822365	-6.480357457	-4.799872346	0.575312331	-1.083141235	0.948600847	1.50589093
CKB	0.00808048	0.48245614	5.197708158	5.318316841	4.791814071	4.791814071	6.121015401	6.392317423	6.238404739	5.469234794
CKLF	0.0471195	0.306397306	0.641546029	0.298658316	1.232660757	1.10433666	2	1.545968369	3.364572432	2.720278465
CKLF-CMTM1	0.0479508	0.291093117	-0.974262439	-0.921390165	0.070389328	-0.343732465	0.748461233	0.23878686	2.111031312	1.427606173
CKM	0.00973552	0.25245098	5.8899602	5.133399125	5.64385619	5.94016675	7.665335917	7.64385619	8.262094845	6.754887502
CKS1B	0.0108913	0.338345865	2.114367025	1.895302621	2.358958826	2.269033146	3.689299161	2.765534746	4.169925001	3.935459748
CLCNKB	0.00670183	0.118666667	-1.905088353	-0.971430848	-1.816037165	-1.461958547	1.15704371	1.09085343	2.295723025	1.459431619
CLDN1	0.027138	3.900943396	-0.035046947	0.422233001	-0.810966176	-1.254977851	-2.023269779	-1.708396442	-2.899695094	-2.64385619
CLDN6	0.00698267	0.169465649	1.097610797	0.333423734	1.292781749	1.608809243	3.432959407	3.596935142	4.385431037	3.121015401
CLEC2A	0.000133292	3.271186441	1.944858446	1.735522177	2.150559677	1.941106311	-0.307572802	0.831877241	-0.002888279	0.189033824
CLIP4	0.00212886	2.065989848	5.416164165	5.004501392	5.590961241	5.311067102	4.263034406	4.217230716	3.906890596	4.700439718
CLSPN	0.0421476	0.340721649	-0.595096878	-0.388355457	-0.646112164	-0.790858602	0.516015147	0.584962501	0.495695163	1.778208576
CMKLR1	2.96E-05	5.659722222	-1.377069649	-1.61705613	-1.634867407	-1.878321443	-4.130365444	-4.622376462	-3.963531833	-3.863546091
CMTM1	0.0117023	0.358126722	-1.40354186	-1.932361283	-2.314732593	-2.329159664	-0.03948829	-1.104697379	-0.813499442	-0.158429363
CNR1	0.039024	8.305084746	-4.345197874	-2.293358943	-1.971430848	-3.645660687	-6.088040035	-6.127841043	-5.356975042	-5.861447625
CNTN3	0.0271328	0.381875	-4.01391678	-3.597714408	-4.570035956	-4.10780329	-3.321928095	-3.171368418	-2.395928676	-2.070966521
COCH	0.0121739	0.268518519	-3.429731384	-2.395928676	-2.634867407	-2.878321443	-1.703689439	-1.200912694	-0.2968993	-0.727379545
COL10A1	0.00801387	3.481276006	-2.590744853	-1.465938398	-1.883635243	-2.279283757	-3.910501849	-3.158429363	-5.078259014	-3.642053947
COL11A2	0.0144671	0.41754386	5.840463234	5.070389328	5.495055528	5.768184325	7.033423002	7.312882955	6.266786541	6.48703608
COL24A1	0.0282566	3.314606742	-2.531156057	-1.921390165	-2.272297327	-3.945637712	-3.76219157	-4.073393259	-5.316168826	-4.176576709
COL9A3	0.00686964	0.418867925	-4.28208783	-3.23786383	-4.710283552	-3.820106829	-2.795859283	-3.023269779	-2.61705613	-2.279283757
COLEC10	0.0031624	3.463035019	-3.710283552	-3.023269779	-3.736965594	-3.624154275	-5.993091631	-5.40506933	-4.470728756	-5.772012541
COTL1	0.0171147	2.721925134	-0.81857936	-0.436353731	-1.13289427	-1.862496476	-2.145605322	-2.395928676	-2.727379545	-2.450084446

CPA4	0.00191989	2.551912568	-1.055891201	-0.849440323	-1.321928095	-1.204233052	-2.336427665	-1.775959726	-3.470728756	-2.717856771
CPE	3.55E-05	2.515991471	3.632268215	3.689299161	3.498250868	3.419538892	2.169925001	1.819668183	2.456806149	2.384049807
CPLX1	0.00159294	0.158054711	-3.330610338	-4.090495686	-3.374823043	-2.61705613	-0.634867407	-0.371459681	-0.268816758	-1.380821784
CPM	0.027473	2.118055556	0.189033824	0.722466024	0.070389328	0.056583528	-1.080087911	0.070389328	-1.948975997	-0.932361283
CPXM1	0.012303	0.180373832	-2.76611194	-2.184424571	-2.418889825	-2.184424571	-0.164884385	-0.325539348	0.855989697	-0.358453971
CPZ	0.00387012	0.359638554	-0.886299501	-0.298672743	-0.979942348	-0.921390165	0.82374936	1.124328135	0.687060688	0.137503524
CR2	0.000985594	8.4	-2.910501849	-2.556393349	-3.035046947	-3.640253953	-6.333516069	-5.820106829	-5.803896602	-6.368849142
CRB2	0.00254527	0.499001996	-2.210896782	-1.477944251	-2.293358943	-2.171368418	-0.800877358	-1.289827252	-0.924125133	-1.01449957
CRIM1	0.00119511	2.092672414	3.419538892	3.028569152	3.249445341	3.378511623	2.451540833	1.655351829	1.981852653	2.587364991
CRTAC1	0.00470478	10.16427105	2.521050737	2.867896464	2.056583528	1.367371066	-0.724992953	-0.543719518	-1.279283757	-2.023269779
CRYBG1	0.00713892	2.179039301	-4.597714408	-4.24879339	-4.470728756	-4.044538396	-5.506352666	-6.532824877	-5.480357457	-4.779917739
CRYM	0.024613	0.406355932	-4.148161027	-2.231074664	-4.336427665	-3.965784285	-1.960159735	-1.977099598	-1.873027144	-2.64385619
CSDC2	0.0320618	0.260909091	-1.943416472	-2.089267338	-1.60823228	-1.61705613	-0.481968507	0.895302621	0.321928095	-0.798366139
CSGALNACT1	0.000314278	4.01523395	1.851998837	2.23572706	1.718087584	1.655351829	-0.430508908	0.250961574	-0.582079992	0.111031312
CSPG5	3.61E-06	0.245753425	-0.367731785	-0.077041036	-0.343732465	0.111031312	1.778208576	1.769771739	1.887525271	2.025028794
CT55	0.0103984	0.45754717	-7.189680297	-5.374823043	-4.988504361	-6.020925839	-4.262572817	-4.673002535	-4.618827395	-4.729291666
CTB-3M24.3	0.000706657	3.217391304	-3.828280761	-3.682232861	-3.995390747	-4.176576709	-4.952322025	-6.020925839	-6.823370847	-5.23786383
CTD-2201I18.1	0.020234	4.614173228	-4.168771307	-3.326262685	-4.477140745	-4.869859865	-6.519528055	-7.142034924	-5.629500897	-6.299027693
CTDSPL	0.000322153	0.332659933	0.137503524	-0.192645078	0.163498732	-0.234465254	1.613531653	1.176322773	1.811471031	1.613531653
CXCL12	0.000656866	2.392857143	5.815063017	5.375039431	5.925999419	5.797012978	4.781359714	4	4.452858965	4.596935142
CXXC4-AS1	0.00268417	2.974358974	-6.506352666	-6.058893689	-6.722419859	-6.519528055	-8.454822365	-9.269790472	-7.345197874	-7.64385619
CXXC5	0.0132775	0.493449782	5.129283017	4.963474124	5.078951341	5.161887682	5.951867504	6.54689446	6.199672345	5.526694846
CYBRD1	0.00221545	0.478764479	-1.454031631	-1.775959726	-1.329159664	-1.210896782	-0.488026018	-0.241270432	-0.720231578	-0.086201035
CYP2C8	0.00930574	0.434322034	-3	-2.217591435	-1.577766999	-2.785875195	-1.573466862	-1.03209363	-0.758769964	-1.089267338
CYP39A1	0.0374538	0.447609359	-1.351074441	-1.612637459	-1.380821784	-0.610433188	-1.061902439	0.443606651	0.22650853	-0.117161344
CYP8B1	0.000439948	2.62295082	-4.874084451	-4.721658341	-5.13289427	-5.179187923	-6.058893689	-6.097887821	-6.757891433	-6.64385619
DAND5	0.00150313	0.128951049	-3.684086035	-3.988504361	-3.622376462	-2.76611194	-0.590744853	-0.971430848	0.042644337	-0.612637459
DBF4	0.0100038	0.384375	-0.808437349	-0.411195433	-0.293358943	-0.30222618	0.713695815	0.263034406	1.389566812	1.163498732
DCDC1	0.0441161	3.492569002	-0.880975897	-1.590744853	-1.564904848	-3.293358943	-3.293358943	-3.529489165	-4.473931188	-2.805912948
DCLK1	0.0390628	3.327526132	-3.842697534	-2.582079992	-4.192315357	-3.462753639	-4.943416472	-5.869859865	-5.259806383	-4.673002535
DCP1B	0.00183266	2.769461078	1.014355293	1.070389328	0.748461233	0.659924558	-0.512513651	0.214124805	-1.775959726	-0.937878288
DDB2	1.60E-05	3.149882904	4.683696454	4.882643049	4.837943242	4.566815154	3.204766751	2.752748591	3.201633861	3.173127433
DDIAS	0.00758779	0.099574468	-2.717856771	-3.095419565	-4.354021725	-4.153286059	-0.564904848	-0.965784285	0.5360529	0.176322773
DDR2	0.039328	0.480885312	-2.139235797	-2.089267338	-2.171368418	-1.867752202	-0.805912948	-0.899695094	-2.210896782	-0.590744853
DDX11	0.000514327	0.369565217	3.200064862	3.40599236	3.301587647	3.510961919	4.44625623	4.649615459	5.017921908	4.968090752
DDX11-AS1	0.0192894	0.346153846	-5.153286059	-4.327710447	-5.756330919	-6.973015854	-4.04930764	-4.402016006	-3.337885669	-3.395928676
DDX12P	0.00341653	0.400605449	-1.836501268	-1.123433941	-1.13289427	-1.343732465	-0.659722595	0.111031312	0.189033824	0.150559677
DENND2D	0.0351646	4.221954162	-1.639354798	-1.10780329	-0.940644722	-3.391379976	-4.49980982	-4.100360306	-2.60823228	-3.943416472
DEPDC1	0.0294104	0.043564815	-3.40506933	-3.645660687	-3.19759996	-3.421979109	0.575312331	-1.164884385	1.839959587	1.646162657
DEPDC1B	0.00593506	0.055295316	-4.304718805	-5.020925839	-3.642053947	-4.168771307	-0.341902795	-1.148800661	0.333423734	0.516015147
DERL3	0.00648463	0.209119497	-1.351074441	-0.15521265	-0.325539348	-0.805912948	0.757023247	1.761285273	2.211012193	1.59454855
DES	8.55E-05	0.052796421	0.90303827	0.22650853	1.344828497	1.956056652	5.329123596	5.563768278	5.804776378	5.137503524
DGCR6	0.00850136	2.334782609	5.683696454	5.675251386	6.002252452	5.587964989	4.153805336	3.827819025	5.469234794	3.981852653

DGKG	0.00962824	6.411764706	-3.861447625	-3.355497628	-3.869859865	-5.227016448	-7.825005629	-6.64385619	-6.299027693	-6.179187923
DGKI	0.00396385	3.152302243	5.201633861	4.705977902	4.529820947	4.371558863	3.632268215	2.618238656	2.169925001	3.44625623
DHRS12	0.0496225	0.49137931	0.111031312	-0.181149439	0.411426246	0.333423734	0.773996325	1.097610797	1.883620816	0.782408565
DHRS13	0.0044447	0.212280702	-1.300448367	-1.514573173	-1.671163536	-1.399730246	0.722466024	0.263034406	1.367371066	0.495695163
DHRS9	0.00286249	2.783088235	-0.17299399	-0.057391664	-0.810966176	-0.715485867	-1.685013515	-2.158429363	-1.732164608	-1.994240731
DINOL	0.000317615	3.722397476	0.443606651	-0.109358756	0.411426246	0.137503524	-2.64385619	-1.477944251	-1.220950447	-1.634867407
DISP2	0.0207007	2.747474747	-2.826232932	-2.272297327	-3.398969131	-3.333516069	-4.432843996	-3.838563734	-5.276485124	-4.163591068
DIXDC1	0.00123457	2.116402116	4.232660757	3.765534746	4.017921908	3.944858446	3.119356177	2.869871406	2.277984747	3.231125158
DKFZP434A062	0.0357979	0.167460317	-8.265344567	-9.278723596	-7.480357457	-7.278723596	-6.519528055	-4.706511798	-4.687799537	-6.28771238
DLEU1	0.0266119	0.483243243	-0.098505545	-0.298672743	-0.045431429	-0.219269964	1.207892852	-0.160040413	1.150559677	0.992768431
DLGAP1	0.034061	2.284710018	-2.590744853	-2.652901329	-3.070966521	-3.717856771	-4.102837037	-3.869859865	-6.601211852	-3.462753639
DLGAP5	0.00254341	0.004957507	-6.221623189	-6.973015854	-4.882570916	-6.097887821	1.769771739	0.713695815	2.028569152	2.319039816
DNAJA4	0.00367875	3.378378378	3.257010618	3.214124805	2.560714954	2.3950628	1.321928095	1.475084883	0.669026766	1.007195501
DOCK2	0.0368891	3.59832636	-5.53951953	-6.221623189	-5.189680297	-7.241134013	-7.328869704	-7.368849142	-8.973015854	-7.687799537
DOCK3	0.00149064	4.903474903	0.765534746	1.682573297	1.613531653	1.117695043	-1.13289427	-0.943416472	-1.351074441	-0.506352666
DOCK8	0.00138889	7.446351931	-0.610433188	0	-0.841662973	-0.836501268	-2.943416472	-3.10780329	-4.506352666	-3.585539694
DOK7	0.0028392	0.257264957	1.327687364	1.316145742	1.673556424	1.944858446	3.523561956	4.008988783	3.5360529	2.912649865
DPP10	0.0174148	3.289473684	-2.244685096	-1.411195433	-1.59946207	-2.457989644	-2.60823228	-3.011587974	-5.011587974	-6.030324537
DPYSL2	1.37E-05	0.254008439	-0.606034724	-0.512513651	-1.13606155	-0.746615764	1.443606651	1.244887059	1.15704371	1.10433666
DPYSL5	0.00472371	0.405511811	-2.343732465	-1.60823228	-2.708396442	-2.785875195	-0.603840511	-1.272297327	-1.181149439	-0.943416472
DRAM1	0.000298831	4.854166667	0.042644337	0.028569152	-0.295128036	-0.227692025	-3.23786383	-1.200912694	-2.899695094	-3.434402824
DSCC1	0.0297241	0.039622642	-5.270904092	-4.921390165	-5.869859865	-5.921390165	-1.49410907	-2.795859283	-0.115597447	-0.134477041
DSN1	0.0189401	0.17659805	0.545968369	0.847996907	0.659924558	0.748461233	2.669026766	2.017921908	3.786596362	3.666756592
DTL	0.016377	0.022811245	-4.402016006	-3.359934417	-5.189680297	-4.181803871	0.389566812	0.163498732	1.86393845	1.952333566
DTYMK	0.0337729	0.412903226	3.051372102	3.336283388	2.767654798	2.464668267	4.053111336	3.584962501	4.892391026	3.99095486
DUSP2	0.000309113	2.110320285	-0.831357964	-0.736965594	-0.732164608	-0.720231578	-2.490050854	-1.407363571	-1.816037165	-1.800877358
DUSP4	0.00971846	13.41496599	0.321928095	0.59454855	-0.380821784	-1.254977851	-3.506352666	-4.161007907	-2.965784285	-5.480357457
DUSP6	0.00208311	6.11969112	1.963474124	1.98550043	1.589763487	0.86393845	-1.54793177	-0.81857936	-0.288064643	-1.535331733
DUX4	0.0322854	3.222748815	-2.450084446	-1.775959726	-2.040971781	-3.337885669	-3.42043364	-2.943416472	-8.133907043	-5.265344567
DWORF	0.0281202	0.376582278	-5.020925839	-6.615287038	-5.40506933	-5.035046947	-4.556393349	-4.371833001	-3.329159664	-4.006941609
DYNLT3	0.000732318	2.385093168	1.803227036	1.937344392	2.201633861	1.773996325	0.056583528	0.575312331	1.021479727	0.918386234
E2F1	0.00974654	0.392746114	-0.595096878	-0.029146346	-0.454031631	-0.595096878	0.495695163	0.847996907	0.757023247	1.485426827
E2F2	0.00489175	0.073676471	-4.706511798	-3.954557029	-4.316168826	-4.395928676	-1.282789701	-1.161653263	-0.134477041	-0.075520008
ECT2	0.0175235	0.255801105	2.13422094	2.17951105	2.324810603	2.198494154	3.95419631	2.904965719	4.578938713	4.66106548
ECT2L	0.0249925	2.39047619	-1.836501268	-1.556393349	-1.926865295	-3.046921047	-3.597714408	-3.756330919	-3.120294234	-2.76611194
EDA	0.0052844	0.327455919	0.321928095	-0.364013496	0.286881148	0.956056652	2.280956314	1.773996325	1.378511623	2.321928095
EDA2R	4.75E-07	4.046997389	7.209453366	7.357552005	7.330916878	7.209453366	5.318316841	5.357552005	4.897240426	5.412781525
EDN2	0.000946111	2.780269058	-0.048412205	0.575312331	0.443606651	0.189033824	-1.810966176	-0.77349147	-1.089267338	-1.168122759
EEPD1	0.0411845	2.203438395	-0.093879047	-0.020340448	-0.662003536	-0.943416472	-1.251538767	-0.666576266	-3.451662024	-1.977099598
EFEMP2	0.00985796	0.433484163	3.049630768	3.135863165	3.432959407	3.392317423	4.350497247	4.426264755	4.963474124	3.944858446
EFHC2	0.0287235	0.49122807	-2.064917477	-2.244685096	-0.937878288	-1.422752464	-0.724992953	-1.171368418	-0.288064643	-0.200912694
EFNA2	0.0348699	0.376623377	-2.265344567	-2.336427665	-2.54793177	-3.083141235	-0.965784285	-0.45600928	-1.450084446	-2.040971781
EGFR	0.00236612	10.53631285	0.286881148	0.310340121	-0.477944251	-0.751465164	-3.81400663	-2.64385619	-4.262572817	-3.742747947

EGR1	0.00105578	4.20624152	2.028569152	1.250961574	1.66448284	1.464668267	-0.112474729	0.084064265	-1.518701058	-0.708396442
EGR2	0.000529085	5.341614907	1.500802053	1.056583528	1.632268215	1.195347598	-1.325539348	0.111031312	-2.457989644	-1.883635243
EMP2	0.011659	0.372781065	3.204766751	3.099295204	3.817623258	4.201633861	5.057450272	4.426264755	5.587964989	5.017921908
ENC1	0.0433193	2.669642857	1.918386234	2.114367025	1.321928095	0.422233001	-0.418889825	0.963474124	-0.687334826	0.201633861
ENDOD1	0.00390281	2.824675325	0.042644337	0.124328135	-0.516635639	-0.595096878	-1.926865295	-1.089267338	-2.231074664	-1.795859283
ENDOG	0.0249571	0.48427673	2.869871406	2.967168608	2.887525271	3.049630768	3.765534746	3.733354341	4.584962501	3.632268215
ENKUR	0.0111158	2.323529412	-4.13289427	-4.377819296	-4.66566056	-4.46434514	-5.168771307	-7.493296513	-6.158429363	-4.869859865
ENO1-AS1	0.00307867	2.058988764	-0.687334826	-0.659722595	-0.289827252	-0.219269964	-2.265344567	-1.329159664	-1.293358943	-1.289827252
ENO3	0.0281046	0.490909091	8.531381461	8.622051819	8.581200582	8.50779464	9.319672121	9.54303182	10.17990909	9.071462363
EP300-AS1	0.0213179	0.341666667	-5.102837037	-4.857259828	-4.058893689	-4.633076351	-2.680382066	-4.383830534	-2.76611194	-2.943416472
EPB41L4B	0.00304353	2.610795455	-0.641603738	0.201633861	-0.089267338	-0.081613766	-1.852042119	-0.991369695	-1.954557029	-1.438307279
EPHA2	0.00430095	3.008962868	4.791814071	4.672425342	4.329123596	4.364572432	3.065227623	3.972692654	0.613531653	2.49057013
EPHA3	0.00315088	2.925170068	0.773996325	0.176322773	-0.023269779	0.422233001	-0.808437349	-1.756330919	-1.780908942	-0.722610301
EPHA4	0.000280555	4.008321775	5.09592442	4.770829046	4.655351829	4.857980995	3.548436625	2.017921908	1.618238656	3.320484678
EPHB2	0.00185036	2.339130435	1.669026766	1.5360529	1.201633861	1.257010618	0.526068812	0.555816155	-0.722610301	0.124328135
EPHX4	0.0044241	0.446621622	-4.073393259	-3.779917739	-3.943416472	-3.895394957	-2.373327247	-2.64385619	-3.13289427	-3.023269779
EPPIN	0.0109281	2.553488372	-4.027969116	-4.426625474	-4.414268267	-3.950090478	-6.078259014	-4.698997744	-8.356975042	-5.073393259
EPS8L1	0.0457253	0.493230174	-3.19759996	-1.590744853	-1.564904848	-2.046921047	-1.177881725	-1.19759996	-0.351074441	-1.300448367
EPSTI1	0.00725021	2.857142857	-0.015957574	0.687060688	0.622930351	-0.279283757	-1.53951953	-1.20756107	-0.826232932	-1.336427665
ERBB3	9.48E-05	0.121088435	-3.279283757	-3.011587974	-2.152003093	-1.937878288	0.847996907	0.516015147	0.189033824	0.584962501
ERCC6L	0.0424019	0.3045	-3.961282892	-3.74081792	-4.042159673	-4.50963525	-2.481968507	-2.932361283	-2.921390165	-1.481968507
ERFE	0.00158624	0.360406091	-5.673002535	-5.030324537	-6.276485124	-5.493296513	-4.383830534	-3.956795501	-3.772012541	-4.293358943
ERVMER34-1	0.00595897	2.263959391	-0.902389203	-0.883635243	-1.329159664	-1.689659879	-2.152003093	-2.083141235	-2.746615764	-2.473931188
ESPL1	0.000589328	0.015534591	-5.179187923	-5.725469955	-4.824188006	-5.878321443	0.411426246	0.35614381	0.5360529	1.201633861
ESPNL	0.0263203	0.462616822	-4.316168826	-4.601211852	-4.235144329	-4.221623189	-4.13796526	-3.224317298	-2.727379545	-3.158429363
ESRP1	0.00698793	2.683760684	1.510961919	2.09085343	1.673556424	1.169925001	-0.277533976	-0.260151897	0.910732662	0.214124805
ETNPPL	0.0210209	2.195187166	-0.01887801	0.084064265	-0.490050854	-0.940644722	-2.457989644	-1.02620507	-1.092340172	-1.49410907
ETV5	0.0011992	8.695054945	3.056583528	2.906890596	2.419538892	2.046141782	-0.106249498	-0.015957574	-1.2968993	-0.76611194
EVA1C	0.00267495	0.361413043	-2.556393349	-2.662003536	-4.886832943	-2.53951953	-1.836501268	-1.49410907	-1.332789088	-1.181149439
EXO1	0.0110781	0.072030651	-2.836501268	-2.251538767	-3.425075022	-1.694321257	0.695993813	0.748461233	1.416839742	2.163498732
EXTL3-AS1	0.00828351	2.170588235	-1.621488377	-1.506352666	-1.12973393	-1.54793177	-3.941198646	-2.095419565	-2.836501268	-2.052894948
EYA1	0.0499409	0.439613527	-0.498178735	0.150559677	0.042644337	-0.352915787	0.378511623	1.608809243	0.321928095	1.416839742
EZR-AS1	0.0181525	3.06122449	-3.011587974	-3.023269779	-2.954557029	-4.483581358	-4.42043364	-5.23786383	-4.310432456	-6.189680297
F10	0.0276812	0.339037433	-2.005782353	-1.430508908	-1.577766999	-1.680382066	-0.865121946	0.250961574	0.485426827	-0.734563104
F11R	0.00078211	2.163179916	2.63691458	2.397802962	2.042644337	2.347665656	1.339137385	1.321928095	1.014355293	1.316145742
F2RL2	0.025642	2.330409357	-3.556393349	-3.070966521	-3.904008087	-4.377819296	-5.173970214	-4.594225422	-4.714065192	-5.078259014
F5	0.00852964	2.168734491	-3.498178735	-3.950090478	-3.307572802	-3.388355457	-4.625934282	-4.832385159	-5.60823228	-3.938984225
F8	0.00349971	2.123430962	-2.279283757	-2.095419565	-2.164884385	-2.756330919	-3.832385159	-3.503077534	-3.446934456	-2.921390165
FA2H	0.00473035	0.316883117	-2.878321443	-3.171368418	-2.910501849	-3.184424571	-0.979942348	-1.114035243	-1.625934282	-2.005782353
FAAH2	0.00218074	0.344927536	-1.244685096	-2.279283757	-0.646112164	-0.646112164	0.137503524	0.411426246	0.389566812	0.831877241
FAAP24	0.0182076	0.413333333	-2.351074441	-2.426625474	-1.960159735	-2.10780329	-0.943416472	-1.675765438	-0.373327247	-1.011587974
FADS2	0.0310947	0.312348668	4.137503524	3.95419631	3.021479727	3.378511623	5.949534933	5.768184325	4.738767837	4.465974465
FAM111A	0.0170332	0.3	-1	-0.708396442	-0.907792562	-0.682695932	0.097610797	0.831877241	0.773996325	1.580145484

FAM111B	0.0168742	0.002953782	-8.702749879	-7.150208856	-5.938984225	-8.720897226	0.070389328	0.263034406	1.875780063	1.843983844
FAM114A1	9.12E-06	0.358925144	0.613531653	0.847996907	0.86393845	1.220329955	2.414135533	2.498250868	2.292781749	2.304511042
FAM124A	0.0146508	0.379850746	-0.926865295	-1.20756107	-0.905088353	-0.883635243	0.739848103	-0.694321257	0.669026766	0.565597176
FAM133A	0.00117266	0.461578947	-0.109358756	-0.281035664	-0.652901329	0.163498732	0.622930351	1.13093087	0.847996907	1.049630768
FAM155A	0.00597915	2.218579235	-1.502259911	-1.184424571	-1.152003093	-1.395928676	-3.265344567	-3.342268543	-2.058893689	-1.790858602
FAM181A	0.0447211	2.878411911	-2.652901329	-4.13289427	-3.046921047	-3.011587974	-3.606473968	-3.840629153	-7.930160375	-7.546245393
FAM181A-AS1	0.0282058	3.294117647	-5.41734766	-4.150721267	-5.442222329	-4.988504361	-6.85141726	-7.40506933	-6.64819078	-5.947862377
FAM198B	1.42E-05	3.432835821	4.491853096	4.357552005	4.385431037	4.224966365	2.599317794	2.307428525	2.241840184	3.070389328
FAM198B-AS1	0.0080246	2.974588939	1.50589093	0.815575429	0.765534746	0.713695815	-0.234465254	-0.418889825	-2.836501268	-0.090802937
FAM209A	0.0315634	3.058823529	-3.775959726	-3.450084446	-4.66566056	-4.451662024	-6.813600866	-4.457989644	-7.28771238	-5.587272661
FAM213B	0.000413816	0.382178218	2.746312766	2.931683057	3.042644337	3.053111336	4.40599236	4.169925001	4.62058641	4.078951341
FAM220CP	0.00539935	2.319444444	-3.930160375	-4.168771307	-3.801883071	-3.754382647	-5.467533417	-5.184424571	-6.158429363	-4.293358943
FAM230A	6.63E-05	2.955357143	-0.775959726	-0.50021788	-0.420819852	-0.713118852	-2.564904848	-2.158429363	-1.841662973	-2.152003093
FAM230B	6.08E-05	9.080068143	-1.061902439	-0.619270551	-0.826232932	-1.200912694	-4.192315357	-4.262572817	-3.963531833	-3.965784285
FAM43A	0.0131102	3.184466019	1.03562391	1.169925001	0.23878686	0.084064265	-0.722610301	-0.351074441	-1.943416472	-1.279283757
FAM43B	0.00268591	0.318047337	-4.878321443	-5.442222329	-6.546245393	-5.764150423	-3.531156057	-3.779917739	-4.006941609	-4.359934417
FAM46C	0.00103337	4.69858156	-1.573466862	-1.800877358	-1.960159735	-2.457989644	-4.423526235	-3.334971132	-4.66566056	-4.622376462
FAM53B	0.00645225	0.481111111	-0.150400989	0	-0.504304837	-0.229382353	1.280956314	0.757023247	0.485426827	0.765534746
FAM53B-AS1	0.0195095	2.186915888	-2.878321443	-3.764150423	-3.307572802	-2.671163536	-4.590744853	-4.676687582	-3.820106829	-3.997693533
FAM72A	0.0220679	0.140336134	-0.763660461	-0.492078535	-1.556393349	-1.465938398	1.411426246	0.23878686	2.381283373	2.381283373
FAM72B	0.0204281	0.154945055	-1.657445255	-1.486004021	-1.816037165	-2.564904848	0.495695163	-0.659722595	1.454175893	1.321928095
FAM72C	0.0113493	0.117112299	-2.171368418	-1.535331733	-2.457989644	-2.977099598	0.704871964	-0.531156057	1.280956314	1.448900951
FAM72D	0.0107973	0.132510288	-1.727379545	-1.086201035	-1.64385619	-2.351074441	1.014355293	-0.023269779	1.735522177	1.757023247
FAM83D	0.00376028	0.031842105	-3.729291666	-3.846843212	-6.985758984	-4.615287038	0.790772038	-0.550042516	0.545968369	1.150559677
FAM91A1	0.0119742	2.012903226	1.82374936	1.411426246	1.59454855	1.700439718	1.077242999	0.250961574	-0.724992953	1.201633861
FAM95A	0.0249098	2.806122449	-3.988504361	-2.625934282	-2.954557029	-3.580353247	-5.48681248	-4.832385159	-4.075824085	-4.640253953
FAM95C	0.00817535	4.973451327	2.063502942	3.168321116	2.370164281	2.053111336	-0.369594529	0.584962501	0.344828497	-0.040971781
FANCA	0.000281136	0.269896194	0.367371066	0.847996907	0.584962501	0.722466024	2.324810603	2.707082992	2.22650853	2.784503983
FANCD2	0.0181234	0.141326531	-1.926865295	-1.857259828	-1.675765438	-1.971430848	0.443606651	0.5360529	0.584962501	1.815575429
FANCG	0.00674167	0.273794003	-3.340806105	-1.556393349	-1.971430848	-2.785875195	-0.886299501	-0.849440323	-0.001443417	-0.042456799
FANCI	0.00430697	0.250666667	0.839959587	1.298658316	0.925999419	0.475084883	2.523561956	2.333423734	3.184280294	3.336283388
FAS	6.81E-07	4.581589958	4.517275693	4.459431619	4.510961919	4.314696526	1.906890596	2.482848283	2.176322773	2.392317423
FAT2	0.0174073	2.055921053	-3.23786383	-3.13289427	-2.564904848	-3.184424571	-3.772012541	-3.647467443	-5.467533417	-3.886832943
FBLN1	0.00149968	0.303463203	-0.375197235	-0.282789701	-0.632628934	-0.826232932	1.220329955	1.03562391	1.627606838	0.815575429
FBLN2	0.02406	3.968911917	1.847996907	2.678071905	1.550900665	1.250961574	-0.588573754	0.98550043	-0.865121946	-0.60164963
FBXL19-AS1	0.0170846	2.576271186	-0.242976753	-0.739372092	-0.805912948	-1.261880711	-1.268816758	-2.152003093	-3.950090478	-2.064917477
FBXO17	0.00116781	0.394238683	-0.10159814	-0.732164608	0.201633861	0.201633861	1.292781749	0.879705766	1.541019153	1.339137385
FBXO22	0.000476137	2.155525239	4.008988783	3.744161096	4.070389328	4.078951341	3.065227623	2.397802962	2.673556424	3.214124805
FBXO32	0.000594266	2.858108108	5.5360529	5.426264755	5.318316841	5.318316841	4.510961919	2.440952198	3.560714954	4.263034406
FCGR1A	0.00583603	3.216216216	-1.03209363	-1.358453971	-1.61705613	-2.158429363	-2.846843212	-2.921390165	-4.158429363	-3.058893689
FCGR1CP	0.0105214	6.231422505	-3.46434514	-4.684086035	-3.851000837	-4.799872346	-5.733123528	-7.135934725	-7.795859283	-7.093940636
FCRLA	0.0318785	7.736842105	1.049630768	1.263034406	-0.229382353	-0.857259828	-1.708396442	-1.473931188	-4.54962012	-4.336427665
FDXR	0.000960687	4.787644788	6.754887502	7.400879436	6.857980995	6.686500527	4.044394119	4.832890014	5.371558863	4.087462841

FEN1	0.00718401	0.285526316	-0.554273297	-0.502259911	-0.899695094	-0.55851652	0.831877241	0.713695815	1.169925001	1.778208576
FEZ1	0.00160187	2.234285714	2.301587647	1.937344392	1.794935663	1.765534746	0.879705766	0.389566812	1.111031312	0.773996325
FGD5	0.0160082	2.922178988	-3.615287038	-3.842697534	-3.293358943	-4.402016006	-5.097887821	-4.284897364	-6.200249538	-6.871548215
FGF10	0.000773019	2.734513274	-1.510457064	-1.49410907	-1.960159735	-1.862496476	-3.400491764	-3.083141235	-3.684086035	-2.61705613
FGF18	0.0443312	0.485714286	-0.529072743	-0.836501268	-0.205896101	-0.195946441	0.5360529	0.070389328	1.269033146	0.321928095
FHL2	0.0343088	0.376086957	8.50779464	7.924812504	9.278449458	9.729620744	10.28771238	9.30833903	10.9068906	10.75655632
FIGNL2	0.0240359	0.423269809	-1.184424571	-0.924125133	-1.411195433	-1.53951953	-0.164884385	0.632268215	-0.349235441	-0.397828209
FKBP7	0.0301289	0.423560209	-0.459972731	-0.448114897	-0.168122759	-0.17299399	0.695993813	0.028569152	1.500802053	1.111031312
FLJ32255	0.00218255	2.057842047	-2.217591435	-2.426625474	-2.64385619	-2.473931188	-3.706511798	-3.23786383	-3.046921047	-4.171368418
FLJ42393	0.0484602	2.105263158	-5.658355759	-6.127841043	-5.333516069	-5.673002535	-8.066608654	-6.88172002	-7.540862196	-5.673002535
FLT1	0.00319207	2.468531469	2.182692298	1.769771739	1.659924558	1.599317794	0.757023247	0.82374936	-0.756330919	0.739848103
FLVCR2	0.0112079	0.490196078	-2.490050854	-3.046921047	-4.299027693	-2.746615764	-2.190997225	-1.883635243	-2.217591435	-1.666576266
FMNL2	0.0197386	2.172330097	3.163498732	3.632268215	2.929790998	2.757023247	2.163498732	2.336283388	0.613531653	2.435628594
FNDC10	0.026116	0.408854167	0.879705766	1.042644337	0.22650853	0.263034406	2.003602237	1.851998837	2.503348735	1.042644337
FNDC5	0.0025764	0.164238411	-0.459972731	-0.710755715	0.584962501	1.070389328	2.280956314	2.746312766	3.419538892	2.983677695
FOLR1	0.0432756	0.481313131	-3.120294234	-2.717856771	-5.194955239	-3.522840789	-2.698997744	-2.746615764	-1.846843212	-2.251538767
FOS	0.0044377	2.894308943	-1.217591435	-1.722610301	-1.354759487	-1.736965594	-3.746615764	-2.017417053	-3.611755347	-3.521183471
FOXI3	0.0142709	2.403703704	-3.461163892	-4.254289378	-3.775959726	-4.526161147	-5.861447625	-5.044538396	-5.092955525	-5.002310161
FOXL2NB	0.00837411	3.169642857	-3.307572802	-3.731206325	-3.974829424	-4.493296513	-5.127841043	-5.002310161	-6.24331826	-5.904008087
FOXM1	0.00461357	0.099730094	-1.058893689	0.084064265	-0.148800661	-0.979942348	3.336283388	1.627606838	3.068670811	3.003602237
FOXRED2	0.00171144	0.470588235	0.669026766	0.632268215	0.678071905	0.739848103	2.097610797	1.40599236	1.757023247	1.722466024
FREM1	0.00547799	2.945578231	5.205548911	5.942514505	5.236492618	5.209453366	3.689299161	3.807354922	3.160274831	4.517275693
FRMPD2	0.0133102	2.215277778	-1.888968688	-1.395928676	-1.77102743	-1.582079992	-2.954557029	-2.490050854	-5.127841043	-2.064917477
FSD1	0.0264185	2.335135135	1.10433666	1.669026766	0.910732662	0.516015147	-0.518701058	-0.199255376	0.584962501	-0.673462652
FUT8-AS1	0.00213756	3.543956044	-4.148161027	-3.647467443	-3.756330919	-4.383830534	-5.733123528	-5.020925839	-7.378419294	-5.869859865
GABRB2	0.000374132	10.10718114	0.084064265	0.263034406	-0.236163541	-0.584241333	-3.19759996	-4.31329779	-3.917024973	-2.76611194
GADD45A	1.71E-05	3.214574899	6.462706751	6.264911693	6.300123725	6.205548911	4.832890014	4.857980995	3.944858446	4.705977902
GAL	0.0121614	2.314049587	-2.272297327	-2.434402824	-2.878321443	-2.775959726	-3.095419565	-3.625934282	-6.088040035	-3.689659879
GAL3ST4	8.65E-05	6.323185012	-1.785875195	-1.694321257	-2.258425153	-1.873027144	-4.19759996	-3.888968688	-5.392894616	-5.316168826
GALNT13	0.000463856	2.074198988	0.389566812	0.485426827	0.23878686	0.056583528	-1.023269779	-0.623709617	-0.921390165	-0.510457064
GALNT17	0.00705442	2.465116279	2.904965719	3.003602237	2.232660757	2.392317423	1.799087306	1.718087584	0.695993813	0.963474124
GALNT18	0.00112614	0.411917098	0.659924558	0.475084883	0.475084883	1	2.10433666	1.819668183	1.584962501	2.198494154
GALNT5	1.90E-05	3.902439024	1.454175893	1.232660757	1.22650853	1.117695043	-0.524915117	-1.293358943	-0.846843212	-0.323732592
GALNT7	0.00128475	2.01826484	2.084064265	1.871843649	2.344828497	2.23572706	0.871843649	1.422233001	0.765534746	1.344828497
GALR2	0.00640773	5.145228216	-2.899695094	-2.426625474	-3.660178495	-3.339345148	-5.097887821	-5.254289378	-4.748553568	-7.987588655
GAS6-AS1	0.000108219	3.514150943	1.344828497	1.575312331	1.847996907	1.480265122	-0.212567535	0.042644337	-0.246395464	-0.603840511
GAS8-AS1	0.0242526	2.198863636	-5.351074441	-4.24879339	-4.736965594	-4.629500897	-5.299027693	-5.702749879	-7.359342057	-5.658355759
GBP5	0.000618528	3.077844311	-0.878321443	-0.717856771	-1.03209363	-1.272297327	-1.965784285	-2.826232932	-3.365871442	-2.522840789
GCC2-AS1	0.0167162	2.286821705	-3.66383089	-3.224317298	-2.59946207	-3.046921047	-3.901849979	-5.304718805	-4.224317298	-4.039784866
GCSAM	0.000193507	3.297587131	0.310340121	0.014355293	0.565597176	0.250961574	-1.329159664	-1.15521265	-2.077041036	-1.293358943
GDF1	0.00710646	0.489265537	-1.123433941	-0.924125133	-1.23786383	-1.63039393	-0.008682243	-0.150400989	0.084064265	-0.77102743
GDF15	0.000347614	13.44086022	3.392317423	3.733354341	3.168321116	2.871843649	0.22650853	-0.504304837	-0.61705613	-1.171368418
GDF2	0.0115229	3.310657596	0.948600847	0.970853654	-0.067938829	0.014355293	-0.687334826	-1.61705613	-1.469929258	-1.13289427

GDNF	0.0183523	11.39303483	-0.248107862	0.201633861	-1.061902439	-1.873027144	-3.412731032	-3.988504361	-4.938984225	-4.279283757
GEM	0.00415001	2.612781955	4.112700133	3.99095486	3.548436625	3.392317423	2.994579724	1.718087584	2.313245852	2.3305584
GGH	0.0318074	0.111914894	-1.518701058	-1.857259828	-1.905088353	-2.652901329	0.422233001	-0.15682011	2.042644337	1.608809243
GGT5	0.0214141	3.716	-0.778432211	0.189033824	-0.052894948	0.042644337	-4.125321051	-4.414268267	-0.321928095	-3.365871442
GGTLC3	0.0289894	2.892523364	-1.756330919	-0.323732592	-0.210896782	-0.954557029	-2.899695094	-2.258425153	-1.446148032	-2.775959726
GINS1	0.0207624	0.196363636	-1.279283757	-1.680382066	-1.017417053	-0.968604804	1.063502942	-0.300448367	1.220329955	1.839959587
GINS4	0.0346134	0.423148148	-0.924125133	-1.008682243	-1.043943348	-1.652901329	-0.261880711	-0.526992432	0.124328135	0.773996325
GJB7	0.0289679	2.531073446	-5.163591068	-4.429731384	-4.073393259	-4.461163892	-4.706511798	-6.573466862	-6.24331826	-6.859771047
GLI2	0.00654707	3.07436182	1.5360529	1.622930351	1.687060688	0.895302621	-0.390245038	0.879705766	-2.184424571	-0.349235441
GLIDR	0.000789158	2.145061728	2.922197848	2.61117238	2.967168608	2.655351829	1.704871964	1.056583528	1.948600847	1.90303827
GLS2	1.91E-05	4.123337364	1.695993813	1.97819563	1.744161096	1.641546029	-0.808437349	0.097610797	-0.475936324	-0.074000581
GNG8	9.99E-05	0.102092676	-3.917024973	-4.13796526	-3.171368418	-4.669326877	-0.713118852	-0.582079992	-0.23786383	-0.862496476
GPA33	0.017274	5.368536854	-3.773984784	-3.974829424	-4.725469955	-5.594225422	-6.020925839	-7.576217473	-7.20237271	-6.811978949
GPER1	0.00195887	0.122321429	-5.532824877	-4.869859865	-5.904008087	-4.756330919	-2.190997225	-3.011587974	-1.732164608	-1.994240731
GPNMB	0.043358	3.983333333	3.364572432	3.485426827	1.974529312	1.678071905	1.23878686	0.5360529	0.82374936	0.704871964
GPR1	4.08E-05	0.4751693	2.17951105	2.185866545	1.695993813	2.176322773	3.201633861	3.153805336	3.217230716	3.003602237
GPR137B	0.0014649	2.117263844	1.13093087	1.111031312	0.963474124	0.575312331	-0.256700472	0.250961574	-0.187707155	-0.352915787
GPR158	0.000125399	2.660660661	0.014355293	-0.150400989	-0.241270432	-0.3382504	-1.377069649	-2.184424571	-1.675765438	-1.268816758
GPR162	0.0115104	0.23907563	-0.994240731	-1.595096878	-0.575615328	-0.369594529	1.049630768	1.077242999	1.925999419	0.604071324
GPR173	0.0349247	0.334545455	-1.2968993	-1.314732593	-0.597277823	-0.434402824	0.084064265	1.49057013	0.411426246	0.475084883
GPR37	0.00390452	2.221311475	-1.595096878	-2.058893689	-2.126580497	-1.816037165	-2.671163536	-3.083141235	-4.120294234	-2.698997744
GPR50	0.00535843	6.513409962	1.014355293	1.244887059	0.321928095	0.22650853	-2.988504361	-0.382701517	-3.733123528	-3.689659879
GPR85	0.00031011	3.780160858	0.613531653	0.333423734	0.59454855	0.422233001	-2.052894948	-2.775959726	-0.496142467	-1.332789088
GPR88	0.000701886	5.904761905	-4.729291666	-4.127841043	-4.442222329	-4.934565554	-7.356975042	-8.495898308	-6.573466862	-6.645299607
GPSM1	0.0324589	0.411564626	3.485426827	3.608809243	3.678071905	3.584962501	4.95419631	5.539158811	4.357552005	4.300123725
GPX2	0.0248191	2.65795207	-3.795859283	-2.727379545	-2.736965594	-3.10780329	-5.04930764	-5.025617534	-5.374823043	-3.343732465
GPX8	0.0112214	0.438248848	3.14404637	3.073820233	3.27351589	3.472487771	4.399171094	3.700439718	4.852997588	4.572889668
GREB1L	0.00121291	2.226950355	1.879705766	1.632268215	1.541019153	1.531069493	0.739848103	0.622930351	-0.514573173	0.782408565
GRIA1	0.00776658	25.13661202	0.124328135	1.163498732	0.604071324	-0.603840511	-3.828280761	-4.284897364	-4.330610338	-4.368849142
GRIK1	0.0247314	12.55977496	0.310340121	0.5360529	-0.734563104	-1.883635243	-3.651087759	-3.432843996	-5.356975042	-3.50963525
GRIK4	0.00444905	4.47761194	-2.286304185	-2.017417053	-2.910501849	-2.888968688	-4.383830534	-4.74081792	-3.770042991	-8.708773666
GRIN2A	0.0248425	2.158730159	-2.286304185	-2.867752202	-3.224317298	-3.383830534	-3.880444615	-4.061300187	-3.947862377	-4.075824085
GRIN2B	0.000536607	3.414225941	-3.467533417	-3.527824196	-3.857259828	-3.636660688	-5.270904092	-6.467533417	-6.078259014	-4.522840789
GRIP2	0.000285331	8.035714286	3.320484678	3.485426827	3.019701914	2.750606505	1.007195501	-0.140825544	-1.586405918	0.298658316
GSDMD	0.0330132	0.424460432	-4.135427537	-2.826232932	-2.53951953	-3.293358943	-2.556393349	-1.430508908	-1.450084446	-2.279283757
GSDME	0.00131269	2.397408207	3.548436625	3.807354922	3.277984747	3.185866545	1.918386234	2.40599236	2.283921772	2.195347598
GSTT2	0.0157135	0.469902913	-0.192645078	0.286881148	0.097610797	-0.488026018	0.773996325	1.15704371	1.510961919	0.5360529
GSTT2B	0.0137225	0.462274176	-1.184424571	-0.862496476	-1.123433941	-1.77102743	-0.360304767	0.042644337	0.35614381	-0.577766999
GTSE1	0.00278751	0.043945578	-4.840629153	-4.153286059	-3.575185379	-3.585539694	0.887525271	-0.258425153	0.189033824	1.042644337
H1FX-AS1	0.0138074	0.395384615	-4.221623189	-3.367359524	-3.559791925	-3.773984784	-3.145605322	-2.023269779	-1.994240731	-2.556393349
H2AFX	0.000227689	0.279821958	0.084064265	0.150559677	-0.384583703	-0.260151897	1.871843649	1.333423734	1.956056652	1.765534746
H2BFXP	0.0322304	0.486956522	-3.51292532	-2.457989644	-3.251538767	-3.750493979	-2.465938398	-2.53951953	-1.63039393	-2.046921047
HAP1	0.0181297	3.84057971	-0.121863233	0.263034406	-0.614845103	-1.300448367	-2.522840789	-1.347398782	-2.785875195	-3.095419565

HAS2	0.0495063	2.488095238	-4.787866492	-3.731206325	-3.361416385	-4.573466862	-6.276485124	-5.506352666	-5.073393259	-4.787866492
HASPIN	0.0267862	0.052325581	-7.419815916	-6.429731384	-7.221623189	-8.434714792	-2.943416472	-4.725469955	-3.19759996	-2.058893689
HAUS4	0.015153	0.493630573	2.053111336	1.851998837	2.403267722	2.475084883	2.9202933	2.769771739	3.64385619	3.432959407
HAUS7	0.023727	0.353521127	-1.029146346	-0.937878288	-0.940644722	-1.077041036	0.042644337	0.111031312	1.214124805	0.298658316
HAUS8	0.00196592	0.327083333	-0.120294234	-0.205896101	-0.502259911	-0.625934282	1.097610797	0.739848103	1.521050737	1.545968369
HCG27	0.049075	0.404705882	-3.649276466	-2.395928676	-2.114035243	-2.426625474	-2.442222329	-0.64385619	-0.971430848	-1.450084446
HCN1	0.00299041	2.157330155	-2.380821784	-2.293358943	-2.708396442	-2.522840789	-3.503077534	-4.566613191	-3.624154275	-3.011587974
HDAC2-AS2	0.0137525	2.293333333	-3.448508591	-4.04930764	-4.042159673	-4.002310161	-4.891107598	-4.799872346	-7.364087768	-4.493296513
HELLS	0.00755512	0.160989011	-0.465938398	-0.606034724	-0.888968688	-1.244685096	0.815575429	1.871843649	1.843983844	2.46727948
HEPACAM	0.0325446	0.263157895	-6.78150399	-5.710283552	-6.601211852	-8.217323052	-4.930160375	-3.878321443	-5.622376462	-4.702749879
HERC5	0.0170015	3.944134078	-1.340075442	-0.844250767	-1.926865295	-2.351074441	-3.423526235	-3.095419565	-3.491672771	-4.088040035
HES1	0.00159466	2.191111111	2.475084883	2.419538892	2.111031312	2.169925001	1.416839742	1.531069493	0.201633861	1.189033824
HHIPL1	0.0101739	2.092050209	-2.736965594	-2.590744853	-2.490050854	-3.251538767	-3.855170479	-3.752437003	-3.23786383	-4.714065192
HIF3A	0.00244691	0.369028007	4.232660757	3.981852653	4.62058641	4.930737338	6.102238194	5.578938713	6.247927513	5.652486495
HILPDA	0.039783	0.425308642	-0.354759487	-1.13289427	-0.36215794	-0.438307279	0.622930351	-0.234465254	1.339137385	0.632268215
HIST1H2BF	0.0238802	4.532871972	-2.329159664	-4.148161027	-2.522840789	-3.411195433	-5.66566056	-4.483581358	-5.143054137	-5.442222329
HIST1H2BG	0.000536673	2.997987928	0.887525271	0.475084883	0.622930351	0.250961574	-0.833927324	-0.724992953	-1.286304185	-1.282789701
HIST1H4E	0.00328391	4.055555556	-0.224317298	-0.00433459	-0.120294234	-1.187707155	-2.775959726	-1.836501268	-2.251538767	-2.671163536
HIST1H4H	0.000478829	2.466666667	1.835924074	1.604071324	1.475084883	1.286881148	0.526068812	0.275007047	0.250961574	-0.070966521
HIST2H2AA4	0.0128132	2.012106538	3.23572706	3.321928095	2.981852653	2.560714954	1.565597176	2.289834465	2.560714954	1.469885976
HIST2H3D	0.000886132	4.098360656	-2.899695094	-2.910501849	-2.795859283	-3.49980982	-4.756330919	-5.573466862	-4.229720657	-6.532824877
HJURP	0.00278347	0.009929907	-4.811978949	-3.279283757	-6.200249538	-5.803896602	1.646162657	1.316145742	2.430285273	2.608809243
HMGB2	0.0124956	0.397701149	3.847996907	3.548436625	4.485426827	4.357552005	4.87282876	5.498250868	5.224966365	5.956521363
HMGN5	0.0294428	0.481595092	-2.011587974	-1.531156057	-1.351074441	-1.899695094	-1.061902439	-1.095419565	-0.074000581	-0.496142467
HMMR	0.0111643	0.067647059	-1.577766999	-1.77102743	-2.775959726	-2.070966521	1.604071324	0.422233001	2.456806149	2.353323291
HNRNPA1P33	0.0018887	2.136422136	-2.805912948	-2.746615764	-2.265344567	-2.60823228	-3.542878542	-4.232429944	-3.477140745	-3.606473968
HPDL	0.00464566	0.442508711	-3.058893689	-3.184424571	-2.680382066	-3.046921047	-2.244685096	-1.486004021	-1.582079992	-2.035046947
HPN	0.0182615	0.345844504	-2.177881725	-1.666576266	-1.988504361	-2.035046947	-0.849440323	0.176322773	-0.256700472	-1.110915901
HS3ST1	0.00101633	3.058823529	-4.359934417	-4.526161147	-4.445362036	-5.13796526	-5.921390165	-6.559791925	-6.221623189	-6.13796526
HS3ST4	0.00834302	24.26160338	-3.48681248	-2.64385619	-2.60823228	-4.42043364	-7.565246355	-8.380821784	-7.039784866	-8.338177447
HSD3B7	0.04187	0.335582822	-1.358453971	-0.746615764	-0.81857936	-0.655171503	0.263034406	0.150559677	1.521050737	0.422233001
HSD3BP4	0.0211602	2.434146341	-8.470089122	-7.612460993	-7.272018572	-7.485519163	-11.00675607	-8.501116018	-8.895394957	-8.459893355
HSPA4L	0.000403904	2.37254902	3.267535798	2.965322548	3.007195501	3.0721058	1.627606838	2.13422094	1.137503524	2.201633861
HSPB6	0.00188191	0.224780702	-5.49980982	-5.710283552	-5.717856771	-5.519528055	-3.927962819	-3.682232861	-2.977099598	-3.41734766
HTRA3	0.0151016	0.483471074	-2.888968688	-4.145605322	-3.120294234	-2.625934282	-2.077041036	-2.61705613	-1.727379545	-1.910501849
IER5	0.00819775	2.156626506	2.608809243	2.381283373	2.451540833	2.232660757	1.427606173	2.066950244	-0.182786076	1.14404637
IFI30	0.0130207	0.379690949	1.084064265	0.422233001	0.659924558	0.86393845	2.066950244	1.937344392	2.771885579	1.713695815
IFIT1	0.0372195	0.281725888	0.82374936	0.887525271	1.257010618	1.510961919	2.684818738	1.427606173	3.62058641	3.336283388
IGF2	0.00153307	0.354455446	5.10433666	4.87282876	5.137503524	5.462706751	6.686500527	6.364572432	7.055282436	6.432959407
IGFBP5	0.00165825	0.19537037	-0.673462652	-0.883635243	-0.258425153	0.454175893	2.358958826	1.948600847	1.485426827	2.456806149
IGFBPL1	0.00108776	4.259259259	0.847996907	0.622930351	0.124328135	0.097610797	-1.625934282	-1.639354798	-1.634867407	-1.595096878
IGFL2	0.00192436	4.229508197	-0.974262439	-0.562772261	-0.888968688	-1.569179503	-3.773984784	-2.365871442	-3.527824196	-2.888968688
IL17RC	0.0249902	0.405405405	4.426264755	4.638073837	4.857980995	5.031218731	5.572889668	5.862947248	6.714245518	5.786596362

IL23A	0.0477749	2.361413043	-4.216250017	-3.544560985	-3.10780329	-3.437525542	-4.74081792	-3.638456202	-5.594225422	-6.838151005
IL31RA	0.0130339	2.816901408	-0.951763814	-1.148800661	-0.623709617	-0.356605547	-3.035046947	-1.117161344	-2.64385619	-3.184424571
IMPA1P1	0.0345887	2.115637319	-5.930160375	-5.601211852	-5.636660688	-7.045490984	-7.464982232	-6.795859283	-7.26091232	-6.753214946
INCENP	0.0184085	0.309489051	-0.212567535	-0.052894948	-0.434402824	-0.277533976	1.871843649	0.739848103	0.831877241	1.944858446
INHA	0.0398872	0.25840708	-0.619270551	-0.758769964	-0.12973393	0.432959407	1	1.835924074	2.584962501	0.992768431
INHBA-AS1	0.000109803	2.026548673	-1.977099598	-2.217591435	-2.13289427	-2.184424571	-3.323371512	-2.888968688	-3.035046947	-3.409661467
INPP1	0.00184913	2.491289199	2.831877241	3.229587923	2.698218478	2.488000771	1.495695163	1.565597176	1.584962501	1.427606173
INPP5D	1.38E-05	5.795454545	0.704871964	0.35614381	0.748461233	0.59454855	-1.395928676	-2.351074441	-2.272297327	-1.873027144
INS-IGF2	0.00177064	0.353333333	2.931683057	2.691534165	2.976363636	3.301587647	4.504620392	4.193771743	4.902073579	4.263034406
INTS6L	0.00050647	2.619047619	7.238404739	7.426264755	7.312882955	7.055282436	6.102238194	6.151777655	4.632268215	6.145677455
IQGAP2	0.0362428	2.3325	0.084064265	0.485426827	-0.461958547	-0.891642822	-1.994240731	-1.369594529	-1.055891201	-1.058893689
IQGAP3	0.000120711	0.014038462	-4.327710447	-5.200249538	-3.364384894	-4.070966521	1.752748591	1.773996325	2.198494154	2.40053793
IQSEC3	0.0177756	2.412451362	-3.601211852	-2.671163536	-2.756330919	-3.171368418	-5.063710705	-3.425075022	-4.921390165	-4.336427665
IRF1	0.010016	0.488673139	0.475084883	0.298658316	0.933572638	0.584962501	1.280956314	2.03562391	1.752748591	1.298658316
IRF7	0.0491917	0.216022099	-1.498178735	-0.883635243	-3.483581358	-0.783389931	0.475084883	0.622930351	1.786596362	-0.219269964
IRGM	0.0276499	2.168458781	-2.708396442	-2.954557029	-3.321928095	-3.279283757	-3.472329084	-3.54962012	-6.321928095	-4.832385159
IRX4	0.00132349	0.466431095	3.632268215	3.827819025	3.666756592	3.733354341	5.004501392	4.498250868	5.061776198	4.64385619
IRX6	0.0329131	4.146551724	-0.736965594	-0.356605547	-1.612637459	-2.210896782	-3.844768884	-2.244685096	-3.467533417	-3.442222329
ISG20	0.0445355	4.308300395	-0.935117148	0.98550043	0.137503524	-0.373327247	-3.772012541	-1.388355457	-1.184424571	-3.10780329
ITGA1	0.000336028	2.762589928	-1.268816758	-1.289827252	-1.380821784	-1.612637459	-2.61705613	-3.475535074	-3.307572802	-2.293358943
ITGA11	0.00325268	2.185089974	0.782408565	1.097610797	0.632268215	0.485426827	-0.075520008	-0.153606979	-1.171368418	-0.289827252
ITM2A	0.00123494	4.232673267	0.411426246	0.485426827	1.201633861	0.839959587	-1.304006187	-1.751465164	-1.329159664	-0.957355663
ITPR1	0.00380315	2.083916084	1.641546029	1.744161096	1.454175893	1.427606173	0.807354922	0.918386234	-0.839079812	0.584962501
ITPR2	0.00602704	2.450331126	0.584962501	0.895302621	0.250961574	0.464668267	-0.808437349	-0.109358756	-2.426625474	-0.446148032
IYD	0.0156794	2.651162791	-5.316168826	-4.442222329	-4.54962012	-5.429731384	-6.880019731	-5.702749879	-6.088040035	-6.753214946
JAG1	0.000405648	3.558091286	1.981852653	1.918386234	1.63691458	1.5360529	-0.048412205	0.678071905	-1.210896782	-0.224317298
JAKMIP2	0.00372714	0.453769559	-5.097887821	-4.448508591	-5.386845572	-5.122805453	-3.480357457	-4.115284871	-3.943416472	-3.861447625
JDP2	0.00889413	0.409411765	0.831877241	0.765534746	0.575312331	0.98550043	2.370164281	1.811471031	2.464668267	1.485426827
JMJD1C-AS1	0.00396508	2.886554622	-0.286304185	-0.19759996	-0.867752202	-0.974262439	-2.171368418	-1.60823228	-2.64385619	-2.035046947
JSRP1	0.00822409	7.091633466	-1.586405918	-0.392137097	-0.492078535	-1.59946207	-3.785875195	-4.095419565	-3.035046947	-4.371833001
KAZALD1	0.000192337	0.477037037	2.307428525	2.680324357	2.700439718	2.97819563	3.776103988	3.560714954	3.906890596	3.765534746
KBTBD8	0.000667882	2.475247525	-0.4639471	-0.54793177	-0.179514657	-0.496142467	-1.915935735	-1.282789701	-2.53951953	-1.465938398
KCNA5	6.05E-05	4.47037702	-1.736965594	-2.10159814	-2.017417053	-2.19759996	-3.8303315	-4.324816374	-4.799872346	-3.904008087
KCNC1	0.019437	0.239336493	-7.754772091	-9.714822711	-8.506352666	-6.429731384	-5.163591068	-6.917896955	-5.216250017	-5.553002759
KCNH8	0.0150277	2.962382445	-2.60823228	-1.910501849	-2.473931188	-2.785875195	-3.685941591	-5.820106829	-5.058893689	-2.943416472
KCNJ2	0.00136912	3.038167939	-0.152003093	-0.282789701	-0.332789088	-0.582079992	-1.486004021	-4.580353247	-2.070966521	-1.279283757
KCNJ2-AS1	0.000184267	3.628571429	-0.475936324	-0.506352666	-0.666576266	-1.029146346	-2.442222329	-3.083141235	-2.481968507	-2.177881725
KCNN1	0.000308121	0.357522124	-1.152003093	-1.2968993	-1.282789701	-1.531156057	0.411426246	0.214124805	0.22650853	-0.200912694
KCNQ3	0.00273469	6.62962963	1.280956314	1.13093087	0.367371066	0.321928095	-1.20756107	-1.821126042	-3.058893689	-2.040971781
KCNT2	0.0145839	3.180555556	-0.592919225	-1.736965594	-0.844250767	-1.671163536	-2.514573173	-3.035046947	-3.376320392	-2.450084446
KCTD12	0.000288523	2.662576687	1.280956314	0.847996907	1.14404637	1.169925001	-0.093879047	-1.152003093	-0.15521265	-0.035046947
KCTD8	0.0022332	2.81848929	-1.780908942	-1.846843212	-1.948975997	-2.53951953	-4.095419565	-2.867752202	-3.629500897	-3.673002535
KEL	0.0312152	0.399717514	-2.573466862	-1.543719518	-1.634867407	-1.732164608	-1.098505545	-0.985644707	0.137503524	-0.40354186

KHK	0.0405217	0.395652174	-0.040971781	-0.220950447	-0.430508908	-1.556393349	0.422233001	0.163498732	1.550900665	0.98550043
KIAA0513	0.0272599	2.005235602	2.344828497	2.09423607	1.59454855	1.570462931	1.575312331	0.948600847	-0.104697379	0.831877241
KIF11	0.0074402	0.072121212	-4.930160375	-4.41734766	-4	-4.371833001	-0.997117491	-1.713118852	-0.192645078	-0.06039728
KIF14	0.00876699	0.060891089	-3.210896782	-2.64385619	-2.442222329	-4.662003536	0.918386234	-0.195946441	1.021479727	1.718087584
KIF15	0.0146858	0.153648069	-1.214240226	-1.595096878	-1.74178261	-1.438307279	1	0.263034406	1.111031312	1.981852653
KIF18A	0.0225604	0.060705128	-5.345197874	-3.923577725	-3.035046947	-2.590744853	0.056583528	-1.083141235	1.292781749	1.176322773
KIF18B	0.00143692	0.199266504	-0.268816758	-0.139235797	-0.386468347	-0.397828209	2.10433666	1.416839742	1.952333566	2.464668267
KIF20A	0.0111446	0.021545894	-3.904008087	-2.746615764	-3.721658341	-3.930160375	2.372952098	1.189033824	1.201633861	2.767654798
KIF20B	0.0140801	0.156716418	-2.836501268	-2.671163536	-3.171368418	-2.465938398	-0.76121314	-0.805912948	0.056583528	0.632268215
KIF22	0.00542402	0.223529412	1.63691458	1.682573297	1.929790998	1.82374936	3.596935142	3.560714954	4.554588852	3.765534746
KIF23	0.00500203	0.094155844	0.367371066	0.454175893	0.526068812	0.757023247	3.584962501	3.046141782	4.240314329	4.491853096
KIF2C	0.00299582	0.056070288	-1.59946207	-1.465938398	-1.293358943	-1.722610301	2.709290636	1.925999419	2.367371066	3.255500733
KIF4A	0.00717767	0.03	-6.10780329	-5.795859283	-5.912672948	-6.117787378	-0.826232932	-1.926865295	-1.241270432	-0.200912694
KIF5C	0.0333346	3.112179487	-2.910501849	-2.846843212	-3.846843212	-4.395928676	-4.625934282	-4.702749879	-6.815224608	-4.756330919
KIFAP3	0.000667241	2.027027027	7.982993575	8	7.672425342	7.539158811	6.832890014	6.845490051	6.700439718	6.794415866
KIFC1	0.00264503	0.019136961	-3.095419565	-4.576905946	-3.095419565	-2.899695094	2.513490746	1.757023247	2.025028794	3.028569152
KITLG	5.08E-05	3.379888268	0.443606651	0.214124805	0.298658316	0.097610797	-1.522840789	-1.888968688	-1.736965594	-0.960159735
KLHDC7A	0.0293586	2.166666667	-1.657445255	-2.265344567	-2.286304185	-1.948975997	-3.541198058	-2.126580497	-4.002310161	-3.64385619
KLHL20	0.000521312	3.095541401	5.439623138	5.906890596	5.593951284	5.409390936	4.232660757	3.584962501	3.336283388	4.44625623
KLHL29	0.0255949	3.35600907	-1.746615764	-1.13606155	-1.76121314	-2.878321443	-2.785875195	-3.184424571	-5.702749879	-3.66383089
KLHL35	0.0466681	0.337995338	-5.733123528	-4.392894616	-5.117787378	-5.594225422	-4.150721267	-3.307572802	-2.836501268	-4.454822365
KLHL4	0.0107461	2.132653061	2.435628594	1.827819025	1.922197848	1.97819563	1.03562391	1.09085343	-0.369594529	1.531069493
KLKB1	0.0300121	3.802631579	-2.490050854	-1.002888279	-1.790858602	-2.388355457	-3.483581358	-3.23786383	-4.483581358	-3.972562817
KLLN	0.00630113	3.190661479	-1.727379545	-1.713118852	-2.388355457	-2.426625474	-3.959037452	-2.756330919	-4.208227596	-4.587272661
KLRF2	0.000612635	4.175365344	0.59454855	0.879705766	1.339137385	1.09085343	-0.926865295	-2.204233052	-0.758769964	-0.780908942
KNL1	0.0490173	0.090854392	-3.414268267	-4.051698188	-3.779917739	-3.735043282	0.097610797	-1.910501849	-1.318325858	0.659924558
KNSTRN	0.0312382	0.369158879	2.10433666	1.933572638	1.875780063	1.996388746	3.285402219	2.353323291	4.070389328	3.459431619
KNTC1	0.0426994	0.302158273	0.137503524	0.464668267	0.250961574	0.443606651	1.731183242	1.459431619	1.613531653	2.918386234
KPNA2	0.0141063	0.265384615	3.392317423	3.27351589	2.942983598	3.001802243	4.897240426	4.240314329	5.741466986	5.044394119
KPTN	0.014384	0.492242595	1.748461233	2.114367025	1.691534165	1.613531653	2.44625623	2.981852653	3.260025656	2.438292852
L1CAM	0.0487779	0.292046936	-4.993091631	-5.48681248	-6.493296513	-5.339345148	-4.392894616	-3.383830534	-2.965784285	-4.832385159
L3MBTL3	0.00343004	2.691867125	1.163498732	1.608809243	1.275007047	0.748461233	0.176322773	-0.036525876	-1.055891201	-0.142417045
LACC1	0.000237291	3.622722401	4.867896464	5.314696526	5.205548911	4.87774425	2.66448284	3.754887502	2.86393845	3.350497247
LAMA3	0.0286484	0.418831169	-2.077041036	-1.369594529	-2.258425153	-2.336427665	-0.76611194	-0.708396442	-1.531156057	-0.125006361
LARGE2	0.0260225	0.389982111	-2.498178735	-2.077041036	-1.921390165	-2.358453971	-1.358453971	-0.217591435	-0.706041021	-1.426625474
LCMT1-AS1	0.00190226	2.540229885	-0.756330919	-0.378944497	-0.473931188	-0.808437349	-2.836501268	-2.145605322	-1.971430848	-1.241270432
LCN9	0.00545446	18.12121212	1.23878686	2.304511042	1.40599236	1	-3.527824196	-2.634867407	-1.878321443	-2.816037165
LDHA	0.00794677	0.365384615	5.224966365	4.802193217	4.95419631	5.031218731	6.560714954	6.033423002	6.977279923	6.074676686
LDHD	0.00190083	0.39181916	1.98550043	1.695993813	1.879705766	1.879705766	3.198494154	3.378511623	3.485426827	2.666756592
LEFTY1	0.00513295	0.155454545	-6.068543859	-4.496549491	-5.13289427	-4.345197874	-2.76611194	-1.586405918	-2.61705613	-2.070966521
LEFTY2	0.00152687	0.1475	-1.486004021	-0.479954976	-0.543719518	0.097610797	1.495695163	2.565597176	2.555816155	2.192194165
LGALS3BP	0.0041753	0.255517241	2.678071905	3.13093087	2.757023247	2.948600847	4.666756592	4.995484519	5.343407822	4.177917792
LGALS8-AS1	0.00148805	2.24248927	-2.351074441	-2.231074664	-1.994240731	-2.498178735	-3.785875195	-3	-3.906169428	-3.210896782

LGSN	0.00266129	8.079710145	-2.145605322	-1.873027144	-1.841662973	-3.158429363	-4.925768606	-7.060818566	-4.398969131	-5.48681248
LHX1-DT	0.0380134	2.209737828	-2.867752202	-2.680382066	-3.224317298	-3.772012541	-6.168771307	-3.478748205	-4.597714408	-3.859352207
LIF	3.23E-05	3.762886598	1.298658316	1.14404637	1.014355293	1.056583528	-0.870387262	-0.826232932	-0.195946441	-1.535331733
LIMS1	7.90E-05	2.398809524	5.343407822	5.292781749	5.520422249	5.145677455	4	4.070389328	3.867896464	4.321928095
LIMS3	4.53E-05	3.700189753	1.15704371	0.765534746	1.049630768	0.847996907	-0.948975997	-0.831357964	-1.434402824	-0.60164963
LIMS3-LOC440895	0.00057272	4.057471264	-0.169744676	-0.785875195	-0.535331733	-0.588573754	-2.017417053	-2.506352666	-4.587272661	-2.10780329
LIMS4	0.000424314	3.582644628	3.432959407	2.895302621	3.10433666	2.969012308	1.63691458	1.298658316	0.042644337	1.622930351
LMTK3	0.00260058	0.339037433	-3.867752202	-3.585539694	-4.354021725	-4.246053228	-2.279283757	-2.164884385	-2.293358943	-3.095419565
LOXL1	0.020856	0.43902439	0.286881148	0.918386234	-0.131313235	0.042644337	1.35614381	2.09085343	1.201633861	1.232660757
LOXL4	4.92E-05	3.656934307	2.292781749	2.289834465	2.381283373	2.3305584	-0.064917477	1.263034406	0.189033824	-0.010134377
LPAR2	0.0130391	0.350694444	-2.756330919	-2.231074664	-1.932361283	-2.426625474	-1.142417045	-0.307572802	-0.537424112	-1.498178735
LPAR5	0.00266993	0.248717949	-5.710283552	-4.725469955	-3.457989644	-7.091971086	-3.145605322	-2.465938398	-2.54793177	-2.64385619
LRFN1	0.00160021	0.264204545	-1.446148032	-0.66428809	-1.377069649	-1.067938829	1.111031312	1.09085343	0.575312331	0.321928095
LRP1B	0.00293238	2.283950617	-5.710283552	-5.10780329	-5.74081792	-5.506352666	-6.333516069	-6.912672948	-6.601211852	-6.974829424
LRP2	0.0390852	2.81938326	-3.715959736	-3.330610338	-4.629500897	-4.633076351	-5.836501268	-5.74081792	-6.210896782	-4.611755347
LRRC3	0.0206211	0.499425947	-4.687799537	-4.580353247	-4.974829424	-4.020925839	-3.293358943	-3.145605322	-4.088040035	-3.744680559
LRRC38	0.0147092	3.300699301	0.275007047	0.111031312	-0.190997225	-0.717856771	-3.13289427	-0.488026018	-2.498178735	-2.846843212
LRRC3C	0.000557251	2.262135922	1.250961574	0.895302621	1.344828497	1.350497247	0.111031312	0.298658316	-0.459972731	0.124328135
LRRCC1	0.0161923	0.391975309	0.111031312	0.454175893	0.275007047	0.526068812	1	1.475084883	1.765534746	2.257010618
LRRK2	0.000948969	2.475409836	-0.841662973	-0.790858602	-0.810966176	-0.49410907	-2.481968507	-1.948975997	-2.59946207	-1.418889825
LRRTM2	0.00126282	3.025568182	-5.519528055	-5.983931631	-5.423526235	-5.356975042	-6.792656851	-7.871548215	-7.85141726	-6.573466862
LRRTM3	0.000339941	4.799196787	-1.114035243	-0.715485867	-1.139235797	-1.36215794	-3.307572802	-4.861447625	-3.083141235	-2.795859283
LSMEM1	0.00916357	2.509225092	0.23878686	0.855989697	0.59454855	-0.083141235	-1.590744853	-1.251538767	-0.343732465	-0.666576266
LUCAT1	0.00140705	2.432723358	1.150559677	1.49057013	1.220329955	0.748461233	-0.118726939	-0.356605547	0.028569152	-0.010134377
LY6E-DT	0.00151337	0.418627451	-4.908334012	-4.74081792	-4.411195433	-4.232429944	-3.738890471	-3.120294234	-3.307572802	-3.083141235
LYPD6B	0.0344067	3.825	0.879705766	1.350497247	-0.174621396	-0.217591435	-1.926865295	-1.526992432	-0.66428809	-1.477944251
LYPLAL1-AS1	0.0491845	0.382882883	-6.333516069	-7.08020992	-6.454822365	-4.760235373	-3.956795501	-5.092955525	-5.073393259	-4.205563338
LYRM9	0.0010292	2.189393939	2.275007047	2.627606838	2.704871964	2.485426827	0.970853654	1.15704371	1.883620816	1.416839742
LZTFL1	0.021514	2.094059406	1.859969548	1.63691458	2.596935142	2.039138394	1.244887059	0.23878686	0.956056652	1.378511623
MAB21L2	0.018681	2.005772006	-2.522840789	-2.698997744	-2.932361283	-3.362899876	-3.840629153	-4.477140745	-3.19759996	-4.229720657
MACROD2	0.00288037	0.435359116	-0.948975997	-0.582079992	-0.070966521	0.014355293	0.918386234	0.378511623	0.970853654	1.056583528
MAD2L1	0.026145	0.104678363	-2.746615764	-3.445362036	-2.231074664	-1.937878288	0.321928095	-1.061902439	1.214124805	1.475084883
MAFB	0.0144527	3.17373461	-1.60823228	-1.775959726	-2.64385619	-2.746615764	-3.251538767	-4.004624027	-3.995390747	-3.997693533
MAML3	0.00320091	2.037383178	-2.171368418	-2.442222329	-2.152003093	-2.040971781	-2.899695094	-3.811978949	-3.725469955	-2.746615764
MAP2	0.0134561	5.466237942	4.498250868	4.626439137	3.700439718	2.963474124	1.887525271	2.080657663	0.910732662	1.3950628
MAP2K6	0.00411055	0.4	-2.522840789	-2.473931188	-2.805912948	-2.652901329	-1.314732593	-1.943416472	-1.040971781	-1.017417053
MAP3K7CL	0.0115216	2.521008403	-1.694321257	-1.272297327	-2.244685096	-1.910501849	-2.652901329	-2.921390165	-4.594225422	-2.795859283
MAPK12	0.0113823	0.446854664	1.042644337	1.056583528	1.084064265	0.970853654	1.996388746	2.13093087	2.733354341	1.769771739
MAPT	0.0136061	3.12414734	1.257010618	1.773996325	0.941106311	0.526068812	-0.179514657	0.163498732	-1.092340172	-1.110915901
MARVELD2	0.0381805	2.108433735	-0.727379545	-1.722610301	-1.145605322	-0.430508908	-2.514573173	-1.732164608	-2.498178735	-1.53951953
MASP1	0.000114738	2.298245614	8.77148947	8.714245518	8.535275377	8.426264755	7.409390936	7.400879436	7.672425342	7.118941073
MASP2	0.044714	2.203125	-2.23786383	-2.265344567	-1.564904848	-1.430508908	-2.307572802	-4.684086035	-3.857259828	-2.293358943
MAST1	0.0330137	0.477272727	-3.023269779	-2.293358943	-3.307572802	-3.744680559	-2.064917477	-1.373327247	-2.152003093	-2.300448367

MAT1A	0.0374875	0.346982759	-6.710283552	-6.265344567	-6.506352666	-5.025617534	-4.615287038	-5.227016448	-4.54962012	-3.733123528
MATN1	0.00430241	2.524590164	-5.622376462	-4.760235373	-4.865647613	-4.979373349	-6.506352666	-6.64385619	-6.713308071	-5.787866492
MATN1-AS1	0.00537411	2.532967033	-3.046921047	-3.35254733	-3.876201392	-3.604717796	-5.025617534	-4.28771238	-5.601211852	-4.53616832
MAZ	0.00631384	0.328588375	4.786596362	4.738767837	4.760220946	4.87774425	6.431288654	6.118941073	6.930737338	5.8899602
MCAM	0.0201619	0.254672897	3.432959407	3.419538892	3.336283388	3.596935142	5.692092375	6.046578367	4.300123725	5.078951341
MCF2	0.014523	2.416851441	-3.10780329	-3.224317298	-2.888968688	-3.687799537	-3.511279347	-5.054092703	-4.714065192	-5.351074441
MCM2	0.00213631	0.178875639	-0.126580497	0.084064265	0.084064265	0.23878686	2.456806149	2.333423734	2.124328135	3.102658131
MCM3	0.00920864	0.405263158	2.364572432	2.117695043	2.084064265	2.250961574	3.548436625	3.321928095	2.952333566	4.017921908
MCM4	0.028027	0.470938897	1.752748591	1.480265122	1.570462931	1.815575429	2.469885976	1.948600847	3.182692298	3.063502942
MCM5	0.00394144	0.035387324	-2.321928095	-2.625934282	-2.380821784	-2.005782353	1.956056652	1.887525271	3.142413438	2.657640005
MCM6	0.00868276	0.121145374	-1.713118852	-2.083141235	-1.905088353	-1.76611194	0.516015147	0.389566812	1.704871964	1.622930351
MCM7	0.000488902	0.448160535	3.5360529	3.827819025	3.867896464	3.733354341	4.572889668	4.887525271	4.958842675	5.133399125
MDGA2	0.0328547	2.138888889	1.124328135	0.765534746	0.250961574	0.150559677	0.028569152	-0.498178735	-1.943416472	-0.166502663
MDM2	0.000458171	2.766666667	5.754887502	6.22881869	6.183883459	6.002252452	3.944858446	5.091699834	4.626439137	4.432959407
MEGF10	0.00201617	6.247379455	-1.461958547	-1.395928676	-2.358453971	-1.977099598	-4.636660688	-3.293358943	-6.158429363	-4.861447625
MELK	0.0123196	0.069264069	-0.582079992	-0.57132159	-0.272297327	-1.351074441	2.66448284	1.963474124	3.722466024	3.765534746
MELTF	0.000580261	3.065902579	3.498250868	3.711494907	3.364572432	3.058316496	1.807354922	2.269033146	1.411426246	1.580145484
METTL7B	0.000208091	3.108571429	-1.921390165	-2.190997225	-1.680382066	-1.77102743	-3.799872346	-3.507993024	-3.070966521	-3.81400663
MFAP4	0.0174553	0.144262295	0.495695163	0.028569152	0.650764559	1.604071324	3.300123725	2.871843649	4.426264755	3.336283388
MFSD4A	0.000579163	2.238938053	-2.070966521	-2.184424571	-1.954557029	-1.76121314	-3.389866924	-3.488430758	-2.736965594	-3.083141235
MGARP	0.0017163	2.92543021	-2.380821784	-2.61705613	-2.76611194	-3.19759996	-4.721658341	-4.423526235	-4.19759996	-3.832385159
MHRT	0.00897239	0.406349206	6.714245518	7.044394119	7.14974712	7.055282436	7.900866808	8.696967526	8.569855608	7.813781191
MIP	0.00119162	2.741784038	-3.952322025	-4.161007907	-3.863546091	-4.496549491	-6.011587974	-6.002310161	-5.073393259	-5.368849142
MIR1307	0.00460056	2.578125	-2.190997225	-2.035046947	-2.40354186	-1.883635243	-4.13796526	-2.634867407	-4.615287038	-3.327710447
MIR210HG	0.00404131	0.296851574	-2.272297327	-2.373327247	-2.418889825	-2.286304185	-0.637109357	-0.413115187	-0.179514657	-1.347398782
MIR3064	0.00205487	2.299516908	5.255500733	5.716990894	5.827819025	5.422905743	4.017921908	4.689299161	3.897240426	4.683696454
MIR34AHG	0.00104111	3.277372263	2.063502942	2.432959407	2.17951105	1.937344392	0.35614381	1.298658316	-0.971430848	0.298658316
MIR3936	0.0227428	0.424752475	-2.164884385	-1.446148032	-1.231074664	-0.518701058	-0.614845103	0.526068812	-0.005782353	-0.083141235
MIR4712	0.0198067	2.025462963	-0.239566125	-0.436353731	0.042644337	-0.17299399	-0.954557029	-3.095419565	-1.430508908	-0.465938398
MIR5047	0.0117017	2.394366197	5.339850003	5.314696526	6.085339669	5.799605422	4.277984747	4.498250868	3.275007047	5.061776198
MIR6820	0.0359831	2.88	-1.926865295	-1.13289427	-1.727379545	-2.943416472	-3.874084451	-2.689659879	-3.348133165	-3.647467443
MIR99AHG	0.0425495	0.39	-1.049904906	-1.077041036	-0.281035664	0.23878686	0.659924558	-0.171368418	1.250961574	1.469885976
MIS18BP1	0.0399309	0.461538462	-0.184424571	-0.330973234	0	-0.117161344	0.748461233	0.056583528	1.09085343	1.560714954
MKI67	0.00283873	0.004081146	-7.208761038	-5.725469955	-4.832385159	-7.013450718	2.241840184	1.333423734	1.659924558	2.671293372
MMAA	0.000523242	2.329317269	0.333423734	0.176322773	0.097610797	0.214124805	-0.780908942	-1.713118852	-1.311148256	-0.506352666
MMEL1	0.0184257	0.325221239	-0.780908942	-0.450084446	-0.036525876	-0.61705613	0.731183242	0.790772038	1.86393845	1.007195501
MMP2	0.00742883	2.244019139	2.289834465	2.646162657	2.060047384	1.790772038	0.992768431	1.469885976	1.117695043	0.526068812
MMP23B	0.0360959	0.12019544	1.731183242	1.704871964	1.815575429	2.22342255	4.399171094	4.578938713	5.918863237	4.10433666
MOXD1	0.00679395	2.143478261	-0.780908942	-0.886299501	-1.220950447	-1.248107862	-2.40354186	-3.10780329	-1.490050854	-1.948975997
MPP3	0.00581781	0.294776119	2.503348735	2.891419187	3.014355293	3.378511623	4.517275693	5.292781749	4.240314329	4.711494907
MROH5	0.0288034	2.406504065	-2.662003536	-1.64385619	-1.365871442	-1.657445255	-4.546245393	-2.272297327	-3.434402824	-2.736965594
MRPL23-AS1	0.0098485	2.116935484	-2.816037165	-2.139235797	-2.083141235	-2.083141235	-3.566613191	-4.23786383	-2.867752202	-3.035046947
MRVI1	0.00332147	5.893854749	-2.736965594	-1.790858602	-1.948975997	-2.76611194	-4.791857353	-4.356975042	-4.799872346	-5.467533417

MSH5	0.0332972	0.296735905	-1.098505545	0.516015147	0.250961574	-0.123433941	0.739848103	2.488000771	1.443606651	1.799087306
MSH5-SAPCD1	0.016556	0.297117517	-2.698997744	-1.399730246	-1.727379545	-2.070966521	-1.340075442	0.333423734	0.097610797	-0.184424571
MT1E	0.0163651	0.243362832	-1.556393349	-1.304006187	-0.698997744	-0.253257284	0.189033824	1.327687364	1.815575429	0.895302621
MT1F	0.0181017	0.101392111	-4.832385159	-4.546245393	-4.061300187	-2.23786383	-1.074000581	0.028569152	0.545968369	-1.03209363
MT1G	0.0494202	0.33006993	-4.687799537	-4.120294234	-4.48681248	-4.383830534	-3.184424571	-2.064917477	-4.110292842	-2.60823228
MT1X	0.0497009	0.312676056	-0.332789088	-0.428565884	0.378511623	0.678071905	0.604071324	2.518535139	2.207892852	1.23878686
MTFR2	0.0307949	0.18452381	-3.380821784	-3.058893689	-3.070966521	-4.736965594	-1.422752464	-2.114035243	-0.144010303	-0.979942348
MTHFD2P1	0.0246241	2.11751663	-5.993091631	-6.321928095	-5.526161147	-5.23786383	-7.26091232	-6.493296513	-7.150208856	-6.454822365
MTX3	9.24E-05	2.902027027	2.942983598	3.168321116	3.234194723	3.047887329	1.464668267	1.014355293	1.469885976	2.100977648
MUC19	0.000325476	9.540229885	-2.943416472	-2.888968688	-2.307572802	-2.343732465	-6.573466862	-5.411195433	-7.068543859	-5.143054137
MUC4	0.0482031	2.330935252	-5.23786383	-4.49980982	-5.64385619	-4.691522623	-5.28208783	-5.756330919	-8.097887821	-7.037887831
MYBL2	0.00830455	0.007151899	-6.392894616	-4.601211852	-6.200249538	-5.411195433	1.182692298	0.687060688	2.408711861	1.790772038
MYCBPAP	0.0399107	2.763157895	-2.556393349	-1.304006187	-2.373327247	-2.046921047	-4.122805453	-3.251538767	-5.10780329	-2.531156057
MYCNOS	0.00106841	2.047440699	0.443606651	0.782408565	0.963474124	0.604071324	-0.392137097	-0.504304837	-0.011587974	-0.424687669
MYH7	0.000166406	0.455339806	8.741466986	8.636624621	8.977279923	9.092757141	9.994353437	10.21674586	9.945443836	9.845490051
MYL2	0.0461975	0.340304183	7.491853096	6.918863237	7.562242424	7.826548487	8.839203788	8.527477006	9.861086906	8.438791853
MYLK	0.00237259	2.679245283	1.604071324	2.275007047	2.286881148	2.09423607	0.485426827	0.604071324	-0.027674958	1.298658316
MYLK3	0.000406318	2.302013423	6.251719093	6.040015679	5.963474124	6.12722055	5.108524457	4.649615459	4.285402219	5.325530332
MYO10	0.00379313	2.770053476	-0.659722595	-0.751465164	-1.046921047	-1.477944251	-2.662003536	-1.852042119	-3.330610338	-2.217591435
MYO1D	0.0352116	2.122762148	0.704871964	1.292781749	0.275007047	0.411426246	-0.226003675	-0.095419565	-1.713118852	0.042644337
MYO1H	0.00246483	3.206666667	-4.633076351	-3.923577725	-4.414268267	-4.669326877	-5.651087759	-6.701247854	-5.795859283	-6.321928095
MYO5B	0.00178571	3.700564972	-0.514573173	-0.190997225	-0.852042119	-1.03209363	-2.258425153	-2.418889825	-3.293358943	-2.258425153
MYO7A	0.00478601	0.234090909	-3.828280761	-3.719756304	-3.371833001	-2.564904848	-1.498178735	-0.800877358	-1.857259828	-0.849440323
MYOF	0.00100643	2.354285714	0.748461233	1.063502942	1.214124805	1.111031312	-0.696657606	-0.182786076	-0.486004021	0.367371066
MYOT	0.0299685	2.379166667	2.336283388	3.12763328	2.531069493	1.713695815	0.790772038	1.40053793	1.589763487	1.150559677
MZT2A	0.0427408	0.376494024	4.09592442	4.044394119	4.378511623	4.399171094	5.296457407	5.133399125	6.434628228	5.311067102
NABP1	0.000675699	3.284313725	1.03562391	1.176322773	1.220329955	0.475084883	-0.625934282	-0.977099598	-0.756330919	-0.516635639
NADSYN1	0.0126755	2.340807175	2.080657663	2.90303827	2.341985747	2.039138394	0.659924558	1.516015147	1.475084883	0.757023247
NAPRT	0.0490534	0.347607053	3.44625623	3.817623258	3.935459748	3.887525271	4.727920455	5.181897643	6.137503524	4.683696454
NAT1	0.0371778	0.468181818	0.189033824	0	-0.452056689	0.310340121	0.713695815	0.378511623	1.545968369	1.545968369
NCAPG	0.0209006	0.076643991	-1.98279071	-1.662003536	-0.915935735	-1.98279071	1.411426246	0.757023247	2.771885579	2.687060688
NCAPG2	0.0225611	0.172651934	0.263034406	0.275007047	0.604071324	0.111031312	2.430285273	1.879705766	2.843983844	3.666756592
NCAPH	0.00542395	0.029745223	-4.752437003	-4.53951953	-4.744680559	-3.846843212	0.815575429	-0.678071905	0.632268215	1.23878686
NDC80	0.0128661	0.011240876	-7.08020992	-4.570035956	-6.880019731	-8.093940636	-0.03209363	-1.083141235	1.049630768	0.970853654
NEFL	0.00249167	3.051591658	1.704871964	1.803227036	1.244887059	1.007195501	-0.564904848	0.333423734	-0.083141235	-0.395928676
NEFM	0.0042684	5.30162413	-0.907792562	-0.648371671	-1.373327247	-1.894321922	-3.925768606	-2.899695094	-4.181803871	-3.473931188
NEK2	0.00883423	0.008982456	-4.848920527	-5.221623189	-5.764150423	-5.467533417	1.432959407	-0.199255376	2.121015401	1.794935663
NEK6	0.00387442	0.476070529	1.063502942	0.799087306	0.641546029	1.117695043	2.257010618	2.060047384	1.454175893	2.066950244
NELL2	0.000563214	2.904884319	0.344828497	0.432959407	-0.058893689	-0.104697379	-1.461958547	-1.666576266	-1.254977851	-1.126580497
NEMP1	0.00900921	0.449864499	0.773996325	0.59454855	0.695993813	0.847996907	1.803227036	1.655351829	1.531069493	2.389566812
NET1	0.032908	0.43161435	-1.486004021	-1.522840789	-1.194294815	-1.329159664	-0.805912948	-0.76121314	0.163498732	0.35614381
NETO1	0.00485354	3.402985075	-0.873027144	-1.841662973	-1.300448367	-0.758769964	-3.428177593	-2.64385619	-3.307572802	-2.465938398
NFATC4	0.00034259	2.375478927	2.454175893	2.821710215	2.675815931	2.550900665	1.22650853	1.871843649	1.150559677	1.137503524

NFE2	0.00893332	0.489270386	-3.40812913	-4.324816374	-2.40354186	-3.035046947	-2.217591435	-2.224317298	-2.035046947	-1.954557029
NFKBIZ	0.00056258	2.130102041	0.669026766	0.887525271	0.82374936	0.5360529	-0.144010303	-0.364013496	-0.960159735	-0.092340172
NGF	0.00906679	3.529411765	-1.518701058	-1.369594529	-1.805912948	-2.717856771	-4.28208783	-3.171368418	-2.977099598	-4.48681248
NIPAL2	0.000212128	2.051282051	4.385431037	4.161887682	4.465974465	4.255500733	3.350497247	3.321928095	2.86393845	3.523561956
NKAIN2	0.0256863	3.903345725	0.214124805	0.765534746	-0.249822294	-1.035046947	-1.224317298	-2.029146346	-2.736965594	-1.98279071
NKD1	0.00423821	0.266542751	-3.636660688	-4.654717182	-3.925768606	-3.307572802	-1.392137097	-2.114035243	-2.442222329	-1.841662973
NKPD1	0.017212	2.843016069	-2.736965594	-1.510457064	-2.411195433	-2.126580497	-3.19759996	-4.171368418	-4.058893689	-3.334971132
NLGN4X	0.00786817	2.401847575	-0.069451881	0.333423734	0.124328135	-0.249822294	-1.311148256	-2.336427665	-1.522840789	-0.352915787
NLRP8	0.00988764	3.218604651	-7.312724268	-7.326552121	-7.702749879	-6.587272661	-8.26091232	-9.655444164	-9.315019726	-8.6266469
NME1-NME2	0.0452951	0.401834862	4.485426827	4.217230716	4.491853096	4.590961241	5.658211483	5.311067102	6.525129251	5.161887682
NME2	0.0374474	0.333333333	6.894817763	6.478971805	6.977279923	7.118941073	8.299208018	7.882643049	9.257387843	7.977279923
NME4	0.033121	0.169291339	3.972692654	3.754887502	4.689299161	4.949534933	6.595443985	6.338067798	7.906890596	6.475733431
NMNAT2	0.0238794	0.487116564	-1.810966176	-1.268816758	-1.142417045	-1.200912694	0.201633861	-0.446148032	-0.971430848	-0.210896782
NMRK2	0.00586171	0.351084813	4.439623138	4.137503524	3.935459748	4.03562391	5.9795681	5.532940288	5.974988112	4.935459748
NOVA1	0.00323938	3.642533937	4.145677455	4.432959407	3.847996907	3.40599236	2.280956314	1.13093087	2.469885976	2.344828497
NOX4	0.00754481	0.298245614	-2.54793177	-2.19759996	-2.785875195	-2.775959726	-1.029146346	-0.321928095	-0.612637459	-1.560642822
NPAS1	0.0138914	0.194444444	-1.552156356	-1.473931188	-1.621488377	-2.10159814	0.214124805	1.056583528	1.275007047	-0.307572802
NPAS3	0.00251003	2.752173913	-0.298672743	-0.60164963	-0.888968688	-0.948975997	-2.442222329	-2.954557029	-1.689659879	-1.751465164
NPB	0.0336791	0.430722892	-2.556393349	-2.052894948	-3.265344567	-4.158429363	-1.77102743	-1	-1.932361283	-1.867752202
NPPB	0.00462505	3.35071708	8.118941073	8.214319121	7.960001932	7.686500527	5.933100475	7.50779464	4.329123596	5.44625623
NPY1R	0.0163149	2.330677291	0.344828497	0.687060688	0.124328135	-0.488026018	-0.808437349	-1.74178261	-1.110915901	-0.569179503
NRCAM	3.92E-06	3.665480427	4.378511623	4.392317423	4.378511623	4.300123725	2.97819563	2.277984747	1.887525271	2.596935142
NRP2	0.00533056	2.48049922	0.799087306	0.250961574	0.565597176	0.963474124	-0.793356776	-0.438307279	-1.926865295	-0.02180437
NRTN	0.00174732	0.188235294	-0.013043037	0.084064265	0.321928095	0.250961574	2.440952198	2.555816155	3.070389328	2.032100843
NRXN1	0.0031547	4.453961456	0.722466024	1.201633861	1.516015147	0.59454855	-1.095419565	-1.785875195	-1.899695094	-0.249822294
NRXN3	0.0255193	3.655913978	-0.641603738	0.622930351	0.411426246	-0.808437349	-2.126580497	-1.746615764	-2.708396442	-1.194294815
NT5DC3	0.041303	2.134646962	0.815575429	-0.013043037	-0.024736678	0.565597176	0.056583528	-0.701341684	-2.988504361	-0.60823228
NTF4	0.00325273	0.220852018	0.40053793	0.150559677	1.448900951	1.438292852	2.684818738	3.336283388	3.632268215	2.771885579
NTHL1	0.00844724	0.352985075	2.17951105	2.207892852	2.316145742	2.257010618	3.711494907	3.432959407	4.314696526	3.314696526
NTM	0.0373421	5.787671233	0.704871964	1.669026766	-0.126580497	0.042644337	-2.017417053	-1.321928095	-2.652901329	-1.473931188
NTN1	0.00211919	4.113029827	1.819668183	1.438292852	1.007195501	1.150559677	0.124328135	-0.418889825	-2.329159664	-0.954557029
NTRK2	0.0228006	3.545966229	-2.805912948	-1.675765438	-3.023269779	-2.498178735	-3.871970611	-3.721658341	-5.519528055	-4.395928676
NTRK3	0.0289596	3.708609272	-1.586405918	-1.234465254	-2	-3.158429363	-2.698997744	-4.13289427	-5.143054137	-3.990796173
NUF2	0.0252255	0.014227799	-4.359934417	-3.435963338	-3.685941591	-3.719756304	1.454175893	1.077242999	3.253989266	2.66448284
NUP210L	0.0113729	2.694444444	-3.834441746	-3.459575895	-4.181803871	-4.173970214	-6.559791925	-5.380821784	-5.853084152	-4.359934417
NUPR1	0.0116606	0.05203125	-4.988504361	-4.816037165	-5.030324537	-4.816037165	-0.813499442	-0.974262439	0.176322773	-1.502259911
NUSAP1	0.0164967	0.019159664	-0.123433941	0.565597176	0.201633861	-0.017417053	5.318316841	4.350497247	6.601399391	6.334496768
NXPH4	0.0398674	0.439189189	0.722466024	0.815575429	-0.15521265	-0.140825544	1.411426246	2.056583528	1.835924074	0.545968369
NYAP1	0.0476817	0.237086093	-4.925768606	-5.073393259	-4.590744853	-4.673002535	-2.582079992	-1.857259828	-3.795859283	-3.478748205
OAF	0.00694467	0.422492401	-2.680382066	-2.152003093	-3.210896782	-3.925768606	-1.888968688	-1.442222329	-1.314732593	-1.846843212
OCIAD1-AS1	0.0228255	2.542372881	-1.373327247	-1.043943348	-0.231074664	-0.564904848	-2.698997744	-1.932361283	-2.910501849	-1.336427665
OIP5	0.0308025	0.046641221	-4.496549491	-4.756330919	-4.395928676	-3.10780329	-0.351074441	-1.286304185	1.250961574	0.722466024
OLFM5P	0.0157485	3.273322422	-2.224317298	-1.74178261	-2.564904848	-3.083141235	-4.402016006	-3.457989644	-5.573466862	-3.554697058

OLFML3	0.0242058	0.379190751	-0.685013515	-0.352915787	-0.836501268	-0.60164963	0.411426246	0.263034406	1.464668267	0.695993813
OLR1	0.0110135	4.489795918	-3.023269779	-2.011587974	-2.816037165	-3.265344567	-5.173970214	-5.820106829	-4.748553568	-4.194955239
OPCML	0.046482	2.062972292	-3.803896602	-2.988504361	-4.221623189	-3.706511798	-4.760235373	-5.526161147	-4.284897364	-4.354021725
OPRL1	0.0362114	0.482608696	0.070389328	0.650764559	-0.420819852	0.111031312	1.516015147	1.627606838	0.918386234	0.443606651
ORC1	0.0205087	0.180778032	-2.727379545	-2.258425153	-2.329159664	-3.775959726	-0.831357964	-1.095419565	0.014355293	0.545968369
ORC6	0.00294676	0.292105263	0.298658316	0.250961574	0.056583528	-0.035046947	1.996388746	1.5360529	1.59454855	2.403267722
OSBPL3	0.00189137	3.184713376	4.412781525	4.678071905	4.263034406	3.817623258	2.608809243	3.102658131	1.827819025	2.784503983
OTOG	0.0189457	0.449519231	-5.895394957	-5.435963338	-6.868173488	-5.24879339	-4.345197874	-4.457989644	-5.40506933	-4.380821784
OTOGL	0.00146757	3.493975904	1.280956314	1.584962501	1.117695043	0.739848103	-0.440263476	-0.694321257	-1.354759487	-0.134477041
OVAAL	0.0182663	2.297520661	-2.184424571	-1.883635243	-1.261880711	-2.293358943	-2.954557029	-3.158429363	-2.910501849	-3.210896782
P2RX6	0.00306541	0.374545455	0.097610797	0.111031312	0.042644337	-0.114035243	0.879705766	1.726831217	1.735522177	1.333423734
PALM3	0.0453154	0.325748503	-4.374823043	-3.956795501	-4.392894616	-4.117787378	-3.462753639	-1.795859283	-2.465938398	-3.184424571
PANX2	0.0347816	0.433333333	-1.675765438	-1.089267338	-1.502259911	-1.23786383	-0.351074441	-0.857259828	0.50589093	-0.244685096
PAPSS2	0.00660925	3.513888889	-0.380821784	0.150559677	-0.698997744	-0.883635243	-2.61705613	-2.279283757	-1.535331733	-2.756330919
PAQR4	0.0224213	0.356846473	1.427606173	1.659924558	1.344828497	0.941106311	2.114367025	3.112700133	3.432959407	2.370164281
PAQR8	0.000864805	0.409259259	-1.01449957	-1.569179503	-1.279283757	-0.929610672	0.23878686	0.176322773	-0.329159664	0.250961574
PARPBP	0.0181509	0.096508728	-1.473931188	-0.422752464	-2.005782353	-2.365871442	1.843983844	0.014355293	2.477677328	2.550900665
PASD1	0.0493734	2.531293463	-5.339345148	-6.759453637	-6.200249538	-5.299027693	-7.121800441	-7.548944543	-8.133907043	-6.299027693
PASK	0.0144929	0.339224138	-0.334607229	-0.10159814	-0.284545873	-0.729770093	0.925999419	1.263034406	0.5360529	1.815575429
PBK	0.00680232	0.007070254	-5.411195433	-4.844768884	-3.210896782	-5.429731384	2.378511623	1.464668267	3.214124805	3.252476214
PCDH10	0.031285	2.162162162	-1.675765438	-1.921390165	-2.671163536	-2.13289427	-2.531156057	-2.932361283	-5.680382066	-3.058893689
PCGF5	0.000344557	2.018927445	6.147713722	6.053111336	6.0725346	5.683696454	5.057450272	5.048759312	4.986410935	4.842978832
PCNX2	0.00516355	2.330120482	-0.274040765	-0.382701517	0.263034406	0.097610797	-1.446148032	-0.957355663	-2.204233052	-0.831357964
PCSK4	0.0019015	0.328018223	0.516015147	0.773996325	0.5360529	0.214124805	2.107687869	2.301587647	2.438292852	1.526068812
PCSK5	0.0127866	2.695924765	-0.244685096	0.214124805	-0.160040413	-0.883635243	-1.564904848	-0.828793173	-2.514573173	-2.321928095
PCYT2	0.0111423	0.442273535	4.906890596	5.053111336	4.137503524	4.224966365	6.01346226	5.942514505	6.040015679	5.053111336
PDE11A	0.00256323	5.588235294	-2.943416472	-2.190997225	-2.680382066	-3.279283757	-5.030324537	-6.254289378	-4.961282892	-4.930160375
PDE1B	0.000446423	0.270440252	-3.23786383	-2.19759996	-3.293358943	-3.470728756	-0.823677227	-0.929610672	-1.286304185	-1.2968993
PDE1C	0.0088039	0.31292517	2	1.176322773	2.201633861	2.912649865	4.053111336	3.523561956	3.336283388	4.357552005
PDE3A	0.00199283	2.392857143	-1.340075442	-1.780908942	-1.49410907	-1.74178261	-2.418889825	-2.59946207	-4.112786697	-2.746615764
PDE4B	0.00384409	2.502628812	4.847996907	4.478971805	4.504620392	4.432959407	3.837943242	1.646162657	2.835924074	3.754887502
PDLIM3	0.000125577	4.160305344	6.807354922	6.845490051	6.894817763	6.477353527	3.916476644	5.51412226	4.378511623	4.5360529
PEG10	0.0100603	0.340909091	0.201633861	-0.219269964	-0.010134377	0.250961574	1.748461233	0.765534746	1.550900665	2.111031312
PFKFB2	0.00484954	2.343949045	5.402585758	4.986410935	5.193771743	5.197708158	4.666756592	3.678071905	2.384049807	4.292781749
PGF	0.0203808	0.451669596	-1.461958547	-1.926865295	-2.158429363	-2.490050854	-0.275786313	-1.200912694	-0.800877358	-1.184424571
PGP	0.00413723	0.475644699	0.82374936	0.831877241	0.659924558	0.565597176	1.879705766	1.659924558	2.169925001	1.384049807
PHACTR3	0.0133409	0.457746479	-2.329159664	-1.434402824	-0.639354798	-0.708396442	-0.144010303	-0.340075442	0.40053793	-0.058893689
PHEX	0.0120937	5.017241379	-4.085588556	-5.13289427	-3.451662024	-4.202903992	-6.773590119	-6.848089242	-6.651087759	-5.733123528
PHF19	0.000861112	0.199264706	1.333423734	1.263034406	1.59454855	1.526068812	3.689299161	3.139142019	4.03562391	4
PHGDH	0.00344725	0.162433862	1.735522177	1.948600847	1.422233001	1.275007047	4	4.285402219	4.802193217	3.596935142
PHLDA1	0.00150513	4.792	2.893362211	2.853995647	2.419538892	1.981852653	0.765534746	-0.756330919	0.545968369	0.321928095
PIANP	0.00199843	3.608040201	-1.184424571	-1.204233052	-1.708396442	-1.971430848	-3.279283757	-2.736965594	-3.787866492	-3.775959726
PIP4K2A	0.0024948	2.013404826	2.924099886	3.184280294	2.972692654	2.462052319	1.757023247	2.010779839	1.835924074	1.98550043

PITPNM3	0.0320804	2.078431373	-1.418889825	-1.899695094	-0.854648614	-1.002888279	-2.19759996	-2.035046947	-3.838563734	-1.821126042
PKN3	6.81E-05	0.347465438	-0.471928835	-0.147202107	-0.426625474	-0.623709617	1.137503524	1.298658316	1.150559677	0.847996907
PKP4-AS1	0.0214532	2.510067114	-4.40506933	-5.448508591	-4.760235373	-4.54962012	-5.912672948	-6.559791925	-7.79906884	-5.158429363
PLA2G4C-AS1	0.00261465	2.043859649	-1.083141235	-1.035046947	-0.844250767	-1.531156057	-2.343732465	-1.831357964	-2.351074441	-2.083141235
PLAU	0.0105023	2.396606575	1.378511623	1.422233001	1.176322773	0.59454855	-0.592919225	-0.352915787	0.739848103	-0.612637459
PLAUR	0.00386362	0.236882129	-1.227692025	-0.388355457	-0.632628934	-0.612637459	0.678071905	1.275007047	1.883620816	1.485426827
PLB1	0.0118337	2.068010076	-0.77102743	0.137503524	-0.415037499	-0.231074664	-1.560642822	-0.857259828	-1.573466862	-1.477944251
PLCB1	0.0102912	2.126984127	-1.805912948	-2.10780329	-1.751465164	-1.954557029	-3.070966521	-4.0185857	-3.355497628	-2.139235797
PLCD3	0.0116939	0.495364238	1.948600847	1.843983844	1.748461233	2.056583528	3.316145742	3.056583528	2.333423734	2.776103988
PLCG2	0.00056666	0.317431193	-3	-3.058893689	-2.442222329	-1.932361283	-1.017417053	-1.064917477	-0.913216234	-0.562772261
PLCXD1	3.43E-06	0.25	-0.392137097	-0.10780329	-0.067938829	-0.092340172	1.731183242	1.773996325	1.831877241	2.014355293
PLD5	0.0058473	2.809917355	0.176322773	-0.40354186	0.422233001	-0.227692025	-0.717856771	-2.058893689	-1.841662973	-1.625934282
PLEKHA6	0.0106107	2.017316017	5.395748328	5.842978832	5.62058641	5.244125943	4.432959407	5.161887682	3.64385619	4.491853096
PLEKHB1	0.000298543	0.31092437	0.263034406	0.411426246	-0.03948829	-0.084670324	1.811471031	2.003602237	2.017921908	1.432959407
PLEKHH2	0.0283771	6.336633663	-3.023269779	-1.40354186	-1.347398782	-2.921390165	-4.836501268	-4.187050041	-6.148161027	-4.112786697
PLIN2	0.000210265	0.481	2.353323291	1.819668183	2.336283388	2.472487771	3.44625623	3.112700133	3.392317423	3.336283388
PLK1	0.00181638	0.067261905	-4.179187923	-2.582079992	-2.977099598	-3.293358943	0.879705766	0	1.257010618	0.555816155
PLK2	0.00032082	3.455210238	4.485426827	4.392317423	4.087462841	3.944858446	2.983677695	2.229587923	2.12763328	2.301587647
PLK3	0.00121863	4.977029096	1.82374936	2.042644337	1.673556424	1.117695043	-0.968604804	0.298658316	-0.897006007	-1.569179503
PLK4	0.0371804	0.0544	-3.532824877	-4.28208783	-3.334971132	-3.481968507	0.40053793	-0.77349147	0.097610797	1.604071324
PLLP	0.000880732	0.278217822	-4.725469955	-6.546245393	-4.752437003	-5.227016448	-3.395928676	-3.046921047	-3.145605322	-3.735043282
PLP2	0.0313668	0.227578475	0.731183242	0.545968369	1.189033824	1.454175893	2.778208576	2.531069493	4.017921908	2.767654798
PLPP4	0.0388713	2.914072229	-2.522840789	-1.465938398	-1.76611194	-3.23786383	-3.359934417	-3.636660688	-3.504714171	-4.173970214
PLXDC1	0.0043799	3.541284404	-4.395928676	-3.224317298	-3.631287516	-3.76611194	-5.580353247	-6.030324537	-5.200249538	-5.398969131
PLXNA2	0.0137945	2.435897436	4.061776198	3.925999419	3.321928095	3.485426827	3.196921734	2.687060688	0.432959407	2.286881148
PLXNB3	0.0479984	2.909698997	-2.867752202	-1.76611194	-3	-2.846843212	-4.583808806	-2.965784285	-4.882570916	-4.787866492
PMAIP1	0.000727894	5.775749674	-0.836501268	-1.265344567	-1.043943348	-1.689659879	-4.529489165	-4.345197874	-2.805912948	-3.818070562
PMEL	0.0473483	0.300925926	2.250961574	2.046141782	0.933572638	1.137503524	3.817623258	4.09592442	3.03562391	1.959770155
POC1A	0.0185833	0.172161172	-0.043943348	1.09085343	0.757023247	-0.248107862	2.516015147	2.111031312	3.786596362	3.165107985
PODN	0.0033413	2.049056604	2.22650853	2.424922088	2.59454855	2.493134922	1.028569152	1.014355293	2.066950244	1.232660757
POLA2	0.00896729	0.292469352	0.782408565	0.632268215	0.687060688	0.831877241	2.344828497	1.682573297	2.653060017	3.049630768
POLD1	4.91E-05	0.305882353	1.599317794	1.82374936	1.691534165	1.416839742	3.288358562	3.165107985	3.596935142	3.336283388
POLE	0.016864	0.273631841	0.150559677	-0.054392297	0.084064265	0.321928095	2.066950244	2.272023189	0.575312331	2.480265122
POLE2	0.0338564	0.077184466	-2.10159814	-1.564904848	-2.177881725	-1.058893689	0.98550043	0.575312331	2.773996325	2.627606838
POLH	0.000180292	2.607407407	4.378511623	4.10433666	4.061776198	3.981852653	3.100977648	2.327687364	2.61117238	2.86393845
POU5F1B	0.00196093	2.619047619	-3.526161147	-2.988504361	-3.171368418	-3.13289427	-5.392894616	-5.210896782	-3.950090478	-4.24879339
POU5F1P3	0.0124699	2.606951872	-3.803896602	-3.481968507	-2.76611194	-3.60823228	-4.832385159	-4.496549491	-4.891107598	-4.775959726
PP2D1	0.00301966	2.522727273	-3.910501849	-3.925768606	-4.615287038	-4.354021725	-5.580353247	-5.930160375	-5.587272661	-5.058893689
PPFIA2	0.00248941	3.554603854	1.344828497	2.147306699	1.859969548	1.422233001	-0.043943348	-0.461958547	-0.708396442	0.516015147
PPM1E	0.014954	2	-2.258425153	-2.19759996	-1.59946207	-2.343732465	-3.554697058	-3.321928095	-2.954557029	-2.61705613
PPM1H	0.0153552	2.537878788	0.790772038	0.687060688	0.028569152	-0.020340448	-0.18606493	-1.148800661	-2.388355457	-0.763660461
PPM1K	0.000828228	2.476851852	5.498250868	5.675251386	5.95419631	5.794415866	4.240314329	3.666756592	4.614709844	4.921245889
PPP1R14A	0.00615912	2.394463668	-0.457989644	-0.246395464	-0.722610301	-0.758769964	-3.070966521	-2.272297327	-1.10159814	-1.457989644

PPP1R1A	0.032446	0.265322581	3.209453366	3.019701914	3.364572432	3.560714954	5.01346226	4.070389328	6	5.153805336
PPP2R2C	0.00769969	3.464285714	-1.590744853	-0.844250767	-1.746615764	-1.446148032	-2.727379545	-2.286304185	-4.988504361	-4.044538396
PRC1	0.00172952	0.053333333	1.195347598	1.269033146	1.66448284	1.871843649	5.62058641	4.935459748	5.914086097	6.247927513
PRC1-AS1	0.00768515	0.076439791	-6.221623189	-5.102837037	-4.027969116	-3.807932116	-1.377069649	-1.481968507	-0.095419565	-0.708396442
PRIM1	0.0108452	0.103571429	-4.171368418	-3.649276466	-3.708396442	-4.187050041	-1.03209363	-1.867752202	-0.084670324	-0.199255376
PRIMA1	0.000962647	0.137847222	-4.526161147	-4.461163892	-4.691522623	-4.997693533	-1.286304185	-1.954557029	-2.077041036	-2.029146346
PRKCB	0.00363713	2.713815789	-2.272297327	-2.411195433	-3.011587974	-2.826232932	-4.374823043	-4.48681248	-4.187050041	-3.389866924
PRKCQ-AS1	0.0192156	0.397883598	-3.184424571	-3.456405136	-4.423526235	-4.246053228	-2.698997744	-2.126580497	-1.971430848	-3.10780329
PRKG2	0.00501146	2.778145695	-0.030619235	-0.066427362	-0.19759996	-0.862496476	-2.13289427	-0.965784285	-2.582079992	-1.722610301
PRKX	8.19E-05	4.382978723	0.201633861	0.201633861	-0.058893689	-0.239566125	-1.717856771	-1.921390165	-3.023269779	-2.011587974
PRKY	0.00580901	3.947750363	-4.583808806	-5.448508591	-5.506352666	-5.48681248	-6.933683441	-7.388053353	-7.40996813	-7.056971377
PROCA1	0.0443469	0.387559809	0.807354922	1.50589093	1.560714954	1.117695043	1.422233001	3.032100843	3.200064862	2.3305584
PROCR	0.00686694	2.429906542	-2.836501268	-3.158429363	-2.634867407	-3.265344567	-5.480357457	-4.210896782	-3.494922085	-4.348133165
PRODH	0.000625625	2.703125	2.316145742	2.625270489	2.462052319	2.03562391	0.782408565	1.304511042	1.111031312	0.422233001
PROM1	0.00456201	2.842105263	2.680324357	3.124328135	2.403267722	2.462052319	-0.084670324	1.847996907	1.244887059	1.137503524
PRR11	0.00650999	0.169026549	-2.434402824	-2.465938398	-2.351074441	-2.300448367	0.432959407	-1.023269779	0.176322773	0.641546029
PRR5	0.0233355	0.488235294	2.430285273	2.462052319	2.070389328	2.269033146	3.272023189	3.392317423	3.847996907	2.627606838
PRRT4	0.00289521	0.204761905	-1.190997225	-1.224317298	-1.625934282	-0.924125133	0.526068812	1.560714954	1.232660757	0.731183242
PRSS12	0.00249506	6.768060837	-2.365871442	-2.005782353	-2.64385619	-3.210896782	-5.930160375	-4.519528055	-5.189680297	-5.811978949
PRSS27	0.0112237	0.3525	-0.862496476	-0.729770093	-0.698997744	-1.040971781	0.378511623	0.495695163	1.292781749	0.321928095
PRSS3	0.0297252	3.606102635	-2.114035243	-1.181149439	-2.76611194	-2.184424571	-3.327710447	-6.232429944	-3.41734766	-3.611755347
PRSS8	0.0208022	0.363636364	-3.684086035	-4.40812913	-2.652901329	-2.126580497	-1.932361283	-1.960159735	-0.98279071	-1.388355457
PSAT1	0.036173	0.491596639	0.495695163	0.790772038	-0.377069649	-0.390245038	1.59454855	1.613531653	0.790772038	0.765534746
PSMB8-AS1	0.00451032	3.088552916	0.367371066	1.03562391	0.411426246	0.070389328	-1.498178735	-0.957355663	-0.883635243	-1.184424571
PSRC1	0.000980273	0.04515873	-0.267079618	-0.407363571	-1.977099598	-1.200912694	3.432959407	3.014355293	4.112700133	3.827819025
PTCHD4	0.00088151	2.75625	2.358958826	1.891419187	2.107687869	2.169925001	1.316145742	-0.189351252	0.286881148	0.86393845
PTGER4	0.0268687	4.779661017	-1.168122759	-1.438307279	-2.775959726	-2.573466862	-4.205563338	-7.158429363	-3.736965594	-3.326262685
PTGER4P2-CDK2AP2P2	0.00135359	2.00204918	-3.411195433	-3.336427665	-3.40354186	-3.279283757	-4.791857353	-4.760235373	-3.79385693	-4.316168826
PTH1R	0.00510155	0.379716981	3.733354341	4.153805336	4.017921908	4.09592442	5.177917792	5.791814071	5.62058641	4.847996907
PTK6	0.0366741	0.314049587	-4.654717182	-6.088040035	-8.078259014	-5.803896602	-5.10780329	-4.41734766	-3.573466862	-3.604717796
PTMS	0.0289607	0.432485323	4.485426827	4.452858965	4.399171094	4.517275693	5.504620392	5.5360529	6.323730338	5.026800059
PTP4A1	0.000864497	2.164251208	5.526694846	5.116863758	5.697662633	5.54225805	4.061776198	4.578938713	4.452858965	4.343407822
PTPN18	0.0038493	0.330246914	-1.351074441	-1.231074664	-0.646112164	-0.550042516	0.028569152	0.90303827	1.03562391	0.613531653
PTPN7	0.0262961	3.945720251	-2.300448367	-1.76121314	-3.578628567	-2.531156057	-4.590744853	-3.293358943	-8.066608654	-4.503077534
PTPRB	0.00199818	4.88372093	0.014355293	0.097610797	-0.382701517	-0.960159735	-2.708396442	-2.358453971	-2.921390165	-2.272297327
PTRHD1	0.0419435	0.442663379	2.107687869	1.748461233	1.761285273	1.726831217	2.895302621	2.336283388	3.711494907	2.776103988
PTTG1	0.0216513	0.063271605	1.117695043	1.3950628	1.117695043	0.321928095	4.791814071	3.700439718	5.902073579	4.862947248
PURPL	5.81E-05	5.113636364	3.498250868	3.584962501	3.972692654	3.887525271	0.948600847	1.570462931	1.459431619	1.545968369
PUSL1	0.033324	0.479807692	2.201633861	2.657640005	2.289834465	2.060047384	3.336283388	3.160274831	3.981852653	2.776103988
PVT1	0.00260189	2.319018405	-1.438307279	-1.029146346	-1.662003536	-1.569179503	-2.54793177	-2.717856771	-2.244685096	-3.10780329
PXDNL	0.00896566	2.428571429	-1.117161344	-0.652901329	-1.158429363	-1.785875195	-2.411195433	-2.351074441	-2.54793177	-2.321928095
PXMP2	0.0322983	0.376528117	0.799087306	0.389566812	0.584962501	0.687060688	1.735522177	1.521050737	2.767654798	1.735522177
PYGL	0.0070901	2.41416309	1.448900951	1.49057013	0.963474124	0.584962501	-0.40354186	-0.192645078	0.250961574	-0.144010303

PYGM	0.00240448	0.240769231	1.389566812	1.327687364	1.794935663	1.974529312	3.584962501	3.472487771	4.217230716	3.350497247
QDPR	0.0143167	0.49201278	2.070389328	2.097610797	2.378511623	2.263034406	2.914564523	2.87774425	3.733354341	3.228049048
QPCT	0.00200373	3.605691057	-3.324816374	-3.210896782	-3.712173133	-3.826232932	-6.078259014	-5.895394957	-4.333516069	-5.853084152
RAB17	0.0373484	3.517241379	1.618238656	2.803227036	2.03562391	1.117695043	-0.475936324	1.097610797	-0.40927823	0.056583528
RAB26	0.00135887	0.333333333	-3.293358943	-2.411195433	-2.210896782	-2.152003093	-1.384583703	-0.698997744	-0.857259828	-0.63039393
RAB39B	0.0108776	2.357414449	-2.899695094	-2.522840789	-3.265344567	-3.573466862	-4.304718805	-4.265344567	-4.31329779	-4.115284871
RAB44	0.0182373	6.917562724	-3.35254733	-1.746615764	-1.943416472	-3.120294234	-6.532824877	-5.473931188	-5.10780329	-4.339345148
RABL2A	0.00404847	2.013363029	6.510961919	6.807354922	6.357552005	6.257387843	5.725195817	5.773468928	4.727920455	5.50779464
RABL2B	0.00662423	2.114583333	5.361066489	5.635173947	5.339850003	4.963474124	4.54225805	4.694880193	3.204766751	4.217230716
RAC3	0.0274141	0.279132791	0.895302621	0.970853654	1.15704371	1.13093087	2.438292852	2.464668267	3.689299161	2.513490746
RACGAP1	0.00560653	0.08401487	1.321928095	1.304511042	1.15704371	0.871843649	4.578938713	3.572889668	5.03562391	5.277984747
RAD51AP1	0.0322573	0.018455285	-5.102837037	-4.865647613	-5.904008087	-6.532824877	-0.55851652	-1.080087911	0.443606651	1.263034406
RAD51AP2	0.0167235	2.277992278	-3.145605322	-3.859352207	-2.756330919	-2.795859283	-3.714065192	-4.580353247	-4.768076127	-4.251538767
RANGAP1	0.000633104	0.481675393	4.672425342	4.733354341	4.902073579	4.837943242	5.689299161	5.995484519	6.046578367	5.581953751
RAP1GAP	0.00139324	0.280503145	1.799087306	2.538538164	2.319039816	1.831877241	3.711494907	4.40599236	4.061776198	3.666756592
RAP2B	0.000228225	3.306666667	-0.184424571	-0.270555993	-0.634867407	-0.680382066	-1.960159735	-2.321928095	-2.307572802	-2.052894948
RAPGEF3	0.00504648	0.431005111	-2.064917477	-1.977099598	-2.336427665	-1.648371671	-1.061902439	-0.524915117	-0.454031631	-1.174621396
RAPGEF5	0.0153657	10.69444444	-1.139235797	-0.650634722	-1.76121314	-2.689659879	-4.208227596	-4.321928095	-6.179187923	-5.259806383
RASAL2-AS1	3.28E-05	2.341040462	-1.261880711	-1.293358943	-1.200912694	-1.473931188	-2.662003536	-2.321928095	-2.826232932	-2.388355457
RASD2	0.000287186	2.184466019	1.263034406	1.15704371	1.22650853	1.014355293	0.056583528	-0.50021788	0.464668267	0.014355293
RASEF	0.013352	2.379807692	-4.299027693	-3.988504361	-4.40812913	-4.760235373	-6.13796526	-5.345197874	-6.958588783	-4.775959726
RASGRF1	0.0106887	5.535168196	-3.874084451	-3.691522623	-5.184424571	-4.49980982	-5.622376462	-8.834853415	-8.495898308	-6.158429363
RASGRP2	0.0247911	0.375714286	2.5360529	2.648465443	3.184280294	3.378511623	4.044394119	3.972692654	5.074676686	4.169925001
RASL11B	0.0047534	2.208333333	2.5360529	2.310340121	2.140778656	1.970853654	0.650764559	1.799087306	0.86393845	0.831877241
RBFOX3	0.0135886	5.694444444	-2.746615764	-1.481968507	-1.194294815	-2.300448367	-4.559791925	-4.324816374	-5.573466862	-3.49980982
RBL1	0.0380349	0.43255814	-0.612637459	-0.813499442	-0.680382066	-1.380821784	0.23878686	-0.448114897	0.286881148	1.021479727
RCAN1	0.00285726	2.288961039	6.249824549	6.371558863	6.081083929	5.789207575	4.791814071	5.560714954	4.426264755	4.744161096
RCOR2	0.000601049	0.375518672	0.748461233	0.622930351	0.879705766	1.117695043	2.558267634	2.084064265	2.370164281	1.992768431
RDH10	0.00082892	2.089093702	0.464668267	0.565597176	0.475084883	0.23878686	-0.780908942	-0.15521265	-1.174621396	-0.552156356
RECQL4	0.00439777	0.238383838	0.933572638	1.722466024	1.137503524	1.028569152	2.752748591	3.10433666	3.867896464	3.279471296
REEP4	0.0320107	0.334500876	0.933572638	1.327687364	0.650764559	0.704871964	2.14404637	2.327687364	3.275007047	1.891419187
REEP6	0.0433032	0.26544021	1.084064265	0.86393845	1.310340121	0.722466024	2.797012978	2.475084883	3.797012978	2.010779839
RENBP	0.0130051	0.41689008	1.700439718	1.63691458	1.580145484	1.627606838	2.641546029	3.22342255	3.279471296	2.182692298
RERG	0.0375301	2.113744076	-0.058893689	0.378511623	-0.552156356	-0.687334826	-1.395928676	-0.910501849	-2.293358943	-0.808437349
RETSAT	0.000828019	2.161538462	1.411426246	1.722466024	1.361768359	1.427606173	0.622930351	0.604071324	-0.349235441	0.443606651
RFC4	0.0165023	0.33970276	0.799087306	0.650764559	0.584962501	0.669026766	1.669026766	1.731183242	2.845991771	2.361768359
RFPL2	0.0321644	0.383891213	-4.262572817	-3.746615764	-7.64385619	-6.030324537	-4.148161027	-3.330610338	-3.058893689	-3.224317298
RFTN1	0.00413326	3.668571429	6.339850003	6.330916878	5.675251386	5.462706751	4.201633861	4.781359714	3.257010618	3.857980995
RFX3	0.00108095	2.651376147	1.550900665	1.321928095	1.613531653	1.622930351	0.35614381	-0.378944497	-0.865121946	0.831877241
RGMA	0.0372265	9.553571429	0.454175893	0.963474124	-1.13606155	-0.954557029	-3.171368418	-2.465938398	-3.389866924	-4.097887821
RGS14	0.00194472	0.4473342	-1.648371671	-1.145605322	-2.244685096	-1.347398782	-0.321928095	-0.199255376	-0.295128036	-0.768567592
RGS20	0.018427	2.636363636	-0.894321922	-0.490050854	-0.673462652	-1.60823228	-4.03268381	-1.36215794	-2.473931188	-2.286304185
RHOF	0.00200387	0.470790378	0.495695163	0.310340121	0.464668267	0.5360529	1.89917563	1.448900951	1.516015147	1.22650853

RIN1	0.0435441	0.478142077	-3.332062472	-3.332062472	-3.865647613	-3.594225422	-3.251538767	-1.873027144	-2.265344567	-2.76611194
RMI2	0.00892264	0.129537367	-1.49410907	-1.713118852	-1.54793177	-1.13606155	0.799087306	0.799087306	2.107687869	1.786596362
RNASEH2A	0.0427136	0.170901639	-0.286304185	0.014355293	-0.392137097	-0.434402824	1.608809243	1.416839742	3.246408087	2.100977648
RNF144B	0.00397118	2.65408805	0.097610797	-0.042456799	-0.703689439	-0.4639471	-1.321928095	-2.506352666	-1.556393349	-1.481968507
RNF165	0.00260034	0.340949033	-2.23786383	-2.481968507	-2.717856771	-2.10159814	-0.483984853	-0.878321443	-1.418889825	-0.639354798
RNF207	0.012382	0.420305677	4.666756592	5.022367813	5.539158811	5.635173947	6.402585758	6.988684687	5.899659026	6.571373436
ROPN1L	0.000773169	2.287234043	-1.336427665	-1.080087911	-1.017417053	-1.486004021	-2.76611194	-2.052894948	-2.307572802	-2.64385619
RPARP-AS1	0.0474949	0.3528	-1.988504361	-0.849440323	-0.675765438	-1.595096878	-0.564904848	0.263034406	1.097610797	0
RPL23AP53	0.000259514	2.535971223	2.720278465	2.782408565	2.90303827	2.855989697	1.427606173	1.929790998	0.454175893	1.691534165
RPL23AP7	6.90E-05	2.240740741	3.44625623	3.042644337	3.307428525	3.282439805	2.204766751	2.107687869	2.007195501	2.111031312
RPL23AP82	4.86E-05	2.365325077	3.049630768	2.726831217	2.952333566	2.987320866	1.839959587	1.713695815	1.35614381	1.807354922
RPL23AP87	0.000242974	2.034623218	0.137503524	-0.205896101	0.028569152	0.014355293	-0.798366139	-1.080087911	-1.426625474	-0.878321443
RPS27L	0.00878662	2.300518135	5.590961241	4.94016675	5.672425342	5.575917361	4.169925001	3.229587923	5	4.145677455
RRM1	0.02316	0.392666667	2.625270489	2.462052319	2.46727948	2.66448284	3.472487771	3.177917792	4.153805336	4.459431619
RRM2	0.00614278	0.037279597	-2.943416472	-2.717856771	-2.59946207	-2.795859283	1.526068812	0.948600847	2.503348735	2.438292852
RRM2B	0.000123202	3.944636678	3.632268215	3.187451054	3.666756592	3.523561956	1.718087584	1.117695043	0.879705766	2.084064265
RSPH14	0.0263807	0.409893993	-3.13289427	-3.636660688	-2.556393349	-3.337885669	-2.358453971	-2.418889825	-1.336427665	-1.498178735
RTKN2	0.0131966	0.239814815	-1.76611194	-1.921390165	-1.873027144	-2.272297327	-0.134477041	-1.020340448	0.50589093	0.584962501
RTL9	0.00254018	0.268092105	-2.708396442	-3.797864419	-2.336427665	-2.120294234	-0.418889825	-0.751465164	-1.395928676	-0.49410907
RUNX1	0.0493644	2.375	-1.164884385	-1.63039393	-1.426625474	-2.708396442	-3.011587974	-1.926865295	-3.376320392	-4.023269779
RXRG	0.000451901	2.351190476	1.981852653	2.077242999	2.087462841	1.765534746	0.948600847	0.124328135	1.189033824	0.50589093
RYR3	0.00117655	3.416030534	-0.614845103	0.014355293	0.124328135	-0.281035664	-2.279283757	-1.948975997	-2.434402824	-1.325539348
S100A2	0.0371298	0.259223301	-5.895394957	-6.907467789	-5.695255342	-4	-3.450084446	-4.848920527	-2.857259828	-2.756330919
S100A4	0.0218878	0.206557377	2.526068812	1.176322773	1.90303827	1.744161096	4.277984747	3.350497247	4.95419631	3.655351829
SALL4	0.0456378	2.020725389	-0.182786076	0.50589093	0.411426246	0.042644337	-1.10159814	0.150559677	-2.582079992	-0.798366139
SAPCD2	8.80E-08	0.034947644	-5.13796526	-4.820106829	-5.23786383	-5.912672948	-0.365871442	-0.522840789	-0.304006187	-0.365871442
SATB2	0.00823361	2.646310433	0.042644337	0.367371066	0.214124805	-0.545824107	-0.675765438	-1.873027144	-2.582079992	-0.98279071
SCAANT1	0.0117166	2.294736842	-2.265344567	-1.977099598	-2.177881725	-2.395928676	-3.914847319	-2.411195433	-4.389866924	-3.687799537
SCG5	0.00181824	2.838983051	-1.694321257	-1.214240226	-1.556393349	-1.943416472	-3.293358943	-3.251538767	-2.514573173	-3.477140745
SCGB2B2	0.00686671	2.082644628	-3.662003536	-3.332062472	-3.98165069	-4.039784866	-4.684086035	-5.316168826	-4.74081792	-4.506352666
SCHLAP1	0.0267785	2.047619048	0.641546029	1.097610797	1.09085343	0.070389328	0.22650853	-0.13606155	-1.171368418	-0.265344567
SCN1B	0.00559135	0.346028292	-1.438307279	-1.40354186	-1.921390165	-1.943416472	-0.327348371	-0.029146346	0.344828497	-0.680382066
SCN2A	0.0399793	7.197452229	-3.365871442	-2.184424571	-4.218934102	-3.649276466	-5.965784285	-7.000461736	-6.299027693	-5.24331826
SCN3B	0.00326762	0.292828685	-3.279283757	-4.53951953	-3.846843212	-3.671163536	-1.915935735	-1.531156057	-2.395928676	-2.293358943
SCN4B	1.87E-05	5.183544304	3.258518925	2.891419187	2.952333566	3.005399988	1.056583528	0.855989697	-0.219269964	0.659924558
SCN7A	0.00208414	4.282208589	-1.473931188	-1.384583703	-1.184424571	-2.244685096	-4.304718805	-3.613520111	-3.963531833	-2.954557029
SDC1	0.00169147	3.626262626	2.014355293	2.217230716	1.608809243	1.389566812	-0.533242384	-0.304006187	0.275007047	0.321928095
SDC3	0.0401662	2.093385214	6.070389328	6.085339669	5.339850003	5.314696526	5.277984747	5.133399125	2.66448284	4.504620392
SDR42E2	0.0487695	0.327586207	-6.601211852	-4.828280761	-7.67006126	-6.573466862	-4.676687582	-4.24331826	-3.79385693	-5.532824877
SEL1L3	0.0241117	2.639791938	1.195347598	1.321928095	0.925999419	0.50589093	-1.311148256	0.847996907	-1.506352666	-0.943416472
SELENBP1	0.0145688	0.242753623	0.111031312	0.23878686	0.555816155	0.704871964	1.859969548	2.025028794	3.173127433	2.416839742
SEMA3D	0.00690757	2.926229508	-1.104697379	-1.535331733	-1.450084446	-1.977099598	-2.634867407	-4.333516069	-3.990796173	-2.224317298
SEMA4A	0.0246916	2.031914894	1.339137385	2.163498732	2.263034406	1.799087306	-0.289827252	1.169925001	1.427606173	0.807354922

SEPT4	0.0147441	2.98013245	4.185866545	4.754887502	3.857980995	3.632268215	1.811471031	2.3305584	3.350497247	2.443606651
SERPINE2	0.00552022	2.496183206	1.773996325	2.153805336	1.475084883	1.275007047	0.713695815	0.028569152	0.35614381	0.35614381
SESN1	1.03E-05	2.739018088	4.491853096	4.224966365	4.478971805	4.426264755	2.887525271	3.152183419	2.726831217	3.007195501
SESN2	0.00517476	2.526143791	3.168321116	3.250961574	2.704871964	2.560714954	1.952333566	2.13093087	1.03562391	0.956056652
SGCD	0.00230288	2.504504505	-0.524915117	-0.937878288	-0.943416472	-1.035046947	-2.522840789	-1.603840511	-3.046921047	-1.915935735
SGIP1	0.00408214	3.875	0.028569152	0.214124805	0.214124805	-0.977099598	-2.13289427	-2.481968507	-2.058893689	-1.53951953
SGK1	0.00385626	4.245742092	2.189033824	2.13093087	1.464668267	1.163498732	-0.02620507	-0.304006187	-0.639354798	-0.229382353
SGO1	0.00824698	0.073817035	-1.960159735	-1.415037499	-2.795859283	-2.625934282	1.847996907	0.189033824	1.66448284	2.253989266
SGO2	0.0147954	0.100645161	-1.336427665	-1.457989644	-2.139235797	-1.937878288	1.220329955	0.23878686	2.250961574	2.042644337
SGSM1	0.0226168	0.22360515	-4.120294234	-3.828280761	-4.506352666	-4.775959726	-1.434402824	-2.114035243	-3.597714408	-2.011587974
SH3BP1	0.00145467	2.079310345	-0.516635639	-0.526992432	-0.913216234	-1.029146346	-1.921390165	-1.577766999	-1.862496476	-1.810966176
SH3BP2	0.0103618	0.473537604	0.565597176	0.613531653	0.918386234	0.925999419	2.192194165	1.827819025	1.150559677	2.003602237
SH3GL2	0.00106379	2.009049774	-1.043943348	-1.388355457	-0.968604804	-1.321928095	-2.300448367	-2.556393349	-1.971430848	-1.960159735
SHCBP1	0.00216448	0.05371179	-3.893249685	-2.61705613	-2.522840789	-3.551310448	1.028569152	0.378511623	1.310340121	1.731183242
SHD	0.0148769	0.472864322	3.08236197	3.218781168	3.364572432	3.258518925	4.193771743	4.169925001	4.852997588	3.867896464
SHISA7	0.0061744	3.017077799	-6.356975042	-5.733123528	-5.869859865	-6.020925839	-8.480357457	-7.708773666	-8.951428992	-6.442222329
SHMT1	0.000285294	0.304979253	0.765534746	0.650764559	0.650764559	0.084064265	1.875780063	2.280956314	2.508428653	2.341985747
SHQ1	0.00312355	2.574412533	3.678071905	3.321928095	3.032100843	3.0721058	2.277984747	1.82374936	1.03562391	2.289834465
SHROOM4	0.00585201	2.543269231	-0.968604804	-1.415037499	-0.816037165	-0.595096878	-1.746615764	-3.120294234	-2.878321443	-1.821126042
SIGIRR	0.0429185	0.325072886	0.641546029	1.014355293	1.182692298	1.618238656	2.124328135	2.538538164	3.596935142	2.361768359
SIGLEC10	0.0041695	0.291666667	-3.702749879	-3.145605322	-5.143054137	-4.011587974	-2.59946207	-1.831357964	-2.293358943	-1.685013515
SIPA1	0.0100892	0.467592593	-1.092340172	-0.49410907	-1.13606155	-1.373327247	-0.392137097	0.378511623	0.389566812	-0.10159814
SKA1	0.013903	0.156603774	-2.590744853	-2.481968507	-2.395928676	-2.954557029	-0.434402824	-0.997117491	0.526068812	0.650764559
SLC11A1	0.00125231	0.360824742	-4.090495686	-1.831357964	-2.329159664	-2.625934282	-1.070966521	-0.946193556	-0.897006007	-1.293358943
SLC16A3	0.00918785	0.245283019	2.622930351	2.475084883	2.673556424	2.9800253	4.459431619	5.332707934	4.812498225	3.963474124
SLC1A1	0.0385915	0.482035928	-2.680382066	-2.785875195	-2.867752202	-2.272297327	-1.652901329	-2.64385619	-1.200912694	-1.23786383
SLC22A18	0.0329931	0.290869565	2.469885976	3.09592442	2.625270489	2.698218478	4.10433666	4.608809243	5.277984747	3.5360529
SLC24A3	0.00141414	2.930555556	-1.648371671	-1.457989644	-1.10159814	-0.888968688	-2.846843212	-2.727379545	-2.816037165	-2.795859283
SLC25A10	0.0208406	0.347174164	1.521050737	1.726831217	1.367371066	1.704871964	2.86393845	2.778208576	3.817623258	2.689299161
SLC25A30-AS1	0.0214015	2.144981413	-4.100360306	-3.853084152	-4.640253953	-3.979373349	-4.371833001	-6.189680297	-5.844768884	-5.148161027
SLC26A7	0.0336242	2.61440678	-4.054092703	-4.445362036	-3.554697058	-4.163591068	-6.699747391	-8.121800441	-4.235144329	-5.158429363
SLC26A9	0.000887323	3.773584906	1.070389328	1.310340121	0.98550043	0.526068812	-0.330973234	-1.117161344	-1.816037165	-0.783389931
SLC27A3	0.0192056	0.411695906	-0.935117148	-0.4639471	-0.392137097	-0.312939312	0.124328135	1.201633861	1.124328135	0.344828497
SLC2A13	0.0093198	2.360570687	1.298658316	0.86393845	0.454175893	0.695993813	-0.002888279	-0.899695094	-1.058893689	0.097610797
SLC36A1	0.0134071	2.014705882	3.916476644	3.925999419	3.472487771	3.733354341	3.068670811	3.255500733	0.82374936	2.90303827
SLC37A1	0.0256666	2.089337176	0.704871964	0.887525271	0.464668267	-0.092340172	-0.706041021	0.176322773	-1.595096878	-0.504304837
SLC44A5	0.00244209	2.486928105	2.967168608	2.659924558	3.109360559	2.939226578	1.613531653	0.879705766	1.077242999	2.389566812
SLC45A2	0.00011257	0.222900763	-1.648371671	-1.894321922	-2.662003536	-1.244685096	0.475084883	0.422233001	0.028569152	0.565597176
SLC4A11	0.0419487	4.189602446	0.389566812	1.257010618	0.23878686	-0.715485867	-2.442222329	-0.307572802	-3.359934417	-2.204233052
SLC52A1	0.0256312	0.473180077	-5.506352666	-5.205563338	-8.017183437	-4.445362036	-4.184424571	-4.102837037	-4.227016448	-4.570035956
SLC6A1	0.00865646	3.581081081	0.056583528	1.214124805	0.773996325	0.35614381	-1.960159735	-0.545824107	-1.841662973	-0.852042119
SLC6A2	0.0230616	4.868421053	-1.017417053	-2.272297327	-0.556393349	-0.249822294	-3.970299766	-1.883635243	-4.004624027	-4.095419565
SLC6A6	0.000119747	7.896103896	5.213347282	5.008988783	4.776103988	4.64385619	2.906890596	1.427606173	0.070389328	2.063502942

SLC8A1-AS1	0.00155764	2.953367876	-2.988504361	-3.251538767	-3.570035956	-2.826232932	-5.304718805	-4.702749879	-4.779917739	-4.202903992
SLC9A3R2	0.00379223	0.360509554	2.550900665	2.488000771	2.493134922	2.469885976	3.925999419	4.026800059	4.399171094	3.364572432
SLC9C1	0.00390388	2.761061947	-2.265344567	-2.921390165	-3.070966521	-2.582079992	-4.380821784	-4.020925839	-3.652901329	-4.764150423
SLFN5	0.00233036	2.208860759	-1.399730246	-1.358453971	-1.595096878	-1.756330919	-2.351074441	-3.293358943	-3.184424571	-2.152003093
SLX4	0.00860762	0.45154185	-1.343732465	-0.795859283	-1.666576266	-1.490050854	0.028569152	0.201633861	-0.724992953	-0.210896782
SMC2	0.0243922	0.404651163	3.207892852	2.861955364	3.160274831	3.224966365	4.459431619	3.689299161	4.177917792	5.044394119
SMC4	0.00503118	0.314204545	2.430285273	2.508428653	2.44625623	2.488000771	3.847996907	3.584962501	4.285402219	4.614709844
SMG5	0.000355834	2.060538117	6.387155718	6.469234794	6.700439718	6.518849829	5.794415866	5.432959407	4.981852653	5.584962501
SMIM10	0.0310647	0.41503268	-3.76219157	-3.53951953	-2.204233052	-2.910501849	-2.388355457	-2.070966521	-1.23786383	-1.434402824
SNAP25	0.014574	2.241477273	2.570462931	3.272023189	3.145677455	2.827819025	1.111031312	2.615887074	0.933572638	1.956056652
SNHG26	0.00238043	2.678082192	-3.738890471	-3.974829424	-3.76219157	-3.307572802	-4.908334012	-6.310432456	-4.970299766	-4.684086035
SNORA103	0.0141159	2.303738318	-1.590744853	-0.951763814	-0.612637459	-1.089267338	-2.76611194	-2.164884385	-2.816037165	-1.535331733
SNORD19B	0.0224268	0.47752809	-2.921390165	-2.522840789	-2.307572802	-2.522840789	-1.873027144	-0.951763814	-1.932361283	-1.426625474
SNORD26	0.0207719	2.704626335	0.443606651	0.310340121	0.90303827	0.695993813	-1.826232932	-1.965784285	-1.621488377	0.464668267
SNORD27	0.02243	2.411504425	1.028569152	0.748461233	1.700439718	0.799087306	0.495695163	-0.584241333	-0.979942348	0.042644337
SNORD28	0.0148671	2.104539202	0.555816155	0.739848103	0.454175893	0.695993813	-0.689659879	-0.793356776	-1.621488377	0.464668267
SNORD31	9.41E-05	2.675675676	1.432959407	1.570462931	1.66448284	1.599317794	0.516015147	0.084064265	-0.673462652	0.389566812
SNORD44	0.00499774	2.341772152	0.422233001	-0.10780329	-0.117161344	0.333423734	-0.529072743	-2.083141235	-1.325539348	-0.816037165
SNORD59B	0.00585162	2.201365188	-0.029146346	0.250961574	0.584962501	0.59454855	-0.241270432	-0.965784285	-1.621488377	-0.60164963
SNORD77	0.0374167	2.715083799	-1.514573173	-1.790858602	-0.577766999	-0.655171503	-3.046921047	-3.865647613	-2.522840789	-1.502259911
SNTB1	0.0439313	2.55862069	-4.654717182	-4.229720657	-4.695255342	-5.912672948	-5.559791925	-5.435963338	-6.674475425	-7.973015854
SORCS1	0.00228603	2.438271605	3.09592442	2.881664619	2.790772038	3.12763328	2.003602237	1.5360529	0.214124805	2.289834465
SOX10	0.0214452	4.164179104	-5.293358943	-6.67006126	-4.803896602	-4.618827395	-7.090004222	-7.228097523	-7.888541286	-6.864806637
SOX9	0.00762151	5.081967213	0.713695815	0.704871964	0.014355293	-0.567040593	-2.564904848	-1.038006323	-2.490050854	-2.816037165
SPAG5	0.000881119	0.082258065	-4.171368418	-2.652901329	-3.351074441	-3.409661467	0.201633861	-0.323732592	0.422233001	0.757023247
SPATA17	0.0176417	2.351694915	-3.472329084	-2.775959726	-3.642053947	-2.977099598	-6.030324537	-3.857259828	-4.566613191	-4
SPATA18	1.34E-05	3.323139653	5.221103725	4.995484519	4.95419631	4.921245889	3.596935142	3.10433666	3.160274831	3.266036894
SPATA19	0.000553528	0.338301043	-1.937878288	-2.756330919	-2.395928676	-1.703689439	-0.689659879	-0.646112164	-0.256700472	-0.76611194
SPATA33	0.000964023	0.409356725	-0.734563104	-0.087733372	-0.60164963	-0.736965594	0.765534746	0.641546029	1.084064265	0.5360529
SPC24	0.0329745	0.076759411	-1.380821784	-0.943416472	-1.268816758	-0.844250767	1.895302621	1.049630768	3.498250868	2.889473543
SPIRE2	0.00608072	0.453503185	-0.823677227	-0.241270432	-0.139235797	-0.921390165	0.084064265	0.956056652	0.713695815	0.722466024
SPN	5.24E-05	0.437751004	-3.429731384	-3.307572802	-3.279283757	-2.836501268	-2.13289427	-1.888968688	-2.035046947	-1.98279071
SPOCK3	0.0111028	2.615384615	-2.434402824	-1.960159735	-1.531156057	-1.746615764	-3.35254733	-5.435963338	-3.070966521	-2.514573173
SPRED2	0.0404953	2.028571429	1.906890596	1.589763487	1.3950628	0.98550043	0.941106311	1.14404637	-1.289827252	0.084064265
SPRY1	0.00100699	3.102040816	2.440952198	2.424922088	1.941106311	1.847996907	0.757023247	0.641546029	-0.093879047	0.748461233
SPRY2	0.000485544	2.139303483	3.897240426	3.689299161	3.632268215	3.523561956	2.906890596	2.613531653	2.028569152	2.682573297
SPRY4	0.024146	6.531531532	4.078951341	4.602884409	3.364572432	2.666756592	0.454175893	2.451540833	0.214124805	-0.190997225
SPRY4-IT1	0.000657626	7.030716724	-1.873027144	-2.158429363	-2.64385619	-2.582079992	-5.374823043	-4.386845572	-5.853084152	-5.153286059
SRPK3	0.00445537	0.289124668	-5.461163892	-2.826232932	-3.395928676	-2.490050854	-1.826232932	-1.190997225	-1.040971781	-1.736965594
SRXN1	1.25E-06	0.375630252	-0.139235797	-0.279283757	-0.139235797	-0.095419565	1.286881148	1.333423734	1.250961574	1.124328135
SS18L1	0.0283457	2.047904192	5.361066489	5.529820947	4.760220946	4.478971805	4.350497247	4.263034406	3.378511623	4.070389328
SSC4D	0.0103827	0.334394904	-1.595096878	-1.932361283	-1.522840789	-1.648371671	-0.634867407	0.028569152	0.475084883	-0.486004021
SSX4	0.00133658	4.112903226	0.35614381	1.321928095	1.13093087	1.137503524	-1.775959726	-1.035046947	-0.886299501	-0.595096878

SSX4B	0.00220172	4.290657439	-0.332789088	0.731183242	0.333423734	0.333423734	-2.867752202	-1.926865295	-1.518701058	-1.2968993
SSX5	0.00481242	4.47284345	-2.265344567	-1.158429363	-1.023269779	-0.907792562	-3.979373349	-3.120294234	-3.970299766	-2.910501849
SSX6	0.000996217	4.016806723	-1.625934282	-0.744197163	-0.971430848	-1.055891201	-3.925768606	-3.058893689	-2.910501849	-2.662003536
ST6GAL2	0.00333625	3.33	1.86393845	2.056583528	1.570462931	1.344828497	-0.53951953	0.97819563	-1.347398782	-0.046921047
ST6GALNAC2	0.028799	2.217741935	0.773996325	-0.078563669	-0.209227962	-0.158429363	-1.442222329	-0.775959726	-0.646112164	-1.351074441
ST7-AS1	0.0218356	0.448477752	-1.810966176	-1.473931188	-1.177881725	-1.161653263	-0.59946207	0.344828497	-0.662003536	-0.234465254
STC2	0.0435265	2.109955423	0.887525271	0.757023247	0.23878686	-0.069451881	0.35614381	-1.227692025	-1.139235797	-0.905088353
STIL	0.0352386	0.34741784	-0.584241333	-0.081613766	-0.467932448	-0.678071905	1.049630768	0.575312331	0.454175893	1.843983844
STIM1	0.000628854	2.449541284	3.019701914	3.17951105	2.748461233	3.028569152	2.066950244	1.929790998	0.773996325	1.757023247
STKLD1	0.023144	0.446726573	-5.74081792	-4.319045586	-6.345197874	-4.102837037	-3.986216185	-3.307572802	-3.914847319	-3.61705613
STMND1	0.0375845	0.495625	-3.878321443	-3.546245393	-3.680382066	-3.546245393	-2.531156057	-3.519528055	-2.76611194	-2.10780329
STXBP5-AS1	0.000714747	2.150684932	-4.861447625	-5.04930764	-4.899695094	-5.184424571	-6.345197874	-5.74081792	-6.6627342	-5.820106829
STXBP6	0.0183134	0.424683544	-0.926865295	-0.373327247	-0.673462652	-0.397828209	0.475084883	-0.035046947	0.739848103	1.201633861
SULF2	0.000950822	7.022900763	5.686500527	5.935459748	5.153805336	5.165911939	2.257010618	3.632268215	2.275007047	2.066950244
SULT1A2	0.0208656	0.485054348	1.464668267	1.875780063	2.13422094	1.782408565	2.283921772	2.786596362	3.392317423	2.833902077
SUSD2	0.00631119	0.088744589	-5.153286059	-5.461163892	-7.41488362	-5.310432456	-2.300448367	-2.251538767	-1.411195433	-2.878321443
SUV39H1	0.0116187	0.281818182	-1.800877358	-1.971430848	-1.020340448	-1.145605322	0.22650853	-0.147202107	1.056583528	0.189033824
SV2C	0.000709719	3.445121951	3.754887502	3.711494907	3.19061486	3.267535798	1.859969548	2.169925001	0.82374936	1.678071905
SYCP2L	0.000465904	0.331363636	-8.587272661	-6.794257179	-7.388053353	-6.429731384	-5.629500897	-5.351074441	-5.553002759	-5.49980982
SYK	0.0103646	4.612937434	-4.496549491	-4.171368418	-4.166178862	-5.772012541	-9.208761038	-6.030324537	-6.519528055	-6.673002535
SYN2	0.0322036	2.156424581	-0.988504361	-1.380821784	-1.177881725	-2.224317298	-2.531156057	-1.932361283	-3.846843212	-2.244685096
SYNE1	0.000339625	2.9375	5.87282876	5.593951284	5.646738698	5.638073837	4.161887682	4.700439718	2.733354341	4.321928095
SYNM	0.00100909	2.158878505	4.66106548	4.554588852	4.439623138	4.439623138	3.523561956	3.711494907	2.350497247	3.700439718
SYNPO2L	0.0390992	2.006410256	8.495855027	8.703903573	7.971543554	7.794415866	7.330916878	8.033423002	6.565292521	6.700439718
SYT11	0.000540696	3.773087071	0.799087306	0.669026766	0.084064265	0.40053793	-1.181149439	-1.652901329	-1.703689439	-1.152003093
SYT13	0.00136108	0.411363636	-2.846843212	-3.307572802	-2.473931188	-1.722610301	-1.336427665	-1.126580497	-1.142417045	-1.142417045
SYT14	0.0155101	2.794642857	-2.177881725	-2.029146346	-1.351074441	-1.347398782	-3.457989644	-2.314732593	-5.725469955	-2.878321443
SYT7	0.00396998	0.303664921	3.99095486	4	4.177917792	4.285402219	6.085339669	6.22881869	5.173926932	5.652486495
SYTL1	0.00678698	2.698492462	2.073820233	2.584962501	2.771885579	2.153805336	0.275007047	0.641546029	1.86393845	0.641546029
SYTL5	0.00302776	2.119791667	5.472487771	5.626439137	5.217230716	4.99095486	4.263034406	4.498250868	3.666756592	4.465974465
TAP1	0.00370336	2.81300813	1.910732662	2.229587923	1.40599236	1.464668267	0.189033824	0.475084883	0.097610797	0.40053793
TAPBPL	0.0217189	0.390551181	-0.584241333	-1.040971781	0.739848103	0.201633861	0.50589093	1.799087306	1.475084883	1.321928095
TCF19	0.0068184	0.027430168	-4.522840789	-3.983931631	-4.41734766	-4.546245393	0.432959407	-0.277533976	1.454175893	1.163498732
TCTEX1D4	3.24E-05	19.77941176	-0.606034724	-1.184424571	-0.899695094	-0.946193556	-8.570721485	-6.068543859	-4.522840789	-4.392894616
TEDC1	0.0176322	0.345505618	-0.386468347	0.5360529	0.389566812	0.485426827	1.298658316	1.722466024	2.482848283	1.521050737
TEDC2	0.0270866	0.354248366	-0.899695094	-0.648371671	-0.897006007	-1.12973393	0.321928095	0.014355293	1.339137385	0.432959407
TESMIN	0.0026364	0.271137026	-2.184424571	-2.625934282	-2.231074664	-2.746615764	-0.343732465	-1.373327247	-0.351074441	-0.351074441
TEX14	0.00511238	3.54094579	-1.894321922	-1.13606155	-1.932361283	-2.040971781	-2.988504361	-3.822145975	-3.795859283	-3.684086035
TFPI2	0.00166328	2.307692308	1.622930351	1.584962501	1.361768359	1.111031312	0.042644337	-0.49410907	0.575312331	0.545968369
TGFA	0.0236514	2.042918455	0.310340121	-0.23786383	-0.268816758	-0.17625064	-0.239566125	-1.727379545	-1.586405918	-1.392137097
TGFB1I1	0.0165039	0.321543408	3.485426827	2.983677695	3.523561956	3.246408087	4.94016675	4.921245889	5.557655155	4.008988783
TGFB2	0.00215244	2.664634146	3.472487771	3.266036894	2.893362211	2.769771739	2.025028794	1.704871964	1.124328135	1.843983844
TGFB2-AS1	0.0283964	2.461165049	-1.477944251	-0.696657606	-0.434402824	-1.680382066	-2.089267338	-2.10159814	-2.816037165	-2.231074664

TGFB2-OT1	0.00156071	2.362903226	1.480265122	1.895302621	1.541019153	1.201633861	0.056583528	0.163498732	0.495695163	0.475084883
TGM1	0.0155169	3.164473684	-4.654717182	-4.066125258	-5.039784866	-3.997693533	-6.773590119	-9.175012247	-5.13289427	-5.546245393
THBS1	0.0498598	2.580195258	4.566815154	4.412781525	3.916476644	3.797012978	2.548436625	4.224966365	-0.531156057	1.794935663
THBS4	0.000458122	5.148247978	4.224966365	4.632268215	4.232660757	3.797012978	1.765534746	1.891419187	1.952333566	1.952333566
THEM6	0.00806582	0.444983819	1.608809243	1.464668267	1.176322773	1.555816155	2.784503983	2.257010618	3.053111336	2.250961574
TICAM1	0.0104726	0.292635659	-5.795859283	-5.227016448	-7.185474186	-6.813600866	-4.011587974	-4.148161027	-4	-5.299027693
TICRR	0.0072423	0.169958848	-4.698997744	-5.519528055	-4.307572802	-4.200249538	-2.10780329	-2.59946207	-2.411195433	-1.365871442
TIFA	0.0246898	0.355018587	-2.258425153	-3.083141235	-2.040971781	-2.351074441	-0.846843212	-2.177881725	-0.685013515	-0.411195433
TIGAR	2.59E-06	2.928994083	1.10433666	0.86393845	0.970853654	0.992768431	-0.432454552	-0.744197163	-0.625934282	-0.473931188
TIGD4	0.0300656	2.032622334	-3.10780329	-2.329159664	-2.816037165	-2.380821784	-4.473931188	-4.435963338	-3.42043364	-2.899695094
TIMELESS	0.00774279	0.176666667	-1.457989644	-1.120294234	-1.325539348	-1.083141235	1.049630768	0.97819563	0.748461233	1.963474124
TK1	0.0338625	0.023654822	-2.23786383	-1.926865295	-2.395928676	-1.899695094	2.526068812	2.10433666	4.314696526	3.244887059
TLL2	0.0167128	0.485517241	-4.130365444	-3.293358943	-3.977099598	-4.100360306	-2.727379545	-2.878321443	-2.358453971	-3.345197874
TLR4	0.00774626	2.538461538	-4.522840789	-3.936773198	-4.904008087	-4.161007907	-5.473931188	-6.380821784	-5.853084152	-5.24879339
TM6SF1	0.000416938	2.035830619	2.592158002	2.59454855	2.601696516	2.773996325	1.432959407	1.59454855	1.22650853	2.073820233
TMEM121	0.00212341	0.272138229	-3.293358943	-2	-4.970299766	-3.070966521	-1.293358943	-0.763660461	-1.058893689	-1.411195433
TMEM132B	0.035164	3.273809524	-3.011587974	-2.490050854	-3.857259828	-3.863546091	-5.333516069	-3.98165069	-6.10780329	-4.970299766
TMEM139	0.00403098	0.21875	0.028569152	-0.027674958	-0.413115187	0.782408565	1.713695815	2.724650272	2.715893371	2.010779839
TMEM173	0.0054953	2.615819209	5.409390936	5.997744026	5.432959407	5.145677455	3.733354341	4.053111336	4.689299161	3.925999419
TMEM178B	0.0330777	6.060606061	-6.068543859	-4.556393349	-4.824188006	-6.493296513	-9.28771238	-8.516883334	-8.175012247	-6.737735237
TMEM179	0.0419459	0.245341615	-2.171368418	-1.736965594	-0.910501849	-0.926865295	0.344828497	-0.758769964	0.831877241	1.480265122
TMEM185B	0.000819622	0.451764706	-0.332789088	-0.586405918	-0.588573754	-0.080087911	1.042644337	0.731183242	0.495695163	0.739848103
TMEM200A	0.0226115	2.101123596	-1.98279071	-2.321928095	-3.083141235	-2.506352666	-4.414268267	-3.184424571	-3.54793177	-3.13289427
TMEM220	0.00598891	0.467379679	-1.60823228	-1.780908942	-0.946193556	-0.713118852	-0.131313235	-0.465938398	-0.106249498	0.22650853
TMEM220-AS1	0.00305374	0.299261993	-3.392894616	-4.684086035	-3.544560985	-3.251538767	-2.59946207	-1.666576266	-1.61705613	-1.846843212
TMEM229A	0.00277545	4.388535032	-3.490050854	-3.820106829	-3.706511798	-4.669326877	-5.921390165	-6.480357457	-6.40506933	-5.435963338
TMEM229B	0.000896183	5.426356589	0.613531653	0.847996907	0.35614381	-0.024736678	-1.275786313	-1.722610301	-3.451662024	-2.158429363
TMEM37	0.017854	0.399212598	-1.040971781	-1.411195433	-0.567040593	-1.023269779	-0.103146927	0.722466024	0.739848103	-0.279283757
TMEM44	0.000907505	2.264705882	3.925999419	4.161887682	4.053111336	3.548436625	2.462052319	2.963474124	2.817623258	2.776103988
TMEM47	1.94E-05	2.192468619	2.488000771	2.384049807	2.392317423	2.283921772	1.137503524	0.98550043	1.389566812	1.469885976
TMEM72	0.00860134	0.106896552	-3.13289427	-3.440655033	-3.431286851	-2.351074441	-0.005782353	-0.905088353	0.86393845	0.35614381
TMEM97	0.0044465	0.410472973	1.757023247	1.49057013	0.831877241	0.799087306	2.801158656	2.198494154	2.849999259	2.298658316
TMPO-AS1	0.00770786	0.263274336	-2.662003536	-3.440655033	-3.224317298	-3.058893689	-1.582079992	-1.800877358	-0.662003536	-0.852042119
TMSB15A	0.0193419	0.092576419	-1.994240731	-2.836501268	-1.905088353	-2.411195433	0.992768431	-0.560642822	1.929790998	1.416839742
TNFAIP8L3	0.0390495	2.259615385	-2.343732465	-2.184424571	-1.689659879	-2.224317298	-4.454822365	-2.251538767	-5.345197874	-2.878321443
TNFRSF10A	0.000512464	11.19453925	0.443606651	-0.051399153	-0.336427665	-0.282789701	-3.704629612	-2.634867407	-4.179187923	-4.085588556
TNFRSF10B	0.000317813	2.384937238	5.857980995	5.970393538	5.799605422	5.686500527	4.760220946	5	3.797012978	4.504620392
TNFRSF10C	0.000340011	5.76433121	2.23572706	1.531069493	1.682573297	1.879705766	-0.120294234	-0.696657606	-1.080087911	-0.991369695
TNFRSF10D	5.80E-05	3.086592179	1.327687364	1.220329955	0.918386234	1.09085343	-0.365871442	-0.168122759	-0.744197163	-0.729770093
TNFRSF11B	0.00174995	5.176470588	-0.659722595	-0.63039393	-0.994240731	-1.595096878	-3.799872346	-2.351074441	-3.687799537	-3.988504361
TNFRSF21	0.024902	2.14673913	2.321928095	1.996388746	1.786596362	1.752748591	0.855989697	1.815575429	-0.490050854	0.411426246
TNFSF9	0.00153167	3.112582781	-2.680382066	-2.795859283	-2.899695094	-2.943416472	-6.04930764	-5.127841043	-3.46434514	-4.40812913
TNNI3	0.00980035	0.18559322	5.361066489	4.744161096	5.658211483	5.82527683	7.599912842	7.539158811	8.603626345	7.451211112

TNNT1	0.0390963	0.235294118	4.044394119	4.882643049	5.311067102	4.711494907	5.738767837	6.857980995	7.741466986	6.515699838
TNS2	0.00168498	0.339759036	-0.072482754	0.389566812	0.757023247	0.757023247	1.700439718	2.397802962	2.207892852	1.794935663
TONSL	0.0032753	0.407053942	-0.075520008	0.286881148	-0.128156351	-0.249822294	1.150559677	1.169925001	1.704871964	0.941106311
TONSL-AS1	0.0076657	0.424528302	-2.582079992	-2.652901329	-3.673002535	-2.888968688	-1.486004021	-1.314732593	-1.713118852	-2.272297327
TOP2A	0.0449281	0.010466867	-3.624154275	-5.158429363	-3.456405136	-3.682232861	2.807354922	1.327687364	1.673556424	3.797012978
TP53INP1	0.000995086	2.352231604	4.263034406	4.070389328	4.378511623	4.399171094	3.472487771	2.424922088	2.50589093	3.459431619
TPBG	0.00771052	2.187830688	-0.352915787	0.124328135	-0.36215794	-0.603840511	-1.821126042	-0.798366139	-1.477944251	-1.77102743
TPO	0.0216456	2.536679537	-1.258425153	-0.298672743	-0.305788392	-0.763660461	-2.293358943	-0.954557029	-2.965784285	-2.411195433
TPX2	0.00138625	0.017862595	-2.210896782	-2.126580497	-2.171368418	-1.883635243	3.765534746	2.702657543	4.078951341	3.95419631
TRAIP	0.031306	0.093506494	-3.046921047	-2.867752202	-2.40354186	-2.943416472	-0.150400989	-0.3382504	1.565597176	0.59454855
TRANK1	0.00295576	3.551912568	0.454175893	0.831877241	1.169925001	1.269033146	-1.217591435	-0.514573173	-2.921390165	-0.098505545
TRIM14	0.0202681	0.496801706	-1.937878288	-1.862496476	-2.13289427	-2.564904848	-1.377069649	-1.049904906	-0.588573754	-1.53951953
TRIM22	9.39E-06	8.175675676	0.367371066	0.097610797	0.443606651	0.176322773	-3.011587974	-1.988504361	-3.824188006	-2.76611194
TRIM59	0.00149554	0.496717724	-2.358453971	-2.145605322	-1.926865295	-2.158429363	-1.426625474	-1.286304185	-0.974262439	-0.894321922
TRIM9	3.51E-05	5.268292683	3.655351829	3.510961919	3.313245852	3.185866545	0.790772038	1.014355293	0.90303827	1.372952098
TRIP13	0.00347926	0.140909091	-2.388355457	-2.314732593	-2.53951953	-2.473931188	0.669026766	-0.179514657	-0.100051014	0.895302621
TROAP	0.0143731	0.005071521	-3.640253953	-4.744680559	-6.097887821	-5.380821784	2.553360503	2.028569152	3.827819025	2.715893371
TRPM3	0.0478801	2.346002621	-3.058893689	-2.046921047	-3	-2.139235797	-4.216250017	-4.189680297	-4.51292532	-2.708396442
TSGA10	0.00754503	2.025641026	2.163498732	2.691534165	2.13422094	1.855989697	1.263034406	1.182692298	1.304511042	1.137503524
TSPAN11	1.06E-05	4.833333333	2.543495883	2.480265122	2.17951105	2.304511042	0.389566812	-0.054392297	-0.072482754	0.150559677
TSPAN32	0.0204251	0.408366534	4.185866545	4.145677455	4.523561956	4.5360529	5.173926932	5.855491443	6.191799501	5.070389328
TSPEAR	0.000587149	2.11	0.815575429	1.111031312	1.063502942	1.263034406	-0.354759487	-0.002888279	0.35614381	-0.052894948
TSPEAR-AS1	0.00526993	2.010075567	-0.378944497	-0.687334826	-0.169744676	-0.13289427	-0.883635243	-1.469929258	-2.095419565	-1.142417045
TSPO	0.0277454	0.384066587	1.469885976	1.669026766	1.673556424	1.922197848	2.742006211	2.857980995	3.776103988	2.584962501
TTC39A-AS1	0.00359656	2.046936115	-2.795859283	-3.011587974	-2.652901329	-2.329159664	-3.678533641	-4.080698059	-3.41734766	-3.715959736
TTK	0.0201271	0.017317073	-4.395928676	-3.19759996	-4.254289378	-5.787866492	1.201633861	-0.142417045	2.440952198	2.15704371
TUBA1B	0.0036059	0.346938776	5.193771743	5.161887682	4.857980995	5.10433666	6.419538892	6.112700133	7.055282436	6.700439718
TUBA1C	0.000195496	0.456521739	7.515699838	7.483815777	7.17990909	7.348728154	8.459431619	8.290018847	8.721099189	8.588714636
TUBA8	1.53E-05	2.546153846	-1.526992432	-1.486004021	-1.60823228	-1.775959726	-3.184424571	-2.805912948	-2.921390165	-2.910501849
TUBB	0.000541426	0.485981308	7.375039431	7.409390936	7.108524457	7.247927513	8.21916852	8.139551352	8.603626345	8.303780748
TUBB1	0.00836784	2.402515723	-1.311148256	-0.902389203	-1.698997744	-1.831357964	-2.662003536	-2.746615764	-2.61705613	-2.573466862
UBA7	0.0104942	0.184012539	-2.145605322	-1.268816758	-0.40927823	-0.069451881	0.731183242	1.5360529	2.324810603	1.678071905
UBASH3B	0.0148536	8.899082569	-1.035046947	-0.706041021	-1.883635243	-2.490050854	-5.04930764	-3.171368418	-5.878321443	-5.930160375
UBE2C	0.00442982	0.006883117	-3.912672948	-3.750493979	-1.971430848	-2.064917477	4.439623138	3.289834465	4.716990894	5.112700133
UBE2T	0.0118651	0.180672269	1.333423734	0.782408565	1.339137385	0.887525271	3.459431619	2.214124805	4.09592442	3.887525271
UBR5-AS1	0.00941226	2.404661017	-2.184424571	-2.19759996	-2.60823228	-1.713118852	-3.649276466	-2.816037165	-4.348133165	-3.23786383
UCP2	0.0315642	0.377136752	2.077242999	2.13093087	1.411426246	1.516015147	3.153805336	2.916476644	3.925999419	2.538538164
UHRF1	0.0131418	0.021686047	-5.112786697	-5.795859283	-5.435963338	-3.662003536	0.669026766	0.310340121	-0.086201035	1.641546029
ULK4	0.019886	0.37398374	0.163498732	0.22650853	0.584962501	0.799087306	1.459431619	1.280956314	2.485426827	1.996388746
UNC5D	0.000251951	3.413580247	-0.749038426	-0.720231578	-0.997117491	-0.977099598	-2.307572802	-3.775959726	-3.145605322	-1.943416472
UNC80	0.00458231	4.175824176	-2.120294234	-1.53951953	-1.286304185	-2.114035243	-6.380821784	-2.785875195	-4.200249538	-3.656535324
UPP1	0.0370243	2.283737024	6.596935142	7.592457037	7.159871337	6.563768278	4.608809243	6.373300197	6.395748328	5.314696526
URB1-AS1	0.0433197	0.406417112	-4.662003536	-5.680382066	-5.467533417	-6.678903137	-4.611755347	-3.522840789	-4.764150423	-4.066125258

USH1C	0.00518514	0.465693431	1.459431619	1.15704371	2.017921908	1.895302621	2.538538164	2.528571319	3.160274831	2.780310099
USP18	4.38E-05	9.032258065	0.847996907	0.992768431	1.263034406	0.713695815	-2.177881725	-2.279283757	-2.373327247	-2.017417053
USP41	0.000109306	7.687861272	-1.092340172	-0.736965594	-0.666576266	-1.224317298	-4.092955525	-3.469130202	-5.054092703	-3.354021725
USP44	0.0428792	2.242798354	-1.058893689	-0.349235441	-1.53951953	-0.803392956	-2.59946207	-3.702749879	-1.407363571	-1.49410907
VAT1L	0.00127225	3.53626943	1.726831217	1.695993813	1.176322773	1.077242999	-0.043943348	-0.177881725	-1.430508908	-0.209227962
VEPH1	0.00132927	0.217625899	-1.055891201	-1.164884385	-0.569179503	-0.286304185	1.40599236	0.855989697	1.843983844	1.613531653
VRK1	0.0229266	0.376404494	1.63691458	1.339137385	1.15704371	1.500802053	2.419538892	1.992768431	3.305970521	3.212569339
VWCE	0.00141958	2.956043956	1.150559677	1.778208576	1.565597176	1.10433666	-0.304006187	0.176322773	-0.035046947	-0.467932448
WASF1	0.000560398	2.204359673	3.244887059	2.927896454	2.969012308	2.893362211	2.250961574	1.744161096	1.35614381	2
WDR62	0.0209901	0.429842932	2.983677695	2.965322548	3.142413438	3.051372102	4.343407822	3.867896464	4.832890014	3.700439718
WDR63	0.000423309	5.996503497	2.03562391	2.046141782	1.438292852	1.459431619	-0.373327247	-0.862496476	-1.351074441	-0.803392956
WDR66	0.0111233	2.255717256	-2.083141235	-1.826232932	-2.293358943	-2.756330919	-3.011587974	-3.356975042	-4.493296513	-3.070966521
WDR76	0.00922879	0.182	-1.926865295	-2.126580497	-1.634867407	-1.836501268	0.310340121	-0.189351252	0.584962501	1.263034406
WHRN	0.00362392	2.458015267	2.879705766	3.016139703	2.266036894	2.462052319	1.316145742	1.786596362	1.350497247	0.992768431
WIPF3	0.0125276	2.22972973	-0.329159664	-0.274040765	-0.798366139	-1.177881725	-1.756330919	-1.217591435	-2.210896782	-2.040971781
WNT11	0.00167306	0.335459184	1.40599236	1.077242999	1.422233001	1.627606838	2.698218478	3.320484678	3.147306699	2.59454855
WSCD1	0.00109535	0.195167286	-3.483581358	-3.265344567	-3.660178495	-2.756330919	-0.446148032	-0.862496476	-1.318325858	-1.10780329
WSCD2	0.0218896	5	0.485426827	1.263034406	0.411426246	-0.621488377	-2.070966521	-0.732164608	-2.321928095	-3.158429363
WWOX	0.00813839	0.465393795	1.169925001	0.575312331	0.704871964	1.292781749	2.50589093	2.025028794	1.713695815	1.891419187
XKR5	0.0390589	0.171428571	-4.24331826	-3.882570916	-4.580353247	-5.651087759	-2.23786383	-3.700872592	-1.732164608	-1.077041036
XPC	3.94E-05	3.572368421	5.842978832	5.930737338	5.675251386	5.581953751	3.925999419	4.392317423	3.419538892	3.797012978
XRCC2	0.0439203	0.463492063	-2.910501849	-2.481968507	-2.727379545	-3.058893689	-1.727379545	-1.888968688	-2.388355457	-1.002888279
XRCC4	0.000311365	3.614130435	6.301953395	6.205548911	5.906890596	5.730639956	4.240314329	3.472487771	4.392317423	4.491853096
XYLT1	0.0419176	2.12565445	-0.732164608	-1.746615764	-1.61705613	-1.332789088	-1.751465164	-2.60823228	-3.748553568	-2.126580497
YBX2	0.0273287	0.363636364	0.028569152	0.432959407	0.056583528	0.111031312	1.207892852	1.687060688	2.298658316	0.910732662
YPEL4	0.0111884	2.149038462	-1.336427665	-1.318325858	-1.12973393	-0.907792562	-3.13289427	-1.53951953	-3.184424571	-1.915935735
ZBBX	0.00166293	3.393829401	0.650764559	0.847996907	0.669026766	1.344828497	-1.080087911	-1.556393349	-0.621488377	-0.434402824
ZBTB32	0.0172116	2.304347826	-0.347398782	-0.16326792	-0.531156057	-0.739372092	-2.321928095	-0.862496476	-3.8303315	-1.095419565
ZCCHC12	0.00215281	5.754385965	-0.490050854	-0.117161344	-0.865121946	-1.181149439	-3.342268543	-2.717856771	-3.120294234	-3.451662024
ZDHHC22	0.0321781	2.112068966	-2.13289427	-2.040971781	-3.10780329	-2.336427665	-3.995390747	-2.59946207	-3.627716487	-3.988504361
ZFP57	0.00605789	3.729957806	-0.623709617	0.367371066	-0.148800661	-0.531156057	-2.727379545	-1.648371671	-1.573466862	-2.816037165
ZFYVE16	0.00133184	2.144329897	6.857980995	6.845490051	6.62935662	6.451211112	5.520422249	5.847996907	4.87774425	5.933100475
ZIM3	0.00176623	3.038869258	-4.938984225	-5.254289378	-5.546245393	-5.435963338	-6.467533417	-6.506352666	-8.751659479	-6.731589562
ZMAT3	0.000668807	2.434052758	4.399171094	4	4.504620392	4.426264755	3.350497247	2.414135533	2.970853654	3.321928095
ZNF367	0.0100598	0.312753036	-1.634867407	-1.590744853	-1.531156057	-2.070966521	-0.862496476	0.176322773	-0.192645078	0.485426827
ZNF385B	0.0104855	2.036144578	1.992768431	2.577730931	2.61117238	2.080657663	1.691534165	0.545968369	1.097610797	1.646162657
ZNF385D	0.0113293	2.466019417	-1.857259828	-2.775959726	-1.595096878	-1.932361283	-3.575185379	-3.490050854	-2.736965594	-3.504714171
ZNF423	0.000278999	3.190954774	0.50589093	0.485426827	0.275007047	0.097610797	-0.720231578	-1.77102743	-1.977099598	-1.194294815
ZNF467	0.00332056	0.434883721	0.807354922	0.910732662	0.918386234	0.97819563	2.253989266	2.049630768	2.416839742	1.555816155
ZNF488	0.0400688	2.71450858	-2.977099598	-2.011587974	-2.465938398	-2.826232932	-3.676687582	-10.00231016	-5.088040035	-2.756330919
ZNF503-AS2	0.00261478	0.463636364	-1.502259911	-0.971430848	-1.582079992	-2.114035243	-0.300448367	-0.347398782	-0.179514657	-0.74178261
ZNF561	0.00102046	3.243119266	6.385431037	6.400879436	5.997744026	5.669593751	4.638073837	4.392317423	4.201633861	4.510961919
ZNF793-AS1	0.00393051	2.055944056	1.580145484	1.454175893	1.722466024	1.443606651	0.454175893	-0.332789088	1.169925001	0.389566812

ZNF93	0.000825215	0.456445993	-1.648371671	-1.883635243	-2.152003093	-2.083141235	-0.888968688	-1.049904906	-0.516635639	-0.805912948
ZSWIM5	0.00322991	2.185279188	-0.360304767	-0.446148032	-0.051399153	-0.052894948	-1.490050854	-1.921390165	-1.577766999	-0.691988685
ZWINT	0.0108831	0.072142857	-0.236163541	-0.075520008	0.367371066	-0.080087911	3.314696526	2.613531653	4.201633861	4.419538892

GO Clusters-upregulated genes				
Annotation Cluster 1	Enrichment Score: 2.0340832756829164			
Category	Term	Number of genes in cluster	P-Value	Genes
GOTERM_MF_DIRECT	GO:0005031~tumor necrosis factor-activated receptor activity	8	6.28E-06	TNFRSF10A, TNFRSF21, TNFRSF10C, TNFRSF11B, TNFRSF10B, TNFRSF10D, EDA2R, FAS
GOTERM_MF_DIRECT	GO:0045569~TRAIL binding	4	1.20E-04	TNFRSF10A, TNFRSF10C, TNFRSF10B, TNFRSF10D
GOTERM_BP_DIRECT	GO:0042981~regulation of apoptotic process	18	4.69E-04	EGR1, TNFRSF21, SGK1, GDF2, PMAIP1, ALK, SOX9, TNFRSF10A, GLS2, TNFRSF10C, TNFRSF11B, TNFRSF10B, TNFRSF10D, BBC3, BAX, FAS, GDF15, TP53INP1
GOTERM_BP_DIRECT	GO:0008625~extrinsic apoptotic signaling pathway via death domain receptors	7	0.001156	TNFRSF10A, TNFRSF10C, TNFRSF10B, TNFRSF10D, BAX, FAS, NGF
GOTERM_BP_DIRECT	GO:0071260~cellular response to mechanical stimulus	9	0.001797	TNFRSF10A, EGFR, TNFRSF10B, PTGER4, TLR4, FAS, KCNJ2, SOX9, GADD45A
GOTERM_BP_DIRECT	GO:0042127~regulation of cell proliferation	15	0.002377	TNFRSF21, SGK1, BLK, JAG1, SOX9, BTK, TNFRSF10A, TNFRSF11B, TNFRSF10C, TNFRSF10B, MELTF, TNFRSF10D, ACE2, FAS, PLAU
KEGG_PATHWAY	hsa04210:Apoptosis	8	0.004704	TNFRSF10A, TNFRSF10C, TNFRSF10B, TNFRSF10D, BAX, FAS, AKT3, NGF
GOTERM_BP_DIRECT	GO:0097296~activation of cysteine-type endopeptidase activity involved in apoptotic signaling pathway	4	0.007171	TNFRSF10A, TNFRSF10B, BAX, FAS
GOTERM_BP_DIRECT	GO:0032496~response to lipopolysaccharide	12	0.01567	TNFRSF10A, TNFRSF21, FOS, TNFRSF10C, TNFRSF11B, TNFRSF10B, PTGER4, TNFRSF10D, CNR1, FGF10, TLR4, FAS
GOTERM_BP_DIRECT	GO:0033209~tumor necrosis factor-mediated signaling pathway	8	0.081852	TNFRSF10A, TNFRSF21, TNFRSF10C, TNFRSF11B, TNFRSF10B, TNFRSF10D, EDA2R, FAS
GOTERM_BP_DIRECT	GO:1902041~regulation of extrinsic apoptotic signaling pathway via death domain receptors	3	0.099961	TNFRSF10A, TNFRSF10B, FAS
GOTERM_BP_DIRECT	GO:0097191~extrinsic apoptotic signaling pathway	4	0.147812	TNFRSF10A, BAX, FAS, TGFB2
KEGG_PATHWAY	hsa05162:Measles	8	0.164303	TNFRSF10A, TNFRSF10C, TNFRSF10B, TNFRSF10D, BBC3, TLR4, FAS, AKT3
KEGG_PATHWAY	hsa04650:Natural killer cell mediated cytotoxicity	7	0.232102	TNFRSF10A, TNFRSF10C, TNFRSF10B, TNFRSF10D, FAS, PRKCB, SYK
KEGG_PATHWAY	hsa05164:Influenza A	9	0.236881	TNFRSF10A, TNFRSF10C, TNFRSF10B, TNFRSF10D, PRSS3, TLR4, FAS, AKT3, PRKCB
GOTERM_BP_DIRECT	GO:1902042~negative regulation of extrinsic apoptotic signaling pathway via death domain receptors	3	0.281954	TNFRSF10A, TNFRSF10B, FAS
Annotation Cluster 2	Enrichment Score: 1.96543798643632			
Category	Term	Number of genes in cluster	PValue	Genes
KEGG_PATHWAY	hsa04724:Glutamatergic synapse	13	4.45E-04	GLS2, DLGAP1, GRIN2B, GRIK1, GRIA1, ADCY8, GRIK4, GRIN2A, PLCB1, ITPR1, CACNA1A, PRKCB, ITPR2
GOTERM_BP_DIRECT	GO:0007215~glutamate receptor signaling pathway	5	0.001036	GRIN2B, GRIK1, GRIK4, GRIN2A, PLCB1

GOTERM_MF_DIRECT	GO:0005234~extracellular-glutamate-gated ion channel activity	5	0.00207	GRIN2B, GRIK1, GRIA1, GRIK4, GRIN2A
GOTERM_BP_DIRECT	GO:0035235~ionotropic glutamate receptor signaling pathway	5	0.006436	GRIN2B, GRIK1, GRIA1, GRIK4, GRIN2A
GOTERM_MF_DIRECT	GO:0004970~ionotropic glutamate receptor activity	4	0.010565	GRIK1, GRIA1, GRIK4, GRIN2A
KEGG_PATHWAY	hsa05014:Amyotrophic lateral sclerosis (ALS)	6	0.026222	GRIN2B, GRIA1, BAX, GRIN2A, NEFL, NEFM
KEGG_PATHWAY	hsa05033:Nicotine addiction	5	0.045224	GRIN2B, GRIA1, GABRB2, GRIN2A, CACNA1A
KEGG_PATHWAY	hsa04080:Neuroactive ligand-receptor interaction	14	0.144219	F2RL2, PTGER4, GRIK1, GABRB2, GRIK4, GRIN2A, NPY1R, GRIN2B, GRIA1, CNR1, PRSS3, GALR2, GPR50, CHRNA1
KEGG_PATHWAY	hsa05031:Amphetamine addiction	5	0.184229	FOS, GRIN2B, GRIA1, GRIN2A, PRKCB

Annotation Cluster 3 Enrichment Score: 1.748803401827624

Category	Term	Number of genes in cluster	PValue	Genes
GOTERM_MF_DIRECT	GO:0005003~ephrin receptor activity	4	0.004206	EPHA4, EPHA2, EPHA3, EPHB2
GOTERM_BP_DIRECT	GO:0018108~peptidyl-tyrosine phosphorylation	11	0.024218	EGFR, NTRK3, EPHA4, FLT1, NTRK2, FGF10, ALK, EPHA2, EPHA3, BTK, EPHB2
GOTERM_BP_DIRECT	GO:0048013~ephrin receptor signaling pathway	7	0.055666	NTRK3, EPHA4, GRIN2B, MMP2, EPHA2, EPHA3, EPHB2

Annotation Cluster 4 Enrichment Score: 1.7256967144248567

Category	Term	Number of genes in cluster	PValue	Genes
GOTERM_BP_DIRECT	GO:0007169~transmembrane receptor protein tyrosine kinase signaling pathway	10	0.003428	EGFR, NTRK3, FLT1, BLK, NTRK2, ALK, NGF, SYK, BTK, IL31RA
GOTERM_MF_DIRECT	GO:0004714~transmembrane receptor protein tyrosine kinase activity	6	0.006386	EGFR, NTRK3, FLT1, NTRK2, ALK, EPHA2
GOTERM_BP_DIRECT	GO:0018108~peptidyl-tyrosine phosphorylation	11	0.024218	EGFR, NTRK3, EPHA4, FLT1, NTRK2, FGF10, ALK, EPHA2, EPHA3, BTK, EPHB2
GOTERM_MF_DIRECT	GO:0004713~protein tyrosine kinase activity	10	0.024445	EGFR, EPHA4, FLT1, BLK, FGF10, ALK, EPHA2, SYK, BTK, EPHB2
GOTERM_BP_DIRECT	GO:0046777~protein autophosphorylation	9	0.181486	EGFR, NTRK3, EPHA4, FLT1, NTRK2, LRRK2, ALK, PRKX, SYK

Annotation Cluster 5 Enrichment Score: 1.2779357832370883

Category	Term	Number of genes in cluster	PValue	Genes
GOTERM_BP_DIRECT	GO:0006919~activation of cysteine-type endopeptidase activity involved in apoptotic process	9	0.00479	TNFRSF10A, TNFRSF10B, BBC3, ACER2, BAX, RPS27L, FAS, PMAIP1, NGF
GOTERM_BP_DIRECT	GO:0001844~protein insertion into mitochondrial membrane involved in apoptotic signaling pathway	3	0.018996	BBC3, BAX, PMAIP1
GOTERM_BP_DIRECT	GO:0070059~intrinsic apoptotic signaling pathway in response to endoplasmic reticulum stress	5	0.019819	TNFRSF10B, BBC3, BAX, PMAIP1, ITPR1

GOTERM_BP_DIRECT	GO:0043525~positive regulation of neuron apoptotic process	5	0.046759	BBC3, BAX, ITGA1, PMAIP1, TGFB2
GOTERM_BP_DIRECT	GO:0097193~intrinsic apoptotic signaling pathway	4	0.068571	CDKN1A, BBC3, BAX, PMAIP1
GOTERM_BP_DIRECT	GO:0001836~release of cytochrome c from mitochondria	3	0.164655	BBC3, BAX, PMAIP1
GOTERM_BP_DIRECT	GO:0090200~positive regulation of release of cytochrome c from mitochondria	3	0.222665	BBC3, BAX, PMAIP1
GOTERM_BP_DIRECT	GO:2001244~positive regulation of intrinsic apoptotic signaling pathway	3	0.281954	BBC3, BAX, PMAIP1

Annotation Cluster 6 Enrichment Score: 1.1551575497965025

Category	Term	Number of genes in cluster	PValue	Genes
GOTERM_BP_DIRECT	GO:0000188~inactivation of MAPK activity	4	0.043577	DUSP4, DUSP2, SPRED2, DUSP6
GOTERM_MF_DIRECT	GO:0017017~MAP kinase tyrosine/serine/threonine phosphatase activity	3	0.061056	DUSP4, DUSP2, DUSP6
GOTERM_MF_DIRECT	GO:0004725~protein tyrosine phosphatase activity	7	0.094749	PTPRB, PTPN7, DUSP4, DUSP2, UBASH3B, PTP4A1, DUSP6
GOTERM_BP_DIRECT	GO:0035335~peptidyl-tyrosine dephosphorylation	7	0.095019	PTPRB, PTPN7, DUSP4, DUSP2, UBASH3B, PTP4A1, DUSP6

Annotation Cluster 7 Enrichment Score: 1.1249795255998831

Category	Term	Number of genes in cluster	PValue	Genes
GOTERM_MF_DIRECT	GO:0015254~glycerol channel activity	3	0.052733	MIP, AQP10, AQP3
GOTERM_BP_DIRECT	GO:0015793~glycerol transport	3	0.062249	MIP, AQP10, AQP3
GOTERM_BP_DIRECT	GO:0009992~cellular water homeostasis	3	0.062249	MIP, AQP10, AQP3
GOTERM_MF_DIRECT	GO:0015250~water channel activity	3	0.088358	MIP, AQP10, AQP3
GOTERM_BP_DIRECT	GO:0006833~water transport	3	0.131373	MIP, AQP10, AQP3

Annotation Cluster 8 Enrichment Score: 1.0924426075986888

Category	Term	Number of genes in cluster	PValue	Genes
GOTERM_BP_DIRECT	GO:0017158~regulation of calcium ion-dependent exocytosis	5	0.024138	SYT11, SYT14, SYTL5, SYTL1, CACNA1A
GOTERM_BP_DIRECT	GO:0048791~calcium ion-regulated exocytosis of neurotransmitter	5	0.034367	SYT11, SYT14, SYTL5, SYTL1, CACNA1A
GOTERM_CC_DIRECT	GO:0098793~presynapse	6	0.048716	NRXN3, SYT11, SYTL5, NRXN1, SYTL1, CACNA1A
GOTERM_MF_DIRECT	GO:0030276~clathrin binding	5	0.079954	SYT11, SYT14, SYTL5, LRRK2, SYTL1
GOTERM_MF_DIRECT	GO:0005544~calcium-dependent phospholipid binding	5	0.108669	SYT11, SYT14, SYTL5, ANXA4, SYTL1
GOTERM_MF_DIRECT	GO:0019905~syntaxin binding	5	0.22266	SYT11, SYT14, SYTL5, SYTL1, CACNA1A

GOTERM_BP_DIRECT	GO:0006906~vesicle fusion	4	0.288266	SYT11, SYT14, SYTL5, SYTL1

Annotation Cluster 9 Enrichment Score: 1.088370776834853

Category	Term	Number of genes in cluster	PValue	Genes
GOTERM_MF_DIRECT	GO:0005248~voltage-gated sodium channel activity	4	0.023575	HCN1, SCN2A, SCN4B, SCN7A
GOTERM_BP_DIRECT	GO:0060078~regulation of postsynaptic membrane potential	4	0.031277	HCN1, SCN2A, SCN4B, SCN7A
GOTERM_CC_DIRECT	GO:0001518~voltage-gated sodium channel complex	3	0.068693	SCN2A, SCN4B, SCN7A
GOTERM_BP_DIRECT	GO:0035725~sodium ion transmembrane transport	6	0.082098	HCN1, SLC4A11, SLC24A3, SCN2A, SCN4B, SCN7A
GOTERM_BP_DIRECT	GO:0006814~sodium ion transport	5	0.255936	SGK1, SLC4A11, SCN2A, SCN4B, SCN7A
GOTERM_BP_DIRECT	GO:0034765~regulation of ion transmembrane transport	6	0.277147	HCN1, KCNQ3, SCN2A, KCNH8, SCN7A, CACNA1A

Annotation Cluster 10 Enrichment Score: 1.0841538081814073

Category	Term	Number of genes in cluster	PValue	Genes
GOTERM_BP_DIRECT	GO:0007612~learning	7	0.009168	SLC6A1, NRXN3, NLGN4X, NTRK2, NRXN1, ATP8A1, EPHB2
GOTERM_BP_DIRECT	GO:0007158~neuron cell-cell adhesion	4	0.013084	NRXN3, NLGN4X, ASTN1, NRXN1
GOTERM_BP_DIRECT	GO:0071625~vocalization behavior	3	0.071139	NRXN3, NLGN4X, NRXN1
KEGG_PATHWAY	hsa04514:Cell adhesion molecules (CAMs)	9	0.108395	NRCAM, F11R, SDC1, NRXN3, CD274, NLGN4X, CLDN1, NRXN1, SDC3
GOTERM_BP_DIRECT	GO:0030534~adult behavior	3	0.199196	NRXN3, NLGN4X, NRXN1
GOTERM_MF_DIRECT	GO:0050839~cell adhesion molecule binding	4	0.308565	CPE, NRXN3, NLGN4X, NRXN1
GOTERM_BP_DIRECT	GO:0035176~social behavior	3	0.453075	NRXN3, NLGN4X, NRXN1

Annotation Cluster 11 Enrichment Score: 1.0823080537357794

Category	Term	Number of genes in cluster	PValue	Genes
KEGG_PATHWAY	hsa04068:FoxO signaling pathway	11	0.015232	EGFR, SGK1, CDKN1A, PLK3, PLK2, MDM2, FBXO32, AGAP2, GADD45A, AKT3, TGFB2
GOTERM_BP_DIRECT	GO:0006977~DNA damage response, signal transduction by p53 class mediator resulting in cell cycle arrest	6	0.046628	CDKN1A, PLK3, PLK2, BAX, MDM2, GADD45A
KEGG_PATHWAY	hsa04110:Cell cycle	4	0.797375	CDKN1A, MDM2, GADD45A, TGFB2

Annotation Cluster 12 Enrichment Score: 0.9861970140291849

Category	Term	Number of genes in cluster	PValue	Genes
GOTERM_MF_DIRECT	GO:0030552~cAMP binding	4	0.038182	HCN1, PDE4B, PDE11A, PDE3A

GOTERM_MF_DIRECT	GO:0004115~3',5'-cyclic-AMP phosphodiesterase activity	3	0.078901	PDE4B, PDE11A, PDE3A
GOTERM_BP_DIRECT	GO:0006198~cAMP catabolic process	3	0.080409	PDE4B, PDE11A, PDE3A
KEGG_PATHWAY	hsa05032:Morphine addiction	7	0.087547	ADCY8, GABRB2, PDE4B, PDE11A, PDE3A, CACNA1A, PRKCB
KEGG_PATHWAY	hsa00230:Purine metabolism	7	0.552751	ADCY8, PDE4B, PDE11A, PDE3A, RRM2B, PAPSS2, AMPD3

Annotation Cluster 13 Enrichment Score: 0.9775675686153424

Category	Term	Number of genes in cluster	PValue	Genes
GOTERM_MF_DIRECT	GO:0005160~transforming growth factor beta receptor binding	5	0.042024	RASL11B, BMP2, GDF2, GDF15, TGFB2
GOTERM_BP_DIRECT	GO:0060389~pathway-restricted SMAD protein phosphorylation	3	0.062249	BMP2, GDF2, TGFB2
GOTERM_BP_DIRECT	GO:0060395~SMAD protein signal transduction	5	0.1338	FOS, BMP2, GDF2, GDF15, TGFB2
GOTERM_MF_DIRECT	GO:0005125~cytokine activity	9	0.189762	LIF, BMP2, TNFRSF11B, GDF2, IL23A, KITLG, TNFSF9, GDF15, TGFB2
GOTERM_BP_DIRECT	GO:0010862~positive regulation of pathway-restricted SMAD protein phosphorylation	4	0.194922	BMP2, GDF2, GDF15, TGFB2

Annotation Cluster 14 Enrichment Score: 0.9442881615013738

Category	Term	Number of genes in cluster	PValue	Genes
GOTERM_BP_DIRECT	GO:0008090~retrograde axonal transport	3	0.024801	NEFL, HAP1, MGARP
GOTERM_BP_DIRECT	GO:0008089~anterograde axonal transport	3	0.199196	NEFL, HAP1, MGARP
GOTERM_CC_DIRECT	GO:1904115~axon cytoplasm	3	0.297434	NEFL, HAP1, MGARP

Annotation Cluster 15 Enrichment Score: 0.8917282456238629

Category	Term	Number of genes in cluster	PValue	Genes
KEGG_PATHWAY	hsa05161:Hepatitis B	11	0.025004	FOS, CDKN1A, EGR2, BAX, DDB2, TLR4, NFATC4, FAS, AKT3, PRKCB, TGFB2
KEGG_PATHWAY	hsa05142:Chagas disease (American trypanosomiasis)	8	0.062245	FOS, CCL3L3, TLR4, FAS, PLCB1, PPP2R2C, AKT3, TGFB2
KEGG_PATHWAY	hsa05210:Colorectal cancer	4	0.350897	FOS, BAX, AKT3, TGFB2
KEGG_PATHWAY	hsa05166:HTLV-I infection	10	0.496354	EGR1, FOS, CDKN1A, EGR2, ADCY8, BAX, NFATC4, AKT3, TGFB2, TP53INP1

Annotation Cluster 16 Enrichment Score: 0.8704394297006569

Category	Term	Number of genes in cluster	PValue	Genes
GOTERM_CC_DIRECT	GO:0031463~Cul3-RING ubiquitin ligase complex	7	0.017759	KLHL29, BACH2, ENC1, KBTBD8, KLHL4, KLHDC7A, KLHL20
GOTERM_MF_DIRECT	GO:0004842~ubiquitin-protein transferase activity	14	0.23331	RNF144B, BACH2, HERC5, KLHL4, KLHDC7A, ASB18, KLHL29, TRIM9, KBTBD8, DDB2, MDM2, FBXO32, FBXO22, KLHL20
GOTERM_BP_DIRECT	GO:0016567~protein ubiquitination	12	0.590645	KLHL29, BACH2, TRIM9, ENC1, KBTBD8, MDM2, FBXO32, KLHL4, TRIM22, KLHDC7A, ASB18, KLHL20

Annotation Cluster 17 Enrichment Score: 0.8328805302906921

Category	Term	Number of genes in cluster	PValue	Genes
GOTERM_BP_DIRECT	GO:0016266~O-glycan processing	7	0.011685	GALNT7, B3GNT7, GALNT5, MUC19, GALNT13, ST6GALNAC2, MUC4
GOTERM_MF_DIRECT	GO:0004653~polypeptide N-acetylgalactosaminyltransferase activity	3	0.129049	GALNT7, GALNT5, GALNT13
GOTERM_MF_DIRECT	GO:0030246~carbohydrate binding	9	0.274768	GALNT7, OLR1, GALNT5, FREM1, CLEC2A, CHI3L1, CHODL, KLRF2, GALNT13
KEGG_PATHWAY	hsa00512:Mucin type O-Glycan biosynthesis	3	0.282928	GALNT7, GALNT5, GALNT13
GOTERM_MF_DIRECT	GO:0016740~transferase activity	4	0.584193	GALNT7, XYLT1, GALNT5, GALNT13

Annotation Cluster 18 Enrichment Score: 0.8149162417595034

Category	Term	Number of genes in cluster	PValue	Genes
GOTERM_MF_DIRECT	GO:0015106~bicarbonate transmembrane transporter activity	3	0.078901	SLC4A11, SLC26A7, SLC26A9
GOTERM_BP_DIRECT	GO:0051453~regulation of intracellular pH	4	0.105205	SLC4A11, SLC26A7, SLC26A9, SLC9C1
GOTERM_MF_DIRECT	GO:0015301~anion:anion antiporter activity	3	0.161844	SLC4A11, SLC26A7, SLC26A9
GOTERM_BP_DIRECT	GO:0015701~bicarbonate transport	3	0.409371	SLC4A11, SLC26A7, SLC26A9

Annotation Cluster 19 Enrichment Score: 0.79124394332239

Category	Term	Number of genes in cluster	PValue	Genes
KEGG_PATHWAY	hsa05323:Rheumatoid arthritis	8	0.029086	FOS, FLT1, IL23A, CCL3L3, ATP6V1E2, TLR4, CXCL12, TGFB2
KEGG_PATHWAY	hsa05142:Chagas disease (American trypanosomiasis)	8	0.062245	FOS, CCL3L3, TLR4, FAS, PLCB1, PPP2R2C, AKT3, TGFB2
KEGG_PATHWAY	hsa05132:Salmonella infection	4	0.537704	FOS, WASF1, CCL3L3, TLR4
KEGG_PATHWAY	hsa04620:Toll-like receptor signaling pathway	4	0.702585	FOS, CCL3L3, TLR4, AKT3

Annotation Cluster 20 Enrichment Score: 0.6970085225840524

Category	Term	Number of genes in cluster	PValue	Genes
GOTERM_BP_DIRECT	GO:0045579~positive regulation of B cell differentiation	3	0.071139	INPP5D, SYK, BTK
KEGG_PATHWAY	hsa04662:B cell receptor signaling pathway	6	0.083675	FOS, CR2, INPP5D, AKT3, SYK, BTK
KEGG_PATHWAY	hsa04664:Fc epsilon RI signaling pathway	5	0.198076	INPP5D, AKT3, PRKCB, SYK, BTK
KEGG_PATHWAY	hsa04380:Osteoclast differentiation	7	0.283695	FOS, TNFRSF11B, FCGR1A, AKT3, TGFB2, SYK, BTK
GOTERM_BP_DIRECT	GO:0038095~Fc-epsilon receptor signaling pathway	3	0.97853	FOS, SYK, BTK

Annotation Cluster 21 Enrichment Score: 0.6882437812228342

Category	Term	Number of genes in cluster	PValue	Genes
KEGG_PATHWAY	hsa05223:Non-small cell lung cancer	6	0.040221	EGFR, RXRG, TGFA, ALK, AKT3, PRKCB

KEGG_PATHWAY	hsa05219:Bladder cancer	5	0.048833	EGFR, CDKN1A, MDM2, THBS1, MMP2
KEGG_PATHWAY	hsa05214:Glioma	6	0.068383	EGFR, CDKN1A, TGFA, MDM2, AKT3, PRKCB
KEGG_PATHWAY	hsa05218:Melanoma	5	0.21941	EGFR, CDKN1A, MDM2, FGF10, AKT3
KEGG_PATHWAY	hsa04066:HIF-1 signaling pathway	6	0.225269	EGFR, CDKN1A, FLT1, TLR4, AKT3, PRKCB
KEGG_PATHWAY	hsa05220:Chronic myeloid leukemia	5	0.226656	CDKN1A, MDM2, RUNX1, AKT3, TGFB2
KEGG_PATHWAY	hsa04012:ErbB signaling pathway	5	0.340491	EGFR, CDKN1A, TGFA, AKT3, PRKCB
KEGG_PATHWAY	hsa05215:Prostate cancer	5	0.348258	EGFR, CDKN1A, TGFA, MDM2, AKT3
KEGG_PATHWAY	hsa05212:Pancreatic cancer	4	0.378854	EGFR, TGFA, AKT3, TGFB2
KEGG_PATHWAY	hsa05169:Epstein-Barr virus infection	5	0.597512	CDKN1A, CR2, MDM2, AKT3, SYK
KEGG_PATHWAY	hsa05160:Hepatitis C	5	0.66535	EGFR, CDKN1A, CLDN1, PPP2R2C, AKT3

Annotation Cluster 22 Enrichment Score: 0.6326609986738824

Category	Term	Number of genes in cluster	PValue	Genes
GOTERM_BP_DIRECT	GO:0050853~B cell receptor signaling pathway	5	0.091817	BLK, RFTN1, PRKCB, SYK, BTK
KEGG_PATHWAY	hsa04664:Fc epsilon RI signaling pathway	5	0.198076	INPP5D, AKT3, PRKCB, SYK, BTK
GOTERM_BP_DIRECT	GO:0002250~adaptive immune response	5	0.695445	TNFRSF21, TAP1, PRKCB, SYK, BTK

Annotation Cluster 23 Enrichment Score: 0.6137077486820296

Category	Term	Number of genes in cluster	PValue	Genes
GOTERM_BP_DIRECT	GO:0071805~potassium ion transmembrane transport	7	0.187014	HCN1, KCNQ3, KCNT2, SLC9C1, KCNH8, KCNA5, KCNJ2
GOTERM_BP_DIRECT	GO:0006813~potassium ion transport	5	0.262945	HCN1, KCNQ3, SLC24A3, KCNA5, KCNJ2
GOTERM_CC_DIRECT	GO:0008076~voltage-gated potassium channel complex	5	0.293182	KCNQ3, KCNT2, KCNA5, KCNJ2, SNAP25

Annotation Cluster 24 Enrichment Score: 0.5795631680143967

Category	Term	Number of genes in cluster	PValue	Genes
GOTERM_BP_DIRECT	GO:0018105~peptidyl-serine phosphorylation	8	0.103172	SGK1, PLK2, LRRK2, AKT3, DCLK1, PRKX, PRKCB, SYK
GOTERM_BP_DIRECT	GO:0006468~protein phosphorylation	19	0.202362	BMP2, SGK1, CAMK1G, MYLK3, PRKG2, PRKCB, BTK, TGFB2, PRKY, TEX14, CDC42BPG, PLK3, PLK2, LRRK2, MYLK, DCLK1, AKT3, CAMK1D, SYK
GOTERM_MF_DIRECT	GO:0004674~protein serine/threonine kinase activity	14	0.399608	SGK1, CAMK1G, PRKG2, PRKX, PRKCB, PRKY, CDC42BPG, PLK3, PLK2, LRRK2, DCLK1, AKT3, SYK, CAMK1D
GOTERM_MF_DIRECT	GO:0004672~protein kinase activity	12	0.576001	PRKY, EGFR, EPHA4, TEX14, PRKG2, LRRK2, SOX9, AKT3, DCLK1, PRKCB, SYK, BTK

Annotation Cluster 25 Enrichment Score: 0.5708717807693228

Category	Term	Number of genes in cluster	PValue	Genes
GOTERM_MF_DIRECT	GO:0005254~chloride channel activity	5	0.089057	BEST3, GABRB2, SLC26A7, SLC26A9, BEST4
GOTERM_BP_DIRECT	GO:1902476~chloride transmembrane transport	5	0.341757	BEST3, GABRB2, SLC26A7, SLC26A9, BEST4
GOTERM_BP_DIRECT	GO:0006811~ion transport	6	0.377328	SLC36A1, GABRB2, SLC26A7, SLC24A3, SLC26A9, LRRC38

GOTERM_CC_DIRECT	GO:0034707~chloride channel complex	3	0.453324	BEST3, GABRB2, BEST4

Annotation Cluster 26 Enrichment Score: 0.559558504257432

Category	Term	Number of genes in cluster	PValue	Genes
GOTERM_CC_DIRECT	GO:0031093~platelet alpha granule lumen	5	0.091474	A2M, F5, F8, THBS1, TGFB2
GOTERM_BP_DIRECT	GO:0002576~platelet degranulation	5	0.413999	A2M, F5, F8, THBS1, TGFB2
GOTERM_CC_DIRECT	GO:0005788~endoplasmic reticulum lumen	7	0.553386	ARSE, ADAMTSL1, F5, F8, THBS1, COL24A1, COL10A1

Annotation Cluster 27 Enrichment Score: 0.48622935964563796

Category	Term	Number of genes in cluster	PValue	Genes
GOTERM_MF_DIRECT	GO:0005085~guanyl-nucleotide exchange factor activity	7	0.166934	DOCK2, RASGRF1, MCF2, RAPGEF5, DOCK8, FGD5, DOCK3
GOTERM_MF_DIRECT	GO:0005089~Rho guanyl-nucleotide exchange factor activity	4	0.436822	ECT2L, RASGRF1, MCF2, FGD5
GOTERM_BP_DIRECT	GO:0035023~regulation of Rho protein signal transduction	4	0.476938	ECT2L, RASGRF1, MCF2, FGD5

Annotation Cluster 28 Enrichment Score: 0.41257280781348343

Category	Term	Number of genes in cluster	PValue	Genes
GOTERM_BP_DIRECT	GO:0050853~B cell receptor signaling pathway	5	0.091817	BLK, RFTN1, PRKCB, SYK, BTK
GOTERM_BP_DIRECT	GO:0038083~peptidyl-tyrosine autophosphorylation	3	0.364029	BLK, SYK, BTK
GOTERM_MF_DIRECT	GO:0004715~non-membrane spanning protein tyrosine kinase activity	3	0.425942	BLK, SYK, BTK
GOTERM_CC_DIRECT	GO:0031234~extrinsic component of cytoplasmic side of plasma membrane	3	0.629419	BLK, SYK, BTK
GOTERM_BP_DIRECT	GO:0045087~innate immune response	9	0.965579	TMEM173, CR2, IL23A, BLK, IRGM, HERC5, TLR4, SYK, BTK

Annotation Cluster 29 Enrichment Score: 0.4105640785221116

Category	Term	Number of genes in cluster	PValue	Genes
KEGG_PATHWAY	hsa05322:Systemic lupus erythematosus	9	0.084203	HIST2H2AA4, GRIN2B, HIST1H2BF, FCGR1A, HIST1H2BG, HIST1H4E, GRIN2A, HIST1H4H, HIST2H3D
GOTERM_CC_DIRECT	GO:0000786~nucleosome	6	0.168945	HIST2H2AA4, HIST1H2BF, HIST1H2BG, HIST1H4E, HIST1H4H, HIST2H3D
KEGG_PATHWAY	hsa05034:Alcoholism	9	0.251042	HIST2H2AA4, GRIN2B, HIST1H2BF, HIST1H2BG, NTRK2, HIST1H4E, GRIN2A, HIST1H4H, HIST2H3D
GOTERM_BP_DIRECT	GO:0006334~nucleosome assembly	6	0.32683	HIST1H2BF, HIST1H2BG, HIST1H4E, SOX9, HIST1H4H, HIST2H3D
GOTERM_BP_DIRECT	GO:0000183~chromatin silencing at rDNA	3	0.329171	HIST1H4E, HIST1H4H, HIST2H3D
GOTERM_BP_DIRECT	GO:0045814~negative regulation of gene expression, epigenetic	3	0.474226	HIST1H4E, HIST1H4H, HIST2H3D

Category	Term	Number of genes in cluster	PValue	Genes
GOTERM_BP_DIRECT	GO:0045815~positive regulation of gene expression, epigenetic	3	0.59003	HIST1H4E, HIST1H4H, HIST2H3D
GOTERM_MF_DIRECT	GO:0046982~protein heterodimerization activity	15	0.602295	EGFR, HIST2H2AA4, BMP2, HIST1H2BF, HIST1H2BG, SOX9, CABYR, TGFB2, HIST2H3D, FOS, BAX, HIST1H4E, RUNX1, NEFL, HIST1H4H
GOTERM_BP_DIRECT	GO:0044267~cellular protein metabolic process	4	0.727852	HIST1H4E, MMP2, HIST1H4H, HIST2H3D
GOTERM_MF_DIRECT	GO:0042393~histone binding	4	0.741656	HIST1H4E, PRKCB, HIST1H4H, HIST2H3D
GOTERM_BP_DIRECT	GO:0031047~gene silencing by RNA	3	0.8716	HIST1H4E, HIST1H4H, HIST2H3D

Annotation Cluster 30 Enrichment Score: 0.4094253842445964

Category	Term	Number of genes in cluster	PValue	Genes
GOTERM_BP_DIRECT	GO:0045740~positive regulation of DNA replication	4	0.147812	EGFR, KITLG, FGF10, GLI2
GOTERM_BP_DIRECT	GO:0046854~phosphatidylinositol phosphorylation	5	0.349002	INPP1, EGFR, KITLG, FGF10, PIP4K2A
GOTERM_BP_DIRECT	GO:0014066~regulation of phosphatidylinositol 3-kinase signaling	4	0.452214	EGFR, KITLG, FGF10, PIP4K2A
GOTERM_MF_DIRECT	GO:0046934~phosphatidylinositol-4,5-bisphosphate 3-kinase activity	3	0.58401	EGFR, KITLG, FGF10
GOTERM_BP_DIRECT	GO:0048015~phosphatidylinositol-mediated signaling	4	0.658523	EGFR, KITLG, FGF10, NGF

Annotation Cluster 31 Enrichment Score: 0.37743257149206066

Category	Term	Number of genes in cluster	PValue	Genes
GOTERM_BP_DIRECT	GO:0048208~COPII vesicle coating	4	0.305713	F5, GRIA1, F8, TGFA
GOTERM_CC_DIRECT	GO:0033116~endoplasmic reticulum-Golgi intermediate compartment membrane	4	0.320867	F5, GRIA1, F8, TGFA
GOTERM_BP_DIRECT	GO:0006888~ER to Golgi vesicle-mediated transport	5	0.751731	ANK1, F5, GRIA1, F8, TGFA

Annotation Cluster 32 Enrichment Score: 0.32608549558010025

Category	Term	Number of genes in cluster	PValue	Genes
KEGG_PATHWAY	hsa05414:Dilated cardiomyopathy	5	0.317239	ADCY8, ITGA11, ITGA1, SGCD, TGFB2
KEGG_PATHWAY	hsa05410:Hypertrophic cardiomyopathy (HCM)	4	0.495678	ITGA11, ITGA1, SGCD, TGFB2
KEGG_PATHWAY	hsa05412:Arrhythmogenic right ventricular cardiomyopathy (ARVC)	3	0.668585	ITGA11, ITGA1, SGCD

Annotation Cluster 33 Enrichment Score: 0.3204903474819103

Category	Term	Number of genes in cluster	PValue	Genes

GOTERM_BP_DIRECT	GO:0070098~chemokine-mediated signaling pathway	4	0.392836	CMKLR1, CCL21, CCL3L3, CXCL12
GOTERM_MF_DIRECT	GO:0008009~chemokine activity	3	0.458031	CCL21, CCL3L3, CXCL12
KEGG_PATHWAY	hsa04062:Chemokine signaling pathway	7	0.607327	DOCK2, ADCY8, CCL21, CCL3L3, PLCB1, CXCL12, AKT3

Annotation Cluster 34	Enrichment Score: 0.3095061728222898			
Category	Term	Number of genes in cluster	PValue	Genes
GOTERM_MF_DIRECT	GO:0042626~ATPase activity, coupled to transmembrane movement of substances	3	0.404006	TAP1, ABCC11, ABCB1
KEGG_PATHWAY	hsa02010:ABC transporters	3	0.442268	TAP1, ABCC11, ABCB1
GOTERM_BP_DIRECT	GO:0055085~transmembrane transport	8	0.659794	SLC6A1, SLC6A2, SLC44A5, TAP1, ABCC11, MFSD4A, ABCB1, SV2C

Annotation Cluster 35	Enrichment Score: 0.23608662991231574			
Category	Term	Number of genes in cluster	PValue	Genes
GOTERM_BP_DIRECT	GO:0043484~regulation of RNA splicing	3	0.234482	RBFOX3, ESRP1, AFF2
GOTERM_BP_DIRECT	GO:0008380~RNA splicing	4	0.901121	RBFOX3, ESRP1, AFF2, NOVA1
GOTERM_BP_DIRECT	GO:0006397~mRNA processing	4	0.926506	CELF6, RBFOX3, ESRP1, AFF2

Annotation Cluster 36	Enrichment Score: 0.03501605743901886			
Category	Term	Number of genes in cluster	PValue	Genes
GOTERM_MF_DIRECT	GO:0001077~transcriptional activator activity, RNA polymerase II core promoter proximal region sequence-specific binding	6	0.867106	EGR1, FOS, EGR2, MAFB, GLI2, SOX9
GOTERM_BP_DIRECT	GO:0006366~transcription from RNA polymerase II promoter	13	0.89392	EGR1, SOX10, ZBTB32, BACH2, EGR2, MAFB, GLI2, SOX9, FOS, SALL4, NFATC4, RUNX1, ETV5
GOTERM_MF_DIRECT	GO:0003682~chromatin binding	8	0.964472	EGFR, SOX10, FOS, SATB2, EGR2, GLI2, SOX9, PRKCB
GOTERM_MF_DIRECT	GO:0000978~RNA polymerase II core promoter proximal region sequence-specific DNA binding	7	0.968891	SOX10, FOS, EGR2, MAFB, RFX3, NFATC4, GLI2

Annotation Cluster 37	Enrichment Score: 0.0057315783242675505			
Category	Term	Number of genes in cluster	PValue	Genes
GOTERM_BP_DIRECT	GO:0098609~cell-cell adhesion	5	0.974509	F11R, FMNL2, TMEM47, UNC5D, EPHA2
GOTERM_CC_DIRECT	GO:0005913~cell-cell adherens junction	5	0.991246	EGFR, F11R, FMNL2, FAT2, EPHA2
GOTERM_MF_DIRECT	GO:0098641~cadherin binding involved in cell-cell adhesion	4	0.995034	EGFR, F11R, FMNL2, EPHA2

GO Clusters-downregulated genes				
Annotation Cluster 1	Enrichment Score: 16.620830591643593			
Category	Term	Number of genes in cluster	PValue	Genes
GOTERM_BP_DIRECT	GO:0007062~sister chromatid cohesion	32	4.94E-23	KIF22, KNTC1, RANGAP1, SPC24, KIF2C, CDCA8, DDX11, INCENP, BUB1, SKA1, CDCA5, ERCC6L, CENPN, CENPL, DSN1, KIF18A, NUF2, CENPF, NDC80, CENPE, CDC20, CENPK, CENPI, CENPH, MAD2L1, SGO2, PLK1, SGO1, KNL1, ZWINT, BUB1B, CENPU
GOTERM_CC_DIRECT	GO:0000777~condensed chromosome kinetochore	24	2.46E-16	CENPN, NEK2, NUF2, KNTC1, NDC80, CENPE, RANGAP1, KNSTRN, CENPK, CENPH, SPC24, KIF2C, MAD2L1, SGO2, SPAG5, SGO1, KNL1, HJURP, INCENP, ZWINT, BUB1, BUB1B, CENPU, ERCC6L
GOTERM_CC_DIRECT	GO:0000776~kinetochore	20	1.13E-12	KIF22, NEK2, KIF18A, TTK, CENPF, NDC80, CENPE, RANGAP1, KNSTRN, CENPI, CENPH, KIF2C, MAD2L1, SGO1, PLK1, SPAG5, INCENP, ZWINT, BUB1, BUB1B
Annotation Cluster 2	Enrichment Score: 8.212847017849692			
Category	Term	Number of genes in cluster	PValue	Genes
GOTERM_BP_DIRECT	GO:0006270~DNA replication initiation	17	1.14E-16	CDC7, CDC6, GINS4, POLE, POLA2, MCM2, MCM3, MCM4, MCM5, MCM6, PRIM1, CCNE2, CDC45, MCM7, POLE2, ORC6, ORC1
GOTERM_BP_DIRECT	GO:0000082~G1/S transition of mitotic cell cycle	24	2.26E-14	CDC7, CDC6, CDK1, DBF4, POLE, IQGAP3, POLA2, MCM2, MCM3, MCM4, CDC25A, MCM5, CDT1, MCM6, PRIM1, CCNE2, CDC45, MCM7, POLE2, RRM2, ORC6, CCNA1, CDCA5, ORC1
KEGG_PATHWAY	hsa03030:DNA replication	14	2.46E-11	POLE, POLA2, MCM2, RNASEH2A, MCM3, MCM4, MCM5, MCM6, PRIM1, RFC4, MCM7, POLE2, POLD1, FEN1
GOTERM_CC_DIRECT	GO:0042555~MCM complex	7	4.34E-08	MCM7, TONSL, MCM2, MCM3, MCM4, MCM5, MCM6
GOTERM_BP_DIRECT	GO:0006268~DNA unwinding involved in DNA replication	5	1.41E-04	MCM7, MCM2, MCM4, TOP2A, MCM6
GOTERM_MF_DIRECT	GO:0003678~DNA helicase activity	6	6.17E-04	MCM7, MCM2, MCM3, MCM4, MCM5, MCM6
GOTERM_CC_DIRECT	GO:0000784~nuclear chromosome, telomeric region	12	0.001356	CDK1, SLX4, MCM7, POLD1, BRCA2, MCM2, MCM3, MCM4, ORC1, MCM5, FEN1, MCM6
Annotation Cluster 3	Enrichment Score: 4.880526517141222			
Category	Term	Number of genes in cluster	PValue	Genes
GOTERM_MF_DIRECT	GO:0008017~microtubule binding	24	6.88E-08	KIF14, KIF23, KIF22, KIFC1, KIF4A, KIF11, PRC1, PSRC1, KIF15, KIF18A, DPYSL5, NUSAP1, KIF18B, CENPE, DPYSL2, RACGAP1, RGS14, REEP4, FAM83D, PLK1, KIF20B, SKA1, JAKMIP2, KIF20A

GOTERM_CC_DIRECT	GO:0005874~microtubule	29	1.08E-07	KIF14, HAUS4, KIF23, KIF22, KIFC1, KIF4A, KIF11, NEK2, KIF15, KIF18A, TPX2, NUSAP1, KIF18B, CENPE, AURKA, DPYSL2, RACGAP1, REEP4, RGS14, KIF2C, TUBB, INCENP, KIF20B, HAUS7, HAUS8, TUBA1B, NEK6, TUBA1C, KIF20A
GOTERM_CC_DIRECT	GO:0005871~kinesin complex	12	2.60E-07	KIF23, KIF14, KIFC1, KIF22, KIF2C, KIF4A, KIF11, KIF18A, KIF20B, KIF18B, CENPE, KIF20A
GOTERM_BP_DIRECT	GO:0007018~microtubule-based movement	14	7.32E-07	KIF14, KIF23, KIF22, KIFC1, KIF4A, KIF11, KIF15, KIF18A, KIF18B, CENPE, RACGAP1, KIF2C, KIF20B, KIF20A
GOTERM_BP_DIRECT	GO:0007080~mitotic metaphase plate congression	10	1.01E-06	KIF14, CCNB1, KIFC1, KIF22, KIF2C, CDCA8, PSRC1, KIF18A, CENPE, CDCA5
GOTERM_MF_DIRECT	GO:0003777~microtubule motor activity	13	4.19E-06	KIF14, KIF23, KIF22, KIFC1, KIF4A, KIF11, KIF15, KIF18A, KIF18B, CENPE, KIF2C, KIF20B, KIF20A
GOTERM_MF_DIRECT	GO:0008574~ATP-dependent microtubule motor activity, plus-end-directed	6	1.07E-04	KIF14, KIF4A, KIF11, KIF18A, KIF20B, KIF18B
GOTERM_BP_DIRECT	GO:0019886~antigen processing and presentation of exogenous peptide antigen via MHC class II	10	0.001665	KIF23, KIF22, KIF2C, KIF4A, KIF11, KIF15, KIF18A, IFI30, CENPE, RACGAP1
GOTERM_BP_DIRECT	GO:0006890~retrograde vesicle-mediated transport, Golgi to ER	9	0.003025	KIF23, KIF22, KIF2C, KIF4A, KIF11, KIF15, KIF18A, CENPE, RACGAP1
GOTERM_MF_DIRECT	GO:0016887~ATPase activity	11	0.048262	KIF23, KIF14, KIFC1, KIF22, KIF2C, BLM, KIF15, KIF20B, CENPE, MYH7, KIF20A

Annotation Cluster 4 Enrichment Score: 2.7666547665325054

Category	Term	Number of genes in cluster	PValue	Genes
KEGG_PATHWAY	hsa00240:Pyrimidine metabolism	12	3.01E-04	NME4, PRIM1, NME2, POLE2, NME1-NME2, POLD1, RRM2, DTYMK, POLE, RRM1, POLA2, TK1
GOTERM_BP_DIRECT	GO:0015949~nucleobase-containing small molecule interconversion	6	7.44E-04	NME4, NME2, NME1-NME2, RRM2, DTYMK, RRM1
KEGG_PATHWAY	hsa00230:Purine metabolism	12	0.022383	NME4, PRIM1, NME2, POLE2, PDE1B, NME1-NME2, PDE1C, POLD1, RRM2, POLE, RRM1, POLA2

Annotation Cluster 5 Enrichment Score: 2.743144728846937

Category	Term	Number of genes in cluster	PValue	Genes
GOTERM_MF_DIRECT	GO:0004674~protein serine/threonine kinase activity	27	6.65E-05	CDK18, NEK2, PASK, TTK, AURKA, ULK4, CHEK2, VRK1, PKN3, BUB1, LMTK3, STKLD1, MAP2K6, CDC7, SRPK3, CDK1, PBK, NME2, PLK4, MAST1, NME1-NME2, MAPK12, PLK1, BUB1B, CIT, MELK, NEK6
GOTERM_MF_DIRECT	GO:0004672~protein kinase activity	22	0.002704	CDC7, SRPK3, CDK1, CDK18, ERBB3, NEK2, PASK, AURKA, PBK, ULK4, CHEK2, VRK1, PKN3, MAPK12, PLK1, BUB1, BUB1B, LMTK3, CIT, MAP2K6, MELK, NEK6

GOTERM_BP_DIRECT	GO:0006468~protein phosphorylation	22	0.032798	SRPK3, CDK18, NEK2, PASK, AURKA, PBK, ULK4, CHEK2, CDC25B, VRK1, PLK4, MAST1, PKN3, PLK1, PTK6, BUB1, BUB1B, LMTK3, WNT11, STKLD1, CIT, NEK6

Annotation Cluster 6	Enrichment Score: 2.6923154739659063			
Category	Term	Number of genes in cluster	PValue	Genes
GOTERM_BP_DIRECT	GO:0000731~DNA synthesis involved in DNA repair	9	6.77E-06	EXO1, XRCC2, RAD51AP1, BLM, POLD1, POLE, BRCA2, BRIP1, RMI2
GOTERM_BP_DIRECT	GO:0000732~strand displacement	7	9.37E-05	EXO1, XRCC2, RAD51AP1, BLM, BRCA2, BRIP1, RMI2
GOTERM_BP_DIRECT	GO:0000724~double-strand break repair via homologous recombination	8	0.006417	SLX4, XRCC2, RAD51AP1, BLM, TONSL, BRCA2, H2AFX, FEN1
KEGG_PATHWAY	hsa03440:Homologous recombination	4	0.061296	XRCC2, BLM, POLD1, BRCA2
GOTERM_BP_DIRECT	GO:0010165~response to X-ray	3	0.138438	XRCC2, BLM, BRCA2

Annotation Cluster 7	Enrichment Score: 2.5540021688177412			
Category	Term	Number of genes in cluster	PValue	Genes
GOTERM_BP_DIRECT	GO:0045926~negative regulation of growth	6	1.89E-04	PTK6, MT1E, MT1X, MT1G, MT1F, IGFBP5
GOTERM_BP_DIRECT	GO:0071294~cellular response to zinc ion	5	0.002109	TSPO, MT1E, MT1X, MT1G, MT1F
KEGG_PATHWAY	hsa04978:Mineral absorption	6	0.011688	CYBRD1, MT1E, ATP1A3, MT1X, MT1G, MT1F
GOTERM_BP_DIRECT	GO:0071276~cellular response to cadmium ion	4	0.013074	MT1E, MT1X, MT1G, MT1F

Annotation Cluster 8	Enrichment Score: 2.149100377731649			
Category	Term	Number of genes in cluster	PValue	Genes
GOTERM_BP_DIRECT	GO:0051225~spindle assembly	6	0.001076	HAUS4, TUBB, NEK2, HAUS7, HAUS8, NEK6
GOTERM_BP_DIRECT	GO:0051297~centrosome organization	5	0.016047	HAUS4, XRCC2, PLK1, HAUS7, HAUS8
GOTERM_CC_DIRECT	GO:0070652~HAUS complex	3	0.02067	HAUS4, HAUS7, HAUS8

Annotation Cluster 9	Enrichment Score: 1.9546850497519228			
Category	Term	Number of genes in cluster	PValue	Genes
GOTERM_CC_DIRECT	GO:0031390~Ctf18 RFC-like complex	4	0.001195	RFC4, DDX11, CHTF18, DSCC1
GOTERM_BP_DIRECT	GO:1900264~positive regulation of DNA-directed DNA polymerase activity	3	0.016825	RFC4, CHTF18, DSCC1
GOTERM_MF_DIRECT	GO:0003689~DNA clamp loader activity	3	0.022107	RFC4, CHTF18, DSCC1
GOTERM_MF_DIRECT	GO:0043142~single-stranded DNA-dependent ATPase activity	3	0.034157	RFC4, CHTF18, DSCC1

Annotation Cluster 10	Enrichment Score: 1.8148560113732715

Category	Term	Number of genes in cluster	PValue	Genes
KEGG_PATHWAY	hsa04114:Oocyte meiosis	13	1.72E-04	CCNB1, CCNE2, CDK1, MAD2L1, CCNB2, PLK1, SGO1, BUB1, AURKA, CDC20, ESPL1, PTTG1, CDC25C
GOTERM_BP_DIRECT	GO:0031145~anaphase-promoting complex-dependent catabolic process	9	0.002392	CCNB1, CDK1, MAD2L1, PLK1, BUB1B, AURKA, CDC20, PTTG1, UBE2C
GOTERM_BP_DIRECT	GO:0051439~regulation of ubiquitin-protein ligase activity involved in mitotic cell cycle	5	0.004385	CCNB1, CDK1, PLK1, CDC20, UBE2C
GOTERM_BP_DIRECT	GO:0042787~protein ubiquitination involved in ubiquitin-dependent protein catabolic process	11	0.016345	CCNB1, CDK1, UHRF1, MAD2L1, RNF165, PLK1, BUB1B, AURKA, CDC20, PTTG1, UBE2C
GOTERM_BP_DIRECT	GO:0051437~positive regulation of ubiquitin-protein ligase activity involved in regulation of mitotic cell cycle transition	7	0.025617	CCNB1, CDK1, MAD2L1, PLK1, BUB1B, CDC20, UBE2C
GOTERM_BP_DIRECT	GO:1904668~positive regulation of ubiquitin protein ligase activity	3	0.027729	PLK1, CDC20, UBE2C
GOTERM_BP_DIRECT	GO:0051436~negative regulation of ubiquitin-protein ligase activity involved in mitotic cell cycle	6	0.060266	CCNB1, CDK1, MAD2L1, BUB1B, CDC20, UBE2C
GOTERM_CC_DIRECT	GO:0005680~anaphase-promoting complex	3	0.141097	BUB1B, CDC20, UBE2C
GOTERM_BP_DIRECT	GO:0043161~proteasome-mediated ubiquitin-dependent protein catabolic process	9	0.260053	KIF14, CDK1, MAD2L1, RNF165, ARRB1, BUB1B, CDC20, UBE2C, GTSE1

Annotation Cluster 11 Enrichment Score: 1.7955617087243398

Category	Term	Number of genes in cluster	PValue	Genes
GOTERM_BP_DIRECT	GO:0000722~telomere maintenance via recombination	8	3.51E-05	PRIM1, RFC4, POLE2, POLD1, POLE, BRCA2, POLA2, FEN1
KEGG_PATHWAY	hsa03410:Base excision repair	5	0.018955	POLE2, POLD1, POLE, NTHL1, FEN1
GOTERM_MF_DIRECT	GO:0003887~DNA-directed DNA polymerase activity	4	0.054458	POLE2, POLD1, POLE, POLA2
GOTERM_BP_DIRECT	GO:0006297~nucleotide-excision repair, DNA gap filling	3	0.159268	RFC4, POLD1, POLE
KEGG_PATHWAY	hsa03420:Nucleotide excision repair	4	0.182497	RFC4, POLE2, POLD1, POLE

Annotation Cluster 12 Enrichment Score: 1.7472168659756713

Category	Term	Number of genes in cluster	PValue	Genes
GOTERM_BP_DIRECT	GO:0051056~regulation of small GTPase mediated signal transduction	12	0.002232	ARHGAP22, ARHGAP33, RAP1GAP, RAC3, SIPA1, DEPDC1B, ARHGAP11A, RACGAP1, ARHGAP8, ECT2, RHOF, NET1
GOTERM_MF_DIRECT	GO:0005096~GTPase activator activity	16	0.020789	RAP1GAP, SIPA1, RANGAP1, DEPDC1, RACGAP1, ECT2, RGS14, ARHGAP22, ARHGAP33, SGSM1, ARRB1, RIN1, WNT11, ARHGAP11A, DEPDC1B, ARHGAP8

GOTERM_BP_DIRECT	GO:0043547~positive regulation of GTPase activity	23	0.123522	FGF18, NRTN, RAP1GAP, ARHGEF39, ERBB3, SIPA1, RANGAP1, DEPDC1, RACGAP1, ECT2, RGS14, ARHGAP22, ARHGAP33, ARRB1, RASGRP2, RIN1, WNT11, RAPGEF3, ARHGAP11A, DEPDC1B, ARHGAP8, EPS8L1, NET1

Annotation Cluster 13 Enrichment Score: 1.521234374612948

Category	Term	Number of genes in cluster	PValue	Genes
GOTERM_BP_DIRECT	GO:0015949~nucleobase-containing small molecule interconversion	6	7.44E-04	NME4, NME2, NME1-NME2, RRM2, DTYMK, RRM1
GOTERM_BP_DIRECT	GO:0006220~pyrimidine nucleotide metabolic process	3	0.027729	NME4, NME2, NME1-NME2
GOTERM_BP_DIRECT	GO:0006163~purine nucleotide metabolic process	3	0.033988	NME4, NME2, NME1-NME2
GOTERM_BP_DIRECT	GO:0006228~UTP biosynthetic process	3	0.047939	NME4, NME2, NME1-NME2
GOTERM_BP_DIRECT	GO:0006241~CTP biosynthetic process	3	0.047939	NME4, NME2, NME1-NME2
GOTERM_BP_DIRECT	GO:0006183~GTP biosynthetic process	3	0.055563	NME4, NME2, NME1-NME2
GOTERM_BP_DIRECT	GO:0009142~nucleoside triphosphate biosynthetic process	3	0.063577	NME4, NME2, NME1-NME2
GOTERM_MF_DIRECT	GO:0004550~nucleoside diphosphate kinase activity	3	0.118806	NME4, NME2, NME1-NME2

Annotation Cluster 14 Enrichment Score: 1.3966796584591972

Category	Term	Number of genes in cluster	PValue	Genes
GOTERM_MF_DIRECT	GO:0004721~phosphoprotein phosphatase activity	7	0.002084	TNS2, PGP, CTDSPL, DLGAP5, CDC25C, CDC25A, CDC25B
GOTERM_BP_DIRECT	GO:0035335~peptidyl-tyrosine dephosphorylation	6	0.172861	EYA1, PGP, PTPN18, CDC25C, CDC25A, CDC25B
GOTERM_MF_DIRECT	GO:0004725~protein tyrosine phosphatase activity	6	0.179199	EYA1, PGP, PTPN18, CDC25C, CDC25A, CDC25B

Annotation Cluster 15 Enrichment Score: 1.3725806825759672

Category	Term	Number of genes in cluster	PValue	Genes
GOTERM_BP_DIRECT	GO:0060048~cardiac muscle contraction	7	0.002056	SCN1B, MYL2, SCN3B, ATP1A3, MYH7, TNNI3, MAP2K6
KEGG_PATHWAY	hsa04260:Cardiac muscle contraction	7	0.029838	MYL2, CACNG6, ATP1A3, MYH7, TNNI3, CACNA2D2, CACNA1S
GOTERM_BP_DIRECT	GO:0055010~ventricular cardiac muscle tissue morphogenesis	3	0.169888	MYL2, MYH7, TNNI3
GOTERM_CC_DIRECT	GO:0030017~sarcomere	3	0.310251	MYL2, MYH7, TNNI3

Annotation Cluster 16 Enrichment Score: 1.1785813825455265

Category	Term	Number of genes in cluster	PValue	Genes
KEGG_PATHWAY	hsa01230:Biosynthesis of amino acids	7	0.024996	SHMT1, MAT1A, ALDOC, PHGDH, ENO3, PSAT1, ASL
KEGG_PATHWAY	hsa01130:Biosynthesis of antibiotics	13	0.034517	NME4, SHMT1, PGP, NME2, ACSS1, LDHA, NME1-NME2, ALDOC, PHGDH, ENO3, PSAT1, ASL, ACAT2

KEGG_PATHWAY	hsa01200:Carbon metabolism	8	0.064347	SHMT1, PGP, ACSS1, ALDOC, PHGDH, ENO3, PSAT1, ACAT2
KEGG_PATHWAY	hsa00260:Glycine, serine and threonine metabolism	3	0.347718	SHMT1, PHGDH, PSAT1

Annotation Cluster 17 Enrichment Score: 1.0227029937424514

Category	Term	Number of genes in cluster	PValue	Genes
GOTERM_BP_DIRECT	GO:0071333~cellular response to glucose stimulus	6	0.0189	NOX4, NME2, NME1-NME2, UCP2, ENDOG, GPER1
GOTERM_CC_DIRECT	GO:0031966~mitochondrial membrane	6	0.134922	NME2, NME1-NME2, UCP2, SLC25A10, GPER1, SLC27A3
GOTERM_BP_DIRECT	GO:0030819~positive regulation of cAMP biosynthetic process	3	0.335233	NME2, NME1-NME2, GPER1

Annotation Cluster 18 Enrichment Score: 0.8766209002503985

Category	Term	Number of genes in cluster	PValue	Genes
GOTERM_BP_DIRECT	GO:0043408~regulation of MAPK cascade	5	0.033013	GDF1, LEFTY2, INHA, ULK4, LEFTY1
GOTERM_BP_DIRECT	GO:0010862~positive regulation of pathway-restricted SMAD protein phosphorylation	5	0.054043	GDF1, LEFTY2, TTK, INHA, LEFTY1
GOTERM_MF_DIRECT	GO:0008083~growth factor activity	10	0.054257	FGF18, NRTN, NTF4, GDF1, PGF, LEFTY2, IGF2, INHA, CSPG5, LEFTY1
GOTERM_BP_DIRECT	GO:0060395~SMAD protein signal transduction	5	0.112952	GDF1, LEFTY2, INHA, ATOH8, LEFTY1
GOTERM_BP_DIRECT	GO:0048468~cell development	4	0.115685	GDF1, LEFTY2, INHA, LEFTY1
GOTERM_MF_DIRECT	GO:0005160~transforming growth factor beta receptor binding	4	0.129623	GDF1, LEFTY2, INHA, LEFTY1
GOTERM_BP_DIRECT	GO:0042981~regulation of apoptotic process	6	0.764116	NME4, GDF1, LEFTY2, ACTN3, INHA, LEFTY1
GOTERM_MF_DIRECT	GO:0005125~cytokine activity	5	0.774708	CMTM1, GDF1, LEFTY2, INHA, LEFTY1

Annotation Cluster 19 Enrichment Score: 0.7607893502903403

Category	Term	Number of genes in cluster	PValue	Genes
KEGG_PATHWAY	hsa04261:Adrenergic signaling in cardiomyocytes	11	0.011293	SCN1B, MYL2, MAPK12, PPP1R1A, CACNG6, ATP1A3, MYH7, RAPGEF3, TNNI3, CACNA2D2, CACNA1S
KEGG_PATHWAY	hsa04260:Cardiac muscle contraction	7	0.029838	MYL2, CACNG6, ATP1A3, MYH7, TNNI3, CACNA2D2, CACNA1S
KEGG_PATHWAY	hsa05410:Hypertrophic cardiomyopathy (HCM)	6	0.097753	DES, MYL2, CACNG6, TNNI3, CACNA2D2, CACNA1S
KEGG_PATHWAY	hsa05414:Dilated cardiomyopathy	6	0.123547	DES, MYL2, CACNG6, TNNI3, CACNA2D2, CACNA1S
GOTERM_BP_DIRECT	GO:0061337~cardiac conduction	4	0.149573	SCN1B, CACNG6, CACNA2D2, CACNA1S
GOTERM_CC_DIRECT	GO:0005891~voltage-gated calcium channel complex	3	0.203033	CACNG6, CACNA2D2, CACNA1S
GOTERM_MF_DIRECT	GO:0005245~voltage-gated calcium channel activity	3	0.336412	CACNG6, CACNA2D2, CACNA1S
KEGG_PATHWAY	hsa05412:Arrhythmogenic right ventricular cardiomyopathy (ARVC)	4	0.351917	DES, CACNG6, CACNA2D2, CACNA1S
GOTERM_BP_DIRECT	GO:0070588~calcium ion transmembrane transport	5	0.474849	TMEM37, CACNG6, ASIC1, CACNA2D2, CACNA1S

GOTERM_BP_DIRECT	GO:0034765~regulation of ion transmembrane transport	4	0.64642	TMEM37, CACNG6, CACNA2D2, CACNA1S
KEGG_PATHWAY	hsa04921:Oxytocin signaling pathway	3	0.952715	CACNG6, CACNA2D2, CACNA1S

Annotation Cluster 20 Enrichment Score: 0.5281674325949556

Category	Term	Number of genes in cluster	PValue	Genes
KEGG_PATHWAY	hsa05204:Chemical carcinogenesis	6	0.106021	GSTT2B, CYP2C8, NAT1, SULT1A2, GSTT2, ALDH3B1
KEGG_PATHWAY	hsa00982:Drug metabolism - cytochrome P450	4	0.360567	GSTT2B, CYP2C8, GSTT2, ALDH3B1
KEGG_PATHWAY	hsa00980:Metabolism of xenobiotics by cytochrome P450	3	0.680958	GSTT2B, GSTT2, ALDH3B1

Annotation Cluster 21 Enrichment Score: 0.4888792424918583

Category	Term	Number of genes in cluster	PValue	Genes
GOTERM_BP_DIRECT	GO:0030705~cytoskeleton-dependent intracellular transport	4	0.015348	KIF14, TUBB, TUBA1B, TUBA1C
GOTERM_BP_DIRECT	GO:0007017~microtubule-based process	4	0.091001	TUBB, TUBA1B, TUBA1C, GTSE1
GOTERM_MF_DIRECT	GO:0005200~structural constituent of cytoskeleton	5	0.417292	TUBB, DES, NDC80, TUBA1B, TUBA1C
KEGG_PATHWAY	hsa05130:Pathogenic Escherichia coli infection	3	0.479313	TUBB, TUBA1B, TUBA1C
KEGG_PATHWAY	hsa04540:Gap junction	4	0.525815	CDK1, TUBB, TUBA1B, TUBA1C
GOTERM_MF_DIRECT	GO:0003924~GTPase activity	8	0.551496	GNG8, TUBB, RAP1GAP, RAC3, ARL4D, RHOF, TUBA1B, TUBA1C
GOTERM_MF_DIRECT	GO:0005198~structural molecule activity	8	0.608801	TUBB, DES, LAMA3, CLDN6, OTOG, TUBA1B, TUBA1C, ADD2
KEGG_PATHWAY	hsa04145:Phagosome	4	0.855923	TUBB, C1R, TUBA1B, TUBA1C
GOTERM_MF_DIRECT	GO:0005525~GTP binding	8	0.943287	TUBB, RAC3, TPX2, RAB26, ARL4D, RHOF, TUBA1B, TUBA1C

Annotation Cluster 22 Enrichment Score: 0.39786956854633326

Category	Term	Number of genes in cluster	PValue	Genes
KEGG_PATHWAY	hsa04610:Complement and coagulation cascades	5	0.170644	F10, C1R, C1S, CFD, PLAUR
KEGG_PATHWAY	hsa05150:Staphylococcus aureus infection	3	0.509667	C1R, C1S, CFD
GOTERM_BP_DIRECT	GO:0006956~complement activation	3	0.736231	C1R, C1S, CFD

Annotation Cluster 23 Enrichment Score: 0.362822736841735

Category	Term	Number of genes in cluster	PValue	Genes
KEGG_PATHWAY	hsa05169:Epstein-Barr virus infection	6	0.337237	MAPK12, PLCG2, CCNA1, CCNA2, MAP2K6, SPN
KEGG_PATHWAY	hsa04664:Fc epsilon RI signaling pathway	4	0.360567	MAPK12, RAC3, PLCG2, MAP2K6
KEGG_PATHWAY	hsa04750:Inflammatory mediator regulation of TRP channels	5	0.370387	MAPK12, ASIC4, PLCG2, ASIC1, MAP2K6
KEGG_PATHWAY	hsa04380:Osteoclast differentiation	4	0.785475	MAPK12, PLCG2, FHL2, MAP2K6

Annotation Cluster 24 Enrichment Score: 0.35152354387628304

Category	Term	Number of genes in cluster	PValue	Genes
GOTERM_BP_DIRECT	GO:0017158~regulation of calcium ion-dependent exocytosis	3	0.279961	C2CD4C, SYT13, SYT7
GOTERM_BP_DIRECT	GO:0048791~calcium ion-regulated exocytosis of neurotransmitter	3	0.324256	C2CD4C, SYT13, SYT7
GOTERM_MF_DIRECT	GO:0030276~clathrin binding	3	0.462615	C2CD4C, SYT13, SYT7
GOTERM_MF_DIRECT	GO:0005544~calcium-dependent phospholipid binding	3	0.520295	C2CD4C, SYT13, SYT7
GOTERM_BP_DIRECT	GO:0006906~vesicle fusion	3	0.528047	C2CD4C, SYT13, SYT7
GOTERM_MF_DIRECT	GO:0019905~syntaxin binding	3	0.674111	C2CD4C, SYT13, SYT7

Annotation Cluster 25 Enrichment Score: 0.22407336122580374

Category	Term	Number of genes in cluster	PValue	Genes
KEGG_PATHWAY	hsa05160:Hepatitis C	6	0.406352	IFIT1, MAPK12, IRF7, CLDN6, TICAM1, IRF1
KEGG_PATHWAY	hsa04620:Toll-like receptor signaling pathway	4	0.65248	MAPK12, IRF7, TICAM1, MAP2K6
KEGG_PATHWAY	hsa05164:Influenza A	5	0.802251	MAPK12, IRF7, TICAM1, KPNA2, MAP2K6

Annotation Cluster 26 Enrichment Score: 0.19555284504211712

Category	Term	Number of genes in cluster	PValue	Genes
GOTERM_BP_DIRECT	GO:0018108~peptidyl-tyrosine phosphorylation	6	0.480486	FGF18, ERBB3, TTK, DDR2, MAP2K6, MELK
GOTERM_MF_DIRECT	GO:0004713~protein tyrosine kinase activity	5	0.564458	FGF18, ERBB3, PTK6, TTK, MAP2K6
GOTERM_BP_DIRECT	GO:0000165~MAPK cascade	5	0.955057	FGF18, NRTN, MAPK12, ERBB3, MAP2K6

Annotation Cluster 27 Enrichment Score: 0.13271303721737404

Category	Term	Number of genes in cluster	PValue	Genes
GOTERM_MF_DIRECT	GO:0020037~heme binding	5	0.587961	NOX4, CYP39A1, FA2H, CYP2C8, FLVCR2
GOTERM_CC_DIRECT	GO:0031090~organelle membrane	3	0.719216	CYP39A1, FA2H, CYP2C8
GOTERM_MF_DIRECT	GO:0005506~iron ion binding	3	0.945482	CYP39A1, FA2H, CYP2C8

Annotation Cluster 28 Enrichment Score: 0.06312509052029312

Category	Term	Number of genes in cluster	PValue	Genes
GOTERM_BP_DIRECT	GO:0098609~cell-cell adhesion	7	0.821367	LDHA, CCNB2, STXBP6, RANGAP1, ANLN, SLC9A3R2, EPS8L1
GOTERM_MF_DIRECT	GO:0098641~cadherin binding involved in cell-cell adhesion	7	0.868008	LDHA, CCNB2, STXBP6, RANGAP1, ANLN, SLC9A3R2, EPS8L1
GOTERM_CC_DIRECT	GO:0005913~cell-cell adherens junction	7	0.906909	LDHA, CCNB2, STXBP6, RANGAP1, ANLN, SLC9A3R2, EPS8L1
