# Supplementary material for: Transcriptomic profiling reveals p53 as a key regulator of doxorubicin-induced cardiotoxicity
Source: Cell Death Discov. 2019 Jun 12;5:102. doi: 10.1038/s41420-019-0182-6 (PMC6561911; doi:10.1038/s41420-019-0182-6)
Supplement: Supplementary file 2 — Supplemental Table 2 [file 41420_2019_182_MOESM2_ESM.pdf]

File edited from: IPA 2000-2019 QIAGEN. All rights reserved. red = upregulated; green = downregulated

Gene Symbol	Entrez Gene Name	Expr p-value	Expr Fold Change	Location	Family
A2M	alpha-2-macroglobulin	0.00141964	3.054487179	Extracellular Space	transporter
ABCB1	ATP binding cassette subfamily B member 1	0.00192435	5.127701375	Plasma Membrane	transporter
ACER2	alkaline ceramidase 2	1.28E-04	8.181818182	Cytoplasm	enzyme
ACTA2	actin, alpha 2, smooth muscle, aorta	0.00601991	2.005571031	Cytoplasm	other
AEN	apoptosis enhancing nuclease	7.12E-06	3.317919075	Nucleus	enzyme
AKR1B1	aldo-keto reductase family 1 member B	0.0093365	2	Cytoplasm	enzyme
AKT3	AKT serine/threonine kinase 3	0.00401076	2.463601533	Cytoplasm	kinase
ANK1	ankyrin 1	3.23E-04	3.849693252	Plasma Membrane	other
ANLN	anillin actin binding protein	0.00714308	-10.26058632	Cytoplasm	other
ANXA4	annexin A4	2.20E-04	2.004008016	Plasma Membrane	other
AQP3	aquaporin 3 (Gill blood group)	0.0267104	2.417142857	Plasma Membrane	transporter
ASF1B	anti-silencing function 1B histone chaperone	0.00370147	-30.17421603	Nucleus	other
ASL	argininosuccinate lyase	0.0372255	-2.059006211	Cytoplasm	enzyme
ASNS	asparagine synthetase (glutamine-hydrolyzing)	0.00548714	-2.02	Cytoplasm	enzyme
ASPM	abnormal spindle microtubule assembly	0.0120816	-51.89189189	Nucleus	other
ASTN2	astrotactin 2	7.54E-06	2.390946502	Cytoplasm	other
AURKA	aurora kinase A	0.0105547	-3.506493506	Nucleus	kinase
BAX	BCL2 associated X, apoptosis regulator	0.00107206	2.076109937	Cytoplasm	transporter
BBC3	BCL2 binding component 3	8.10E-05	3.547120419	Cytoplasm	other
BRCA2	BRCA2, DNA repair associated	0.0314167	-11.65822785	Nucleus	transcription regulator
BTK	Bruton tyrosine kinase	0.0130276	4.009009009	Cytoplasm	kinase
BUB1	BUB1 mitotic checkpoint serine/threonine kinase	0.00588033	-6.140625	Nucleus	kinase
BUB1B	BUB1 mitotic checkpoint serine/threonine kinase B	0.00523514	-8.154761905	Nucleus	kinase
C9orf116	chromosome 9 open reading frame 116	0.0484475	-3.572744015	Nucleus	other
CARHSP1	calcium regulated heat stable protein 1	0.0167394	-2.697247706	Cytoplasm	transcription regulator
CCDC80	coiled-coil domain containing 80	8.82E-04	2.838345865	Nucleus	other
CCL3L3	C-C motif chemokine ligand 3 like 3	0.00242218	8.19112628	Extracellular Space	cytokine
CCNA2	cyclin A2	0.0130085	-16.66666667	Nucleus	other
CCNB1	cyclin B1	0.00754784	-18.62068966	Cytoplasm	kinase
CCNB2	cyclin B2	0.00840825	-70.58823529	Cytoplasm	kinase
CCNE2	cyclin E2	0.0335467	-4.439834025	Nucleus	kinase
CDC20	cell division cycle 20	0.00290144	-41.92546584	Nucleus	other
CDC25A	cell division cycle 25A	0.0010372	-4.363852556	Nucleus	phosphatase
CDC25B	cell division cycle 25B	0.00653231	-3.23364486	Nucleus	phosphatase
CDC25C	cell division cycle 25C	0.0151394	-63.60655738	Nucleus	phosphatase
CDC6	cell division cycle 6	0.0131685	-31.23732252	Nucleus	other
CDC7	cell division cycle 7	0.0275165	-3.901639344	Nucleus	kinase
CDH10	cadherin 10	0.0151303	2.080536913	Plasma Membrane	other
CDK1	cyclin dependent kinase 1	0.0252482	-55.46218487	Nucleus	kinase
CDKN1A	cyclin dependent kinase inhibitor 1A	2.89E-04	4.981273408	Nucleus	kinase
CDT1	chromatin licensing and DNA replication factor 1	0.0251219	-19.43143813	Nucleus	other
CENPF	centromere protein F	0.00655505	-51.00502513	Nucleus	other
CHEK2	checkpoint kinase 2	0.0138613	-3.104395604	Nucleus	kinase
CKB	creatine kinase B	0.00808048	-2.072727273	Cytoplasm	kinase
CKM	creatine kinase, M-type	0.00973552	-3.961165049	Cytoplasm	kinase
CKS1B	CDC28 protein kinase regulatory subunit 1B	0.0108913	-2.955555556	Other	kinase

CXCL12	C-X-C motif chemokine ligand 12	6.57E-04	2.392857143	Extracellular Space	cytokine
DBF4	DBF4 zinc finger	0.0100038	-2.601626016	Nucleus	kinase
DDB2	damage specific DNA binding protein 2	1.60E-05	3.149882904	Nucleus	other
DDIAS	DNA damage induced apoptosis suppressor	0.00758779	-10.04273504	Cytoplasm	other
DGCR6/LOC102724770	DiGeorge syndrome critical region gene 6	0.00850136	2.334782609	Nucleus	other
DHRS9	dehydrogenase/reductase 9	0.00286249	2.783088235	Cytoplasm	enzyme
DLGAP1	DLG associated protein 1	0.034061	2.284710018	Plasma Membrane	other
DLGAP5	DLG associated protein 5	0.00254341	-201.7142857	Nucleus	phosphatase
DRAM1	DNA damage regulated autophagy modulator 1	2.99E-04	4.854166667	Cytoplasm	other
DSN1	DSN1 homolog, MIS12 kinetochore complex component	0.0189401	-5.662576687	Nucleus	other
DUSP2	dual specificity phosphatase 2	3.09E-04	2.110320285	Nucleus	phosphatase
DUSP4	dual specificity phosphatase 4	0.00971846	13.41496599	Nucleus	phosphatase
DUSP6	dual specificity phosphatase 6	0.00208311	6.11969112	Cytoplasm	phosphatase
E2F1	E2F transcription factor 1	0.00974654	-2.546174142	Nucleus	transcription regulator
E2F2	E2F transcription factor 2	0.00489175	-13.57285429	Nucleus	transcription regulator
EDA2R	ectodysplasin A2 receptor	4.75E-07	4.046997389	Plasma Membrane	transmembrane receptor
EGFR	epidermal growth factor receptor	0.00236612	10.53631285	Plasma Membrane	kinase
EGR1	early growth response 1	0.00105578	4.20624152	Nucleus	transcription regulator
EGR2	early growth response 2	5.29E-04	5.341614907	Nucleus	transcription regulator
EPHA2	EPH receptor A2	0.00430095	3.008962868	Plasma Membrane	kinase
ESPL1	extra spindle pole bodies like 1, separase	5.89E-04	-64.37246964	Nucleus	peptidase
EXO1	exonuclease 1	0.0110781	-13.88297872	Nucleus	enzyme
F11R	F11 receptor	7.82E-04	2.163179916	Plasma Membrane	other
F5	coagulation factor V	0.00852964	2.168734491	Extracellular Space	other
FAM83D	family with sequence similarity 83 member D	0.00376028	-31.40495868	Cytoplasm	other
FANCI	FA complementation group I	0.00430697	-3.989361702	Nucleus	other
FAS	Fas cell surface death receptor	6.81E-07	4.581589958	Plasma Membrane	transmembrane receptor
FAT2	FAT atypical cadherin 2	0.0174073	2.055921053	Plasma Membrane	other
FBLN2	fibulin 2	0.02406	3.968911917	Extracellular Space	other
FBXO22	F-box protein 22	4.76E-04	2.155525239	Cytoplasm	enzyme
FDXR	ferredoxin reductase	9.61E-04	4.787644788	Cytoplasm	enzyme
FEN1	flap structure-specific endonuclease 1	0.00718401	-3.502304147	Nucleus	enzyme
FHL2	four and a half LIM domains 2	0.0343088	-2.658959538	Nucleus	transcription regulator
FOS	Fos proto-oncogene, AP-1 transcription factor subunit	0.0044377	2.894308943	Nucleus	transcription regulator
FOXM1	forkhead box M1	0.00461357	-10.0270636	Nucleus	transcription regulator
GADD45A	growth arrest and DNA damage inducible alpha	1.71E-05	3.214574899	Nucleus	other
GAL3ST4	galactose-3-O-sulfotransferase 4	8.65E-05	6.323185012	Cytoplasm	enzyme
GDF15	growth differentiation factor 15	3.48E-04	13.44086022	Extracellular Space	growth factor
GLS2	glutaminase 2	1.91E-05	4.123337364	Cytoplasm	enzyme
GTSE1	G2 and S-phase expressed 1	0.00278751	-22.75541796	Cytoplasm	other
H2AFX	H2A histone family member X	2.28E-04	-3.573700954	Nucleus	transcription regulator
HAS2	hyaluronan synthase 2	0.0495063	2.488095238	Plasma Membrane	enzyme
HERC5	HECT and RLD domain containing E3 ubiquitin protein ligase 5	0.0170015	3.944134078	Cytoplasm	enzyme
HJURP	Holliday junction recognition protein	0.00278347	-100.7058824	Nucleus	other
HMGB2	high mobility group box 2	0.0124956	-2.514450867	Nucleus	transcription regulator
HMMR	hyaluronan mediated motility receptor	0.0111643	-14.7826087	Plasma Membrane	transmembrane receptor
HS3ST1	heparan sulfate-glucosamine 3-sulfotransferase 1	0.00101633	3.058823529	Cytoplasm	enzyme
HSPA4L	heat shock protein family A (Hsp70) member 4 like	4.04E-04	2.37254902	Cytoplasm	other

IER5	immediate early response 5	0.00819775	2.156626506	Other	other
IFI30	IFI30, lysosomal thiol reductase	0.0130207	-2.63372093	Cytoplasm	enzyme
IGF2	insulin like growth factor 2	0.00153307	-2.82122905	Extracellular Space	growth factor
IGFBP5	insulin like growth factor binding protein 5	0.00165825	-5.118483412	Extracellular Space	other
IL31RA	interleukin 31 receptor A	0.0130339	2.816901408	Plasma Membrane	transmembrane receptor
IRF7	interferon regulatory factor 7	0.0491917	-4.62915601	Nucleus	transcription regulator
KIF23	kinesin family member 23	0.00500203	-10.62068966	Cytoplasm	other
KIFC1	kinesin family member C1	0.00264503	-52.25490196	Nucleus	enzyme
KITLG	KIT ligand	5.08E-05	3.379888268	Extracellular Space	growth factor
KLLN	killin, p53 regulated DNA replication inhibitor	0.00630113	3.190661479	Nucleus	other
KNTC1	kinetochore associated 1	0.0426994	-3.30952381	Nucleus	other
KPNA2	karyopherin subunit alpha 2	0.0141063	-3.768115942	Nucleus	transporter
LDHA	lactate dehydrogenase A	0.00794677	-2.736842105	Cytoplasm	enzyme
LIF	LIF, interleukin 6 family cytokine	3.23E-05	3.762886598	Extracellular Space	cytokine
LOC102724788/PRODH	proline dehydrogenase 1	6.26E-04	2.703125	Cytoplasm	enzyme
LRRC3	leucine rich repeat containing 3	0.0206211	-2.002298851	Other	other
MAD2L1	mitotic arrest deficient 2 like 1	0.026145	-9.553072626	Nucleus	other
MAFB	MAF bZIP transcription factor B	0.0144527	3.17373461	Nucleus	transcription regulator
MAP2K6	mitogen-activated protein kinase kinase 6	0.00411055	-2.5	Cytoplasm	kinase
MAPK12	mitogen-activated protein kinase 12	0.0113823	-2.237864078	Cytoplasm	kinase
MCAM	melanoma cell adhesion molecule	0.0201619	-3.926605505	Plasma Membrane	other
MCM2	minichromosome maintenance complex component 2	0.00213631	-5.59047619	Nucleus	enzyme
MCM3	minichromosome maintenance complex component 3	0.00920864	-2.467532468	Nucleus	enzyme
MCM4	minichromosome maintenance complex component 4	0.028027	-2.123417722	Nucleus	enzyme
MCM5	minichromosome maintenance complex component 5	0.00394144	-28.25870647	Nucleus	enzyme
MCM6	minichromosome maintenance complex component 6	0.00868276	-8.254545455	Nucleus	enzyme
MCM7	minichromosome maintenance complex component 7	4.89E-04	-2.231343284	Nucleus	enzyme
MDM2	MDM2 proto-oncogene	4.58E-04	2.766666667	Nucleus	transcription regulator
MELK	maternal embryonic leucine zipper kinase	0.0123196	-14.4375	Cytoplasm	kinase
MIS18BP1	MIS18 binding protein 1	0.0399309	-2.166666667	Nucleus	transcription regulator
MKI67	marker of proliferation Ki-67	0.00283873	-245.0292398	Nucleus	other
MMP2	matrix metallopeptidase 2	0.00742883	2.244019139	Extracellular Space	peptidase
MMP23B	matrix metallopeptidase 23B	0.0360959	-8.319783198	Extracellular Space	peptidase
MYBL2	MYB proto-oncogene like 2	0.00830455	-139.8230088	Nucleus	transcription regulator
MYO10	myosin X	0.00379313	2.770053476	Cytoplasm	enzyme
MYOF	myoferlin	0.00100643	2.354285714	Nucleus	other
NCAPG	non-SMC condensin I complex subunit G	0.0209006	-13.04733728	Nucleus	other
NCAPH	non-SMC condensin I complex subunit H	0.00542395	-33.61884368	Nucleus	other
NDC80	NDC80, kinetochore complex component	0.0128661	-88.96103896	Nucleus	other
NEK2	NIMA related kinase 2	0.00883423	-111.328125	Cytoplasm	kinase
NFE2	nuclear factor, erythroid 2	0.00893332	-2.043859649	Nucleus	transcription regulator
NKD1	naked cuticle homolog 1	0.00423821	-3.751743375	Other	other
NOX4	NADPH oxidase 4	0.00754481	-3.352941176	Cytoplasm	enzyme
NUPR1	nuclear protein 1, transcriptional regulator	0.0116606	-19.21921922	Nucleus	transcription regulator
NUSAP1	nucleolar and spindle associated protein 1	0.0164967	-52.19298246	Nucleus	other
PBK	PDZ binding kinase	0.00680232	-141.4376321	Cytoplasm	kinase
PCNX2	pecanex 2	0.00516355	2.330120482	Other	other
PDE4B	phosphodiesterase 4B	0.00384409	2.502628812	Cytoplasm	enzyme

PEG10	paternally expressed 10	0.0100603	-2.933333333	Nucleus	other
PHGDH	phosphoglycerate dehydrogenase	0.00344725	-6.156351792	Cytoplasm	enzyme
PHLDA1	pleckstrin homology like domain family A member 1	0.00150513	4.792	Cytoplasm	other
PLAU	plasminogen activator, urokinase	0.0105023	2.396606575	Extracellular Space	peptidase
PLAUR	plasminogen activator, urokinase receptor	0.00386362	-4.221508828	Plasma Membrane	transmembrane receptor
PLK1	polo like kinase 1	0.00181638	-14.86725664	Nucleus	kinase
PLK2	polo like kinase 2	3.21E-04	3.455210238	Nucleus	kinase
PMAIP1	phorbol-12-myristate-13-acetate-induced protein 1	7.28E-04	5.775749674	Cytoplasm	other
POLD1	DNA polymerase delta 1, catalytic subunit	4.91E-05	-3.269230769	Nucleus	enzyme
POLE2	DNA polymerase epsilon 2, accessory subunit	0.0338564	-12.95597484	Nucleus	enzyme
PRC1	protein regulator of cytokinesis 1	0.00172952	-18.75	Nucleus	other
PRIM1	DNA primase subunit 1	0.0108452	-9.655172414	Nucleus	enzyme
PRKCB	protein kinase C beta	0.00363713	2.713815789	Cytoplasm	kinase
PROM1	prominin 1	0.00456201	2.842105263	Plasma Membrane	other
PSRC1	proline and serine rich coiled-coil 1	9.80E-04	-22.14411248	Cytoplasm	other
PTCHD4	patched domain containing 4	8.82E-04	2.75625	Other	other
PTP4A1	protein tyrosine phosphatase type IVA, member 1	8.64E-04	2.164251208	Cytoplasm	phosphatase
PTTG1	pituitary tumor-transforming 1	0.0216513	-15.80487805	Nucleus	transcription regulator
PVT1	Pvt1 oncogene	0.00260189	2.319018405	Other	other
RACGAP1	Rac GTPase activating protein 1	0.00560653	-11.90265487	Cytoplasm	transporter
RAD51AP1	RAD51 associated protein 1	0.0322573	-54.18502203	Nucleus	other
RANGAP1	Ran GTPase activating protein 1	6.33E-04	-2.076086957	Nucleus	other
RAP2B	RAP2B, member of RAS oncogene family	2.28E-04	3.306666667	Plasma Membrane	enzyme
RBL1	RB transcriptional corepressor like 1	0.0380349	-2.311827957	Nucleus	transcription regulator
RECQL4	RecQ like helicase 4	0.00439777	-4.194915254	Nucleus	enzyme
RFC4	replication factor C subunit 4	0.0165023	-2.94375	Nucleus	other
RNF144B	ring finger protein 144B	0.00397118	2.65408805	Cytoplasm	enzyme
RPS27L	ribosomal protein S27 like	0.00878662	2.300518135	Cytoplasm	translation regulator
RRM1	ribonucleotide reductase catalytic subunit M1	0.02316	-2.546689304	Nucleus	enzyme
RRM2	ribonucleotide reductase regulatory subunit M2	0.00614278	-26.82432432	Nucleus	enzyme
RRM2B	ribonucleotide reductase regulatory TP53 inducible subunit M2B	1.23E-04	3.944636678	Nucleus	enzyme
RUNX1	runt related transcription factor 1	0.0493644	2.375	Nucleus	transcription regulator
S100A2	S100 calcium binding protein A2	0.0371298	-3.857677903	Nucleus	other
S100A4	S100 calcium binding protein A4	0.0218878	-4.841269841	Cytoplasm	other
SCN3B	sodium voltage-gated channel beta subunit 3	0.00326762	-3.414965986	Plasma Membrane	ion channel
SERPINE2	serpin family E member 2	0.00552022	2.496183206	Extracellular Space	other
SESN1	sestrin 1	1.03E-05	2.739018088	Nucleus	other
SESN2	sestrin 2	0.00517476	2.526143791	Cytoplasm	enzyme
SGK1	serum/glucocorticoid regulated kinase 1	0.00385626	4.245742092	Cytoplasm	kinase
SLC6A6	solute carrier family 6 member 6	1.20E-04	7.896103896	Plasma Membrane	transporter
SMC2	structural maintenance of chromosomes 2	0.0243922	-2.471264368	Nucleus	transporter
SMC4	structural maintenance of chromosomes 4	0.00503118	-3.182640145	Nucleus	transporter
SPATA18	spermatogenesis associated 18	1.34E-05	3.323139653	Cytoplasm	other
SPN	sialophorin	5.24E-05	-2.28440367	Plasma Membrane	transmembrane receptor
SULF2	sulfatase 2	9.51E-04	7.022900763	Plasma Membrane	enzyme
TAP1	transporter 1, ATP binding cassette subfamily B member	0.00370336	2.81300813	Cytoplasm	transporter
TFPI2	tissue factor pathway inhibitor 2	0.00166328	2.307692308	Extracellular Space	other
TGFA	transforming growth factor alpha	0.0236514	2.042918455	Extracellular Space	growth factor

TGFB1I1	transforming growth factor beta 1 induced transcript 1	0.0165039	-3.11	Nucleus	transcription regulator
TGFB2	transforming growth factor beta 2	0.00215244	2.664634146	Extracellular Space	growth factor
THBS1	thrombospondin 1	0.0498598	2.580195258	Extracellular Space	other
TIGAR	TP53 induced glycolysis regulatory phosphatase	2.59E-06	2.928994083	Cytoplasm	enzyme
TMEM97	transmembrane protein 97	0.0044465	-2.436213992	Extracellular Space	other
TMSB15A	thymosin beta 15a	0.0193419	-10.80188679	Cytoplasm	other
TNFRSF10A	TNF receptor superfamily member 10a	5.12E-04	11.19453925	Plasma Membrane	transmembrane receptor
TNFRSF10B	TNF receptor superfamily member 10b	3.18E-04	2.384937238	Plasma Membrane	transmembrane receptor
TNFRSF10C	TNF receptor superfamily member 10c	3.40E-04	5.76433121	Plasma Membrane	transmembrane receptor
TNFRSF10D	TNF receptor superfamily member 10d	5.80E-05	3.086592179	Plasma Membrane	transmembrane receptor
TNFSF9	TNF superfamily member 9	0.00153167	3.112582781	Plasma Membrane	cytokine
TOP2A	DNA topoisomerase II alpha	0.0449281	-95.53956835	Nucleus	enzyme
TP53	tumor protein p53	N/A	unchanged	Nucleus	transcription regulator
TP53INP1	tumor protein p53 inducible nuclear protein 1	9.95E-04	2.352231604	Nucleus	other
TPX2	TPX2, microtubule nucleation factor	0.00138625	-55.98290598	Nucleus	other
TRIM22	tripartite motif containing 22	9.39E-06	8.175675676	Cytoplasm	transcription regulator
TSPO	translocator protein	0.0277454	-2.60371517	Cytoplasm	transmembrane receptor
TTK	TTK protein kinase	0.0201271	-57.74647887	Nucleus	kinase
TUBB	tubulin beta class I	5.41E-04	-2.057692308	Cytoplasm	other
UBE2C	ubiquitin conjugating enzyme E2 C	0.00442982	-145.2830189	Cytoplasm	enzyme
UBE2T	ubiquitin conjugating enzyme E2 T	0.0118651	-5.534883721	Nucleus	enzyme
UHRF1	ubiquitin like with PHD and ring finger domains 1	0.0131418	-46.11260054	Nucleus	transcription regulator
UPP1	uridine phosphorylase 1	0.0370243	2.283737024	Cytoplasm	enzyme
VRK1	vaccinia related kinase 1	0.0229266	-2.656716418	Nucleus	kinase
VWCE	von Willebrand factor C and EGF domains	0.00141958	2.956043956	Cytoplasm	other
WDR63	WD repeat domain 63	4.23E-04	5.996503497	Other	other
XPC	XPC complex subunit, DNA damage recognition and repair factor	3.94E-05	3.572368421	Nucleus	other
ZMAT3	zinc finger matrin-type 3	6.69E-04	2.434052758	Nucleus	other
