# Supplementary material for: Transcriptomic profiling reveals p53 as a key regulator of doxorubicin-induced cardiotoxicity
Source: Cell Death Discov. 2019 Jun 12;5:102. doi: 10.1038/s41420-019-0182-6 (PMC6561911; doi:10.1038/s41420-019-0182-6)
Supplement: Supplementary file 3 — Supplemental Table 3 [file 41420_2019_182_MOESM3_ESM.pdf]

All 315 dysreg. genes on day 14										
			Log 2 values per sample: red = upregulated; green = downregulated							
**Gene Name**	**P-Value**	**Fold Change**	**dox-day14-a**	**dox-day14-b**	**dox-day14-c**	**dox-day14-d**	**control-day14-a**	**control-day14-b**	**control-day17-c**	**control-day14-d**
ABCA12	0.001636	7.215619694	-5.189680297	-4.721658341	-4.097887821	-4.429731384	-7.301301444	-6.615287038	-7.898834041	-8.454822365
ABRA	0.047898	2.3125	-0.710755715	0.454175893	-0.680382066	-0.316528107	-2.625934282	-1.011587974	-1.011587974	-1.662003536
ACBD7	0.00433	0.442982456	-2.465938398	-3.580353247	-4.290532899	-3.490050854	-2.145605322	-2.017417053	-2.210896782	-2.152003093
ACSM5	0.010236	0.469811321	-4.348133165	-4.054092703	-5.64385619	-3.838563734	-2.878321443	-3.592484088	-3.307572802	-3.23786383
ACSS1	0.034292	0.341441441	-0.749038426	-2.490050854	-1.307572802	-1.560642822	-0.281035664	-0.880975897	0.659924558	0.584962501
ACTN3	0.034906	0.320134228	0.321928095	-2.244685096	-2.442222329	-1.910501849	1.15704371	0.613531653	0.201633861	0.056583528
ADAMTS10	0.014629	0.391213389	1.411426246	-0.846843212	0.782408565	1.350497247	2.166715445	1.5360529	2.541019153	2.56315813
ADCY3	0.036411	0.469262295	2.114367025	0.50589093	0.650764559	0.86393845	2.737686761	2.361768359	2.073820233	1.815575429
AHRR	0.0162	2.171717172	-3.385337265	-2.590744853	-3.293358943	-2.736965594	-3.647467443	-3.662003536	-4.702749879	-4.629500897
AKR1C1	0.006093	0.40530303	-1.340075442	-3.397448103	-2.224317298	-2.708396442	-0.951763814	-0.857259828	-1.224317298	-0.698997744
APLP1	0.007161	2.247933884	4.378511623	4.626439137	4.744161096	5.185866545	2.9202933	4.044394119	3.548436625	3.632268215
APOLD1	0.018266	0.322274882	-0.910501849	-3.970299766	-3.293358943	-3.083141235	-0.239566125	-0.659722595	-0.918660373	-0.932361283
ARHGAP11A	0.044656	0.285169492	1.042644337	-1.965784285	-2.727379545	-2.13289427	1.89917563	1.195347598	0.839959587	0.704871964
ARHGEF34P	0.01727	0.272033898	-7.74545433	-7.848089242	-9.923139947	-8.475214154	-6.41734766	-5.733123528	-7.04930764	-6.787866492
ARHGEF38	0.031963	2.914438503	-4.895394957	-3.884700356	-4.908334012	-3.580353247	-6.265344567	-5.356975042	-5.993091631	-5.519528055
ARHGEF5	0.001711	0.329376855	-6.179187923	-5.938984225	-7.748553568	-6.705758629	-5.205563338	-4.53951953	-4.934565554	-4.970299766
ARMC4	0.031149	0.313029316	-2.321928095	-2.634867407	-5.853084152	-7.189680297	-1.187707155	-1.522840789	-1.915935735	-2.522840789
ARMC4P1	0.035691	3.176271186	-7.39775218	-5.956795501	-7.258701293	-6.807124109	-9.142034924	-8.454822365	-7.965784285	-8.292227861
ARNTL2	0.015764	0.450549451	0.963474124	-0.886299501	-0.390245038	0.722466024	1.739848103	1	1.333423734	1.608809243
ART4	0.012244	2.705882353	-6.667125969	-5.983931631	-5.702749879	-5.687799537	-6.821737915	-8.25208847	-6.761017534	-8.673002535
ASGR1	0.00765	0.447826087	-0.13606155	-1.603840511	-1.15521265	-0.347398782	0.298658316	0.411426246	0.82374936	0.23878686
ASPM	0.022675	0.197115385	-0.461958547	-5.51292532	-5.587272661	-4.273691909	0.739848103	-0.110915901	-0.334607229	-0.371459681
ATP13A4	0.02627	2.423076923	-3.083141235	-2.442222329	-3.553002759	-3.120294234	-4.695255342	-3.428177593	-4.377819296	-5.13796526
ATP2B2	0.014973	2.716981132	-0.378944497	0.35614381	0.925999419	0.839959587	-1.486004021	-0.588573754	-0.74178261	-1
ATP8B3	0.046848	0.312384473	-0.962969269	-5.553002759	-5.573466862	-3.035046947	-0.498178735	-0.715485867	-1.841662973	-0.81857936
AURKB	0.012952	0.208252427	4.017921908	-2.795859283	-1.214240226	-1.430508908	4.705977902	3.887525271	4.498250868	4.232660757
BBC3	0.017625	2.008733624	4.240314329	4.439623138	4.137503524	3.944858446	2.448900951	4.053111336	2.881664619	2.839959587
BEST3	0.027524	2.094276094	-1.15521265	-0.246395464	-1.261880711	-0.356605547	-1.612637459	-1.675765438	-1.662003536	-2.10159814
BORA	0.005639	0.45	0.014355293	-1.354759487	-1.321928095	-0.717856771	0.464668267	0.097610797	0.604071324	0.464668267
BRIP1	0.028297	0.229565217	2.09085343	-2.070966521	-1.816037165	-0.991369695	3.212569339	2.336283388	2.137503524	2.09085343
BUB1	0.032477	0.317829457	2.500802053	-0.19759996	-0.121863233	-0.380821784	3.23572706	2.66448284	2.333423734	2.321928095
BUB1B	0.029546	0.30516129	0.344828497	-2.23786383	-2.336427665	-2.244685096	1.207892852	0.59454855	0.275007047	0.189033824
C2CD4C	0.01708	0.400483092	-2.878321443	-5.836501268	-5.020925839	-2.76611194	-2.224317298	-2.564904848	-2.351074441	-2.017417053
C5orf34	0.028773	0.352727273	-0.010134377	-2.857259828	-2.490050854	-2.035046947	0.575312331	0.150559677	-0.03209363	-0.291592017
CCDC150	0.042782	0.372703412	0.790772038	-0.058893689	-0.229382353	1.111031312	1.555816155	0.831877241	2.310340121	2.475084883
CCDC74A	0.009932	0.415073116	2.843983844	0.831877241	1.286881148	1.757023247	3.181102551	2.817623258	3.158660175	3.392317423
CCDC74B	0.005314	0.345454545	0.555816155	-2.035046947	-1.074000581	-0.524915117	1.150559677	0.704871964	1.03562391	1.316145742
CCKBR	0.027755	0.303703704	-3.625934282	-5.351074441	-6.731589562	-5.938984225	-4.368849142	-2.867752202	-2.954557029	-3.083141235

CCNA1	0.011782	0.255014327	-1.369594529	-6.168771307	-3.13289427	-2.351074441	-0.330973234	-1.49410907	-0.110915901	-0.483984853
CCNB1	0.013121	0.274755927	2.596935142	-0.717856771	-0.550042516	-0.940644722	3.147306699	2.839959587	2.724650272	2.592158002
CCNB2	0.021092	0.227027027	0.687060688	-6.41734766	-5.044538396	-5.24331826	1.316145742	0.941106311	0.659924558	0.50589093
CCNF	0.040209	0.439534884	-0.424687669	-2.671163536	-1.486004021	-1.994240731	0.286881148	-0.227692025	-0.397828209	-0.734563104
CDC20	0.009367	0.231553398	0.722466024	-4.078259014	-3.3703403	-3.307572802	1.327687364	0.925999419	1.014355293	0.86393845
CDC25B	0.00331	0.406349206	3.817623258	2.526068812	2.372952098	2.50589093	4.392317423	4	4.285402219	4.255500733
CDC25C	0.016015	0.243362832	1.956056652	-1.910501849	-3.011587974	-2.795859283	2.456806149	1.782408565	2.298658316	2.077242999
CDCA3	0.005068	0.286166843	2.754887502	0.163498732	0.367371066	0.765534746	3.572889668	3.117695043	3.114367025	3.116031993
CDH24	0.018553	0.367593712	-1.461958547	-4.073393259	-1.680382066	-1.049904906	-0.471928835	-1.020340448	0	0.124328135
CDH5	0.003773	0.368879217	-4.570035956	-6.221623189	-4.461163892	-4.853084152	-3.323371512	-3.693387776	-3.083141235	-3.779917739
CDKN1A	0.008464	2.13877551	9.063395081	9.233619677	8.912889336	8.891783703	7.312882955	8.787902559	7.636624621	7.499845887
CDKN2D	0.002729	0.402173913	0.925999419	-0.897006007	-0.01887801	0.028569152	1.646162657	1.22650853	1.575312331	1.372952098
CDKN3	0.022023	0.283018868	2.430285273	-1.552156356	-1.275786313	-1.187707155	2.771885579	2.733354341	2.185866545	2.403267722
CENPA	0.005223	0.177470776	-0.736965594	-5.24879339	-6.759453637	-4.878321443	0.22650853	-0.057391664	-0.637109357	-0.008682243
CENPE	0.019407	0.232342007	1.077242999	-2.888968688	-2.932361283	-3.035046947	1.974529312	1.14404637	1.361768359	1.042644337
CENPF	0.013471	0.204137931	1.761285273	-4.938984225	-4.542878542	-3.558091636	2.580145484	1.959770155	1.970853654	1.843983844
CENPI	0.009946	0.325888325	0.495695163	-1.831357964	-1.286304185	-1.095419565	1.389566812	0.545968369	1.03562391	0.815575429
CENPW	0.01443	0.305454545	0.799087306	-1.621488377	-3.826232932	-0.867752202	1.541019153	1.124328135	0.765534746	1.014355293
CHI3L1	0.042591	2.875960483	-5.310432456	-4.676687582	-5.756330919	-5.49980982	-8.051219761	-7.368849142	-8.459893355	-5.392894616
CHTF18	0.015669	0.435294118	2.765534746	1.650764559	1.575312331	2.961623328	3.485426827	2.950468414	3.867896464	3.837943242
CIP2A	0.004073	0.326530612	0.555816155	-0.926865295	-1.139235797	-0.411195433	1.700439718	0.98550043	1.263034406	1.10433666
CKS2	0.042089	0.423404255	2.247927513	0.056583528	-0.227692025	0.422233001	2.488000771	2.464668267	1.918386234	1.956056652
CLCNKB	0.009467	0.440677966	1	-0.77349147	-0.935117148	0.084064265	1.111031312	1.201633861	1.327687364	1.298658316
CLDN6	0.02028	0.357377049	1.956056652	-0.030619235	0.731183242	1.14404637	2.214124805	2.042644337	2.759155834	3.145677455
COL10A1	0.010926	2.446428571	-2.286304185	-1.59946207	-1.53951953	-2.19759996	-2.846843212	-2.556393349	-3.671163536	-4.023269779
COL15A1	0.008126	0.431851852	-4.221623189	-3.963531833	-4.290532899	-3.956795501	-2.388355457	-3.293358943	-3.083141235	-2.954557029
CPXM1	0.003671	0.251184834	-0.775959726	-4.276485124	-3.983931631	-2.746615764	0	-0.242976753	-0.373327247	-0.392137097
CYP21A1P	0.006174	0.461139896	-1.354759487	-1.926865295	-1.526992432	-1.241270432	-0.330973234	-1.005782353	-0.140825544	-0.168122759
CYP21A2	0.011604	0.468627451	-0.798366139	-1.708396442	-1.10780329	-0.821126042	0.084064265	-0.689659879	0.298658316	0.250961574
CYP4F29P	0.033642	0.495726496	-2.10159814	-3.828280761	-4.140507455	-3.251538767	-1.910501849	-2.351074441	-2.152003093	-2.005782353
DBIL5P2	0.005128	0.434782609	-6.753214946	-5.210896782	-6.615287038	-5.836501268	-4.820106829	-5.054092703	-4.618827395	-4.601211852
DDIAS	0.025887	0.261682243	-0.444183845	-4.392894616	-3.332062472	-2.058893689	0.731183242	0.189033824	-0.375197235	-0.496142467
DDX11	0.007771	0.439175258	4.963474124	3.807354922	3.459431619	4.852997588	5.882643049	5.181897643	5.64096791	5.614709844
DDX12P	0.011265	0.390862944	0.150559677	-1.061902439	-1.347398782	0.137503524	1.077242999	0.22650853	1.176322773	1.22650853
DENND2A	0.014371	0.429565217	0.604071324	-1.098505545	-0.444183845	0.298658316	1.03562391	0.678071905	1.584962501	1.350497247
DEPDC7	0.016658	0.484320557	-2.231074664	-3.448508591	-3.145605322	-2.857259828	-1.948975997	-2.336427665	-1.477944251	-1.586405918
DERL3	0.013781	0.466666667	2.017921908	-0.921390165	1	1.40599236	2.472487771	2.182692298	2.207892852	2.350497247
DINOL	0.000375	2.286501377	-0.407363571	-0.089267338	-0.506352666	-0.121863233	-1.756330919	-1.395928676	-1.220950447	-1.526992432
DMRT2	0.044447	0.285280728	-1.694321257	-2.531156057	-4.299027693	-2.177881725	-1.652901329	-0.888968688	0.214124805	-0.687334826
DMRTC1	0.045515	0.276811594	-3.158429363	-5.594225422	-6.392894616	-6.94608237	-2.204233052	-2.717856771	-3.735043282	-3.23786383
DMRTC1B	0.039735	0.285384615	-3.224317298	-5.411195433	-6.221623189	-7.767290131	-2.351074441	-2.816037165	-3.797864419	-3.19759996

DNAI1	0.026283	0.470046083	-2.411195433	-4.787866492	-3.604717796	-3.307572802	-1.988504361	-2.756330919	-2.058893689	-2.126580497
DNAJC22	0.025989	0.424137931	-2.652901329	-7.834853415	-2.329159664	-2.977099598	-1.708396442	-2.23786383	-1.98279071	-1.358453971
DNALI1	0.004848	0.474285714	0.516015147	-0.567040593	-0.543719518	0.250961574	1.049630768	0.713695815	1.286881148	1.176322773
DPF1	0.00034	0.294805195	-0.413115187	-2.59946207	-1.300448367	-1.011587974	0.475084883	0.485426827	0.713695815	0.790772038
DPYSL5	0.037917	0.428571429	-1.061902439	-3.568323559	-2.573466862	-2.481968507	-0.465938398	-0.790858602	-1.184424571	-1.369594529
E2F1	0.040238	0.332386364	1.526068812	-0.81857936	-1.19759996	-0.336427665	2.464668267	1.744161096	1.510961919	1.23878686
ELOA3	0.035986	4.71257485	-4.904008087	-2.826232932	-3.633076351	-4.063710705	-5.733123528	-6.380821784	-5.467533417	-6.210896782
ELOA3B	0.021654	5.3515625	-5.006941609	-3.095419565	-3.869859865	-4.117787378	-5.861447625	-6.601211852	-5.956795501	-7.015315871
ELOA3C	0.037945	4.233695652	-5.006941609	-2.910501849	-3.481968507	-4.117787378	-5.276485124	-6.601211852	-5.276485124	-6.429731384
ERFE	0.008594	0.337552743	-4.952322025	-6.321928095	-5.227016448	-5.362899876	-4.368849142	-4.202903992	-3.402016006	-3.521183471
ERICH5	0.044236	0.422794118	-0.612637459	-3.307572802	-0.354759487	-0.394031641	0.014355293	-0.152003093	0.545968369	1.042644337
ERICH6	0.045043	0.477375566	-3.848920527	-4.811978949	-5.380821784	-4.651087759	-4.221623189	-3.19759996	-3.809954113	-3.070966521
ESPL1	0.007998	0.174583333	0.678071905	-5.035046947	-6.030324537	-5.153286059	1.700439718	0.918386234	1.339137385	0.941106311
EVA1C	0.021634	0.407809111	-1.369594529	-5.368849142	-1.899695094	-3.781900826	-1.023269779	-0.960159735	-1.347398782	-1.158429363
EVC2	0.028445	0.388953488	-3.279283757	-3.977099598	-5.194955239	-3.773984784	-2.300448367	-3.483581358	-2.035046947	-2.698997744
EYS	0.029221	3.082644628	-6.011587974	-4.432843996	-4.687799537	-4.362899876	-5.40506933	-6.068543859	-7.062746015	-8.708773666
EZH2	0.006209	0.433070866	4.837943242	3.165107985	3.336283388	4.209453366	5.469234794	5.017921908	5.325530332	5.157852169
FAAH2	0.043189	0.460992908	1.10433666	0.815575429	-1.227692025	-0.194294815	1.944858446	1.691534165	0.941106311	1.182692298
FABP5	0.006754	0.45229682	2.229587923	0.731183242	0.575312331	1.263034406	2.64385619	2.488000771	2.430285273	2.427606173
FAM110A	0.008241	0.466486486	0.464668267	-1.717856771	0.189033824	-0.671163536	0.98550043	0.98550043	0.748461233	0.82374936
FAM111A	0.001817	0.304878049	1.655351829	-0.454031631	-0.033569533	1.10433666	2.752748591	2.124328135	2.61117238	2.523561956
FAM187A	0.031867	0.477054429	-3.507993024	-6.41734766	-4.590744853	-4.725469955	-3.210896782	-3.651087759	-3.618827395	-3.23786383
FAM209A	0.014432	0.42194745	-5.24879339	-4.947862377	-4.974829424	-5.748553568	-4.519528055	-3.439089439	-3.859352207	-4.202903992
FAM83D	0.016365	0.207831325	0.35614381	-6.020925839	-4.938984225	-4.330610338	1.214124805	0.704871964	0.659924558	0.163498732
FANCA	0.009515	0.390446521	2.843983844	1.214124805	1.350497247	1.589763487	3.655351829	2.906890596	3.217230716	3.192194165
FANCD2	0.026838	0.263806971	1.5360529	-1.785875195	-1.498178735	-1.365871442	2.531069493	1.761285273	1.526068812	1.526068812
FANCG	0.046466	0.490178571	0.084064265	-2.224317298	-1.894321922	-0.614845103	0.545968369	0.111031312	-0.15682011	0.070389328
FANCI	0.042758	0.405311778	2.965322548	0.831877241	0.632268215	1.526068812	3.632268215	3.182692298	2.720278465	2.715893371
FAS	0.003712	2.272727273	3.797012978	4.070389328	3.689299161	3.632268215	2.432959407	3.350497247	2.017921908	2.310340121
FBLN1	0.035546	0.291428571	0.35614381	-3.046921047	-3.818070562	-2.680382066	1.028569152	0.475084883	0.097610797	0.150559677
FBN3	0.007438	0.290909091	-4.365871442	-4.816037165	-6.675949819	-4.869859865	-2.932361283	-3.337885669	-3.954557029	-2.785875195
FOXM1	0.026352	0.278797997	2.321928095	-0.492078535	-1.634867407	-0.606034724	3.099295204	2.648465443	2.310340121	2.053111336
FRMD4B	0.007271	0.442567568	3.523561956	2.046141782	2.416839742	2.397802962	4.217230716	3.807354922	3.797012978	3.689299161
FRMPD3	0.016848	0.454304636	-3.19759996	-4.874084451	-4.760235373	-3.412731032	-2.662003536	-3.251538767	-2.61705613	-2.506352666
GAS2L3	0.019401	0.423369565	0.35614381	-0.732164608	-0.98279071	-0.465938398	1.422233001	0.641546029	0.790772038	0.485426827
GATA1	0.012967	2.048681542	-3.421979109	-3.158429363	-3.10780329	-3.576905946	-4.383830534	-4.622376462	-3.564904848	-5.386845572
GDPD3	0.037904	0.466666667	-3.691522623	-4.348133165	-4.205563338	-3.035046947	-2.943416472	-3.307572802	-2.358453971	-2.171368418
GLDC	0.004829	0.424561404	-3.717856771	-5.24879339	-4.327710447	-4.583808806	-3.382325375	-3.400491764	-2.899695094	-2.910501849
GLDN	0.034501	13.48484848	-6.912672948	-5.695255342	-6.759453637	-4.865647613	-10.50111602	-8.789461512	-9.854752972	-9.655444164
GLIS3	0.032717	0.367741935	-3.307572802	-3.908334012	-3.10780329	-2.531156057	-2.19759996	-2.465938398	-1.095419565	-1.415037499
GLP2R	0.007066	2.666666667	-4.633076351	-4.23786383	-5.265344567	-4.874084451	-6.210896782	-6.493296513	-5.673002535	-6.200249538

GOLGA2P5	0.009904	0.485517241	-0.932361283	-1.722610301	-2.244685096	-1.434402824	-0.074000581	-0.66428809	-0.634867407	-0.567040593
GPER1	0.029664	0.326962457	-2.204233052	-3.956795501	-3.968040259	-4.736965594	-1.244685096	-1.502259911	-2.373327247	-2.279283757
GPIHBP1	0.026575	0.299319728	-5.221623189	-3.428177593	-5.112786697	-5.184424571	-2.64385619	-3.789860543	-2.158429363	-2.899695094
GPR83	0.001451	0.363070539	-7.767290131	-8.88172002	-8.347545629	-7.895394957	-6.767290131	-6.380821784	-6.64385619	-7.084119665
GRIK1	0.014726	2.46687054	-2.689659879	-1.977099598	-1.915935735	-1.756330919	-4.496549491	-4.161007907	-2.53951953	-3.058893689
GRIP2	0.017505	2.92022792	0.250961574	1.632268215	0.839959587	1.077242999	-0.962969269	-0.027674958	-0.325539348	-0.960159735
GRK5-IT1	0.047732	3.480392157	-5.10780329	-3.995390747	-5.965784285	-4.865647613	-5.895394957	-6.392894616	-7.480357457	-7.221623189
GTSE1	0.018431	0.213605442	0.084064265	-5.184424571	-4.293358943	-3.083141235	1.15704371	0.411426246	0.310340121	0.097610797
H2AFX	0.006245	0.415204678	1.464668267	-0.50021788	0.042644337	0.23878686	1.922197848	1.580145484	1.786596362	1.786596362
H2BFM	0.03831	0.368181818	-3.893249685	-4.874084451	-7.77675046	-3.496549491	-3.840629153	-2.965784285	-2.878321443	-2.358453971
HCLS1	0.042687	2.354609929	-6.179187923	-4.445362036	-4.857259828	-4.691522623	-5.680382066	-6.86648908	-5.748553568	-6.701247854
HELLS	0.022422	0.273049645	2.114367025	-0.614845103	-0.724992953	-0.828793173	3.080657663	2.430285273	2.097610797	2.150559677
HIST1H4E	0.046964	2.337016575	-1.498178735	-2.19759996	-1.020340448	-0.673462652	-1.988504361	-2.251538767	-2.343732465	-3.893249685
HJURP	0.00272	0.166236162	1.790772038	-4.100360306	-4.791857353	-4.28208783	2.632268215	2.163498732	2.462052319	2.456806149
HMGB2	0.014412	0.425225225	5.584962501	4.209453366	3.897240426	3.689299161	6.102238194	5.760220946	5.666756592	5.599912842
HMMR	0.044557	0.299293286	1.207892852	-1.318325858	-1.708396442	-1.415037499	2.150559677	1.641546029	0.748461233	1.063502942
HMMR-AS1	0.019062	0.347778982	-4.168771307	-5.117787378	-7.198129486	-4.748553568	-2.910501849	-3.72356289	-3.321928095	-4.058893689
HPR	0.001346	0.207612457	-0.98279071	-5.097887821	-4.853084152	-2.727379545	-0.375197235	-0.311148256	-0.13289427	-0.029146346
IFI16	0.019347	0.48206278	1.765534746	0.028569152	1.40053793	0.632268215	2.555816155	2.073820233	1.839959587	2.056583528
IL1R1	0.017849	0.463975155	-0.312939312	-1.231074664	-0.597277823	0.137503524	0.807354922	-0.077041036	0.807354922	0.98550043
IQANK1	0.018454	0.284501062	-5.448508591	-7.39775218	-7.477783514	-5.702749879	-4.522840789	-5.506352666	-3.936773198	-4.117787378
IQGAP3	0.00644	0.135041322	1.622930351	-3.412731032	-6.546245393	-3.578628567	2.693765712	1.678071905	3.025028794	2.673556424
IQSEC3	0.036875	2.756052142	-3.425075022	-3.251538767	-2.60823228	-2.120294234	-4.811978949	-4.542878542	-3.293358943	-4.840629153
ITGA10	0.024541	0.457974138	2.765534746	1.350497247	2.277984747	1.49057013	3.722466024	3.077242999	2.776103988	3.111031312
KCNAB3	0.01134	0.445269017	-2.120294234	-1.948975997	-1.988504361	-2.204233052	-1.190997225	-1.395928676	-0.420819852	-0.76121314
KCND2	0.029413	3.311111111	-4.912672948	-3.717856771	-4.618827395	-5.122805453	-5.66566056	-6.467533417	-6.559791925	-6.321928095
KCNK3	0.046457	2.3	2.22342255	2.786596362	1.5360529	1.963474124	-0.756330919	1.803227036	1.056583528	0.879705766
KCNQ5	0.009834	0.437333333	2.077242999	0.189033824	1.778208576	2.121015401	3.097610797	2.310340121	3.016139703	3.0721058
KIAA1211	0.014135	0.379310345	-0.98279071	-4.200249538	-1.680382066	-1.473931188	-0.592919225	-0.77349147	-0.106249498	0.097610797
KIF15	0.015496	0.273209549	1.531069493	-0.873027144	-1.36215794	-1.810966176	2.424922088	1.655351829	1.713695815	1.713695815
KIF18A	0.012563	0.265972222	0.111031312	-2.514573173	-2.145605322	-4.161007907	0.970853654	0.555816155	0.275007047	0.163498732
KIF18B	0.009602	0.34086629	1.910732662	-0.069451881	-0.089267338	0.650764559	2.843983844	2.053111336	2.381283373	2.23572706
KIF20A	0.017209	0.212045455	1.835924074	-4.380821784	-3.443791328	-5.310432456	2.568032105	2.22342255	1.941106311	1.655351829
KIF20B	0.003243	0.332520325	-0.386468347	-1.717856771	-1.76121314	-1.878321443	0.669026766	0.124328135	0.298658316	0.028569152
KIF22	0.007315	0.468263473	3.786596362	2.084064265	2.372952098	3.019701914	4.112700133	3.935459748	4.169925001	4.017921908
KIF23	0.020923	0.295918367	3.596935142	0.632268215	0.333423734	1.333423734	4.385431037	3.807354922	3.64385619	3.523561956
KIF2C	0.028255	0.273752013	2.493134922	-1.2968993	-1.785875195	-1.077041036	3.116031993	2.61117238	2.298658316	2.35614381
KIFC1	0.041909	0.214076246	2.503348735	-4.546245393	-4.625934282	-3.756330919	3.498250868	2.599317794	2.495695163	2.10433666
LAMA3	0.012031	0.23625498	-0.048412205	-1.926865295	-1.514573173	-0.3382504	1.097610797	0.333423734	1.831877241	1.627606838
LANCL1-AS1	0.027417	2.01754386	-3.531156057	-2.582079992	-3.279283757	-3.251538767	-3.558091636	-4.251538767	-4.348133165	-4.594225422
LARGE2	0.003934	0.449152542	-1.873027144	-1.857259828	-2.300448367	-1.698997744	-0.446148032	-1.043943348	-0.564904848	-1.110915901

LCN9	0.045257	2.312633833	-0.595096878	0.275007047	-0.336427665	0.713695815	-1.595096878	-0.490050854	-0.913216234	-1.77102743
LGSN	0.020144	3.043668122	-3.824188006	-3.606473968	-4.148161027	-3.840629153	-5.651087759	-3.983931631	-9.24331826	-7.175012247
LIF	0.021194	2.712264151	-0.55851652	0.641546029	-0.12973393	0.545968369	-2.046921047	-0.50021788	-1.268816758	-1.586405918
LPAR2	0.009847	0.393617021	-2.046921047	-2.126580497	-3.844768884	-2.314732593	-1.560642822	-1.282789701	-1.038006323	-0.632628934
LRP2	0.028144	2.808035714	-4.857259828	-3.886832943	-4.194955239	-3.397448103	-4.680382066	-5.368849142	-6.011587974	-6.559791925
LRRC25	0.046567	2.712328767	-4.13796526	-4.490050854	-5.658355759	-4.748553568	-8.162557248	-4.956795501	-6.978463419	-6.058893689
LRRC55	0.033842	2.571428571	-7.620955788	-7.210896782	-8.28771238	-6.838151005	-7.998615677	-9.909200756	-8.99493063	-8.739275755
LXN	0.016997	0.438345865	0.124328135	-1.486004021	-0.780908942	-1.698997744	0.739848103	0.5360529	0.189033824	0.070389328
LYNX1-SLURP2	0.005089	2.072463768	-2.052894948	-1.621488377	-1.514573173	-2.126580497	-2.426625474	-3.184424571	-3.058893689	-2.867752202
LYPD3	0.016424	0.340740741	-5.795859283	-6.573466862	-5.658355759	-7.206628451	-5.629500897	-4.684086035	-4.365871442	-4.19759996
MAP7D2	0.038445	0.478761062	-0.77102743	-1.522840789	-1.639354798	-0.1376478	0.275007047	-0.597277823	0.59454855	0.189033824
MCM7	0.025929	0.489104116	5.213347282	3.321928095	3.678071905	4.385431037	5.700439718	5.378511623	5.14974712	5.181897643
MCTP2	0.009141	3.485074627	-3.576905946	-2.932361283	-4.023269779	-3.361416385	-4.710283552	-5.695255342	-4.411195433	-8.620955788
MELK	0.046415	0.25	3.122672719	-0.979942348	-1.775959726	-1.020340448	4	3.40599236	2.698218478	2.811471031
MFAP4	0.010238	0.45	3.754887502	1.794935663	3.085764554	2.257010618	4.217230716	3.87774425	3.916476644	4.232660757
MGP	0.027707	0.351173709	0.855989697	-1.139235797	-0.997117491	-2.171368418	1.589763487	1.028569152	0.641546029	0.918386234
MIR3190	0.029934	2.513245033	-1.351074441	-0.325539348	-0.304006187	0.042644337	-2.184424571	-0.823677227	-1.915935735	-2.652901329
MIR616	0.039869	2.647798742	-2.040971781	-0.826232932	-1.905088353	-0.715485867	-3.19759996	-2.10159814	-2.60823228	-2.932361283
MKI67	0.011852	0.191413238	2.066950244	-6.310432456	-6.846428108	-4.268121651	2.944858446	2.260025656	2.414135533	2.182692298
MLXIPL	0.037723	0.483225806	-4.28771238	-3.046921047	-4.744680559	-3.473931188	-2.717856771	-2.53951953	-2.286304185	-3.435963338
MRPL23-AS1	0.045698	2.333333333	-1.910501849	-1.244685096	-1.816037165	-2.498178735	-2.19759996	-2.61705613	-3.846843212	-4.64385619
MSH5	0.042907	0.408679928	1.459431619	0.704871964	-0.020340448	1.871843649	2.140778656	1.5360529	2.82374936	2.948600847
MSH5-SAPCD1	0.045685	0.410714286	-0.522840789	-1.275786313	-1.846843212	-0.131313235	0.084064265	-0.434402824	0.879705766	0.963474124
MT2A	0.038014	0.287857143	-0.145605322	-3.184424571	-2.046921047	-1.49410907	0.028569152	-0.02180437	1.263034406	0.275007047
MTTP	0.045263	0.440643863	-5.615287038	-5.629500897	-5.411195433	-5.41734766	-3.625934282	-4.480357457	-4.532824877	-5.073393259
MUSK	0.048961	2.686131387	-7.254289378	-5.895394957	-5.179187923	-5.454822365	-7.066608654	-7.464982232	-6.64385619	-7.88172002
MXD3	0.013493	0.5	1.584962501	0.137503524	-0.109358756	0.669026766	1.786596362	1.622930351	1.761285273	1.709290636
MYO3B	0.001959	5.901639344	-3.10780329	-2.300448367	-2.070966521	-2.61705613	-6.011587974	-3.993091631	-5.006941609	-6.299027693
NCALD	0.017219	0.445238095	1.35614381	-1.671163536	0.678071905	1.580145484	2.22650853	1.613531653	2.260025656	2.09423607
NCAPG	0.036537	0.269767442	1.82374936	-1.407363571	-1.210896782	-1.826232932	2.746312766	2.13093087	1.700439718	1.521050737
NDC80	0.02203	0.213138686	0.150559677	-5.921390165	-6.998615677	-5.088040035	0.970853654	0.555816155	0.124328135	-0.03948829
NEFL	0.049071	2.044198895	0.275007047	0.632268215	-0.309359421	-0.189351252	-1.54793177	0	-1.332789088	-1.220950447
NKAIN3	0.034654	0.283367556	-11.32054377	-5.442222329	-6.519528055	-5.587272661	-5.216250017	-3.611755347	-4.654717182	-4.432843996
NRG1	0.000581	2.23495702	0.799087306	0.678071905	0.35614381	0.695993813	-0.932361283	-0.120294234	-0.448114897	-0.696657606
NRM	0.038252	0.489270386	2.735522177	0.014355293	1.378511623	1.773996325	3.147306699	2.724650272	2.634593268	2.646162657
NRXN3	0.034198	4.472972973	-1.365871442	-0.305788392	-1.569179503	0.163498732	-2.005782353	-3.613520111	-3.456405136	-2.564904848
NUDT1	0.02222	0.473394495	3.319039816	1.695993813	1.944858446	1.839959587	3.632268215	3.5360529	3.019701914	3.510961919
NUF2	0.009167	0.214137214	1.944858446	-3.40506933	-3.210896782	-4.027969116	2.543495883	2.140778656	2.166715445	2.176322773
NUSAP1	0.018061	0.220458554	5.523561956	0.286881148	0.310340121	0.475084883	6.321928095	5.744161096	5.590961241	5.491853096
NYAP2	0.017128	5.605536332	-7.514243452	-5.803896602	-5.912672948	-5.333516069	-8.013450718	-7.818477586	-9.764150423	-8.895394957
ODAM	0.014571	0.409090909	-1.486004021	-2.564904848	-2.450084446	-4.454822365	-0.785875195	-1.53951953	-1.177881725	-1.046921047

OLFML3	0.00911	0.381118881	0.124328135	-1.531156057	-1.473931188	-1.384583703	0.839959587	0.604071324	0.378511623	0.150559677
OR7E2P	0.02906	3.569767442	-4.857259828	-3.351074441	-4.717856771	-3.736965594	-6.179187923	-5.49980982	-5.594225422	-6.333516069
OTULINL	0.032314	0.481636936	-3.629500897	-5.423526235	-5.51292532	-4.115284871	-3.210896782	-3.070966521	-3.676687582	-3.712173133
P2RY14	0.011149	0.41038961	0.111031312	-2.351074441	-0.685013515	-0.675765438	0.925999419	0.59454855	0.111031312	0.748461233
PADI2	0.018678	0.409929078	-4.13289427	-3.961282892	-3.504714171	-5.506352666	-2.358453971	-3.10780329	-2.680382066	-3.386845572
PALD1	0.007909	0.49	-0.632628934	-1.418889825	-1.089267338	-0.582079992	0.50589093	-0.281035664	0.028569152	0.201633861
PAQR4	0.008474	0.44459279	2.584962501	0.722466024	1.207892852	1.739848103	3.084064265	2.639232163	2.835924074	3.023255352
PDE11A	0.031653	3.894967177	-4.03268381	-2.442222329	-2.418889825	-1.831357964	-5.566613191	-3.490050854	-5.078259014	-4.53951953
PHF19	0.037159	0.359722222	3.744161096	0.941106311	1.744161096	1.063502942	4.321928095	3.916476644	3.548436625	3.419538892
PIF1	0.009689	0.144656489	0.495695163	-7.294490912	-5.930160375	-3.647467443	1.292781749	0.443606651	1.887525271	1.560714954
PIMREG	0.010183	0.217928287	1.063502942	-4.470728756	-5.333516069	-5.097887821	1.50589093	1.3950628	1.163498732	1.232660757
PINLYP	0.027575	0.379790941	-0.646112164	-3.293358943	-1.231074664	-2.826232932	-0.454031631	0.298658316	-0.698997744	-0.210896782
PKMYT1	0.00767	0.339285714	2.568032105	0.275007047	0.070389328	1.682573297	3.419538892	2.765534746	3.0721058	2.944858446
PKN3	0.014493	0.375862069	0.722466024	-1.407363571	-0.377069649	0.632268215	1.438292852	0.82374936	1.922197848	1.739848103
PLCD4	0.016932	0.495151515	0.150559677	-1.023269779	-0.327348371	-0.202571918	0.59454855	0.124328135	1.03562391	0.970853654
PLEKHG4	0.024673	0.284539474	-1.727379545	-3.965784285	-2.857259828	-2.40354186	-1.2968993	-1.204233052	0	-0.76121314
PLG	0.005998	3.907056799	-5.921390165	-4.917024973	-5.448508591	-5.764150423	-7.388053353	-8.292227861	-6.573466862	-8.125824697
PLK1	0.005268	0.277777778	0.111031312	-3.388355457	-2.805912948	-1.805912948	0.739848103	0.321928095	0.495695163	0.526068812
POLE	0.016258	0.498595506	2.422233001	1.117695043	1.580145484	1.883620816	3.241840184	2.459431619	2.752748591	2.757023247
POLQ	0.031503	0.308602151	1.15704371	-0.727379545	-1.652901329	-1.795859283	2.107687869	1.263034406	1.189033824	1.10433666
PRC1	0.041961	0.280943026	5.50779464	1.608809243	2.176322773	1.989139007	6.266786541	5.752213368	5.296457407	5.06608919
PRC1-AS1	0.004811	0.242424242	-1.329159664	-5.803896602	-4.882570916	-4.01391678	-0.805912948	-1.035046947	-0.924125133	-0.924125133
PRKCQ	0.004465	0.373626374	-2.314732593	-1.994240731	-1.671163536	-2.418889825	-1.321928095	-0.397828209	-0.384583703	-0.680382066
PRKCQ-AS1	0.038671	0.419230769	-2.689659879	-5.60823228	-5.200249538	-4.097887821	-2.921390165	-2.336427665	-2.826232932	-2.698997744
PRKX	0.010047	2.36	-1.543719518	-0.623709617	-0.399730246	-0.713118852	-2.351074441	-1.671163536	-2.158429363	-1.910501849
PRSS3	0.005501	4.207048458	-3.35254733	-1.98279071	-2.358453971	-2.210896782	-3.899695094	-4.216250017	-5.173970214	-4.917024973
PRSS46P	0.018965	2.021834061	1.5360529	2.10433666	2.582556003	2.419538892	1.485426827	1.454175893	1.028569152	0.650764559
PSMB8	0.019217	0.292923077	-2.046921047	-3.988504361	-5.068543859	-4.442222329	-1.200912694	-1.332789088	-2.13289427	-2.052894948
PSRC1	0.006517	0.204666667	3.336283388	-0.510457064	-0.968604804	-0.128156351	4.177917792	3.378511623	3.827819025	4.10433666
PTGDS	0.03996	0.323333333	0.263034406	-1.994240731	-0.857259828	-0.488026018	0.367371066	0.367371066	1.286881148	1.761285273
PTTG1	0.003248	0.169198312	3.906890596	-3	-1.358453971	-0.991369695	4.478971805	4.247927513	4.812498225	4.66106548
PYHIN1	0.007324	2.142222222	-4.359934417	-4.503077534	-4.293358943	-4.354021725	-5.10780329	-6.002310161	-4.775959726	-6.838151005
RAB6C-AS1	0.047569	2.053475936	-5.216250017	-5.083141235	-4.40812913	-4.319045586	-5.063710705	-5.454822365	-5.869859865	-7.606825459
RACGAP1	0.0323	0.287755102	4.465974465	1.622930351	1.007195501	0.042644337	5.121015401	4.672425342	4.277984747	4.193771743
RASSF2	0.024969	0.460869565	-3.783886642	-3.932361283	-2.775959726	-2.846843212	-2.046921047	-2.836501268	-1.666576266	-2.171368418
RECQL4	0.005148	0.349659864	3.152183419	0.790772038	1.613531653	2.786596362	4.153805336	3.419538892	3.916476644	3.906890596
RHBDL3	0.025999	0.365957447	-2.977099598	-3.321928095	-3.801883071	-1.354759487	-0.929610672	-1.184424571	-1.76611194	-0.685013515
RHEBL1	0.031375	0.476129032	-3.35254733	-5.097887821	-3.986216185	-3.251538767	-2.717856771	-3.070966521	-2.171368418	-2.965784285
RIPOR2	0.048816	2.276422764	2.211012193	3.472487771	4.044394119	3.666756592	1.613531653	2.292781749	2.575312331	2.526068812
RIPPLY1	0.041865	0.272563177	-6.559791925	-7.045490984	-6.030324537	-5.205563338	-4.844768884	-4.176576709	-3.351074441	-4.895394957
RLN1	0.030043	0.417931034	-3	-6.88172002	-3.544560985	-5.429731384	-2.795859283	-2.531156057	-3.13289427	-2.756330919

RNASEH2A	0.032521	0.460176991	2.007195501	0.286881148	0.495695163	0.731183242	2.565597176	2.333423734	1.739848103	1.914564523
RSPH10B	0.021732	0.386060606	-4.563198526	-3.742747947	-4.075824085	-3.669326877	-2.139235797	-2.932361283	-3.426625474	-2.258425153
RSPH10B2	0.037505	0.460897436	-4.316168826	-3.095419565	-4.48681248	-3.727379545	-2.19759996	-2.76611194	-3.503077534	-2.53951953
RTKN2	0.046509	0.313913043	0.014355293	-3.035046947	-3.083141235	-2.40354186	0.86393845	-0.121863233	0.111031312	-0.329159664
SAPCD2	0.003321	0.181208054	-1.10159814	-7.152259595	-5.28771238	-4.60823228	-0.215914857	-0.76121314	-0.220950447	-0.573466862
SCN2A	0.012239	5.073298429	-3.965784285	-3.914847319	-3.23786383	-2.727379545	-4.764150423	-5.221623189	-9.726997425	-6.429731384
SCN4B	0.038013	2.330645161	1.292781749	2.13093087	1.531069493	0.887525271	-0.084670324	1.124328135	-0.134477041	-0.10780329
SCTR	0.034654	4.405594406	-3.265344567	-2.481968507	-4.563198526	-2.465938398	-7.305859726	-4.130365444	-4.429731384	-7.720897226
SERTAD4	0.019646	0.334975369	-1.905088353	-5.148161027	-3.307572802	-2.756330919	-0.708396442	-1.685013515	-1.560642822	-1.461958547
SFRP5	0.043416	0.429718876	-2.077041036	-5.687799537	-2.888968688	-4.857259828	-1.722610301	-2.023269779	-2.411195433	-1.948975997
SGCD	0.039277	2.080717489	-1.268816758	0.163498732	0.286881148	-0.064917477	-1.092340172	-0.946193556	-1.560642822	-1.13606155
SGSM1	0.003839	0.339892665	-1.639354798	-3.23786383	-2.556393349	-2.634867407	-0.800877358	-1.446148032	-0.662003536	-0.590744853
SHISAL1	0.046891	3.316062176	-1.286304185	0.042644337	-0.314732593	-1.675765438	-2.988504361	-1.411195433	-2.988504361	-2.805912948
SHMT1	0.021456	0.40201005	2.411426246	0.59454855	0.59454855	0.344828497	2.891419187	2.682573297	2.17951105	2.462052319
SKA1	0.027234	0.351886792	-0.131313235	-2.358453971	-2.795859283	-2.058893689	0.5360529	0.214124805	-0.231074664	-0.352915787
SKA3	0.028787	0.231617647	0.250961574	-5.974829424	-8.342853934	-4.279283757	0.963474124	0.5360529	0.111031312	-0.046921047
SLAMF7	0.011992	0.449685535	-3.478748205	-6.656899227	-3.373327247	-3.472329084	-2.531156057	-2.805912948	-2.932361283	-2.395928676
SLC15A2	0.024364	0.429522752	-0.440263476	-2.689659879	-1.573466862	-1.639354798	0.310340121	-0.356605547	-0.541617996	-0.150400989
SLC16A9	0.037889	0.496363636	2.709290636	1.35614381	2.084064265	3.087462841	3.231125158	2.843983844	3.744161096	3.807354922
SLC24A3	0.009398	2.927777778	-1.454031631	-0.498178735	-0.659722595	-1.318325858	-3.590744853	-1.943416472	-2.418889825	-2.395928676
SLC27A3	0.010314	0.426829268	0.839959587	-0.888968688	-0.4639471	0.176322773	1.23878686	0.82374936	1.589763487	1.432959407
SLC43A3	0.009578	0.27076412	-2.300448367	-4.938984225	-4.706511798	-4.259806383	-1.254977851	-2.139235797	-1.722610301	-1.971430848
SLC6A6	0.033736	3.084507042	1.678071905	3.432959407	2.790772038	2.430285273	0.941106311	1.5360529	1.117695043	0.622930351
SLC7A9	0.021818	0.337801609	-4.921390165	-7.736196362	-7.228097523	-7.361712961	-5.13289427	-4.48681248	-4.48681248	-4.983931631
SLCO2A1	0.040592	0.412244898	-4.988504361	-5.423526235	-8.117787378	-5.480357457	-3.895394957	-5.112786697	-4.74081792	-3.997693533
SLFN11	0.003767	0.288118812	-0.910501849	-2.506352666	-2.756330919	-1.694321257	0.464668267	-0.30222618	0.028569152	-0.256700472
SLFN13	0.006076	0.37755102	2.655351829	1.286881148	0.757023247	1.526068812	3.485426827	2.885574364	3.119356177	2.992768431
SMC4	0.025695	0.438071066	4.087462841	2.397802962	2.639232163	2.601696516	4.754887502	4.217230716	4.053111336	4.03562391
SNORA11G	0.033694	0.286413043	-5.063710705	-3.842697534	-3.921390165	-4.470728756	-2.217591435	-1.727379545	-3.046921047	-3.368849142
SPAG5	0.01497	0.266433566	0.150559677	-3.853084152	-3.680382066	-1.921390165	0.963474124	0.454175893	0.333423734	0.150559677
SPATA46	0.03504	2.319672131	-1.486004021	-2.145605322	-1.354759487	-2.662003536	-2.634867407	-2.671163536	-3.251538767	-3.963531833
SSX4	0.004237	3.264	0.23878686	1.077242999	1.13093087	1.422233001	-0.727379545	-0.506352666	-0.932361283	-0.582079992
SSX4B	0.002916	3.962848297	-0.430508908	0.516015147	0.40053793	0.704871964	-1.621488377	-1.152003093	-2.434402824	-1.590744853
SSX5	0.006334	4.14953271	-2.279283757	-0.833927324	-0.913216234	-1.064917477	-2.867752202	-3.184424571	-3.279283757	-3.717856771
SSX6	0.007612	3.046052632	-1.932361283	-1.117161344	-1.029146346	-0.648371671	-3.023269779	-2.434402824	-2.965784285	-2.531156057
SUSD2	0.044472	0.444897959	-2.19759996	-5.13289427	-3.511279347	-3.279283757	-1.612637459	-2.171368418	-1.883635243	-2.662003536
SYNPR	0.025032	5.10326087	-0.450084446	0.641546029	0.042644337	-1.210896782	-2.473931188	-1.272297327	-3.633076351	-4.085588556
TESMIN	0.03222	0.438016529	-0.531156057	-3.145605322	-1.921390165	-2.300448367	-0.241270432	-0.241270432	-0.623709617	-0.828793173
TIAM1	0.003354	0.491525424	0.748461233	-0.10159814	-0.166502663	0.176322773	1.500802053	1.021479727	1.304511042	1.084064265
TMEFF2	0.029039	2.923387097	-3.279283757	-3.59596886	-2.564904848	-2.152003093	-4.319045586	-4.899695094	-3.807932116	-4.526161147
TMEM176A	0.005172	0.320560748	0.084064265	-0.582079992	-0.657445255	-1.384583703	0.790772038	0.659924558	1.220329955	1.550900665

TMEM176B	0.037223	0.479532164	2.565597176	1.028569152	1.555816155	1.137503524	2.448900951	2.283921772	3.148934105	3.030336078
TMEM220-AS1	0.004483	0.358203125	-2.60823228	-3.754382647	-3.443791328	-4.748553568	-2.177881725	-1.603840511	-2.210896782	-1.948975997
TMEM236	0.003389	0.11097561	-8.854752972	-7.63809692	-9.305859726	-8.848089242	-4.828280761	-6.189680297	-5.153286059	-5.546245393
TMSB15A	0.015925	0.23814433	0.765534746	-5.189680297	-3.945637712	-4.232429944	1.23878686	0.992768431	0.918386234	0.622930351
TMSB15B	0.007054	0.480769231	2.084064265	1.028569152	0.871843649	0.918386234	2.440952198	2.275007047	2.592158002	2.166715445
TNFAIP8L1	0.01041	0.428415301	-0.533242384	-2.921390165	-1.756330919	-1.158429363	-0.171368418	-0.473931188	-0.067938829	0.137503524
TNFRSF10C	0.030324	2.220496894	0.163498732	0.918386234	0.765534746	0	-0.833927324	0.201633861	-1.469929258	-0.985644707
TNS2	0.010631	0.488465396	2.666756592	1.580145484	2.35614381	2.321928095	3.364572432	2.704871964	3.459431619	3.596935142
TNXA	0.015794	0.45412844	-4.262572817	-4.083141235	-5.083141235	-4.125321051	-2.756330919	-3.899695094	-3.210896782	-3.13289427
TNXB	0.007735	0.456617647	-3.770042991	-4.115284871	-4.546245393	-3.746615764	-2.490050854	-3.409661467	-2.867752202	-2.899695094
TP63	0.023229	6.776923077	-4.706511798	-2.689659879	-3.853084152	-3.472329084	-5.702749879	-7.543551284	-5.820106829	-6.698248487
TPRG1	0.033701	3.041401274	-4.576905946	-3.224317298	-3.023269779	-3.184424571	-5.24331826	-3.760235373	-5.803896602	-7.060818566
TPX2	0.028016	0.230357143	3.285402219	-2.746615764	-2.481968507	-2.10780329	4.008988783	3.523561956	3.217230716	2.9800253
TRAIP	0.013863	0.393220339	0.545968369	-2.029146346	-1.395928676	-0.522840789	1.150559677	0.739848103	0.687060688	0.650764559
TRIM22	0.041009	2.219409283	-1.790858602	-0.564904848	-1.293358943	-0.454031631	-2.380821784	-1.703689439	-1.821126042	-2.582079992
TRIP13	0.039318	0.318367347	0.298658316	-2.625934282	-2.005782353	-2.145605322	1.189033824	0.516015147	0.163498732	0.070389328
TRPM8	0.01186	4.780487805	-4.636660688	-3.738890471	-4.861447625	-3.542878542	-6.587272661	-5.930160375	-5.803896602	-7.767290131
TSPAN11	0.007908	2.229166667	1.541019153	1.769771739	1.959770155	1.40599236	0.464668267	1.304511042	-0.254977851	0.111031312
UBA7	0.009656	0.432712215	2.582556003	0.622930351	1.765534746	2.531069493	3.19061486	2.809414444	3.498250868	3.485426827
UBE2C	0.004159	0.183035714	4	-2.698997744	-5.28208783	-2.314732593	4.655351829	4.371558863	4.432959407	4.478971805
UBE2T	0.024032	0.362135922	3.173127433	1.257010618	1.182692298	0.321928095	3.733354341	3.560714954	3.037382222	3.019701914
USH1C	0.013829	0.481528662	2.693765712	2.111031312	0.82374936	1.35614381	3.250961574	2.859969548	2.851998837	2.887525271
VAT1L	0.007394	3.096446701	-0.380821784	0.378511623	0.773996325	0.124328135	-1.790858602	-0.823677227	-1.258425153	-1.713118852
VWA2	0.027187	2.946666667	-4.733123528	-3.797864419	-4.997693533	-4.799872346	-5.912672948	-7.424765132	-5.40506933	-6.148161027
WDFY4	0.013347	5.813559322	-6.232429944	-4.380821784	-4.457989644	-5.044538396	-7.39775218	-6.751659479	-7.18337572	-9.371235735
WDR63	0.021813	2.356973995	-0.395928676	0.565597176	-0.057391664	-0.3382504	-1.227692025	-0.592919225	-1.59946207	-1.888968688
XRCC4	0.003886	2.411764706	4.292781749	4.649615459	4.754887502	4.733354341	3.204766751	4.169925001	2.731183242	2.767654798
YBX2	0.011393	0.414239482	1.021479727	-0.036525876	0.084064265	0.070389328	1.275007047	1.195347598	2.025028794	1.835924074
ZNF385B	0.021707	3.336700337	2.298658316	3.95419631	3.459431619	3.046141782	1.310340121	1.90303827	1.678071905	1.292781749
ZNF833P	0.012187	0.351123596	-3.023269779	-5.787866492	-5.058893689	-3.660178495	-2.573466862	-3.046921047	-2.177881725	-2.314732593
ZRANB2-AS2	0.037025	0.438356164	-7.640973681	-4.423526235	-4.695255342	-4.729291666	-3.473931188	-3.414268267	-3.886832943	-4.625934282
