# Supplementary material for: Transcriptomic profiling reveals p53 as a key regulator of doxorubicin-induced cardiotoxicity
Source: Cell Death Discov. 2019 Jun 12;5:102. doi: 10.1038/s41420-019-0182-6 (PMC6561911; doi:10.1038/s41420-019-0182-6)
Supplement: Supplementary file 4 — Supplemental Table 3-part 2 [file 41420_2019_182_MOESM4_ESM.pdf]

GO Clusters-upregulated genes

Annotation Cluster 1 — Enrichment Score: 1.295225630723447

Category	Term	Number of genes in cluster	PValue	Genes
GOTERM_BP_DIRECT	GO:0050731~positive regulation of peptidyl-tyrosine phosphorylation	4	0.00344986	LIF, GATA1, HCLS1, NRG1
GOTERM_BP_DIRECT	GO:0045944~positive regulation of transcription from RNA polymerase II promoter	7	0.152696587	LIF, GATA1, AHRR, HCLS1, TP63, ABRA, NRG1
GOTERM_BP_DIRECT	GO:0008284~positive regulation of cell proliferation	4	0.246997393	LIF, HCLS1, GLP2R, NRG1

Annotation Cluster 2 — Enrichment Score: 1.2941282612173626

Category	Term	Number of genes in cluster	PValue	Genes
GOTERM_BP_DIRECT	GO:1903507~negative regulation of nucleic acid-templated transcription	5	2.67E-05	SSX4B, SSX6, SSX4, SSX5, TRIM22
GOTERM_MF_DIRECT	GO:0003714~transcription corepressor activity	5	0.005515869	SSX4B, SSX6, SSX4, SSX5, TRIM22
GOTERM_MF_DIRECT	GO:0003676~nucleic acid binding	5	0.453965333	SSX4B, SSX6, ZNF385B, SSX4, SSX5
GOTERM_BP_DIRECT	GO:0006355~regulation of transcription, DNA-templated	7	0.484726413	SSX4B, AHRR, HCLS1, SSX6, SSX4, SSX5, TRIM22
GOTERM_BP_DIRECT	GO:0006351~transcription, DNA-templated	8	0.593555371	SSX4B, AHRR, SSX6, TP63, ABRA, SSX4, SSX5, TRIM22
GOTERM_CC_DIRECT	GO:0005634~nucleus	16	0.894270617	XRCC4, HCLS1, TP63, TRIM22, PRKX, GATA1, SSX4B, CDKN1A, AHRR, SSX6, HIST1H4E, ZNF385B, SSX4, FAS, SSX5, NRG1

Annotation Cluster 3 — Enrichment Score: 0.6496459524906847

Category	Term	Number of genes in cluster	PValue	Genes
GOTERM_CC_DIRECT	GO:0005667~transcription factor complex	3	0.147322436	GATA1, HCLS1, TP63
GOTERM_BP_DIRECT	GO:0045944~positive regulation of transcription from RNA polymerase II promoter	7	0.152696587	LIF, GATA1, AHRR, HCLS1, TP63, ABRA, NRG1
GOTERM_BP_DIRECT	GO:0000122~negative regulation of transcription from RNA polymerase II promoter	4	0.499992998	GATA1, AHRR, HCLS1, TP63

GO Clusters-downregulated genes

Annotation Cluster 1 Enrichment Score: 6.731199849155738

Category	Term	Number of genes in cluster	PValue	Genes
GOTERM_CC_DIRECT	GO:0000775~chromosome, centromeric region	10	1.20E-08	KIF2C, MKI67, CENPA, HJURP, NUF2, CENPF, CENPW, CENPE, NDC80, HELLS
GOTERM_CC_DIRECT	GO:0000777~condensed chromosome kinetochore	10	5.14E-07	KIF2C, HJURP, SPAG5, RASSF2, NUF2, BUB1, CENPW, BUB1B, CENPE, NDC80
GOTERM_BP_DIRECT	GO:0007059~chromosome segregation	9	1.04E-06	HJURP, SPAG5, NUF2, CENPF, SKA3, CENPW, CENPE, NDC80, SKA1

Annotation Cluster 2 Enrichment Score: 6.253025943142817

Category	Term	Number of genes in cluster	PValue	Genes
KEGG_PATHWAY	hsa04110:Cell cycle	15	2.74E-11	E2F1, PKMYT1, CDC20, ESPL1, PTTG1, CDC25C, CDC25B, CCNB1, CCNB2, MCM7, PLK1, CDKN2D, BUB1, BUB1B, CCNA1
GOTERM_BP_DIRECT	GO:0000086~G2/M transition of mitotic cell cycle	12	4.96E-07	CCNB1, CCNB2, PLK1, FOXM1, BORA, TPX2, PKMYT1, USH1C, CDC25C, MELK, CDC25B, HMMR
KEGG_PATHWAY	hsa04114:Oocyte meiosis	10	2.19E-06	ADCY3, CCNB1, CCNB2, PLK1, BUB1, PKMYT1, CDC20, ESPL1, PTTG1, CDC25C
KEGG_PATHWAY	hsa04914:Progesterone-mediated oocyte maturation	9	3.25E-06	ADCY3, CCNB1, CCNB2, PLK1, BUB1, PKMYT1, CCNA1, CDC25C, CDC25B
GOTERM_BP_DIRECT	GO:0051726~regulation of cell cycle	8	5.59E-04	CCNB1, CCNB2, PLK1, FOXM1, CCNF, CENPF, PKMYT1, CDC25C

Annotation Cluster 3 Enrichment Score: 6.179230721294134

Category	Term	Number of genes in cluster	PValue	Genes
GOTERM_MF_DIRECT	GO:0008017~microtubule binding	19	1.89E-11	KIF23, GAS2L3, KIF22, KIFC1, PRC1, PSRC1, KIF15, KIF18A, DPYSL5, NUSAP1, KIF18B, CENPE, RACGAP1, FAM83D, PLK1, TIAM1, KIF20B, SKA1, KIF20A
SMART	SM00129:KISc	10	1.78E-09	KIF23, KIFC1, KIF22, KIF2C, KIF15, KIF18A, KIF20B, KIF18B, CENPE, KIF20A
GOTERM_MF_DIRECT	GO:0003777~microtubule motor activity	11	1.94E-08	KIF23, KIFC1, KIF22, KIF2C, DNALI1, KIF15, KIF18A, KIF20B, KIF18B, CENPE, KIF20A
GOTERM_BP_DIRECT	GO:0007018~microtubule-based movement	11	2.81E-08	KIF23, KIFC1, KIF22, KIF2C, KIF15, KIF18A, KIF20B, KIF18B, CENPE, RACGAP1, KIF20A
GOTERM_CC_DIRECT	GO:0005871~kinesin complex	9	1.15E-07	KIF23, KIFC1, KIF22, KIF2C, KIF18A, KIF20B, KIF18B, CENPE, KIF20A
GOTERM_CC_DIRECT	GO:0005874~microtubule	16	2.10E-06	KIF23, GAS2L3, KIF22, KIFC1, KIF15, KIF18A, TPX2, NUSAP1, KIF18B, CENPE, RACGAP1, KIF2C, DNAI1, TIAM1, KIF20B, KIF20A
GOTERM_BP_DIRECT	GO:0007080~mitotic metaphase plate congression	7	3.58E-06	CCNB1, KIFC1, KIF22, KIF2C, PSRC1, KIF18A, CENPE
GOTERM_BP_DIRECT	GO:0006890~retrograde vesicle-mediated transport, Golgi to ER	7	3.52E-04	KIF23, KIF22, KIF2C, KIF15, KIF18A, CENPE, RACGAP1

GOTERM_BP_DIRECT	GO:0019886~antigen processing and presentation of exogenous peptide antigen via MHC class II	7	6.52E-04	KIF23, KIF22, KIF2C, KIF15, KIF18A, CENPE, RACGAP1
GOTERM_MF_DIRECT	GO:0016887~ATPase activity	8	0.004443	KIF23, KIFC1, KIF22, KIF2C, KIF15, KIF20B, CENPE, KIF20A

Annotation Cluster 4 Enrichment Score: 2.3297790654315897

Category	Term	Number of genes in cluster	PValue	Genes
GOTERM_BP_DIRECT	GO:0031145~anaphase-promoting complex-dependent catabolic process	8	3.31E-05	CCNB1, PLK1, BUB1B, CDC20, PTTG1, AURKB, UBE2C, PSMB8
GOTERM_BP_DIRECT	GO:0051437~positive regulation of ubiquitin-protein ligase activity involved in regulation of mitotic cell cycle transition	6	0.001777	CCNB1, PLK1, BUB1B, CDC20, UBE2C, PSMB8
GOTERM_BP_DIRECT	GO:0051439~regulation of ubiquitin-protein ligase activity involved in mitotic cell cycle	4	0.002202	CCNB1, PLK1, CDC20, UBE2C
GOTERM_BP_DIRECT	GO:1904668~positive regulation of ubiquitin protein ligase activity	3	0.004441	PLK1, CDC20, UBE2C
GOTERM_BP_DIRECT	GO:0042787~protein ubiquitination involved in ubiquitin-dependent protein catabolic process	7	0.008344	CCNB1, PLK1, BUB1B, CDC20, PTTG1, AURKB, UBE2C
GOTERM_BP_DIRECT	GO:0051436~negative regulation of ubiquitin-protein ligase activity involved in mitotic cell cycle	5	0.00887	CCNB1, BUB1B, CDC20, UBE2C, PSMB8
GOTERM_CC_DIRECT	GO:0005680~anaphase-promoting complex	3	0.026547	BUB1B, CDC20, UBE2C
GOTERM_BP_DIRECT	GO:0043161~proteasome-mediated ubiquitin-dependent protein catabolic process	5	0.203279	BUB1B, CDC20, UBE2C, PSMB8, GTSE1

Annotation Cluster 5 Enrichment Score: 2.2355060989369635

Category	Term	Number of genes in cluster	PValue	Genes
GOTERM_BP_DIRECT	GO:0032508~DNA duplex unwinding	5	0.001573	RECQL4, DDX11, PIF1, BRIP1, DDX12P
SMART	SM00488:DEXDc2	3	0.002713	DDX11, BRIP1, DDX12P
SMART	SM00491:HELICc2	3	0.002713	DDX11, BRIP1, DDX12P
GOTERM_MF_DIRECT	GO:0004003~ATP-dependent DNA helicase activity	4	0.005317	MCM7, DDX11, BRIP1, DDX12P
GOTERM_BP_DIRECT	GO:0006139~nucleobase-containing compound metabolic process	3	0.107971	DDX11, BRIP1, DDX12P

Annotation Cluster 6 Enrichment Score: 1.398212370625287

Category	Term	Number of genes in cluster	PValue	Genes
GOTERM_BP_DIRECT	GO:0032508~DNA duplex unwinding	5	0.001573	RECQL4, DDX11, PIF1, BRIP1, DDX12P
GOTERM_MF_DIRECT	GO:0008026~ATP-dependent helicase activity	3	0.041144	RECQL4, DDX11, BRIP1
GOTERM_MF_DIRECT	GO:0003676~nucleic acid binding	6	0.987008	RECQL4, GLIS3, DDX11, ZNF833P, BRIP1, DPF1

Annotation Cluster 7 Enrichment Score: 1.1657389638109208

Category	Term	Number of genes in cluster	PValue	Genes
GOTERM_MF_DIRECT	GO:0005201~extracellular matrix structural constituent	5	0.006615	FBLN1, TNXB, COL15A1, MGP, FBN3
GOTERM_CC_DIRECT	GO:0005578~proteinaceous extracellular matrix	7	0.077978	FBLN1, TNXB, TNXA, COL15A1, MGP, ADAMTS10, FBN3
GOTERM_CC_DIRECT	GO:0031012~extracellular matrix	7	0.112458	FBLN1, TNXB, COL15A1, MGP, ADAMTS10, FBN3, MFAP4
SMART	SM00181:EGF	4	0.374568	FBLN1, LAMA3, TNXB, FBN3

Annotation Cluster 8 Enrichment Score: 0.756242851452252

Category	Term	Number of genes in cluster	PValue	Genes
GOTERM_MF_DIRECT	GO:0004672~protein kinase activity	9	0.048074	PRKCQ, PKN3, PLK1, RASSF2, BUB1, PKMYT1, BUB1B, AURKB, MELK
GOTERM_MF_DIRECT	GO:0004674~protein serine/threonine kinase activity	8	0.127979	PRKCQ, PKN3, PLK1, BUB1, PKMYT1, BUB1B, AURKB, MELK
SMART	SM00220:S_TKc	7	0.239721	PRKCQ, PKN3, PLK1, BUB1, PKMYT1, AURKB, MELK
GOTERM_BP_DIRECT	GO:0006468~protein phosphorylation	8	0.267415	PKN3, PLK1, RASSF2, BUB1, PKMYT1, BUB1B, AURKB, CDC25B
GOTERM_BP_DIRECT	GO:0018105~peptidyl-serine phosphorylation	3	0.419605	PRKCQ, PKN3, PLK1

Annotation Cluster 9 Enrichment Score: 0.6151135323044693

Category	Term	Number of genes in cluster	PValue	Genes
SMART	SM00325:RhoGEF	3	0.187293	PLEKHG4, TIAM1, ARHGEF5
GOTERM_MF_DIRECT	GO:0005089~Rho guanyl-nucleotide exchange factor activity	3	0.211596	PLEKHG4, TIAM1, ARHGEF5
GOTERM_BP_DIRECT	GO:0035023~regulation of Rho protein signal transduction	3	0.236863	PLEKHG4, TIAM1, ARHGEF5
SMART	SM00233:PH	5	0.368995	PLEKHG4, TIAM1, ARHGEF5, RTKN2, PLCD4

Annotation Cluster 10 Enrichment Score: 0.5806850765777699

Category	Term	Number of genes in cluster	PValue	Genes
GOTERM_BP_DIRECT	GO:0006334~nucleosome assembly	4	0.155643	HMGB2, CENPA, H2AFX, H2BFM
GOTERM_CC_DIRECT	GO:0000786~nucleosome	3	0.27977	CENPA, H2AFX, H2BFM
GOTERM_MF_DIRECT	GO:0046982~protein heterodimerization activity	7	0.415923	CENPA, MLXIPL, CENPW, H2AFX, H2BFM, MTTP, SMC4

Annotation Cluster 11 Enrichment Score: 0.570957793206228

Category	Term	Number of genes in cluster	PValue	Genes
KEGG_PATHWAY	hsa04512:ECM-receptor interaction	4	0.064595	LAMA3, TNXB, ITGA10, HMMR
KEGG_PATHWAY	hsa04510:Focal adhesion	4	0.374521	LAMA3, TNXB, ITGA10, ACTN3
GOTERM_BP_DIRECT	GO:0007155~cell adhesion	7	0.428354	LAMA3, TNXB, COL15A1, ITGA10, SLAMF7, MFAP4, CDH5
KEGG_PATHWAY	hsa04151:PI3K-Akt signaling pathway	5	0.501987	LAMA3, TNXB, PKN3, ITGA10, LPAR2

Annotation Cluster 12 Enrichment Score: 0.3743136906180636

Category	Term	Number of genes in cluster	PValue	Genes
GOTERM_BP_DIRECT	GO:0051056~regulation of small GTPase mediated signal transduction	4	0.198089	DEPDC7, TIAM1, ARHGAP11A, RACGAP1
GOTERM_MF_DIRECT	GO:0005096~GTPase activator activity	4	0.603708	DEPDC7, SGSM1, ARHGAP11A, RACGAP1
GOTERM_BP_DIRECT	GO:0043547~positive regulation of GTPase activity	7	0.630046	DEPDC7, ODAM, ARHGEF5, EZH2, DENND2A, ARHGAP11A, RACGAP1

Annotation Cluster 13 Enrichment Score: 0.23421969495897535

Category	Term	Number of genes in cluster	PValue	Genes
GOTERM_BP_DIRECT	GO:0045892~negative regulation of transcription, DNA-templated	9	0.213011	E2F1, HMGB2, FOXM1, EZH2, MLXIPL, CENPF, RIPPLY1, IFI16, MXD3
GOTERM_MF_DIRECT	GO:0008134~transcription factor binding	5	0.389699	E2F1, HMGB2, MLXIPL, CENPF, IFI16
GOTERM_BP_DIRECT	GO:0000122~negative regulation of transcription from RNA polymerase II promoter	10	0.440718	E2F1, GLIS3, PHF19, PLK1, FOXM1, EZH2, MLXIPL, RIPPLY1, IFI16, AURKB
GOTERM_BP_DIRECT	GO:0045893~positive regulation of transcription, DNA-templated	7	0.539337	E2F1, HMGB2, FOXM1, PSRC1, CKS2, MLXIPL, ARNTL2
GOTERM_BP_DIRECT	GO:0006357~regulation of transcription from RNA polymerase II promoter	6	0.570362	GLIS3, HMGB2, CKS2, MLXIPL, BRIP1, ARNTL2
GOTERM_MF_DIRECT	GO:0003700~transcription factor activity, sequence-specific DNA binding	9	0.845725	E2F1, HMGB2, FOXM1, DMRTC1, MLXIPL, DMRT2, ARNTL2, PTTG1, DMRTC1B
GOTERM_BP_DIRECT	GO:0045944~positive regulation of transcription from RNA polymerase II promoter	9	0.879145	E2F1, GLIS3, HMGB2, FOXM1, MLXIPL, DMRT2, ARNTL2, IFI16, GPER1
GOTERM_BP_DIRECT	GO:0006355~regulation of transcription, DNA-templated	12	0.962805	E2F1, PRKCQ, HMGB2, PHF19, DMRTC1, EZH2, MLXIPL, ARNTL2, PTTG1, DMRTC1B, YBX2, DPF1
GOTERM_BP_DIRECT	GO:0006351~transcription, DNA-templated	16	0.968035	E2F1, HMGB2, FOXM1, EZH2, MLXIPL, DMRT2, IFI16, MXD3, DPF1, PHF19, DDX11, DMRTC1, RIPPLY1, ARNTL2, DMRTC1B, HELLS

Annotation Cluster 14 Enrichment Score: 0.21891151372842288

Category	Term	Number of genes in cluster	PValue	Genes
KEGG_PATHWAY	hsa01200:Carbon metabolism	3	0.338013	SHMT1, ACSS1, GLDC
KEGG_PATHWAY	hsa01130:Biosynthesis of antibiotics	3	0.66359	SHMT1, ACSS1, GLDC
KEGG_PATHWAY	hsa01100:Metabolic pathways	8	0.982725	SHMT1, ACSS1, PTGDS, CYP21A2, POLE, PLCD4, ACSM5, GLDC
