# Supplementary material for: Transcriptomic profiling reveals p53 as a key regulator of doxorubicin-induced cardiotoxicity
Source: Cell Death Discov. 2019 Jun 12;5:102. doi: 10.1038/s41420-019-0182-6 (PMC6561911; doi:10.1038/s41420-019-0182-6)
Supplement: Supplementary file 5 — Supplemental Material File #1 [file 41420_2019_182_MOESM5_ESM.docx]

**Supplemental table 1**: Full list of dysregulated genes on day 7. The gene names, p-value, and fold change are listed on sheet 1 “All 1290 dysregulated genes on day 7”. DAVID clusters for upregulated genes on sheet 2 “GO Clusters-upregulated genes” and downregulated genes on sheet 3 “GO Clusters-downregulated genes”.

**Supplemental table 2**: List of genes regulated by TP53. The gene names, p-value “Expr p-value”, fold change “Expr Fold Change”, and cellular location are listed on sheet 1.

**Supplemental table 3**: Full list of dysregulated genes on day 14. The gene names, p-value, and fold change are listed on sheet 1 “All 315 dysreg. genes on day 14”. DAVID clusters for upregulated genes on sheet 2 “GO Clusters-upregulated genes” and downregulated genes on sheet 3 “GO Clusters-downregulated genes”.
